# Causal relationship between gut microbiome, plasma metabolites, inflammation, and aortic stenosis: A multi-omics Mendelian randomization analysis

**DOI:** 10.1097/MD.0000000000048238

**Published:** 2026-04-10

**Authors:** Yangyou Zhang, Guolin Zhang

**Affiliations:** a Department of Cardiology, The Second Hospital of Dalian Medical University, Dalian, Liaoning, China.

**Keywords:** aortic stenosis, gut microbiota, immune cells, inflammatory proteins, Mendelian randomization, plasma metabolites

## Abstract

As life expectancy increases and the population ages, aortic stenosis (AS) is the most common heart valve disease. Despite rapid improvements in interventional treatment options in recent years, morbidity and mortality from asymptomatic AS remain high. To date, there is no pharmacological therapy to prevent AS. In this study, we used multi-omics to systematically investigate potential causal association between the gut microbiome, human blood metabolites, inflammation and risk of AS, and search for potential biomarker for AS. Single-nucleotide polymorphisms associated with 207 gut microbiota, 1091 blood metabolites and the ratios of 309 metabolites, 731 immune cell phenotypes, 91 circulating inflammatory proteins, as exposures all were selected from recent large genome-wide association study and explored their causal association with AS using Mendelian randomization methods. We used the inverse variance weighted estimation method as the main method and other methods as supplementary methods. Mendelian randomization analysis has shown that 7 gut microbiota, 80 metabolites, 29 immune cells phenotypes, and 6 circulating inflammatory proteins are causally associated with AS. Co-localization analysis showed a significant correlation between 1-stearoyl-2-acryloyl-GPE levels and AS, with a *P*-value of .981 for posterior probability for hypothesis 4. In addition, metabolic pathway analysis revealed that the valine, leucine, and isoleucine biosynthesis (*P* = .0312) pathways were associated with AS. Four omics, including 207 gut microbiota, 1091 blood metabolites and the ratios of 309 metabolites, 731 immune cells, and 91 inflammatory proteins were used in this study to explain the causal relationship between multi-omics and AS.

## Graphical abstract

**Figure:**
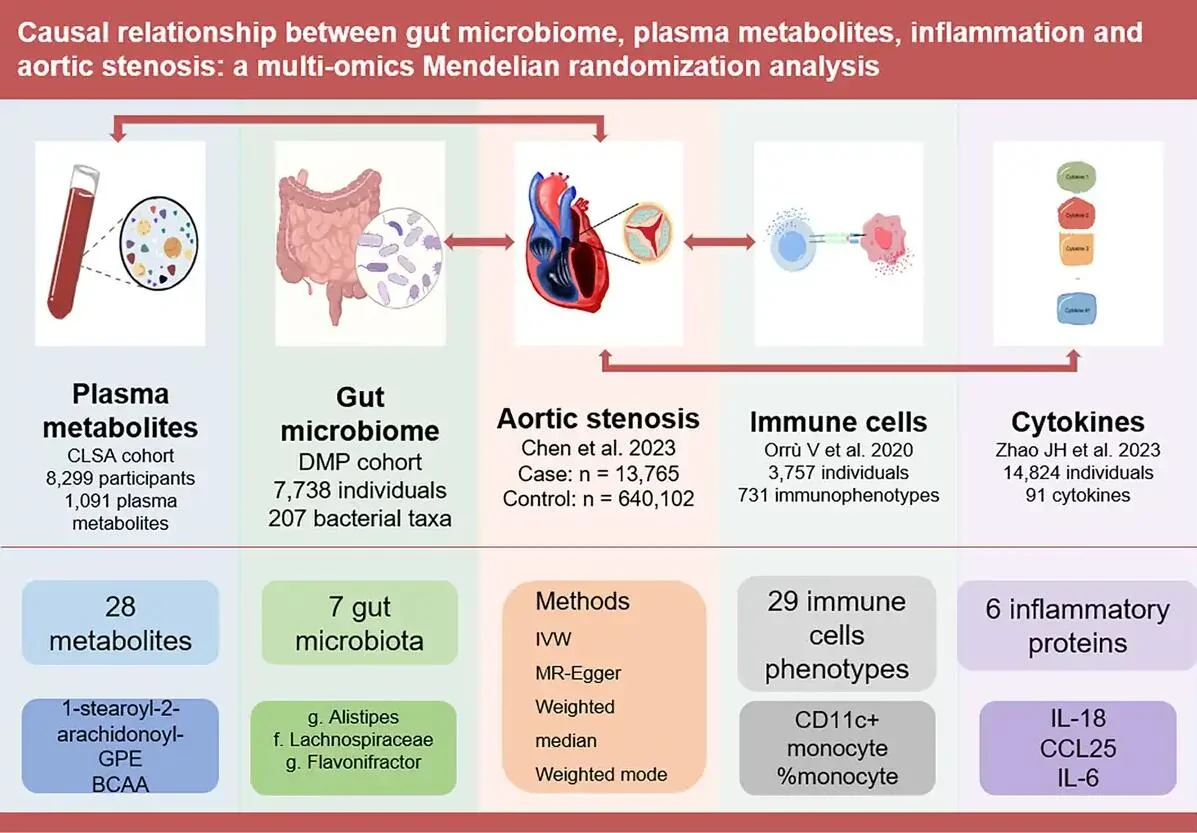

## 1. Introduction

Aortic stenosis (AS) is the most common form of valvular heart disease and is characterized by progressive fibrocalcific remodeling and thickening of the aortic valve cusps, leading to obstruction of the left ventricular outflow tract, and a trend exacerbated by population aging and increased prevalence of cardiovascular risk factors. The 2 major cell types involved in AS pathology are valvular interstitial cells (VICs) and valvular endothelial cells (VECs).^[1]^ VICs reside throughout the valve interstitium and are primarily responsible for maintaining the extracellular matrix (ECM) and structural integrity the valve.^[2]^ Upon pathological stimulation, VICs become activated, contributing to fibrosis and calcification, which are hallmarks of AS progression.^[3]^ VECs form a continuous layer covering the valve surfaces and play a critical role in valve homeostasis by mediating mechanotransduction and regulating inflammatory responses.^[4]^ Dysfunction of VECs, including loss of their protective properties and undergoing endothelial-to-mesenchymal transition, leads to increased inflammation, immune cell infiltration, and promotes VIC activation and osteogenic differentiation, driving AS development.^[5]^

Its pathogenesis is most commonly degenerative, with a prevalence of only 0.02% in subjects aged 18 to 44 years but a prevalence of 2.8% in patients older than 75 years.^[6]^ In addition to age and genetics, smoking, cholesterol levels, lipoprotein (a), diabetes mellitus, and obesity are factors that may increase the risk of AS, but the pathogenesis and causal pathways are not fully understood.^[7]^ An epidemiological survey showed that the prevalence of AS in people older than 75 years is 10% to 15% but is expected to more than double by 2040.^[8]^ Until now, there are no effective medical treatments for AS and many patients ultimately require surgical aortic valve replacement or transcatheter aortic valve replacement, for which costs have been estimated to be up to $120,000, which certainly adds to the burden of medical care.^[9,10]^ Although both modalities have been shown to be effective in treating AS, they are associated with various types of complications. Surgical aortic valve replacement may cause life-threatening or disabling bleeding, encephalopathy, atrial fibrillation, and acute kidney injury, whereas transcatheter aortic valve replacement may cause stroke, conduction disturbances, paravalvular leakage, and coronary artery overlap, all of which may prolong hospitalization and increase procedure-related readmission rates.^[11,12]^ Although AS shares several risk factors with cardiovascular disease, it remains unclear which factors are causative and which should be targeted to reduce valve disease.^[13,14]^ If the causative risk factors for AS can be identified, it may reveal new features for medical prevention and treatment of the disease.

Considering the shared pathogenic pathways between different types of cardiovascular disease,^[15,16]^ exploring the impact of other cardiovascular disease risk factors on AS is more conducive to finding its causative factors. Prior studies have suggested that calcific AS shares the common clinical risk factors with atherosclerotic disease.^[14]^ Relevant studies have shown that metabolic disorders, gut microbes, and inflammation are all significantly associated with atherosclerotic disease and that these risk factors interact with each other.^[17–22]^ Specifically, the effects of metabolites on cardiovascular disease are centered on lipid metabolism such as lipoproteins a, but there are also effects of glucose metabolism and branched-chain amino acid (BCAA) metabolism. The gut microbiota is a potential endocrine system that produces biologically active metabolites that enter the circulation and have a comprehensive impact on cardiac physiology and pathology. In addition to this, the effects of inflammation on AS were mainly demonstrated by tissue repair and tissue regeneration after injury by CCR2-macrophages in heart valves and by the ability of individual members of the TNF cytokine family to promote the expression of calcification markers in isolated valve mesenchymal stromal cells and vascular cells and to increase mineralization in cell cultures.^[23,24]^ Comprehensive studies have shown that the pathogenesis of AS is extremely complex and that multiple factors, including metabolomics, gut microbiota, inflammation, and genetics may combine to contribute to the progression of this disease.

It is important to recognize that previous studies have predominantly used a case–control study design, which poses a challenge in distinguishing exposure factors from outcomes. Furthermore, in observational studies, the association between blood metabolites, gut microbiota, inflammatory cells, inflammatory proteins, and AS may be affected by confounding factors such as age, environment, dietary patterns, and lifestyle, which limits the ability of our study to make causal inferences about the complex interactions between them and AS.

Mendelian randomization (MR) uses genetic variants, usually single-nucleotide polymorphisms (SNPs), as instrumental variables (IVs) for exposure. Genetic variants are randomly arranged and fixed during inheritance, largely independent of confounding factors, and do not change in response to disease progression. Thus, MR reduces potential methodological limitations such as confounding and reverse causality. MR has been widely used to study causality in a variety of diseases. We used MR methods to separately assess and identify common gut microbiomes, metabolites, immune cells, and inflammatory proteins that have causal effects on AS, and to identify metabolic pathways that may contribute to AS.

Previous studies have utilized MR to investigate causal associations of gut microbiota,^[25,26]^ circulating metabolites,^[27]^ immune cell,^[28]^ and inflammatory proteins with other diseases,^[29]^ but no relevant studies have utilized this methodology to reveal causal associations between these exposures and AS. Therefore, the present study investigated the causal associations between gut microbiota, circulating metabolites, immune cell, and inflammatory proteins with AS at the level of genetics.

## 2. Methods

### 2.1. Study overviews

We systematically assessed the causal relationship between gut microbiome, human circulating metabolites, immune cells, inflammatory proteins, and the risk of AS using a 2-sample MR design. A convincing MR study should fulfill 3 basic assumptions: that genetic variants are strongly associated with exposure; that they are not associated with confounders of the exposure–outcome relationship; and that they affect outcomes only through exposure and not through other causal pathways. Genetic variables for exposure and outcome were screened from independent genome-wide association study (GWAS) datasets separately to avoid sample overlap.

### 2.2. Data sources

The dates for exposure and outcome were listed in Table S1, Supplemental Digital Content, https://links.lww.com/MD/R607.

We selected summary data about 207 gut microbiota from a GWAS of 7738 individuals of European descent from the Dutch Microbiome Project.^[30]^ Data about 1091 blood metabolites and the ratios of 309 metabolites were obtained from a recent genomics study on the relationship between SNPs and the human metabolome, which was conducted by the Canadian Longitudinal Study of Aging cohort.^[31]^ Data on 731 immune cell phenotypes were extracted from a study with data from 3757 Europeans.^[32]^ Data on 91 circulating inflammatory proteins were obtained from a study involving 14,824 individuals.^[33]^ Summary statistics on AS were derived from a meta-analysis of 10 European cohorts.^[34]^ Complete description of each cohort and their case definition is available from the original study.

### 2.3. SNP selection

In order to obtain a sufficient number of SNPs for efficient analysis and to more comprehensively evaluate the association between multi-omics and AS, similar to previous studies, for SNPs associated with gut microbiota,^[25,35]^ metabolites,^[27]^ immune cell phenotypes,^[28]^ and circulating immune proteins,^[36]^ we used a threshold of *P* < 1 × 10^-5^ and demonstrate independent association linkage disequilibrium clumping *r*^2^ < 0.001 and kb < 10,000. Additionally, we used *P* < 5 × 10^−8^ as a threshold to select SNPs associated with polyomics and analyzed them again using the same methodology, but conditions using this threshold may not have been able to screen for sufficient IVs resulting in potential positive results being missed, and were therefore used as a secondary analytical outcome in this study.

In addition, we used we used a threshold of *P* < 1 × 10^−5^ and use the same approach to explore the causal relationship between the 2 of the identified gut flora, metabolites, immune cell characteristics, and inflammatory proteins in order to explore the potential relationship and mechanisms between them. The strength of the IVs was examined by using the *F* statistic to exclude the effect of weak instrument bias, and IVs with *F* < 10 will be excluded. *F* statistics were calculated using the following formula: *F* = *R*^2^ × (*N* − *k* − 1)/*k*(1 − *R*^2^), in which *R*^2^ represents the variance explained by the IVs, *k* represents the number of IVs, and *N* represents the sample size. *R*^2^ was estimated by MAF and β value, using the equation: *R*^2^ = 2 × MAF × (1 − MAF) × β^2^.^[37]^ Detecting confounders by using the Phenoscanner V2, and removing SNPs associated with both exposure and outcome.^[38]^

### 2.4. MR analysis

Multiple MR methods were used to assess the causal relationship between multi-omics and AS, including inverse variance weighting (IVW), MR-Egger, weighted median, and weighted mode. Similar to previous studies, we used the IVW approach as the primary MR analysis method,^[39,40]^ which assumes that IVs uniquely affect outcomes only through specific exposures, thus providing unbiased estimates of causality in the absence of horizontal pleiotropic effects.^[41]^ To exclude the effect of confounding factors, we performed sensitivity analyses using several methods: MR-Egger and MR- PRESSO^[42]^ was applied to estimate horizontal pleiotropy and Cochran *Q* test was used to validate the heterogeneity of IVs. In addition, *P*-value > .05 for Egger intercept (*P* for pleiotropy) and *P*-value > .05 for global test indicated that there was no horizontal pleiotropy. If outliers were found, the horizontal pleiotropy test was recalculated after removing the outliers using the MR-PRESSO method. Finally, results with a *P*-value of <.05 for IVW and passing the sensitivity test were considered as positive results.

### 2.5. Bayesian co‑localization analysis

Bayesian homoscedastic analysis is used to assess the likelihood that 2 features share the same causal variable. It provides posterior probabilities for 5 hypotheses: hypothesis 0 (PH0), hypothesis 1 (PH1), hypothesis 2 (PH2), hypothesis 3 (PH3), and hypothesis 4 (PH4). We validated the posterior probabilities of these hypotheses in co-localization analyses, which suggest that the relationship between metabolites and AS is achieved through distinct or shared variants in specific regions. The criterion for determining gene co-localization was a gene-based PPH4 score of more than 80%.^[43]^

### 2.6. Metabolic pathway analysis

Metabolic pathways were analyzed via the web-based Metaconflict 6.0.(https://www.metaboanalyst.ca/).^[44]^ The Kyoto Encyclopedia of Genes and Genomes database were used in this study, and the significance level for pathway analysis was set at 0.05.

### 2.7. Statistical analysis

Statistical analyses were performed using R software (version 4.3.0; R Foundation for Statistical Computing, Vienna, Austria). MR analyses were performed using the TwoSampleMR package. MR-PRESSO was performed using the MR-PRESSO package.

## 3. Results

### 3.1. Instrument variables

According to the predefined selection criteria for IVs, 1203 SNPs (*P* < 1 × 10^−5^) were regarded as IVs associated with 200 gut microbiota taxa in the Dutch Microbiome Project for AS. Similarly, based on previous studies, we identified 23,541 SNPs as IVs associated with 1091 blood metabolites and the ratios of 309 metabolites in the circulating metabolites for AS (*P* < 1 × 10^−5^)^[31]^; 10,669 SNPs for 731 immunophenotypes in the immune cells for AS (*P* < 1 × 10^−5^)^[32]^ and 1851 SNPs for 91 inflammatory cytokines in the inflammatory proteins for AS (*P* < 1 × 10^−5^).^[33]^ In addition, 16 SNPs were extracted as IVs for AS.^[34]^ The detailed characteristics of SNPs associated with gut microbiome, circulating metabolites, immune cells, inflammatory proteins, and AS can be found in Tables S2–S6, Supplemental Digital Content, https://links.lww.com/MD/R607, respectively.

### 3.2. Bidirectional MR analysis of gut microbiota on AS

**Figure 1.**
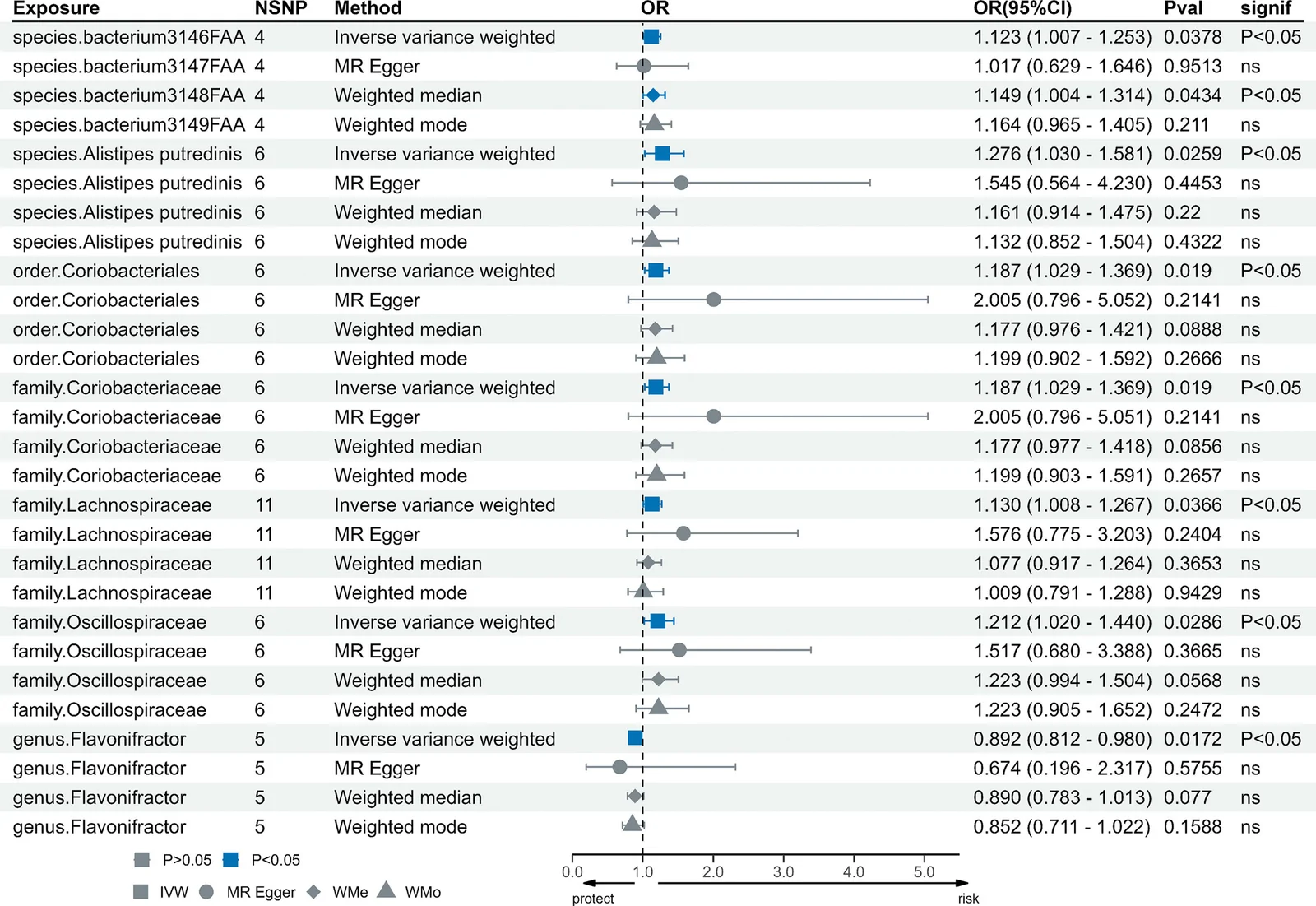
Forest plot of Mendelian randomization analyses. Causal association identified between gut microbiome and aortic stenosis. 95% CI = 95% confidence interval; SNP = single-nucleotide polymorphism.

Gut microbiome taxa’s analysis results are shown in the forest plot (Fig. 1). In brief, a genetically predicted abundance of *bacterium3146FAA* at the species level (OR_IVW_: 1.123, 95% confidence interval [CI]: 1.007–1.253, *P*_IVW_ = 0.038), *Alistipes putredinis* at the species level (OR_IVW_: 1.276, 95% CI: 1.030–1.581, *P* = .026), Coriobacteriaceae at the family level (OR_IVW_: 1.187, 95% CI: 1.029–1.369, *P* = .019), Lachnospiraceae at the family level (OR_IVW_: 1.130, 95% CI: 1.008–1.267, *P* = .008), Oscillospiraceae at the family level (OR_IVW_: 1.212, 95% CI: 1.020–1.440, *P* = .029), and Coriobacteriales at the order level (OR_IVW_: 1.187, 95% CI: 1.029–1.369, *P* = .019) were associated with a higher risk of AS. In contrast, a genetically predicted abundance of *Flavonifractor* at the genus level (OR_IVW_: 0.892, 95% CI: 0.812–0.980, *P* = .017). Reverse MR results showed that AS has no significant causal association with these gut microbiota (Figure S1, Supplemental Digital Content, https://links.lww.com/MD/R607). The outcomes from Cochran *Q*-statistic showed no significant heterogeneity among these selected SNPs (*P* > .05). Finally, MR-Egger regression intercept analyses yielded similar results (*P* > .05), indicating that there was no significant directional level of polytropy (Table S7, Supplemental Digital Content, https://links.lww.com/MD/R607).

### 3.3. Association of blood metabolites and the risk of AS

**Figure 2.**
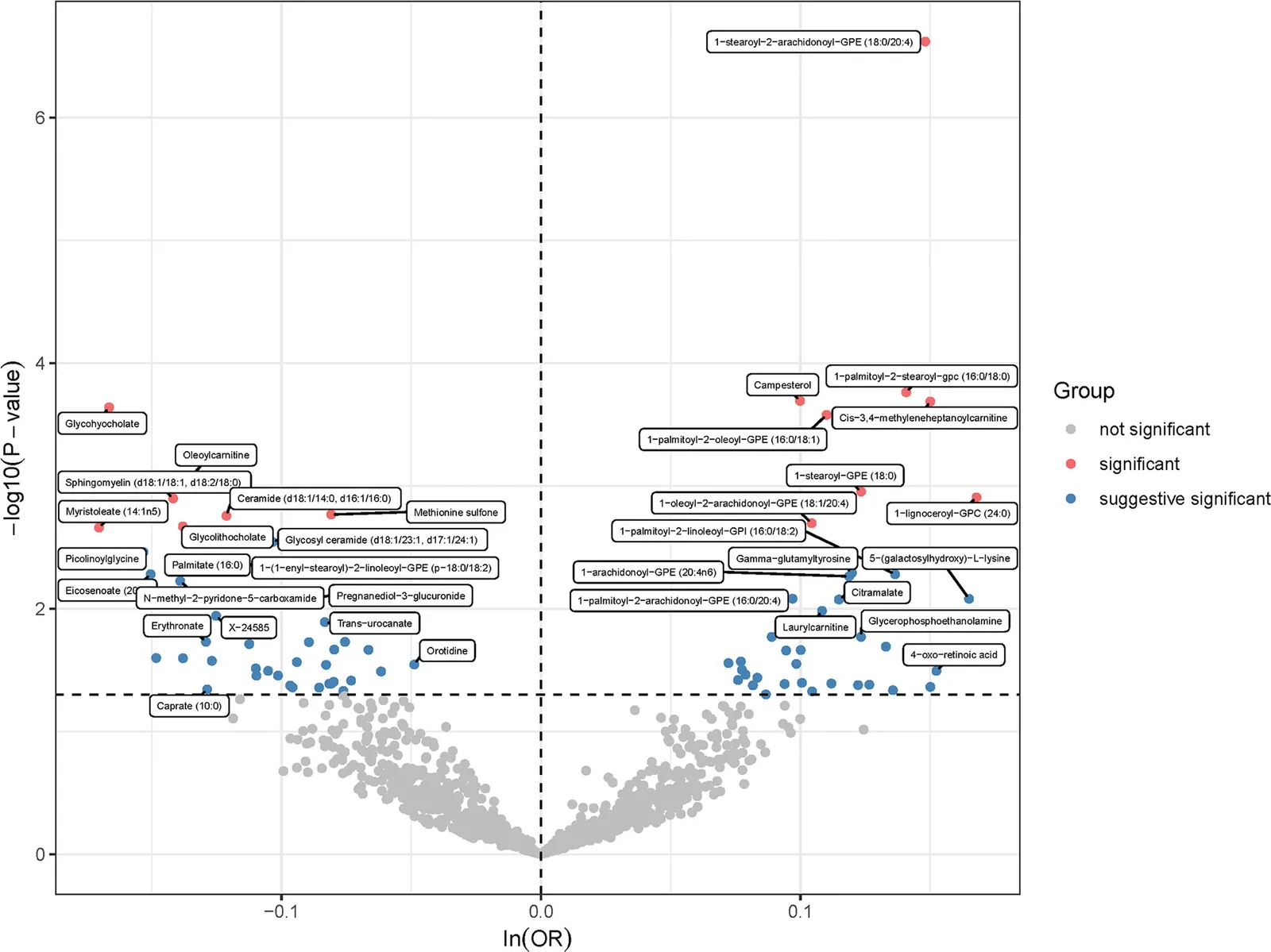
A volcano plot was created to visualize the associations between plasma metabolites and aortic stenosis. The plot displays the odds ratios (OR) on a ln scale, as well as the *P*-values transformed as −log10 (*P*-value) using the inverse variance weighted (IVW) method. The metabolites that showed a causal association with aortic stenosis at a nominal significance level (*P* < .05) were highlighted in pink (*P* < .01, significant) and blue (.01 < *P* < .05, suggestive significant) circles.

**Figure 3.**
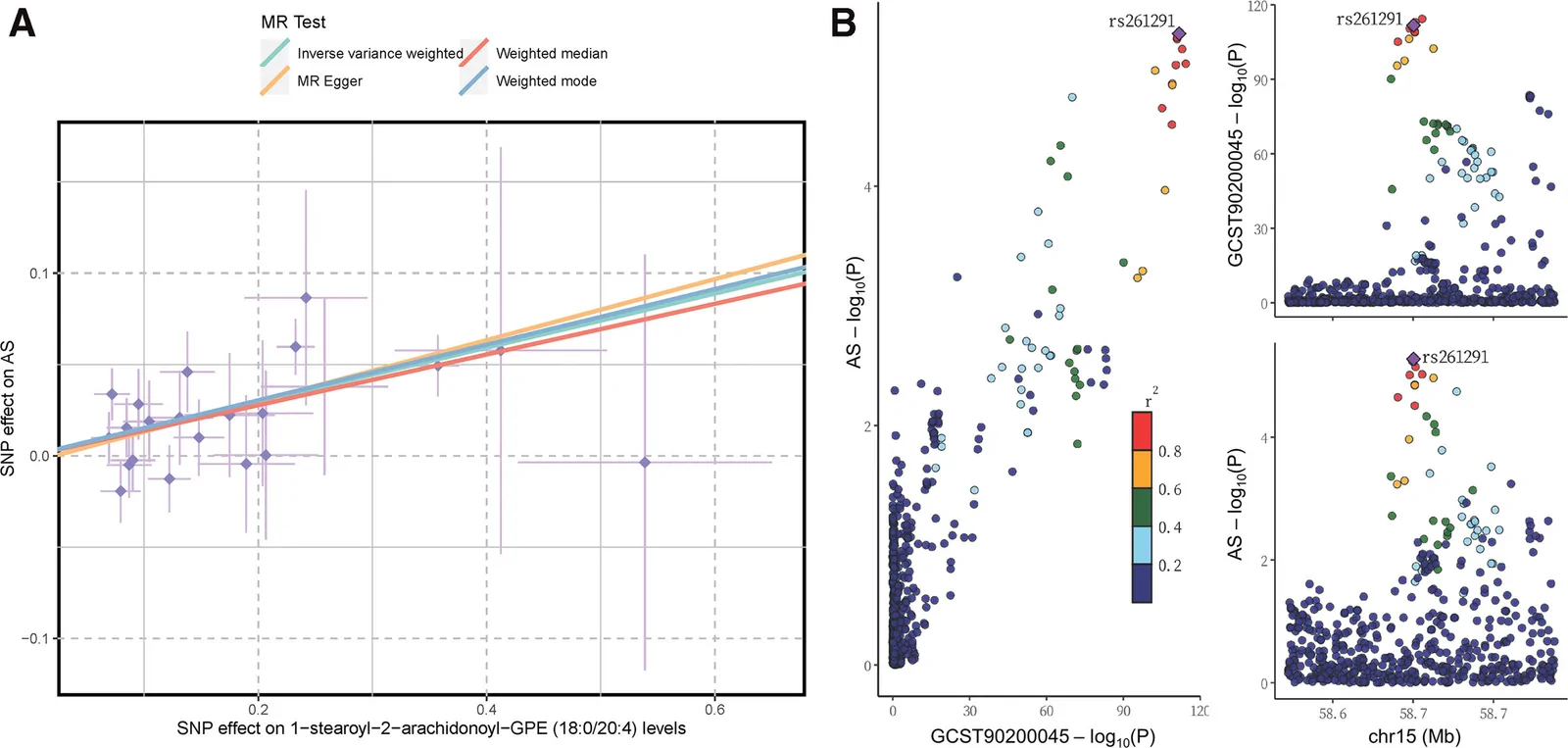
(A) Scatter plots of 1-stearoyl-2-arachidonoyl-GPE (18:0/20:4) levels related to increasing the risk of aortic stenosis. (B) Co-localization analysis of 1-stearoyl-2-arachidonoyl-GPE (18:0/20:4) levels and aortic stenosis (AS). When most points are located on the diagonal, it indicates AS, GWAS, and 1-stearoyl-2-arachidonoyl-GPE (18:0/20:4) levels signals are likely colocalized. Variants are colored by their *r*^2^ value, and the risk variant is labeled and uniquely colored purple. −log10(*P*) association *P*-values for biomarker and −log10(*P*) association *P*-values for expression in AS variants. SNP = single-nucleotide polymorphisms.

We conducted MR analysis on 1091 metabolic, the ratios of 309 metabolites and AS, and discovered 99 suggestive causal associations (*P* < .05), presented the results obtained in the form of a volcano plot (Fig. 2). Among them, there are 38 risk-conferringmetabolites, 42 risk-reducing metabolites, 8 risk-conferring metabolic traits, and 11 risk-reducing metabolite traits. It can be found from the figure that the causal association between the metabolite 1-stearoyl-2-arachidonoyl-GPE (18:0/20:4) levels and AS is very significant. Specifically, a genetically predicted levels of 1-stearoyl-2-arachidonoyl-GPE (18:0/20:4) in blood metabolites (OR_IVW_: 1.160, 95% CI: 1.096–1.227, *P* = 2.40E-07) were associated with a higher risk of AS (Fig. 3A). Afterwards, to ensure the robustness of our results, we further performed inverse MR analysis of this particular metabolite and Bayesian co-localization analysis. The Bayesian co-localization analysis results verified that the causal association of metabolites with AS was significant (Fig. 3B), while the reverse MR results showed that AS had no significant causal association with metabolites (Figure S2, Supplemental Digital Content, https://links.lww.com/MD/R607). MR analysis of metabolites showed without heterogeneity (*P* > .05) and without pleiotropy (*P* > .05). In addition, we performed sensitivity analyzes on the results of 99 significant causal associations (Table S8, Supplemental Digital Content, https://links.lww.com/MD/R607).

### 3.4. Metabolic pathway analysis

In total, we retained 80 causal associations involving 38 metabolites as potential risk factors, and 42 as potential risk reducers. In an attempt to discover metabolic pathways that are associated with AS, we performed metabolic pathway analysis with 80 metabolites. Our results show that the “valine, leucine, and isoleucine biosynthesis” (*P* = .0312) pathways were found to be associated with AS (Figure S3, Supplemental Digital Content, https://links.lww.com/MD/R607).

### 3.5. The causal relationship between immune cells and AS

**Figure 4.**
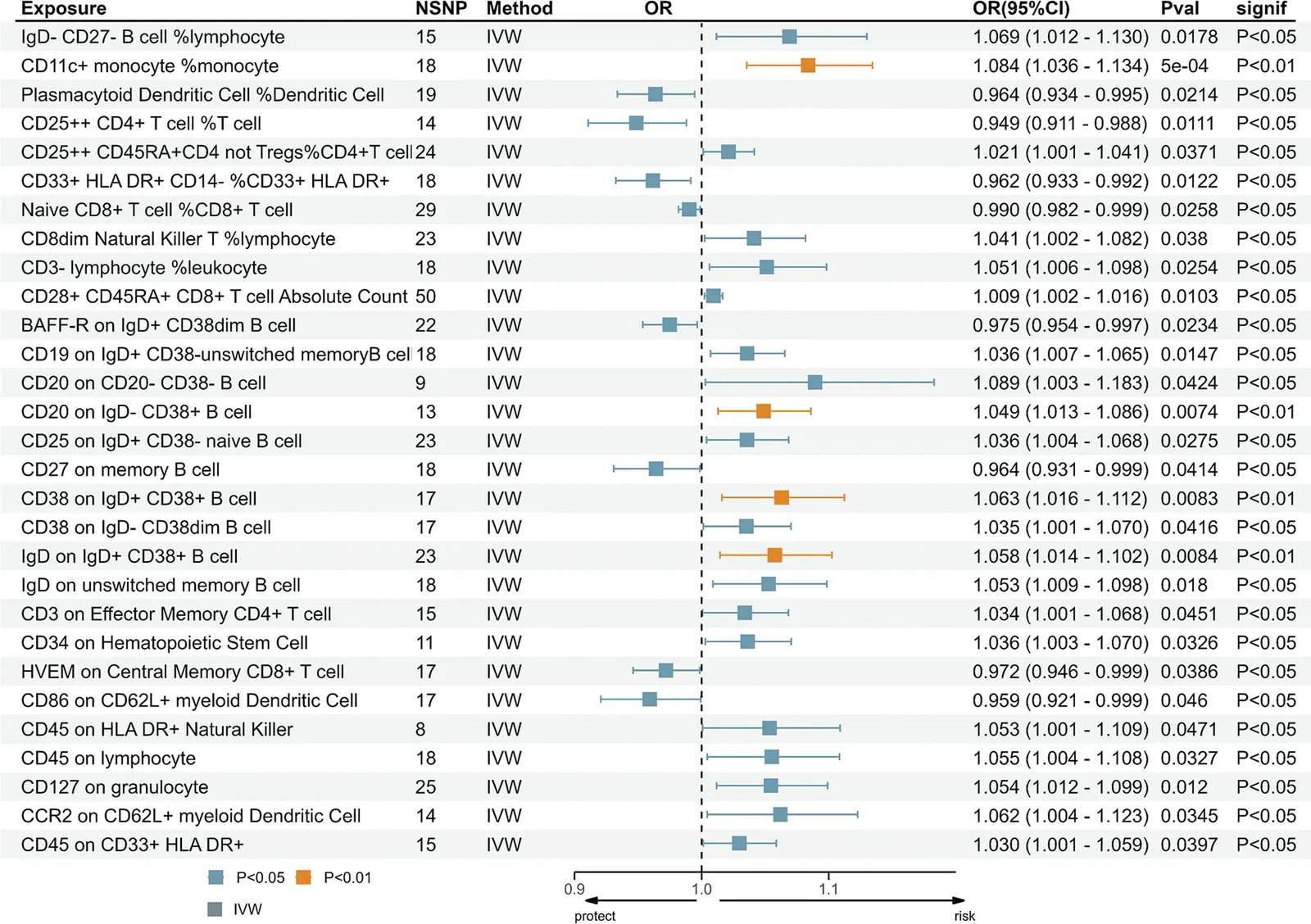
Forest plot of Mendelian randomization analyses. Causal association identified between immune cell phenotypes and aortic stenosis. 95% CI = 95% confidence interval; AS = aortic stenosis; SNP = single-nucleotide polymorphism.

The results of MR analysis showed that 21 types of cells may increase the risk of AS, including IgD− CD27− B cell %lymphocyte (OR: 1.069, 95% CI: 1.012–1.130, *P *= .018), CD11c+ monocyte%monocyte (OR:1.084, 95% CI: 1.036–1.134, *P* = .001), CD25++ CD45RA + CD4 not regulatory T cell %CD4 + T cell (OR: 1.021, 95% CI: 1.001–1.041, *P *= .037), CD8dim Natural Killer T %lymphocyte (OR: 1.041, 95% CI: 1.002–1.082, *P *= .038),CD3- lymphocyte %leukocyte (OR: 1.051, 95% CI: 1.006–1.098, *P* = .025), CD28 + CD45RA + CD8 + T cell absolute count (OR: 1.009, 95% CI: 1.002–1.016, *P* = .010), CD19 on IgD + CD38− unswitched memory B cell (OR: 1.036, 95% CI: 1.007–1.065, *P *= .015),CD20 on CD20− CD38− B cell (OR: 1.089, 95% CI: 1.003–1.183, *P* = .015), CD20 on IgD− CD38 + B cell (OR: 1.049, 95% CI: 1.013–1.186, *P* = .007),CD25 on IgD + CD38− naive B cell (OR: 1.036, 95% CI: 1.004–1.068, *P* = .028), CD38 on IgD + CD38 + B cell (OR: 1.063, 95% CI: 1.016–1.112, *P *= .008), CD38 on IgD-CD38dim B cell (OR: 1.035, 95% CI: 1.001–1.070, *P* = .042), and so on. On the contrary, the results of MR analysis showed that 8 types of cells are negatively correlated with AS, including plasmacytoid dendritic cell %dendritic cell (OR: 0.964, 95% CI: 0.934–0.995, *P* = .021), CD25++ CD4 + T cell %T cell (OR: 0.949, 95% CI: 0.911–0.988, *P* = .011), CD33 + HLA DR + CD14− %CD33 + HLA DR + (OR: 0.962, 95% CI: 0.933–0.992, *P* = .012), naive CD8 + T cell %CD8 + T cell (OR: 0.990, 95% CI: 0.982–0.999, *P* = .026), BAFF-R on IgD + CD38dim B cell (OR: 0.975, 95% CI: 0.954–0.997, *P* = .023), CD27 on memory B cell (OR: 0.964, 95% CI: 0.931–0.999, *P* = .041), HVEM on central memory CD8 + T cell (OR: 0.972, 95% CI: 0.946–0.999, *P* = .039), CD86 on CD62L + myeloid dendritic cell (OR: 0.959, 95% CI: 0.921–0.999, *P *= .046) (Fig. 4).

The most significant result was for the CD11c + monocyte %monocyte, with an odds ratio (OR) of 1.084 and a 95% confidence interval (CI) of 1.036–1.134 (*P* = .00054). Notablily, in addition to this significant result, the levels of CD20 on IgD− CD38 + B cell (*P* = .007), CD38 on IgD + CD38 + B cell (*P*IVW = 0.008) and IgD on IgD + CD38 + B cell (*P* = .008) were also significantly associated with an increased risk of AS. MR sensitivity analyses of weighted median and weighted mode proved the reliability of the results. In reverse MR analysis, we found that the *P*-value of MR-IVW in all inflammatory cells was >.05 (Figure S4, Supplemental Digital Content, https://links.lww.com/MD/R607). MR analysis of immune cells showed without heterogeneity (*P* > .05) and without pleiotropy (*P* > .05). The results of the sensitivity tests were appended to the supplementary document (Table S9, Supplemental Digital Content, https://links.lww.com/MD/R607).

### 3.6. The causal relationship between inflammatory proteins and AS

**Figure 5.**
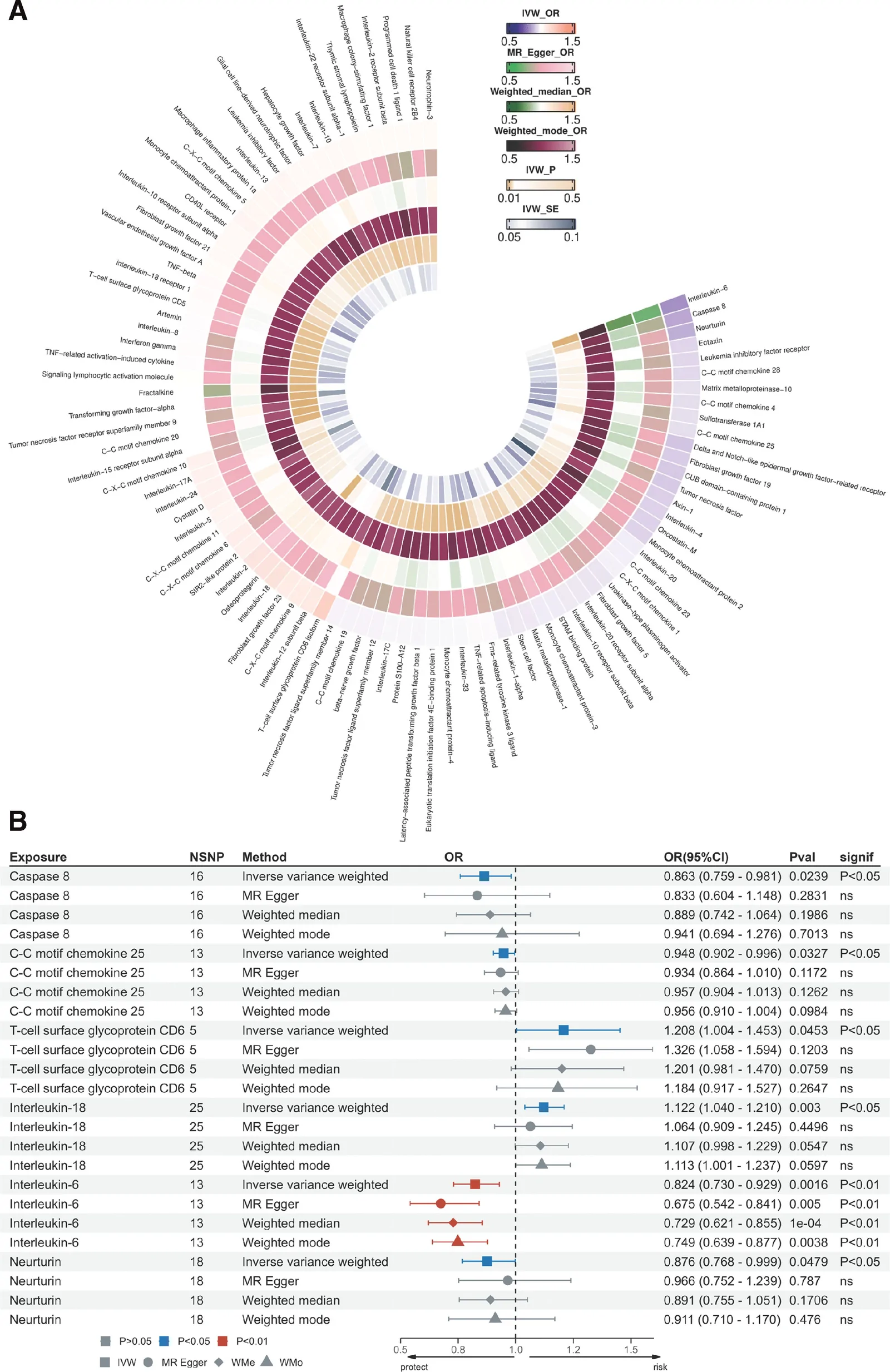
(A) The heatmap plot comprehensively depicts the causal analysis of inflammatory proteins and aortic stenosis. (B) Forest plot of Mendelian randomization analyses. Causal association identified between inflammatory proteins and aortic stenosis. 95% CI = 95% confidence interval; SNP = single-nucleotide polymorphism.

MR results showed that 6 proteins were causally associated with AS, including Caspase 8 levels, C-C motif chemokine 25 levels, T-cell surface glycoprotein CD6 isoform levels, interleukin (IL)-18 levels, interleukin-6 levels, and neurturin levels (Fig. 5A). Specifically, high levels of T-cell surface glycoprotein CD6 isoform levels (OR: 1.208, 95% CI: 1.004–1.453, *P* = .045) and Interleukin-18 levels (ORIVW: 1.122, 95% CI: 1.040–1.210, *P*_IVW_ = 0.003) may increase the risk of AS. On the contrary, high levels of caspase 8 levels (OR: 0.863, 95% CI: 0.759–0.981, *P* = .024), C-C motif chemokine 25 levels (OR: 0.948, 95% CI: 0.902–0.996, *P* = .033), Interleukin-6 levels (OR: 0.824, 95% CI: 0.730–0.929, *P* = .002), and neurturin levels (OR: 0.876, 95% CI: 0.768–0.999, *P* = .048) may reduce the risk of AS (Fig. 5B). It is worth noting that IL-18 levels (*P* = .003) and IL-6 levels (*P* = .002) are the 2 exposures with the most significant causal association with AS (*P* < .01), and the former serves as a risk factor for AS and the latter serves as a protective factor for AS. MR sensitivity analyses of weighted median and weighted mode proved the reliability of the results. Reverse MR results showed that AS has no significant causal association with these inflammatory proteins (Figure S5, Supplemental Digital Content, https://links.lww.com/MD/R607). The MR results of inflammatory proteins have no heterogeneity and pleiotropy (Table S10, Supplemental Digital Content, https://links.lww.com/MD/R607).

### 3.7. The results of secondary analysis

The results of the secondary analysis at *P* < 5 × 10^−8^ as a threshold showed a total of 13 metabolites, 14 immune cells, and 3 inflammatory proteins causally linked to AS. Detailed results are displayed in the Table S11, Supplemental Digital Content, https://links.lww.com/MD/R607.

### 3.8. The causal relationship between the 2 of the identified gut flora, metabolites, immune cell characteristics, and inflammatory proteins

MR results and reverse MR results showed that 3 gut flora were associated with metabolites, 6 metabolites have significant causal association with gut flora; 3 gut flora were associated with immune cell,4 immune cells have significant causal association with gut flora;1 gut flora were associated with inflammatory proteins, inflammatory protein has no significant causal association with gut flora; 7 immune cells were associated with metabolites, 8 metabolites have causal association with immune cells; 4 inflammatory proteins were associated with metabolites and 6 metabolites have causal association with inflammatory proteins. Specifically, *Alistipes* and Lachnospiraceae were revealed to be causally associated with a variety of serum metabolites and immune cell characteristics. Lachnospiraceae was associated with a decrease in 10-heptadecenoate (17:1n7) levels, eicosenoate (20:1) levels, pregnanediol-3-glucuronide levels, oleate/vaccenate (18:1) levels and palmitate (16:0) levels; *Alistipes* were associated with decreased absolute counts of Naive CD8 + T cell %CD8 + T cell and CD28 + CD45RA + CD8 + T cell absolute count; N-acetyltaurine levels were associated with decreases in Lachnospiraceae; CD27 on memory B cell were associated with a decrease in *Alistipes*; sphingomyelin (d18:1/18:1, d18:2/18:0) levels, deoxycholic acid 12-sulfate levels, and trans-4-hydroxyproline levels were associated with increased levels of *Alistipes*; CD38 on IgD- CD38dim B cell and HVEM on central memory CD8 + T cell were associated with an increase in Lachnospiraceae. In addition, we found that erythronate levels were associated with an increase in CD11c + monocyte %monocyte; whereas 4-hydroxychlorothalonillevels, lignoceroyl sphingomyelin (d18:1/24:0) levels, ceramide (d18:1/14:0, d16:1/16:0) levels and caprate (10:0) levels were associated with a decrease in CD11c + monocyte %monocyte. We also identified several metabolites that were causally linked to inflammatory proteins. For example, erythronate levels and 1-(1-enyl-stearoyl)-2-linoleoyl-GPE (*P*-18:0/18:2) levels were associated with increased IL-18, whereas 1-palmitoyl-2-arachidonoyl-GPE (16:0/20:4) levels and 1-linoleoyl-2-arachidonoyl-GPC (18:2/20:4n6) levels were associated with decreased IL-18. In addition, IL-18 was associated with decreased 1-linoleoylglycerol (18:2) levels and eicosenoate (20:1) levels, and increased glycolithocholate levels and deoxycholic acid 12-sulfate levels. Detailed results are displayed in the Table S12, Supplemental Digital Content, https://links.lww.com/MD/R607.

## 4. Discussion

To the best of our knowledge, this is the first MR analysis to explore the causal association between gut microbiome, circulating metabolites, inflammation, and AS. In the present research, we found that 7 gut microbiota, 80 metabolites, 29 immune cells phenotypes, 6 inflammatory proteins, and valine, leucine, and isoleucine biosynthesis are causally associated with AS.

### 4.1. Gut microbiota and AS

We all know that the resident microbial community in the gut converts common nutrients into metabolites, and it has been shown that specific metabolites associated with the microbial community, such as trimethylamine N-oxide, short-chain fatty acids, and secondary bile acids can influence the progression of coronary artery disease.^[45,46]^ In this MR study, we identified potential causal associations between 7 bacterial taxa and AS (*P* < .05 in all 4 MR methods) including *Alistipes putredinis*, Lachnospiraceae and *Flavonifractor*. Previous studies have shown that the genus *Alistipes* has a strong impact on diseases of the cardiovascular system including atrial fibrillation, hypertension, congestive heart failure (HF), and atherosclerosis.^[47–50]^ A metagenomic association study showed that the abundance of genus *Alistipes* was higher in hypertensive patients than in healthy controls. Retrospective and prospective analyzes indicate that hypertension is one of the very important risk factors for AS.^[51]^ Therefore, genus *Alistipes* can be considered a pathogenic bacterium that can increase the risk of AS, which is consistent with our findings. Another MR study showed that genus Lachnospiraceae have a pathogenic role for atherosclerosis.^[52]^ Previous studies have shown that AS and atherosclerosis share the same risk factors, so further exploration of the causal association between family Lachnospiraceae and AS is of great significance to guide future prevention and treatment of AS.^[14]^ Additionally, Prins’ results suggest that species of the genus *Flavonifractor plautii*, can enhance the development of overall cardiovascular disease.^[53]^ In our study, the genus *Flavonifractor* functions as a protective instead of a risk factor for AS. Different arterial sites have different microenvironments and there are also differences in the mechanisms by which the gut microbiota induces cardiovascular disease. The difference in the results from the previous study may also be due to the differences in the methodology and the object of the studies. Accordingly, further exploration of deeper research mechanisms is needed. Furthermore, we also discovered some new gut microbiota, whose effects on AS have never been reported before; for example, *bacteria_3_1_46FAA* and Coriobacteriales have a protective effect on AS, while Oscillospiraceae has a negative effect, and their specific protection mechanisms remain to be further explored. They may serve as new therapeutic targets against AS.

### 4.2. Metabolites and AS

Metabolites are intermediates or end products of metabolic reactions that not only influence the onset and progression of disease but are also targets for therapeutic intervention.^[31]^ Understanding the relationship between genetic variation and metabolites is important to unravel the biological mechanisms of AS. As the incidence of AS continues to rise with the global trend of aging, more and more studies have begun to explore the pathogenesis of AS, and advances in metabolomics have provided valuable insights to this end. For instance, Surendran et al found that lysophosphatidic acid was independently associated with the severity of calcific AS and that lysophosphatidic acid levels were associated with faster progression of calcific AS.^[54]^ In another study, Olkowicz et al used untargeted shotgun proteomics to identify mechanisms and biomarkers of calcific AS. The results highlighted that the most significant differences in urea cycle-related amino acids and BCAA-related amino acids were found in the calcific AS group compared with the control group.^[55]^ Unlike these observational studies, our MR approach revealed innovatively direct causal link between AS risk and 1-stearoyl-2-arachidonoyl-GPE (18:0/20:4) levels, which is validated by Bayesian co-localization analysis and sensitivity analysis. Crucially, through metabolic pathway analysis of the screened positive results, a significant assocaition was found between BCAA and the devlopment of AS, which is consistent with the results of a previous study.^[55]^

To date, no studies have reported the association between 1-stearoyl-2-arachidonoyl-GPE (18:0/20:4) and AS. Notably, the strong causal association between this metabolite and increased risk of AS suggests future risk for various forms of AS and may contribute to its development, rather than simply serving as a novel biomarker. Therefore, our results provide a promising direction for the future search for AS prevention, diagnosis, treatment and drug targets.

This study also found that the metabolism of BCAA has a significant causal relationship with AS. Independent associations supporting a direct role for BCAA in HF,^[56,57]^ vascular disease,^[58,59]^ arrhythmias,^[60]^ and hypertension^[61]^ have emerged. Thus, the link between BCAA and cardiovascular disease has become an area of great interest in both clinical and basic science. The results of this study indicate the significant association between BCAA and AS, providing genetic validation for previous research results.

### 4.3. Immune and inflammatory and AS

AS develops through initial endothelial injury and dysfunction, immune cell infiltration, myofibrillar differentiation of aortic valve mesenchymal cells, and subsequent calcification.^[62]^ A previous study found that valvular changes in AS histology were predominantly inflammatory and partly similar to those in atherosclerotic plaques.^[63]^ Newer studies have also found that immune and inflammatory responses play an important regulatory role in the pathogenesis of AS, including oxidized lipids, various cytokines and biomineralisation.^[64,65]^ The aortic valve is populated by tissue-resident macrophages, mast cells, dendritic cells and T cells.^[66]^ The immune cells in heart valves are predominantly macrophages.^[67]^ Following cardiac injury, monocytes are attracted to CCL2 secreted by mesenchymal cells, leading to an increase in CCR2 + macrophages in the heart.^[68]^ CCR2 + macrophages are associated with inflammation, so they are also called M1 macrophages.^[23]^ According to this study, there is a significant positive correlation between IL-18 levels and an increased risk of AS. In one study, microhaemorrhages were observed in 78% of mineralized AVLs.^[69]^ Microhemorrhage was associated with neovascularisation and macrophage infiltration. Erythrocyte phagocytosis by macrophages promoted IL-18 secretion, thereby exacerbating the mineralization process, which is consistent with our findings.^[70]^ What’s more, we creatively discovered that the level of CD11c + monocyte %monocyte can serve as a factor in AS. A previous study showed that CD11c on blood monocytes is increased during hypercholesterolemia in an ApoE−/− hypercholesterolemic mouse model and that it effects monocyte recruitment and the development of atherosclerosis.^[71]^ Wu et al found that CD11c deficiency reduced solid arrest of mouse monocytes on vascular cell adhesion molecule-1 and E-selectin, decreased monocyte/macrophage aggregation in atherosclerotic lesions, and reduced the development of atherosclerosis in high-fat diet ApoE−/− mice. In another study, Foster et al found that CD11c/CD18 is an inducible integrin whose expression correlates with the inflammatory state of monocytes in patients with myocardial infarction.^[72]^ Despite the knowledge that CD11c + monocytes are associated with certain cardiovascular diseases, the exact relationship between CD11c + monocytes and AS remains unclear. Further exploration of this issue may deepen our understanding of the role of CD11c + monocytes in the development of AS. A previous study has confirmed that CCR9/CCL25 may exert a positive role in MI.^[73]^ However, our study found that CCL25 levels are negatively correlated with increased risk of AS. This may be because although myocardial infarction and aortic calcific stenosis are common types of cardiovascular disease, and they are accompanied by intravascular stenosis and impairment of cardiac function, their pathogenesis is not the same. Thus, changes in CCL25 levels between the 2 diseases are not consistent and may even be in the opposite direction. However, research on the relationship between the levels of CCL25 and health remains limited, and the causal effect of this inflammatory protein on AS warranted further investigation. In addition, we also discovered some new immune cells and inflammatory proteins that have an effect on AS; for example, CD20 on IgD- CD38 + B cell, CD38 on IgD + CD38 + B cell and IgD on IgD + CD38 + B cell and some inflammatory proteins T-cell surface glycoprotein CD6 isoform have a risk effect on AS, while neurturin and caspase 8 has a negative effect, and their specific protective mechanisms remain to be explored further. In addition to inflammatory factors, cardiovascular biomarkers have been shown to be associated with cardiovascular disease. Specifically, it has been noticed that the increase of aortic gradient and LV muscle overload leads to the release of troponin T and N-terminal-pro-B-type natriuretic peptide (NT-proBNP) from cardiomyocytes.^[74]^ An increase in the above-mentioned biomarkers has the ability to predict poor prognosis. Furthermore, there is also a connection between gut microbiota and cardiovascular biomarkers such as high-sensitivity troponin T, B-type natriuretic peptide (BNP) and NT-proBNP. For example, in patients with HF, Parabacteroides and Bacteroides were negatively correlated with BNP, *Klebsiella* was positively correlated with BNP, *Escherichia*–*Shigella* was positively correlated with BNP, and *Alistipes* was negatively correlated with BNP^[75]^; and in patients with prior myocardial infarction, trimethylamine N-oxide, a circulating metabolite produced by gut microbiota was weakly positively correlated with hs-TNT and NT-proBNP.^[76]^ They may serve as new therapeutic targets against AS.

### 4.4. Phenotypic alterations and genetic variation in the aortic valve microenvironment

The relationship between leaflet morphology, fibrotic ECM accumulation, pathological VIC differentiation, and genetic variation in the complex aortic valve microenvironment is multifaceted and interconnected through mechanical, cellular, and molecular pathways influenced by genetic factors. The aortic valve leaflets have a trilaminar ECM structure composed mainly of collagen (fibrosa), proteoglycans and glycosaminoglycans (spongiosa), and elastin (ventricularis). This structure is critical to withstand mechanical forces during the cardiac cycle. In disease states, this ECM architecture is disrupted: collagen fibers become fragmented and accumulate preferentially in the fibrosa layer, contributing to leaflet thickening and stiffening. Proteoglycans and glycosaminoglycans increase significantly, about fourfold, in diseased valves, further altering leaflet morphology and mechanical properties. These ECM changes lead to fibrotic remodeling of the valve leaflets, which manifests as thickened, less compliant leaflets with altered morphology.^[77]^ VICs are the main resident cells responsible for maintaining valve ECM homeostasis. Under pathological stimuli, VICs differentiate into activated myofibroblast-like or osteoblast-like phenotypes. Myofibroblast differentiation promotes fibrotic ECM accumulation through increased collagen synthesis and matrix remodeling enzymes, while osteoblast-like differentiation contributes to calcification. This pathological differentiation is driven by altered mechanical cues (e.g., increased stiffness), biochemical signals (e.g., cytokines, growth factors), and ECM composition changes.^[77,78]^ VIC differentiation and ECM remodeling form a feedback loop that exacerbates leaflet fibrosis and morphological abnormalities. Genetic mutations affect key regulatory pathways that govern valve development, VIC function, and ECM composition. Mutations affect epithelial-to-mesenchymal transition, calcium deposition, and circulating endothelial progenitor cells, influencing valve calcification and fibrosis. These transcription factors (e.g., GATA4, GATA5, GATA6) regulate valve formation and ECM remodeling; mutations can impair endothelial-to-mesenchymal transition and dysregulate matrix metalloproteinases, leading to ECM disorganization and pathological VIC differentiation.^[79]^ Genes involved in the TGF-β signaling pathway (e.g., TGFBR2) and smooth muscle contractile proteins (e.g., ACTA2, MYH11) influence ECM integrity and VIC/smooth muscle cell phenotypes, affecting valve and aortic wall structure. Genetic predisposition can lead to intrinsic ECM abnormalities, making the valve leaflets more susceptible to pathological remodeling and VIC phenotype shifts even before mechanical or hemodynamic stress is applied. In bicuspid aortic valve (BAV) disease, genetic mutations cause abnormal leaflet morphology (2 leaflets instead of 3), which alters valve-related hemodynamics. Altered flow patterns increase regional wall shear stress, promoting localized ECM dysregulation and elastic fiber degeneration in the valve and ascending aorta. This hemodynamic stress interacts with genetic susceptibility to accelerate pathological remodeling. The interplay between genetic factors and altered mechanical environment drives VIC pathological differentiation and ECM fibrosis, further modifying leaflet morphology and valve function.

### 4.5. Bicuspid aortic valve and calcific aortic valve disease (CAVD)

The BAV, the most common congenital cardiac anomaly, significantly increases the risk of CAVD and accelerates valvular dysfunction due to altered hemodynamics and mechanical stress. BAV is highly heritable, with familial clustering observed in 9–20% of cases, yet its inheritance does not follow a simple Mendelian pattern. Genetic studies have implicated mutations in NOTCH1 and other loci (e.g., GATA5, SMAD6), but a polygenic model with environmental modifiers is likely. These genetic alterations disrupt normal valve morphogenesis, leading to the characteristic bicuspid phenotype.^[80]^ Additionally, BAV is frequently associated with congenital abnormalities, such as coarctation of the aorta and aortic root dilation, suggesting shared developmental pathways, potentially involving neural crest cell migration defects.^[81]^ The accelerated progression to CAVD in BAV patients is driven by increased shear stress and upregulation of pro-calcific pathways, including TGF-β and BMP signaling, which promote VIC differentiation and matrix remodeling.^[81]^

### 4.6. Strengths and limitations

Our work has several important advantages. Using MR as a research methodology can largely reduce the effects of confounding factors and mitigate reverse causality. What’s more, the use of multiple methods for multidirectional and sensitivity analyses makes the results more reliable. By incorporating the various exposures into the MR analyses, we were able to limit bias due to potential common genetic effects between phenotypes. In addition, the use of a dataset largely restricted to European ancestry also limited bias due to population stratification. First, this study relied heavily on self-reported phenotypes, which may be subject to reporting bias. Second, we screened for IVs using a *P*-value of *P* < 1 × 10^−5^, so the IVs were not strongly enough correlated, although they do allow for a more comprehensive assessment of the association between multi-omics and AS. Third, similar to previous studies, this study did not correct the results with relevant methods,^[82–85]^ but MR analysis of individual exposures with AS showed no false-positive results. In addition, we finally corrected the results for FDR on the basis of all MR studies and present them in the supplementary file to provide a guide for future research directions. Fourth, further sex- or age-stratified analyses were not possible using pooled-level data. Fifth, we were unable to completely rule out shared genetic influences between polyphenotypes and AS, which could potentially bias MR estimates. Sixth, a significant correlate found in this study is inconsistent with previous research findings. CCR2 + macrophages also secrete IL-6 to promote mineralization. which will aggravate the progression of AS.^[86]^ However, our study results found that IL-6, as a protective factor for AS, was significantly associated with AS. Although different from previous findings, this provides a new insight at the genetic level. Seventh, in this study, only IVW had a statistically significant *P*-value in MR analysis, and the other 3 MR methods had no statistical difference, which may weaken the reliability of the research conclusions to a certain extent, but we only selected IVW as the main method and the other 3 MR methods as supplements, and required that the OR directions of the 4 methods were the same. This minimized the impact of this force majeure factor on the research results. Eighth, for gut microbiota and metabolites, further omics-related studies, such as 16s microbial sequencing and metabolomics sequencing, may be needed in the future to clarify the changes in the gut microbiome in AS and to validate them with the changes in metabolites in serum and tissues. In addition, some biomolecular experiments may be needed for validation, such as fecal transplants to clarify the pathogenicity of gut microbes, and administration of metabolites to determine the role of these metabolites in the disease. For immune cells, techniques such as single-cell sequencing and flow cytometry can be used to further confirm these AS markers. For inflammatory proteins they can be further identified using enzyme-linked immunosorbent assays and protein immunoblotting. Alternatively, AS model mice can be treated with these substances to explore their effects on the disease.

Overall, this study draws a blueprint for the genetic correlation between multi-omics and AS at the genetic level, providing scientific basis and unique insights for basic research and clinical research related to AS.

## 5. Conclusions

This Mendelian randomization study supports a causal effect of gut microbiota, blood metabolites, immune cells, and inflammatory proteins on AS. Seven gut microbiota, 80 blood metabolites, 29 immune cells, and 6 circulating inflammatory proteins were detected that may increase or decrease the risk of developing AS. Co-localization analysis showed a significant correlation between 1-stearoyl-2-acryloyl-GPE levels and AS. These may hold promise for the prevention of AS. In addition, the clinical benefits of gut microbiota, blood metabolites, immune cells, and inflammatory proteins should be further evaluated in future large population-based studies.

## Acknowledgments

We would like to sincerely thank the original GWASs and the related consortiums for sharing and managing the summary statistics.

## Author contributions

**Conceptualization:** Guolin Zhang.

**Data curation:** Guolin Zhang.

**Investigation:** Yangyou Zhang.

**Methodology:** Yangyou Zhang.

**Software:** Yangyou Zhang.

**Visualization:** Yangyou Zhang.

**Writing – review & editing:** Yangyou Zhang, Guolin Zhang.

## Supplementary Material


