# Supplementary material for: Causal relationship between gut microbiome, plasma metabolites, inflammation, and aortic stenosis: A multi-omics Mendelian randomization analysis
Source: Medicine (Baltimore). 2026 Apr 10;105(15):e48238. doi: 10.1097/MD.0000000000048238 (PMC13593140; doi:10.1097/MD.0000000000048238)
Supplement: Supplementary file 1 [file medi-105-e48238-s001.pdf]

Graphical Abstract:

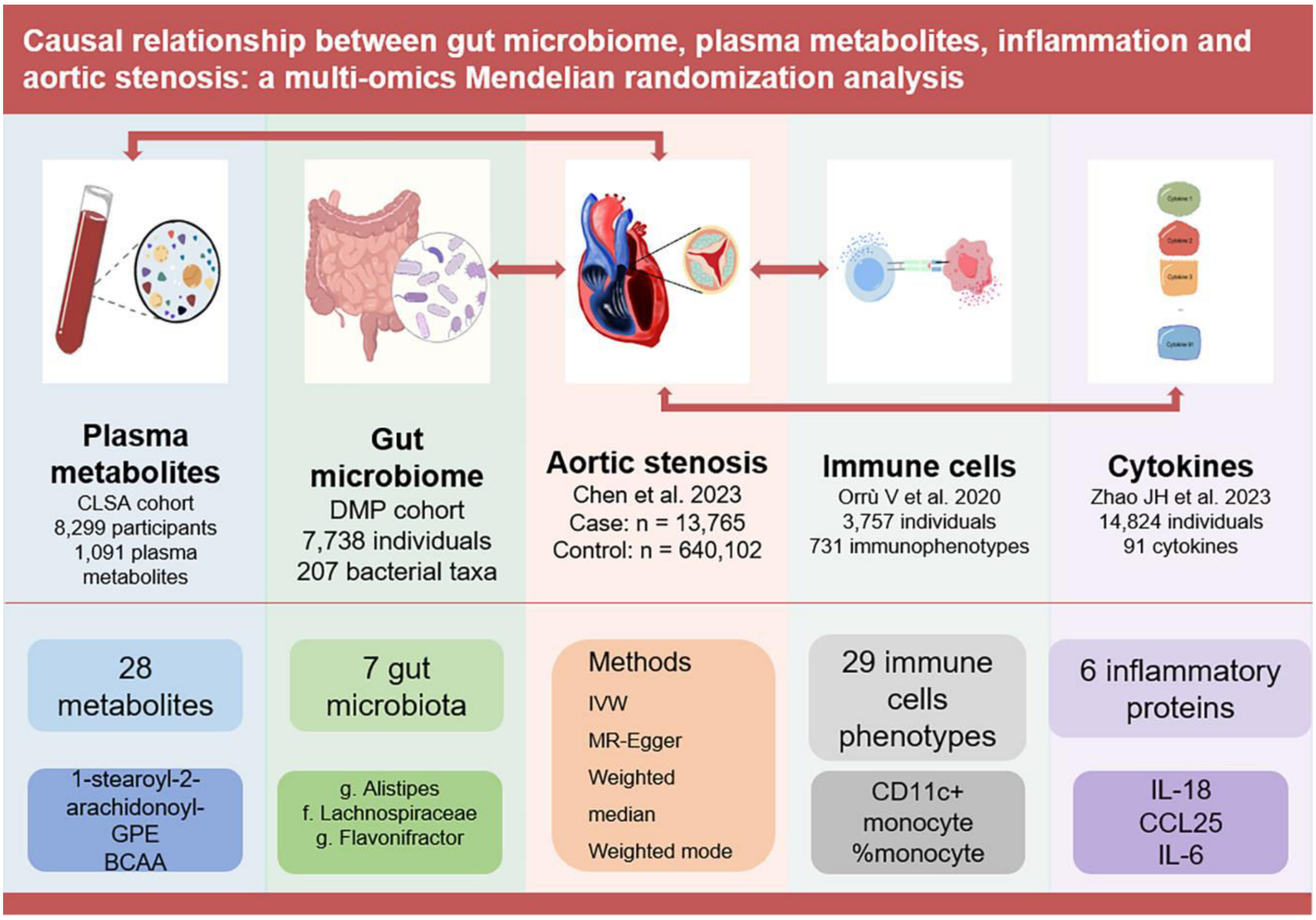

Figure S1. Forest plot of reverse Mendelian randomization analyses. Causal association identified between gut microbiome and aortic stenosis. SNP, single-nucleotide polymorphism; 95%CI, 95% confidence interval.

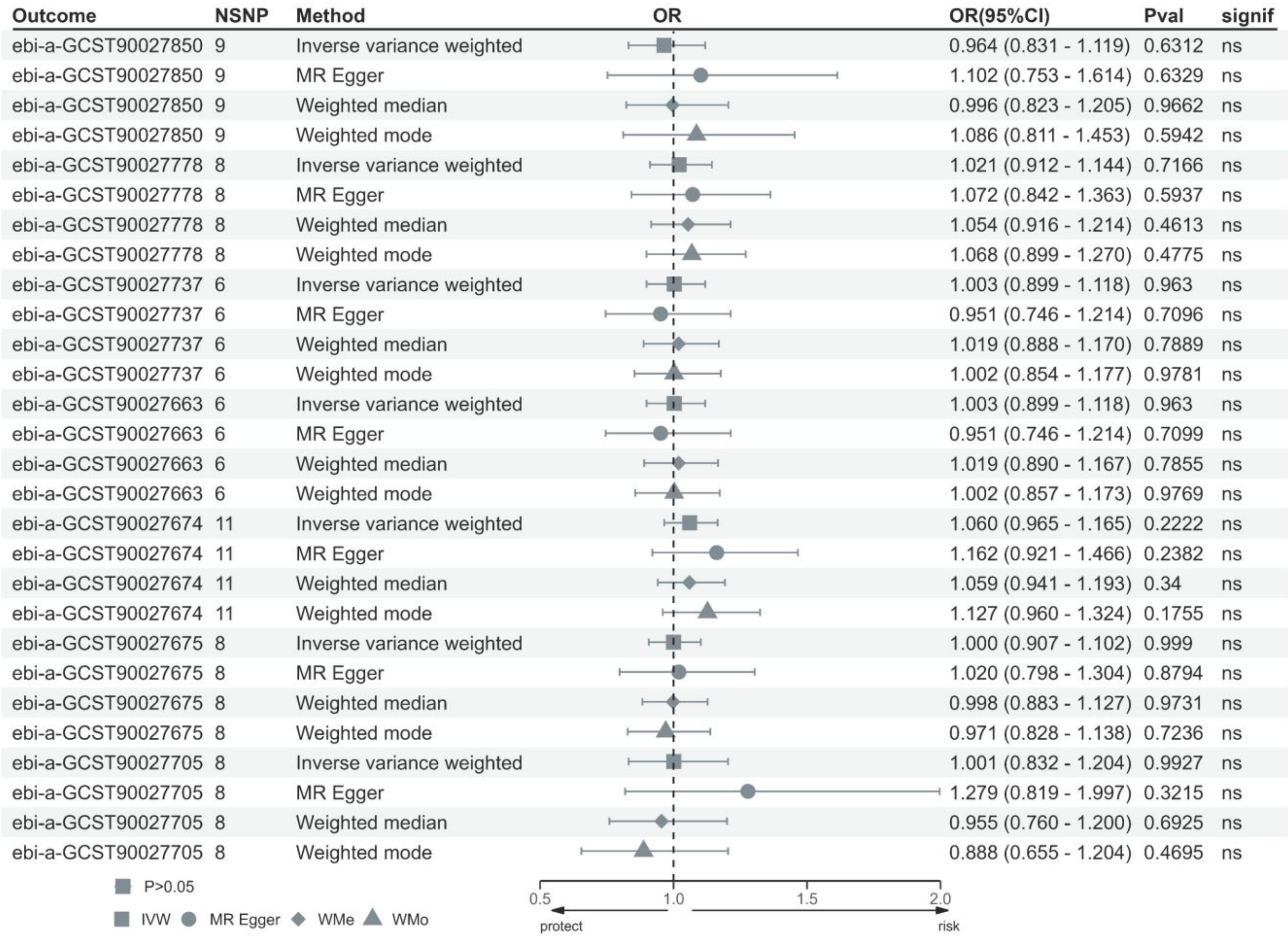

Figure S2. Scatter plots of reverse Mendelian randomization analyses. Causal association identified between metabolites and aortic stenosis.

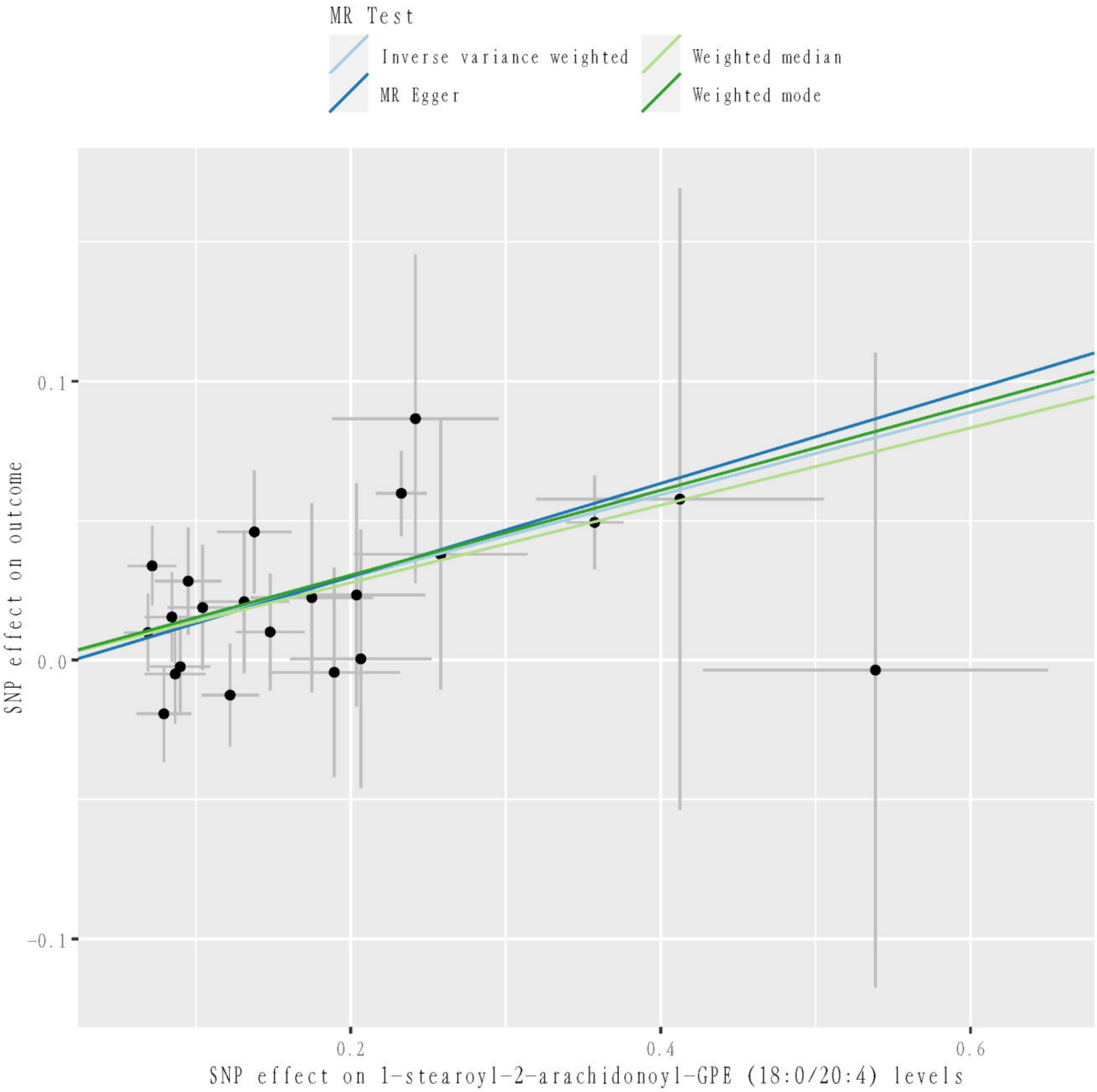

Figure S3. Metabolic pathways analysis with all identified aortic stenosis-related metabolites based on KEGG databases.

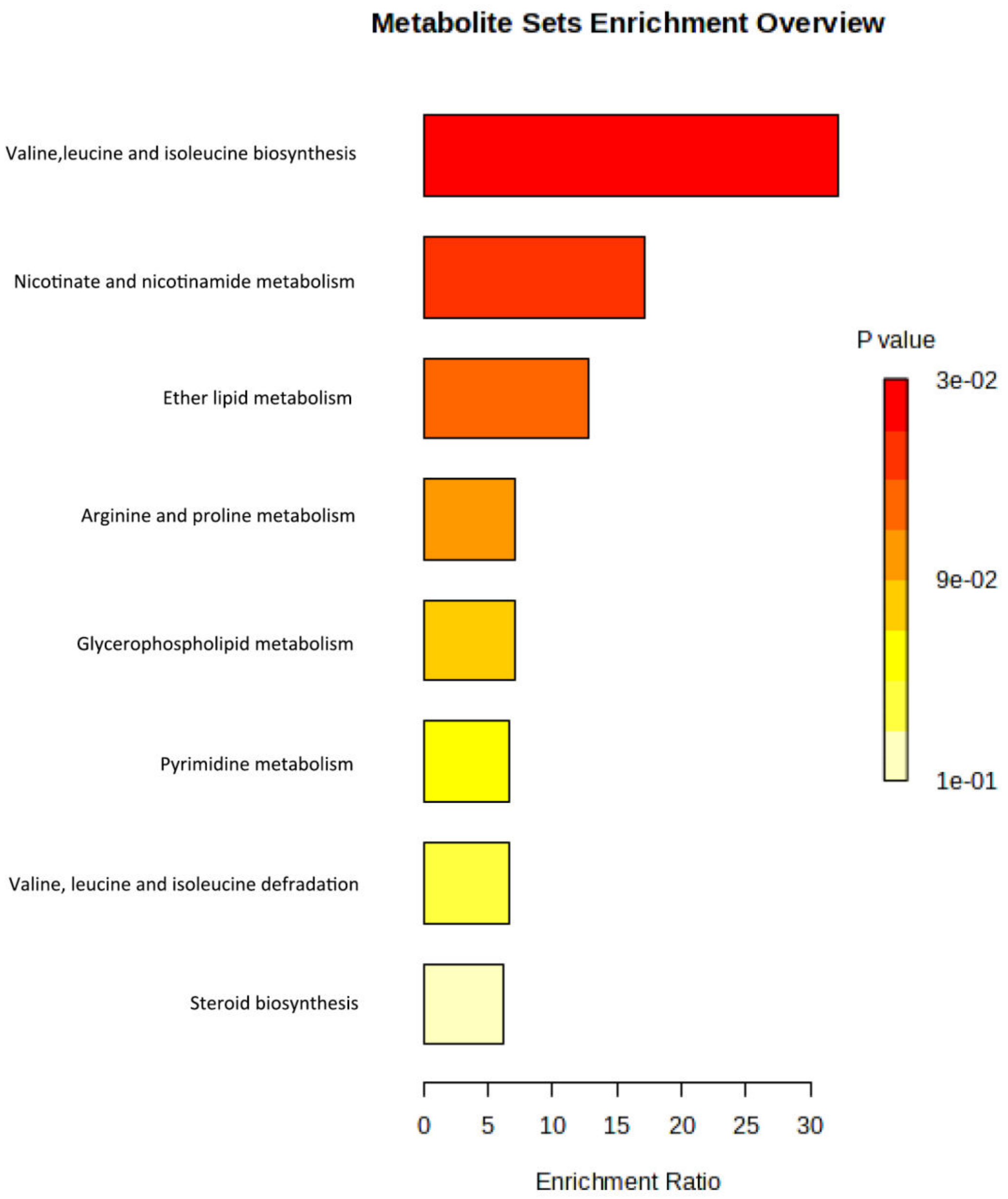

Figure S4. Forest plot of reverse Mendelian randomization analyses. Causal association identified between inflammatory cells and aortic stenosis. SNP, single-nucleotide polymorphism; 95%CI, 95% confidence interval.

ID,Outcome	NSNP	Method	OR(95%CI)	Pval	signif
ebi−a−GCST90001433	16	Inverse variance weighted	1.001 (0.884 − 1.134)	0.9817	ns
ebi−a−GCST90001449	9	Inverse variance weighted	0.991 (0.830 − 1.183)	0.9184	ns
ebi−a−GCST90001474	16	Inverse variance weighted	0.961 (0.848 − 1.088)	0.5287	ns
ebi−a−GCST90001506	16	Inverse variance weighted	0.987 (0.870 − 1.119)	0.8331	ns
ebi−a−GCST90001508	16	Inverse variance weighted	1.002 (0.883 − 1.137)	0.9795	ns
ebi−a−GCST90001519	16	Inverse variance weighted	1.007 (0.852 − 1.190)	0.9344	ns
ebi−a−GCST90001552	7	Inverse variance weighted	0.948 (0.837 − 1.073)	0.3952	ns
ebi−a−GCST90001635	11	Inverse variance weighted	1.055 (0.918 − 1.212)	0.4509	ns
ebi−a−GCST90001641	9	Inverse variance weighted	0.966 (0.824 − 1.131)	0.6652	ns
ebi−a−GCST90001690	5	Inverse variance weighted	1.035 (0.862 − 1.243)	0.7132	ns
ebi−a−GCST90001709	6	Inverse variance weighted	0.973 (0.811 − 1.168)	0.7721	ns
ebi−a−GCST90001728	5	Inverse variance weighted	1.058 (0.776 − 1.442)	0.7219	ns
ebi−a−GCST90001744	7	Inverse variance weighted	1.042 (0.890 − 1.222)	0.6072	ns
ebi−a−GCST90001756	16	Inverse variance weighted	1.061 (0.911 − 1.237)	0.446	ns
ebi−a−GCST90001781	16	Inverse variance weighted	0.984 (0.832 − 1.164)	0.8531	ns
ebi−a−GCST90001805	5	Inverse variance weighted	1.006 (0.829 − 1.221)	0.9516	ns
ebi−a−GCST90001812	6	Inverse variance weighted	0.960 (0.811 − 1.137)	0.636	ns
ebi−a−GCST90001815	6	Inverse variance weighted	0.939 (0.795 − 1.109)	0.4577	ns
ebi−a−GCST90001824	4	Inverse variance weighted	1.073 (0.867 − 1.327)	0.5168	ns
ebi−a−GCST90001826	5	Inverse variance weighted	1.082 (0.895 − 1.308)	0.4138	ns
ebi−a−GCST90001843	16	Inverse variance weighted	1.047 (0.898 − 1.220)	0.5597	ns
ebi−a−GCST90001870	7	Inverse variance weighted	0.954 (0.733 − 1.240)	0.7222	ns
ebi−a−GCST90001882	16	Inverse variance weighted	1.030 (0.842 − 1.260)	0.7733	ns
ebi−a−GCST90001904	5	Inverse variance weighted	0.963 (0.781 − 1.187)	0.7241	ns
ebi−a−GCST90001912	4	Inverse variance weighted	0.939 (0.752 − 1.172)	0.5773	ns
ebi−a−GCST90001914	11	Inverse variance weighted	0.999 (0.856 − 1.165)	0.9878	ns
ebi−a−GCST90001926	5	Inverse variance weighted	1.017 (0.839 − 1.233)	0.8648	ns
ebi−a−GCST90002014	3	Inverse variance weighted	1.139 (0.889 − 1.459)	0.3033	ns
ebi−a−GCST90002053	4	Inverse variance weighted	1.041 (0.758 − 1.430)	0.8044	ns

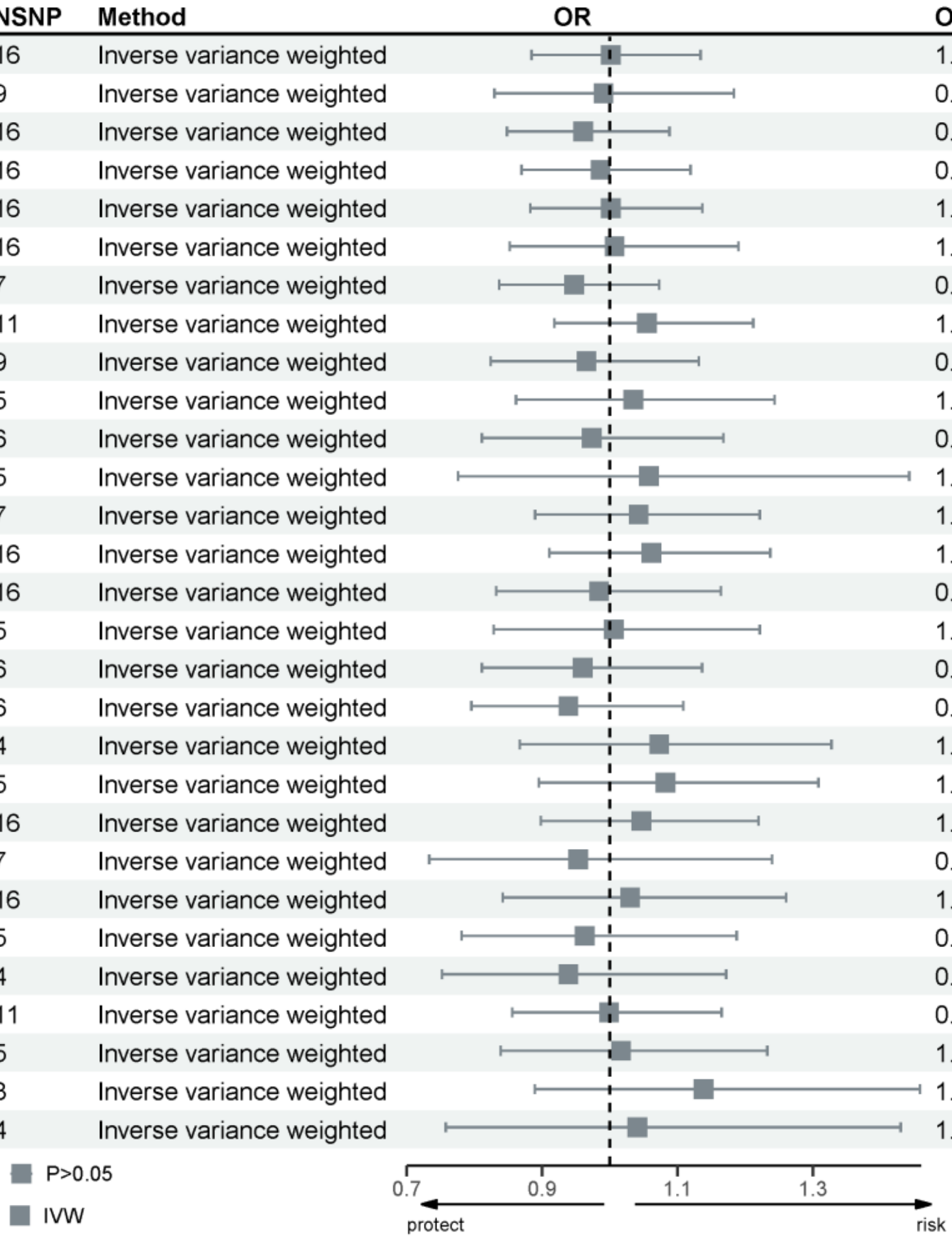

Figure S5. Forest plot of reverse Mendelian randomization analyses. Causal association identified between inflammatory proteins and aortic stenosis. SNP, single-nucleotide polymorphism; 95%CI, 95% confidence interval.

Outcome	NSNP	Method	OR(95%CI)	Pval	signif
Caspase 8	13	Inverse variance weighted	0.981 (0.920 - 1.046)	0.5571	ns
Caspase 8	13	MR Egger	0.934 (0.801 - 1.088)	0.4003	ns
Caspase 8 levels	13	Weighted median	0.984 (0.903 - 1.072)	0.7114	ns
Caspase 8 levels	13	Weighted mode	0.926 (0.818 - 1.048)	0.2475	ns
C-C motif chemokine 25	8	Inverse variance weighted	0.992 (0.915 - 1.077)	0.8547	ns
C-C motif chemokine 25	8	MR Egger	0.877 (0.695 - 1.108)	0.3143	ns
C-C motif chemokine 25	8	Weighted median	0.975 (0.878 - 1.082)	0.6283	ns
C-C motif chemokine 25	8	Weighted mode	0.952 (0.820 - 1.105)	0.5365	ns
T-cell surface glycoprotein CD6	8	Inverse variance weighted	0.979 (0.909 - 1.054)	0.5725	ns
T-cell surface glycoprotein CD6	8	MR Egger	0.948 (0.815 - 1.103)	0.5128	ns
T-cell surface glycoprotein CD6	8	Weighted median	0.967 (0.881 - 1.062)	0.4861	ns
T-cell surface glycoprotein CD6	8	Weighted mode	0.955 (0.852 - 1.070)	0.4529	ns
Interleukin-18	9	Inverse variance weighted	0.933 (0.864 - 1.007)	0.0739	ns
Interleukin-18	9	MR Egger	0.892 (0.766 - 1.038)	0.1828	ns
Interleukin-18	9	Weighted median	0.914 (0.831 - 1.006)	0.0666	ns
Interleukin-18	9	Weighted mode	0.902 (0.800 - 1.018)	0.1349	ns
Interleukin-6	9	Inverse variance weighted	0.963 (0.897 - 1.035)	0.3039	ns
Interleukin-6	9	MR Egger	0.941 (0.786 - 1.127)	0.5304	ns
Interleukin-6	9	Weighted median	0.943 (0.858 - 1.036)	0.2214	ns
Interleukin-6	9	Weighted mode	0.928 (0.829 - 1.040)	0.2339	ns
Neurturin	9	Inverse variance weighted	0.939 (0.868 - 1.015)	0.1129	ns
Neurturin	9	MR Egger	0.923 (0.770 - 1.107)	0.4172	ns
Neurturin	9	Weighted median	0.911 (0.828 - 1.003)	0.0577	ns
Neurturin	9	Weighted mode	0.902 (0.795 - 1.023)	0.1458	ns

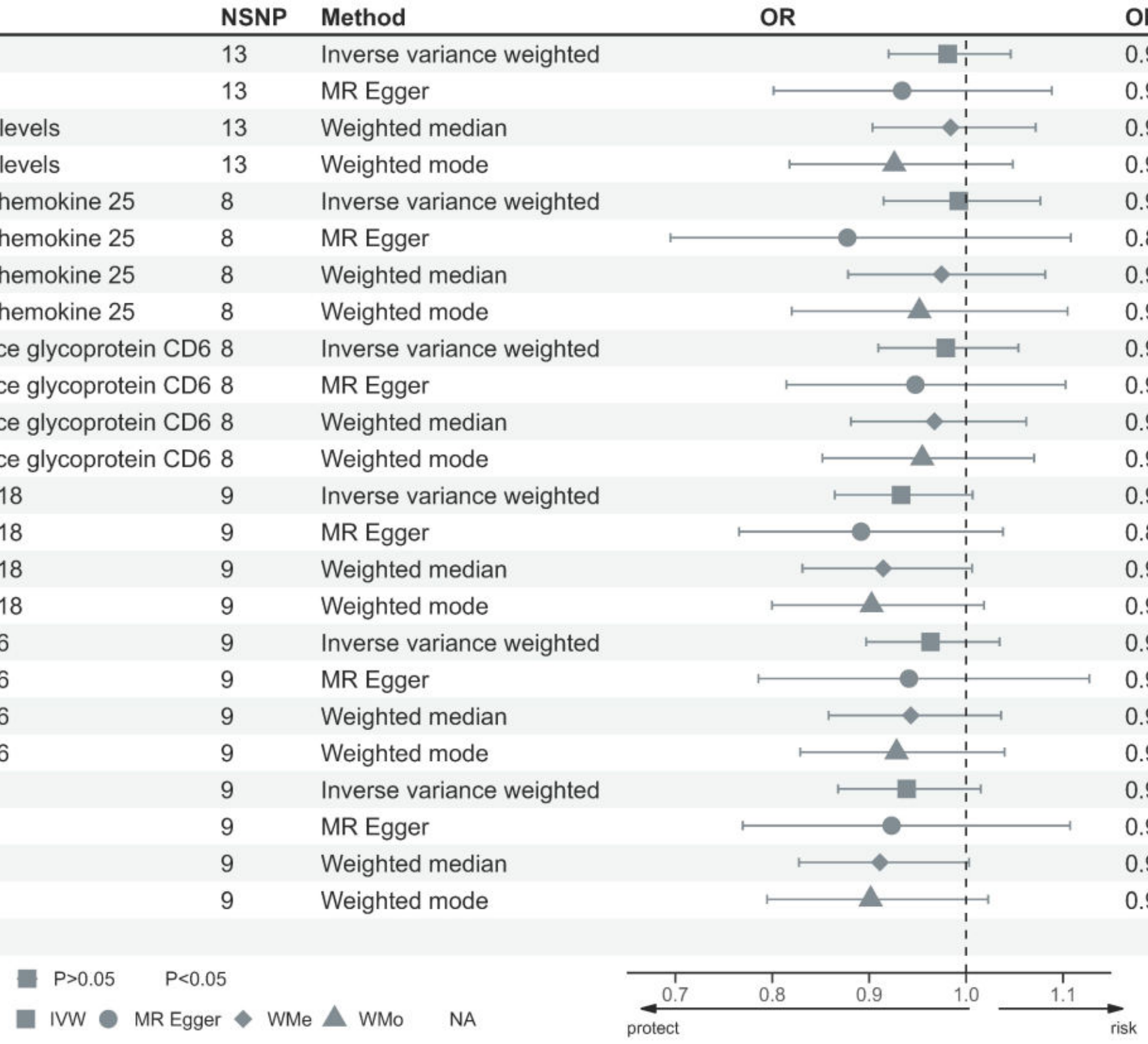

Table S1 Detailed information for genome-wide association studies involved in the present Mendelian Randomization study.

Phenotype	Consortium or study	Sample size	Ancestry	Journal	Year	Author	DOI
gut microbiota	Dutch Microbiome Project	7,738	European	Nature Genetics	2022	Lopera-Maya et al.	10.1038/s41588-021-00992-y
circulating metabolites	Canadian Longitudinal Study on Aging cohort	8,299	European	Nat Genet	2023	Chen et al.	10.1038/s41588-022-01270-1
immune cells	The study encompassed 731 immunophenotypes, including 118 absolute cell counts (AC), 389 median fluorescence intensities (MFI), 32 morphological parameters (MP), and 192 relative cell counts (RC).	3,757	European	Nature Genetics	2020	Orrù et al.	10.1038/s41588-020-0684-4
inflammation-related proteins	91 inflammatory cytokines	14,824	European	Nat Immunol	2023	Zhao et al.	10.1038/s41590-023-01588-w
coronary artery calcification	Summary statistics were obtained from a meta-analysis of 26,909 individuals of European ancestry and 8,867 individuals of African ancestry	26,909 individuals of European ancestry and 8,867 individuals of African ancestry	European and African	Meta-Analysis	2023	Maryam Kavousi et al.	10.1038/s41588-023-01518-4

Table S2 Mendelian Randomization analysis on the causal effect between gut microbiota and coronary artery calcification.

id.exposure	exposure	id.outcome	method	nsnp	b	se	pval	lo_ci	up_ci	or	or_lci95	or_uci95	pleiotropy	heterogeneity	p-FDR
GCST90027809	Gut microbiota abundance (k_Bacteria.p_Proteobacteria.c_Betaproteobacteria.o_Burkholderiales.f_Burkholderiales_noname.g_Burkholderiales_noname.s_Burkholderiales_bacterium_1_1_47)	CAC	Inverse variance weighted	14	-0.1118	0.054724	0.041057	-0.21906	-0.00454	0.894224	0.803275	0.995471	0.352718761	0.179566965	0.733478256
GCST90027809	Gut microbiota abundance (k_Bacteria.p_Proteobacteria.c_Betaproteobacteria.o_Burkholderiales.f_Burkholderiales_noname.g_Burkholderiales_noname.s_Burkholderiales_bacterium_1_1_47)	CAC	MR Egger	14	-0.38286	0.285681	0.205017	-0.94279	0.177077	0.681909	0.389538	1.193723		0.182758916	
GCST90027809	Gut microbiota abundance (k_Bacteria.p_Proteobacteria.c_Betaproteobacteria.o_Burkholderiales.f_Burkholderiales_noname.g_Burkholderiales_noname.s_Burkholderiales_bacterium_1_1_47)	CAC	Weighted median	14	-0.14944	0.068583	0.029336	-0.28386	-0.01502	0.861192	0.752872	0.985097			
GCST90027809	Gut microbiota abundance (k_Bacteria.p_Proteobacteria.c_Betaproteobacteria.o_Burkholderiales.f_Burkholderiales_noname.g_Burkholderiales_noname.s_Burkholderiales_bacterium_1_1_47)	CAC	Weighted mode	14	-0.1958	0.125061	0.141443	-0.44092	0.04932	0.822177	0.643445	1.050556			
GCST90027809	Gut microbiota abundance (k_Bacteria.p_Proteobacteria.c_Betaproteobacteria.o_Burkholderiales.f_Burkholderiales_noname.g_Burkholderiales_noname.s_Burkholderiales_bacterium_1_1_47)	CAC	Contamination mixture	14	-0.22287	0.081633	0.008964	-0.38287	-0.06287	0.800223	0.681905	0.93907			

			method												
GCST90027809	Gut microbiota abundance (k_Bacteria.p_Proteobacteria.c_Betaproteobacteria.o_Burkholderiales.f_Burkholderiales_noname.g_Burkholderiales_noname.s_Burkholderiales_bacterium_1_1_47)	CAC	Robust adjusted profile score (RAPS)	14	-0.13912	0.059539	0.019463	-0.25581	-0.02242	0.870127	0.774287	0.97783			
GCST90027810	Gut microbiota abundance (k_Bacteria.p_Proteobacteria.c_Betaproteobacteria.o_Burkholderiales.f_Oxalobacteraceae.g_Oxalobacter.s_Oxalobacter_formigenes)	CAC	Inverse variance weighted	10	0.106911	0.051735	0.03878	0.00551	0.208311	1.112835	1.005526	1.231596	0.987958873	0.887424539	0.733478256
GCST90027810	Gut microbiota abundance (k_Bacteria.p_Proteobacteria.c_Betaproteobacteria.o_Burkholderiales.f_Oxalobacteraceae.g_Oxalobacter.s_Oxalobacter_formigenes)	CAC	MR Egger	10	0.102946	0.259883	0.70237	-0.40642	0.612316	1.108431	0.666027	1.844699		0.824953038	
GCST90027810	Gut microbiota abundance (k_Bacteria.p_Proteobacteria.c_Betaproteobacteria.o_Burkholderiales.f_Oxalobacteraceae.g_Oxalobacter.s_Oxalobacter_formigenes)	CAC	Weighted median	10	0.115197	0.068466	0.092463	-0.019	0.24939	1.122095	0.981183	1.283243			
GCST90027810	Gut microbiota abundance (k_Bacteria.p_Proteobacteria.c_Betaproteobacteria.o_Burkholderiales.f_Oxalobacteraceae.g_Oxalobacter.s_Oxalobacter_formigenes)	CAC	Weighted mode	10	0.147224	0.105232	0.195306	-0.05903	0.35348	1.158614	0.942678	1.424014			
GCST90027810	Gut microbiota abundance (k_Bacteria.p_Proteobacteria.c_Betaproteobacteria.o_Burkholderiales.f_Oxalobacteraceae.g_Oxalobacter.s_Oxalobacter_formigenes)	CAC	Contamination mixtur	10	0.130329	0.07398	0.033628	-0.01467	0.275329	1.139203	0.985436	1.316964			

			e method												
GCST90027810	Gut microbiota abundance (k_Bacteria.p_Proteobacteria.c_Betaproteobacteria.o_Burkholderiales.f_Oxalobacteraceae.g_Oxalobacter.s_Oxalobacter_formigenes)	CAC	Robust adjusted profile score (RAPS)	10	0.109781	0.057171	0.054829	-0.00227	0.221836	1.116034	0.997729	1.248367			
GCST90027814	Gut microbiota abundance (k_Bacteria.p_Proteobacteria.c_Deltaproteobacteria.o_Desulfovibrionales.f_Desulfovibrionaceae.g_Bilophila.s_Bilophila_wadsworthia)	CAC	Inverse variance weighted	7	0.207213	0.088353	0.019013	0.03404	0.380385	1.230244	1.034626	1.462848	0.587855674	0.691383253	0.733478256
GCST90027814	Gut microbiota abundance (k_Bacteria.p_Proteobacteria.c_Deltaproteobacteria.o_Desulfovibrionales.f_Desulfovibrionaceae.g_Bilophila.s_Bilophila_wadsworthia)	CAC	MR Egger	7	0.350505	0.262887	0.239951	-0.16475	0.865763	1.419785	0.848104	2.376819		0.614876782	
GCST90027814	Gut microbiota abundance (k_Bacteria.p_Proteobacteria.c_Deltaproteobacteria.o_Desulfovibrionales.f_Desulfovibrionaceae.g_Bilophila.s_Bilophila_wadsworthia)	CAC	Weighted median	7	0.275063	0.115038	0.0168	0.049588	0.500538	1.316614	1.050838	1.649609			
GCST90027814	Gut microbiota abundance (k_Bacteria.p_Proteobacteria.c_Deltaproteobacteria.o_Desulfovibrionales.f_Desulfovibrionaceae.g_Bilophila.s_Bilophila_wadsworthia)	CAC	Weighted mode	7	0.281373	0.162358	0.133791	-0.03685	0.599594	1.324947	0.963822	1.821378			
GCST90027814	Gut microbiota abundance (k_Bacteria.p_Proteobacteria.c_Deltaproteobacteria.o_Desulfovibrionales.f_Desulfovibrionaceae.g_Bilophila.s_Bilophila_wadsworthia)	CAC	Contamination	7	0.258169	0.109694	0.020053	0.043169	0.473169	1.294558	1.044115	1.605073			

			mixture method												
GCST90027814	Gut microbiota abundance (k_Bacteria.p_Proteobacteria.c_Deltaproteobacteria.o_Desulfovibrionales.f_Desulfovibrionaceae.g_Bilophila.s_Bilophila_wadsworthia)	CAC	Robust adjusted profile score (RAPS)	7	0.216031	0.098149	0.027732	0.023659	0.408404	1.241141	1.023941	1.504414			
GCST90027787	Gut microbiota abundance (k_Bacteria.p_Firmicutes.c_Clostridia.o_Clostridiales.f_Clostridiaceae.g_Clostridium.s_Clostridium_leptum)	CAC	Inverse variance weighted	7	-0.16472	0.058628	0.004961	-0.27963	-0.04981	0.848131	0.756063	0.951411	0.70377941	0.774811233	0.584523665
GCST90027787	Gut microbiota abundance (k_Bacteria.p_Firmicutes.c_Clostridia.o_Clostridiales.f_Clostridiaceae.g_Clostridium.s_Clostridium_leptum)	CAC	MR Egger	7	-0.03877	0.318182	0.907769	-0.6624	0.584868	0.961974	0.51561	1.794754		0.684004605	
GCST90027787	Gut microbiota abundance (k_Bacteria.p_Firmicutes.c_Clostridia.o_Clostridiales.f_Clostridiaceae.g_Clostridium.s_Clostridium_leptum)	CAC	Weighted median	7	-0.18275	0.078856	0.020475	-0.33731	-0.02819	0.832976	0.713689	0.972201			
GCST90027787	Gut microbiota abundance (k_Bacteria.p_Firmicutes.c_Clostridia.o_Clostridiales.f_Clostridiaceae.g_Clostridium.s_Clostridium_leptum)	CAC	Weighted mode	7	-0.22903	0.107769	0.077719	-0.44026	-0.01781	0.795301	0.643868	0.98235			

GCST90027787	Gut microbiota abundance (k_Bacteria.p_Firmicutes.c_Clostridia.o_Clostridiales.f_Clostridiaceae.g_Clostridium.s_Clostridium_leptum)	CAC	Contamination mixture method	7	-0.2125	0.079082	0.016564	-0.3675	-0.0575	0.808561	0.692464	0.944122			
GCST90027787	Gut microbiota abundance (k_Bacteria.p_Firmicutes.c_Clostridia.o_Clostridiales.f_Clostridiaceae.g_Clostridium.s_Clostridium_leptum)	CAC	Robust adjusted profile score (RAPS)	7	-0.16732	0.065562	0.010707	-0.29583	-0.03882	0.845926	0.743917	0.961922			
GCST90027725	Gut microbiota abundance (k_Bacteria.p_Proteobacteria.c_Betaproteobacteria.o_Burkholderiales.f_Burkholderiales_noname.g_Burkholderiales_noname)	CAC	Inverse variance weighted	14	-0.11176	0.054738	0.041172	-0.21905	-0.00448	0.894256	0.803282	0.995533	0.353783579	0.179365178	0.733478256
GCST90027725	Gut microbiota abundance (k_Bacteria.p_Proteobacteria.c_Betaproteobacteria.o_Burkholderiales.f_Burkholderiales_noname.g_Burkholderiales_noname)	CAC	MR Egger	14	-0.38284	0.28633	0.206004	-0.94404	0.178372	0.681925	0.389052	1.19527		0.182326002	
GCST90027725	Gut microbiota abundance (k_Bacteria.p_Proteobacteria.c_Betaproteobacteria.o_Burkholderiales.f_Burkholderiales_noname.g_Burkholderiales_noname)	CAC	Weighted median	14	-0.14882	0.067053	0.026453	-0.28025	-0.0174	0.86172	0.755595	0.98275			

GCST90027725	Gut microbiota abundance (k_Bacteria.p_Proteobacteria.c_Betaproteobacteria.o_Burkholderiales.f_Burkholderiales_noname.g_Burkholderiales_noname)	CAC	Weighted mode	14	-0.1937	0.130383	0.161227	-0.44925	0.061853	0.823907	0.638108	1.063806			
GCST90027725	Gut microbiota abundance (k_Bacteria.p_Proteobacteria.c_Betaproteobacteria.o_Burkholderiales.f_Burkholderiales_noname.g_Burkholderiales_noname)	CAC	Contamination mixture method	14	-0.22195	0.081633	0.008938	-0.38195	-0.06195	0.800955	0.682529	0.939929			
GCST90027725	Gut microbiota abundance (k_Bacteria.p_Proteobacteria.c_Betaproteobacteria.o_Burkholderiales.f_Burkholderiales_noname.g_Burkholderiales_noname)	CAC	Robust adjusted profile score (RAPS)	14	-0.13913	0.059542	0.019457	-0.25583	-0.02243	0.870116	0.774272	0.977823			
GCST90027680	Gut microbiota abundance (k_Bacteria.p_Proteobacteria.c_Betaproteobacteria.o_Burkholderiales.f_Burkholderiales_noname)	CAC	Inverse variance weighted	14	-0.11179	0.054695	0.040974	-0.21899	-0.00458	0.894236	0.803332	0.995427	0.350034577	0.179714882	0.733478256
GCST90027680	Gut microbiota abundance (k_Bacteria.p_Proteobacteria.c_Betaproteobacteria.o_Burkholderiales.f_Burkholderiales_noname)	CAC	MR Egger	14	-0.38454	0.285793	0.203333	-0.94469	0.175616	0.680765	0.3888	1.19198		0.183504939	

GCST90027680	Gut microbiota abundance (k_Bacteria.p_Proteobacteria.c_Betaproteobacteria.o_Burkholderiales.f_Burkholderiales_noname)	CAC	Weighted median	14	-0.1486	0.066185	0.024755	-0.27832	-0.01888	0.861914	0.757053	0.9813			
GCST90027680	Gut microbiota abundance (k_Bacteria.p_Proteobacteria.c_Betaproteobacteria.o_Burkholderiales.f_Burkholderiales_noname)	CAC	Weighted mode	14	-0.19585	0.129835	0.15536	-0.45032	0.05863	0.822139	0.637423	1.060383			
GCST90027680	Gut microbiota abundance (k_Bacteria.p_Proteobacteria.c_Betaproteobacteria.o_Burkholderiales.f_Burkholderiales_noname)	CAC	Contamination mixture method	14	-0.22005	0.081633	0.008941	-0.38005	-0.06005	0.802476	0.683825	0.941714			
GCST90027680	Gut microbiota abundance (k_Bacteria.p_Proteobacteria.c_Betaproteobacteria.o_Burkholderiales.f_Burkholderiales_noname)	CAC	Robust adjusted profile score (RAPS)	14	-0.13909	0.059489	0.01938	-0.25569	-0.0225	0.870146	0.77438	0.977756			
GCST90027681	Gut microbiota abundance (k_Bacteria.p_Proteobacteria.c_Betaproteobacteria.o_Burkholderiales.f_Oxalobacteraceae)	CAC	Inverse variance weighted	10	0.106575	0.051679	0.039182	0.005285	0.207865	1.112462	1.005299	1.231047	0.972544263	0.886127084	0.733478256

GCST90027681	Gut microbiota abundance (k_Bacteria.p_Proteobacteria.c_Betaproteobacteria.o_Burkholderiales.f_Oxalobacteraceae)	CAC	MR Egger	10	0.097519	0.260229	0.717591	-0.41253	0.607568	1.102432	0.661973	1.835961		0.82334733	
GCST90027681	Gut microbiota abundance (k_Bacteria.p_Proteobacteria.c_Betaproteobacteria.o_Burkholderiales.f_Oxalobacteraceae)	CAC	Weighted median	10	0.113751	0.067129	0.090167	-0.01782	0.245325	1.120474	0.982336	1.278036			
GCST90027681	Gut microbiota abundance (k_Bacteria.p_Proteobacteria.c_Betaproteobacteria.o_Burkholderiales.f_Oxalobacteraceae)	CAC	Weighted mode	10	0.1447	0.1085	0.215088	-0.06796	0.35736	1.155693	0.934298	1.42955			
GCST90027681	Gut microbiota abundance (k_Bacteria.p_Proteobacteria.c_Betaproteobacteria.o_Burkholderiales.f_Oxalobacteraceae)	CAC	Contamination mixture method	10	0.132038	0.07398	0.033937	-0.01296	0.277038	1.141152	0.987122	1.319216			
GCST90027681	Gut microbiota abundance (k_Bacteria.p_Proteobacteria.c_Betaproteobacteria.o_Burkholderiales.f_Oxalobacteraceae)	CAC	Robust adjusted profile score (RAPS)	10	0.10945	0.057082	0.055184	-0.00243	0.22133	1.115664	0.997573	1.247735			

nsnp: number of single nucleotide polymorphisms, se: standard error, lo_ci: lower confidence interval, up_ci: upper confidence interval, or: odds ratio, or_lci95: odds ratio lower 95% confidence interval limit, or_uci95: odds ratio upper 95% confidence interval limit, p-FDR: p-value after False Discovery Rate correction

Table S3 Mendelian Randomization analysis on the causal effect between coronary artery calcification and gut microbiota.

id.exposure	id.outcome	outcome	exposure	method	nsnp	b	se	pval	lo_ci	up_ci	or	or_lci95	or_uci95	pleiotropy	heterogeneity	p_fdr
CAC	ebi-a-GCST90027691	Gut microbiota abundance (k_Bacteria.p_Actinobacteria.c_Actinobacteria.o_Coriobacteriales.f_Coriobacteriaceae.g_Eggerthella) || id:ebi-a-GCST90027691	exposure	Inverse variance weighted	3	0.22938956	0.114984376	0.046046931	0.004020182	0.454758937	1.257831944	1.004028274	1.575793472	0.874841901	0.965204049	0.978570886
CAC	ebi-a-GCST90027691	Gut microbiota abundance (k_Bacteria.p_Actinobacteria.c_Actinobacteria.o_Coriobacteriales.f_Coriobacteriaceae.g_Eggerthella) || id:ebi-a-GCST90027691	exposure	MR Egger	3	0.340398839	0.569095107	0.656829406	-0.77502757	1.455825249	1.40550805	0.460691079	4.288020689		0.859878133	0.99887487
CAC	ebi-a-GCST90027691	Gut microbiota abundance (k_Bacteria.p_Actinobacteria.c_Actinobacteria.o_Coriobacteriales.f_Coriobacteriaceae.g_Eggerthella) || id:ebi-a-GCST90027691	exposure	Weighted median	3	0.231795758	0.130426901	0.075533991	-0.023840968	0.487432484	1.260862181	0.976440982	1.628130598			0.954421078
CAC	ebi-a-GCST90027691	Gut microbiota abundance (k_Bacteria.p_Actinobacteria.c_Actinobacteria.o_Coriobacteriales.f_Coriobacteriaceae.g_Eggerthella) || id:ebi-a-GCST90027691	exposure	Weighted mode	3	0.238059396	0.138933107	0.228757276	-0.034249494	0.510368286	1.268784552	0.966330381	1.665904612			0.999350579
CAC	ebi-a-GCST90027691	Gut microbiota abundance (k_Bacteria.p_Actinobacteria.c_Actinobacteria.o_Coriobacteriales.f_Coriobacteriaceae.g_Eggerthella) || id:ebi-a-GCST90027691	exposure	Contamination mixtur	3	0.224891723	0.147959184	0.054823008	-0.065108277	0.514891723	1.252187125	0.936966006	1.673457294			0.808726186

				e method												
CAC	ebi-a-GCST90027691	Gut microbiota abundance (k_Bacteria.p_Actinobacteria.c_Actinobacteria.o_Coriobacteriales.f_Coriobacteriaceae.g_Eggerthella) || id:ebi-a-GCST90027691	exposure	Robust adjusted profile score (RAPS)	3	0.229580718	0.122244369	0.060374681	-0.010018244	0.469179681	1.258072413	0.990031771	1.598682225			0.949852223
CAC	ebi-a-GCST90027718	Gut microbiota abundance (k_Bacteria.p_Firmicutes.c_Clostridia.o_Clostridiales.f_Ruminococcaceae.g_Ruminococcus) || id:ebi-a-GCST90027718	exposure	Inverse variance weighted	3	-0.123822325	0.061960026	0.045670849	-0.245263977	-0.002380673	0.883536809	0.78249795	0.997622158	0.945687337	0.415960498	0.978570886
CAC	ebi-a-GCST90027718	Gut microbiota abundance (k_Bacteria.p_Firmicutes.c_Clostridia.o_Clostridiales.f_Ruminococcaceae.g_Ruminococcus) || id:ebi-a-GCST90027718	exposure	MR Egger	3	-0.461858709	0.3061174	0.372624414	-1.061848812	0.138131395	0.630111363	0.34581587	1.148126398		0.487145233	0.99887487
CAC	ebi-a-GCST90027718	Gut microbiota abundance (k_Bacteria.p_Firmicutes.c_Clostridia.o_Clostridiales.f_Ruminococcaceae.g_Ruminococcus) || id:ebi-a-GCST90027718	exposure	Weighted median	3	-0.136425141	0.07356226	0.063660027	-0.280607171	0.00775689	0.87247163	0.755324991	1.007787052			0.954421078
CAC	ebi-a-GCST9	Gut microbiota abundance (k_Bacteria.p_Firmicutes.c_Clostridia.o_Clostridiales.f_Ruminococcaceae.g_Ruminococcus) || id:ebi-a-GCST90027718	exposure	Weighted mode	3	-0.166	0.081581898	0.17793497	-0.326	-0.006	0.846561737	0.721464094	0.993350578			0.999350579

	0027718					572148			472668	671628						
CAC	ebi-a-GCST90027718	Gut microbiota abundance (k_Bacteria.p_Firmicutes.c_Clostridia.o_Clostridiales.f_Ruminococcaceae.g_Ruminococcus) || id:ebi-a-GCST90027718	exposure	Contamination mixture method	3	-0.168390604	0.079081633	0.092240882	-0.323390604	-0.013390604	0.845023701	0.723691122	0.986698651			0.808726186
CAC	ebi-a-GCST90027718	Gut microbiota abundance (k_Bacteria.p_Firmicutes.c_Clostridia.o_Clostridiales.f_Ruminococcaceae.g_Ruminococcus) || id:ebi-a-GCST90027718	exposure	Robust adjusted profile score (RAPS)	3	-0.125442396	0.06568619	0.05616895	-0.254187329	0.003302536	0.882106575	0.775546506	1.003307996			0.949852223
CAC	ebi-a-GCST90027769	Gut microbiota abundance (k_Bacteria.p_Bacteroidetes.c_Bacteroidia.o_Bacteroidales.f_Porphyromonadaceae.g_Parabacteroides.s_Parabacteroides_merdae) || id:ebi-a-GCST90027769	exposure	Inverse variance weighted	3	0.144527745	0.065711241	0.027846852	0.015733714	0.273321777	1.155493754	1.01585814	1.314323096	0.821416257	0.893984097	0.978570886
CAC	ebi-a-GCST90027769	Gut microbiota abundance (k_Bacteria.p_Bacteroidetes.c_Bacteroidia.o_Bacteroidales.f_Porphyromonadaceae.g_Parabacteroides.s_Parabacteroides_merdae) || id:ebi-a-GCST90027769	exposure	MR Egger	3	0.052621582	0.325688019	0.898022296	-0.585726935	0.690970099	1.054030706	0.556701029	1.995650574		0.707166385	0.99887487

CAC	ebi-a-GCST90027769	Gut microbiota abundance (k_Bacteria.p_Bacteroidetes.c_Bacteroidia.o_Bacteroidales.f_Porphyromonadaceae.g_Parabacteroides.s_Parabacteroides_merdae) || id:ebi-a-GCST90027769	exposure	Weighted median	3	0.140156925	0.070460992	0.04668552	0.002053381	0.278260468	1.15045432	1.002055491	1.320830187			0.954421078
CAC	ebi-a-GCST90027769	Gut microbiota abundance (k_Bacteria.p_Bacteroidetes.c_Bacteroidia.o_Bacteroidales.f_Porphyromonadaceae.g_Parabacteroides.s_Parabacteroides_merdae) || id:ebi-a-GCST90027769	exposure	Weighted mode	3	0.13067858	0.082000358	0.25204321	-0.030042121	0.291399282	1.139601432	0.970404658	1.338298836			0.999350579
CAC	ebi-a-GCST90027769	Gut microbiota abundance (k_Bacteria.p_Bacteroidetes.c_Bacteroidia.o_Bacteroidales.f_Porphyromonadaceae.g_Parabacteroides.s_Parabacteroides_merdae) || id:ebi-a-GCST90027769	exposure	Contamination mixture method	3	0.143472036	0.066326531	0.03587661	0.013472036	0.273472036	1.154274532	1.013563193	1.314520599			0.808726186
CAC	ebi-a-GCST90027769	Gut microbiota abundance (k_Bacteria.p_Bacteroidetes.c_Bacteroidia.o_Bacteroidales.f_Porphyromonadaceae.g_Parabacteroides.s_Parabacteroides_merdae) || id:ebi-a-GCST90027769	exposure	Robust adjusted profile score (RAPS)	3	0.144682558	0.069976007	0.038677429	0.007529584	0.281835532	1.155672653	1.007558003	1.32556069			0.949852223
CAC	ebi-a-GCST90027776	Gut microbiota abundance (k_Bacteria.p_Bacteroidetes.c_Bacteroidia.o_Bacteroidales.f_Rikenellaceae.g_Alistipes.s_Alistipes_indistinctus) || id:ebi-a-GCST90027776	exposure	Inverse variance	3	-0.182215718	0.068875982	0.008155521	-0.317212642	-0.047218794	0.833421537	0.728175898	0.953878672	0.568471314	0.518550932	0.978570886

				weighted												
CAC	ebi-a-GCST90027776	Gut microbiota abundance (k_Bacteria.p_Bacteroidetes.c_Bacteroidia.o_Bacteroidales.f_Rikenellaceae.g_Alistipes.s_Alistipes_indistinctus) || id:ebi-a-GCST90027776	exposure	MR Egger	3	-0.451262515	0.341201684	0.412145506	-1.120017815	0.217492785	0.636823645	0.326273982	1.242956462		0.414713933	0.99887487
CAC	ebi-a-GCST90027776	Gut microbiota abundance (k_Bacteria.p_Bacteroidetes.c_Bacteroidia.o_Bacteroidales.f_Rikenellaceae.g_Alistipes.s_Alistipes_indistinctus) || id:ebi-a-GCST90027776	exposure	Weighted median	3	-0.183720018	0.07962084	0.021030508	-0.339776864	-0.027663172	0.832168764	0.711929162	0.97271595			0.954421078
CAC	ebi-a-GCST90027776	Gut microbiota abundance (k_Bacteria.p_Bacteroidetes.c_Bacteroidia.o_Bacteroidales.f_Rikenellaceae.g_Alistipes.s_Alistipes_indistinctus) || id:ebi-a-GCST90027776	exposure	Weighted mode	3	-0.197299373	0.091458996	0.163689387	-0.376559006	-0.01803974	0.820944828	0.686218625	0.982122002			0.999350579
CAC	ebi-a-GCST90027776	Gut microbiota abundance (k_Bacteria.p_Bacteroidetes.c_Bacteroidia.o_Bacteroidales.f_Rikenellaceae.g_Alistipes.s_Alistipes_indistinctus) || id:ebi-a-GCST90027776	exposure	Contamination mixture method	3	-0.179492617	0.089285714	0.056297886	-0.354492617	-0.004492617	0.83569412	0.701529297	0.995517459			0.808726186
CAC	ebi-a-GCST90027776	Gut microbiota abundance (k_Bacteria.p_Bacteroidetes.c_Bacteroidia.o_Bacteroidales.f_Rikenellaceae.g_Alistipes.s_Alistipes_indistinctus) || id:ebi-a-GCST90027776	exposure	Robust adjusted profile score	3	-0.184021637	0.073734169	0.012569315	-0.328540609	-0.039502665	0.831917803	0.71997369	0.961267392			0.949852223

				(RAPS)												
CAC	ebi-a-GCST90027799	Gut microbiota abundance (k_Bacteria.p_Firmicutes.c_Clostridia.o_Clostridiales.f_Ruminococcaceae.g_Ruminococcus.s_Ruminococcus_bromii) || id:ebi-a-GCST90027799	exposure	Inverse variance weighted	3	-0.149235862	0.0714070211	0.0366238522	-0.2891936223	-0.0092781022	0.8613659277	0.7488671944	0.9907648077	0.8702256444	0.8618402775	0.9785708866
CAC	ebi-a-GCST90027799	Gut microbiota abundance (k_Bacteria.p_Firmicutes.c_Clostridia.o_Clostridiales.f_Ruminococcaceae.g_Ruminococcus.s_Ruminococcus_bromii) || id:ebi-a-GCST90027799	exposure	MR Egger	3	-0.0777487691	0.3531107533	0.8620290477	-0.7698458444	0.6143483066	0.9251968366	0.46308445	1.8484515822		0.61382857	0.99887487
CAC	ebi-a-GCST90027799	Gut microbiota abundance (k_Bacteria.p_Firmicutes.c_Clostridia.o_Clostridiales.f_Ruminococcaceae.g_Ruminococcus.s_Ruminococcus_bromii) || id:ebi-a-GCST90027799	exposure	Weighted median	3	-0.1383436933	0.0783654933	0.0775024555	-0.29194006	0.0152526744	0.8707993533	0.7468132999	1.01536959			0.9544210788
CAC	ebi-a-GCST90027799	Gut microbiota abundance (k_Bacteria.p_Firmicutes.c_Clostridia.o_Clostridiales.f_Ruminococcaceae.g_Ruminococcus.s_Ruminococcus_bromii) || id:ebi-a-GCST90027799	exposure	Weighted mode	3	-0.1325684533	0.0838307222	0.2545929222	-0.2968766677	0.0317397622	0.8758429788	0.74313566	1.03224884			0.9993505799
CAC	ebi-a-GCST90027799	Gut microbiota abundance (k_Bacteria.p_Firmicutes.c_Clostridia.o_Clostridiales.f_Ruminococcaceae.g_Ruminococcus.s_Ruminococcus_bromii) || id:ebi-a-GCST90027799	exposure	Contamination mixture method	3	-0.1490979877	0.0739795922	0.0450796355	-0.2940979877	-0.0040979877	0.8614846977	0.7452034688	0.9959103999			0.8087261866

CAC	ebi-a-GCST90027799	Gut microbiota abundance (k_Bacteria.p_Firmicutes.c_Clostridia.o_Clostridiales.f_Ruminococcaceae.g_Ruminococcus.s_Ruminococcus_bromii) || id:ebi-a-GCST90027799	exposure	Robust adjusted profile score (RAPS)	3	-0.149474612	0.075958246	0.049085031	-0.298352774	-0.000596449	0.861160301	0.742039521	0.999403729			0.949852223
CAC	ebi-a-GCST90027827	Gut microbiota abundance (k_Bacteria.p_Bacteroidetes.c_Bacteroidia.o_Bacteroidales.f_Bacteroidaceae.g_Bacteroides.s_Bacteroides_finegoldii) || id:ebi-a-GCST90027827	exposure	Inverse variance weighted	3	0.256234412	0.117454579	0.029141896	0.026023437	0.486445388	1.292055566	1.026365003	1.626524269	0.393835641	0.365598666	0.978570886
CAC	ebi-a-GCST90027827	Gut microbiota abundance (k_Bacteria.p_Bacteroidetes.c_Bacteroidia.o_Bacteroidales.f_Bacteroidaceae.g_Bacteroides.s_Bacteroides_finegoldii) || id:ebi-a-GCST90027827	exposure	MR Egger	3	1.053244155	0.579309056	0.320131425	-0.082201595	2.188689904	2.866936834	0.921086254	8.923514793		0.843431066	0.99887487
CAC	ebi-a-GCST90027827	Gut microbiota abundance (k_Bacteria.p_Bacteroidetes.c_Bacteroidia.o_Bacteroidales.f_Bacteroidaceae.g_Bacteroides.s_Bacteroides_finegoldii) || id:ebi-a-GCST90027827	exposure	Weighted median	3	0.318020626	0.133500918	0.01721139	0.056358827	0.579682424	1.374404609	1.057977247	1.785471319			0.954421078
CAC	ebi-a-GCST90027827	Gut microbiota abundance (k_Bacteria.p_Bacteroidetes.c_Bacteroidia.o_Bacteroidales.f_Bacteroidaceae.g_Bacteroides.s_Bacteroides_finegoldii) || id:ebi-a-GCST90027827	exposure	Weighted mode	3	0.353399964	0.145546203	0.135884401	0.068129406	0.638670522	1.423900538	1.070503829	1.893961224			0.999350579

CAC	ebi-a-GCST90027827	Gut microbiota abundance (k_Bacteria.p_Bacteroidetes.c_Bacteroidia.o_Bacteroidales.f_Bacteroidaceae.g_Bacteroides.s_Bacteroides_finegoldii) || id:ebi-a-GCST90027827	exposure	Contamination mixture method	3	0.347149441	0.150510204	0.1126514	0.052149441	0.642149441	1.415028172	1.053533172	1.900561637			0.808726186
CAC	ebi-a-GCST90027827	Gut microbiota abundance (k_Bacteria.p_Bacteroidetes.c_Bacteroidia.o_Bacteroidales.f_Bacteroidaceae.g_Bacteroides.s_Bacteroides_finegoldii) || id:ebi-a-GCST90027827	exposure	Robust adjusted profile score (RAPS)	3	0.25996046	0.124236643	0.036397204	0.01645664	0.50346428	1.296878807	1.016592796	1.654442807			0.949852223

nsnp: number of single nucleotide polymorphisms, se: standard error, lo_ci: lower confidence interval, up_ci: upper confidence interval, or: odds ratio, or_lci95: odds ratio lower 95% confidence interval limit, or_uci95: odds ratio upper 95% confidence interval limit, p-FDR: p-value after False Discovery Rate correction

Table S4 Mendelian Randomization analysis on the causal effect between circulating metabolites and coronary artery calcification.

id.exposure	id.outcome	outcome	exposure	method	nsnp	b	se	pval	lo_ci	up_ci	or	or_lci95	or_uci95	pleiotropy	heterogeneity	p-fdr
GCST90199630	CAC	outcome	Phenylacetate levels	Inverse variance weighted	24	0.196502275	0.058333997	0.000755586	0.082167641	0.310836909	1.21713809	1.085637792	1.364566655	0.585201428	0.675611569	
GCST90199630	CAC	outcome	Phenylacetate levels	MR Egger	24	0.255176267	0.120920736	0.046435216	0.018171624	0.492180911	1.290689107	1.018337732	1.635880041		0.637301655	
GCST90199630	CAC	outcome	Phenylacetate levels	Weighted median	24	0.238344943	0.086010031	0.005586219	0.069765283	0.406924604	1.269146901	1.072256475	1.502190841			
GCST90199630	CAC	outcome	Phenylacetate levels	Weighted mode	24	0.239875437	0.125527885	0.068555815	-0.006159217	0.48591009	1.271090809	0.993859712	1.625653827			
GCST90199630	CAC	outcome	Phenylacetate levels	Contamination mixture method	24	0.2751107	0.091836735	0.00207439	0.0951107	0.4551107	1.316676422	1.099780594	1.576347875			
GCST90199630	CAC	outcome	Phenylacetate levels	Robust adjusted profile score (RAPS)	24	0.21184252	0.063710681	0.000883961	0.086969585	0.336715455	1.235953232	1.090863501	1.400340547			
GCST90199660	CAC	outcome	1,2-dipalmitoyl-gpc (16:0/16:0) levels	Inverse variance weighted	26	0.11186403	0.051845099	0.030954374	0.010247637	0.213480424	1.118360787	1.010300324	1.237979263	0.684162507	0.139034921	
GCST90199660	CAC	outcome	1,2-dipalmitoyl-gpc (16:0/16:0) levels	MR Egger	26	0.156361908	0.120242655	0.205818014	-0.079313695	0.392037511	1.169249287	0.923750103	1.479993226		0.11600321	
GCST90199660	CAC	outcome	1,2-dipalmitoyl-gpc (16:0/16:0) levels	Weighted median	26	0.04904881	0.067166893	0.465235686	-0.082598299	0.180695919	1.050271613	0.920720927	1.19805082			

GCST90199660	CAC	outcome	1,2-dipalmitoyl-gpc (16:0/16:0) levels	Weighted mode	26	0.092068201	0.08256134	0.275391937	-0.069752025	0.253888427	1.096439598	0.932625059	1.289027976			
GCST90199660	CAC	outcome	1,2-dipalmitoyl-gpc (16:0/16:0) levels	Contamination mixture method	26	0.24285293	0.089285714	0.019254005	0.06785293	0.41785293	1.274881113	1.070207901	1.518697303			
GCST90199660	CAC	outcome	1,2-dipalmitoyl-gpc (16:0/16:0) levels	Robust adjusted profile score (RAPS)	26	0.126106629	0.055250128	0.022461747	0.017816378	0.234396881	1.134403122	1.017976036	1.264146108			
GCST90199695	CAC	outcome	Galactonate levels	Inverse variance weighted	16	0.160094278	0.073028809	0.028364551	0.016957813	0.303230743	1.173621513	1.017102413	1.354226907	0.364910393	0.879619077	
GCST90199695	CAC	outcome	Galactonate levels	MR Egger	16	0.025075204	0.161620409	0.878918718	-0.291700798	0.341851206	1.025392231	0.746992004	1.407550846		0.884974911	
GCST90199695	CAC	outcome	Galactonate levels	Weighted median	16	0.117992262	0.102314195	0.248814134	-0.082543559	0.318528084	1.125235405	0.920771329	1.375102239			
GCST90199695	CAC	outcome	Galactonate levels	Weighted mode	16	0.090030586	0.166772582	0.597230911	-0.236843676	0.416904847	1.09420775	0.789114636	1.517258135			
GCST90199695	CAC	outcome	Galactonate levels	Contamination mixture method	16	0.216770137	0.137755102	0.129728599	-0.053229863	0.486770137	1.242058565	0.94816204	1.627052566			
GCST90199695	CAC	outcome	Galactonate levels	Robust adjusted profile score (RAPS)	16	0.1598917	0.079423007	0.044097167	0.004222605	0.315560794	1.173383786	1.004231533	1.37102796			

GCST90199709	CAC	outcome	Adrenate (22:4n6) levels	Inverse variance weighted	21	-0.11824263	0.057775988	0.040700279	-0.231483567	-0.005001693	0.888480454	0.793355732	0.995010795	0.579482927	0.805052705	
GCST90199709	CAC	outcome	Adrenate (22:4n6) levels	MR Egger	21	-0.052791436	0.129671418	0.688472052	-0.306947415	0.201364543	0.948577831	0.735689286	1.223070552		0.773833931	
GCST90199709	CAC	outcome	Adrenate (22:4n6) levels	Weighted median	21	-0.025832314	0.079060996	0.743865266	-0.180791866	0.129127237	0.974498485	0.834609051	1.13783489			
GCST90199709	CAC	outcome	Adrenate (22:4n6) levels	Weighted mode	21	-0.032752064	0.098217949	0.742255415	-0.225259244	0.159755115	0.967778477	0.798309235	1.173223531			
GCST90199709	CAC	outcome	Adrenate (22:4n6) levels	Contamination mixture method	21	-0.079344686	0.109693878	0.207805361	-0.294344686	0.135655314	0.923721476	0.74501965	1.145287062			
GCST90199709	CAC	outcome	Adrenate (22:4n6) levels	Robust adjusted profile score (RAPS)	21	-0.114884709	0.062332487	0.065315109	-0.237056384	0.007286965	0.891468916	0.788946803	1.00731358			
GCST90199725	CAC	outcome	Campesterol levels	Inverse variance weighted	21	0.119550634	0.042412538	0.004821001	0.036422061	0.202679208	1.126990307	1.037093471	1.224679538	0.281826194	0.229254893	
GCST90199725	CAC	outcome	Campesterol levels	MR Egger	21	0.032065975	0.089534865	0.724187029	-0.143422359	0.20755431	1.032585628	0.866388065	1.230664552		0.244813762	
GCST90199725	CAC	outcome	Campesterol levels	Weighted median	21	0.046396548	0.054946877	0.398451975	-0.061299331	0.154092427	1.047489708	0.940541664	1.166598707			

GCST90199725	CAC	outcome	Campesterol levels	Weighted mode	21	0.042392075	0.0595224	0.484564819	-0.07427183	0.159055979	1.043303451	0.928419287	1.172403575			
GCST90199725	CAC	outcome	Campesterol levels	Contamination mixture method	21	0.050754872	0.135204082	0.44880063	-0.214245128	0.315754872	1.052064971	0.807150505	1.371294072			
GCST90199725	CAC	outcome	Campesterol levels	Robust adjusted profile score (RAPS)	21	0.100860434	0.042437909	0.017470236	0.017682131	0.184038736	1.106122254	1.017839386	1.202062386			
GCST90199740	CAC	outcome	P-cresol sulfate levels	Inverse variance weighted	19	-0.160690083	0.072061843	0.025754653	-0.301931295	-0.019448872	0.851555942	0.739388863	0.980739037	0.138572816	0.728788536	
GCST90199740	CAC	outcome	P-cresol sulfate levels	MR Egger	19	-0.419317191	0.181345769	0.033555477	-0.774754897	-0.063879484	0.657495611	0.460816714	0.938118051		0.824341457	
GCST90199740	CAC	outcome	P-cresol sulfate levels	Weighted median	19	-0.121079638	0.104111529	0.244837569	-0.325138234	0.082978958	0.8859634	0.722427483	1.086518946			
GCST90199740	CAC	outcome	P-cresol sulfate levels	Weighted mode	19	-0.228520062	0.179609985	0.219453155	-0.580555632	0.123515509	0.795710334	0.559587355	1.131467552			
GCST90199740	CAC	outcome	P-cresol sulfate levels	Contamination mixture method	19	-0.263797673	0.117346939	0.028968632	-0.493797673	-0.033797673	0.768128937	0.610304251	0.966767088			
GCST90199740	CAC	outcome	P-cresol sulfate levels	Robust adjusted profile score (RAPS)	19	-0.165925495	0.078719436	0.03504756	-0.32021559	-0.011635399	0.847109346	0.725992503	0.98843203			

GCST90199765	CAC	outcome	4-hydroxyhippurate levels	Inverse variance weighted	19	-0.157325224	0.07366509	0.032705323	-0.3017088	-0.012941648	0.854426134	0.739553392	0.987141735	0.444786915	0.625052863	
GCST90199765	CAC	outcome	4-hydroxyhippurate levels	MR Egger	19	-0.025210784	0.184242484	0.892768845	-0.386326053	0.335904486	0.975104354	0.679548921	1.399205375		0.601121701	
GCST90199765	CAC	outcome	4-hydroxyhippurate levels	Weighted median	19	-0.107097211	0.102189212	0.294625454	-0.307388066	0.093193644	0.89843833	0.735365175	1.097674273			
GCST90199765	CAC	outcome	4-hydroxyhippurate levels	Weighted mode	19	-0.093685204	0.152819939	0.547516409	-0.393212283	0.205841876	0.910569361	0.674885465	1.228558924			
GCST90199765	CAC	outcome	4-hydroxyhippurate levels	Contamination mixture method	19	-0.091228787	0.135204082	0.263403302	-0.356228787	0.173771213	0.912808848	0.700312379	1.189783327			
GCST90199765	CAC	outcome	4-hydroxyhippurate levels	Robust adjusted profile score (RAPS)	19	-0.142216516	0.079828512	0.074826671	-0.298680399	0.014247367	0.867433423	0.741796451	1.014349345			
GCST90199772	CAC	outcome	1-stearoyl-GPE (18:0) levels	Inverse variance weighted	41	0.084315712	0.040380312	0.036794215	0.0051703	0.163461124	1.087972326	1.005183689	1.177579575	0.420218716	0.300670102	
GCST90199772	CAC	outcome	1-stearoyl-GPE (18:0) levels	MR Egger	41	0.160176742	0.101567152	0.122861416	-0.038894876	0.359248361	1.173718298	0.961851817	1.432252473		0.28899498	
GCST90199772	CAC	outcome	1-stearoyl-GPE (18:0) levels	Weighted median	41	0.123520042	0.057461856	0.031586819	0.010894804	0.236145279	1.131472681	1.010954368	1.266358272			

GCST90199772	CAC	outcome	1-stearoyl-GPE (18:0) levels	Weighted mode	41	0.102722298	0.069501018	0.147240186	-0.033499698	0.238944294	1.108183621	0.967055204	1.269907793			
GCST90199772	CAC	outcome	1-stearoyl-GPE (18:0) levels	Contamination mixture method	41	0.122505323	0.056122449	0.014008472	0.012505323	0.232505323	1.130325137	1.012583841	1.261757163			
GCST90199772	CAC	outcome	1-stearoyl-GPE (18:0) levels	Robust adjusted profile score (RAPS)	41	0.097112947	0.043011536	0.023955956	0.012810335	0.181415558	1.101984832	1.012892739	1.198913293			
GCST90199827	CAC	outcome	Thymol sulfate levels	Inverse variance weighted	18	0.13609872	0.064355923	0.034448569	0.00996111	0.262236329	1.145795	1.010010887	1.299833695	0.528706029	0.981430021	
GCST90199827	CAC	outcome	Thymol sulfate levels	MR Egger	18	0.247945246	0.18521744	0.199389434	-0.115080936	0.610971428	1.281389769	0.891294004	1.842220114		0.977922748	
GCST90199827	CAC	outcome	Thymol sulfate levels	Weighted median	18	0.087182546	0.084529414	0.302359339	-0.078495107	0.252860198	1.091095836	0.924506584	1.28770324			
GCST90199827	CAC	outcome	Thymol sulfate levels	Weighted mode	18	0.038936435	0.147618302	0.795132242	-0.250395437	0.328268308	1.039704393	0.778492878	1.388561484			
GCST90199827	CAC	outcome	Thymol sulfate levels	Contamination mixture method	18	0.086307092	0.119897959	0.199682096	-0.148692908	0.321307092	1.09014105	0.861833736	1.378928974			
GCST90199827	CAC	outcome	Thymol sulfate levels	Robust adjusted profile score (RAPS)	18	0.125346993	0.069828252	0.072641651	-0.01151638	0.262210366	1.133541716	0.988549679	1.299799947			

GCST90199841	CAC	outcome	Glycocholenate sulfate levels	Inverse variance weighted	33	-0.068032674	0.029655719	0.021785745	-0.126157883	-0.009907465	0.934229948	0.881475666	0.990141452	0.323101629	0.628599904	
GCST90199841	CAC	outcome	Glycocholenate sulfate levels	MR Egger	33	-0.032204796	0.046396375	0.492774317	-0.12314169	0.058732099	0.968308256	0.88413838	1.060491096		0.631180871	
GCST90199841	CAC	outcome	Glycocholenate sulfate levels	Weighted median	33	-0.042254516	0.041187431	0.304934939	-0.122981882	0.038472849	0.958625764	0.884279684	1.039222512			
GCST90199841	CAC	outcome	Glycocholenate sulfate levels	Weighted mode	33	-0.048808365	0.035537884	0.17916925	-0.118462618	0.020845888	0.952363618	0.888285021	1.021064681			
GCST90199841	CAC	outcome	Glycocholenate sulfate levels	Contamination mixture method	33	-0.048819262	0.030612245	0.131285911	-0.108819262	0.011180738	0.952353241	0.896892506	1.011243476			
GCST90199841	CAC	outcome	Glycocholenate sulfate levels	Robust adjusted profile score (RAPS)	33	-0.060218552	0.030927748	0.0515261	-0.120836938	0.000399833	0.941558731	0.88617845	1.000399913			
GCST90199851	CAC	outcome	Androstenediol (3beta,17beta) disulfate (2) levels	Inverse variance weighted	36	-0.138661893	0.050964256	0.006513055	-0.238551835	-0.038771951	0.870522308	0.787767854	0.96197006	0.804566539	0.299148209	
GCST90199851	CAC	outcome	Androstenediol (3beta,17beta) disulfate (2) levels	MR Egger	36	-0.168604151	0.130708773	0.205782701	-0.424793346	0.087585043	0.844843267	0.653904903	1.091535088		0.261760348	
GCST90199851	CAC	outcome	Androstenediol (3beta,17beta) disulfate (2) levels	Weighted median	36	-0.091251321	0.078538469	0.245289149	-0.24518672	0.062684077	0.912788279	0.782558405	1.064690426			

GCST90199851	CAC	outcome	Androstenediol (3beta,17beta) disulfate (2) levels	Weighted mode	36	-0.072723853	0.102969191	0.484696691	-0.274543466	0.129095761	0.929857572	0.759918973	1.137799075			
GCST90199851	CAC	outcome	Androstenediol (3beta,17beta) disulfate (2) levels	Contamination mixture method	36	-0.228847245	0.125	0.045962739	-0.473847245	0.016152755	0.795450034	0.622602351	1.016283916			
GCST90199851	CAC	outcome	Androstenediol (3beta,17beta) disulfate (2) levels	Robust adjusted profile score (RAPS)	36	-0.148819979	0.05778637	0.010013944	-0.262081265	-0.035558694	0.861724229	0.769448492	0.965066089			
GCST90199902	CAC	outcome	Hydantoin-5-propionate levels	Inverse variance weighted	16	-0.156669162	0.07457125	0.035646892	-0.302828811	-0.010509513	0.854986874	0.738725547	0.989545519	0.113867323	0.25968881	
GCST90199902	CAC	outcome	Hydantoin-5-propionate levels	MR Egger	16	-0.471984858	0.199780882	0.033162521	-0.863555386	-0.08041433	0.623762958	0.421660249	0.922733951		0.37759832	
GCST90199902	CAC	outcome	Hydantoin-5-propionate levels	Weighted median	16	-0.103879105	0.103532086	0.315691152	-0.306801994	0.099043784	0.901334258	0.735796279	1.104114641			
GCST90199902	CAC	outcome	Hydantoin-5-propionate levels	Weighted mode	16	-0.109699507	0.170682947	0.53011504	-0.444238083	0.22483907	0.896103368	0.641312717	1.252121196			
GCST90199902	CAC	outcome	Hydantoin-5-propionate levels	Contamination mixture method	16	-0.147889533	0.12755102	0.131865297	-0.397889533	0.102110467	0.862526391	0.671736228	1.107505808			
GCST90199902	CAC	outcome	Hydantoin-5-propionate levels	Robust adjusted profile score (RAPS)	16	-0.149168799	0.075074898	0.046929812	-0.296315599	-0.002021999	0.861423695	0.743552726	0.997980044			

GCST90199905	CAC	outcome	Formiminoglutamate levels	Inverse variance weighted	20	-0.246276199	0.066850716	0.000229627	-0.377303603	-0.115248796	0.781706288	0.685707859	0.891144403	0.326949188	0.924879024	
GCST90199905	CAC	outcome	Formiminoglutamate levels	MR Egger	20	-0.416724737	0.181875999	0.034228064	-0.773201695	-0.06024778	0.659202349	0.461533012	0.941531212		0.933414967	
GCST90199905	CAC	outcome	Formiminoglutamate levels	Weighted median	20	-0.196187328	0.09265954	0.034235051	-0.377800026	-0.014574629	0.821858263	0.685367542	0.985531067			
GCST90199905	CAC	outcome	Formiminoglutamate levels	Weighted mode	20	-0.098981233	0.168753778	0.564415595	-0.429738639	0.231776172	0.905759706	0.650679135	1.260837486			
GCST90199905	CAC	outcome	Formiminoglutamate levels	Contamination mixture method	20	-0.377402898	0.12244898	0.00465577	-0.617402898	-0.137402898	0.685639776	0.53934335	0.871618982			
GCST90199905	CAC	outcome	Formiminoglutamate levels	Robust adjusted profile score (RAPS)	20	-0.244073454	0.073661313	0.000921509	-0.388449627	-0.099697282	0.783430086	0.67810738	0.90511137			
GCST90199935	CAC	outcome	Glycohyocholate levels	Inverse variance weighted	17	0.162478486	0.06317713	0.010117343	0.038651312	0.286305661	1.176423009	1.039407991	1.33149938	0.792529667	0.961210487	
GCST90199935	CAC	outcome	Glycohyocholate levels	MR Egger	17	0.124449253	0.155444058	0.435858297	-0.18022011	0.429119606	1.132524547	0.835085554	1.535904727		0.943077564	
GCST90199935	CAC	outcome	Glycohyocholate levels	Weighted median	17	0.126826541	0.085987757	0.140229431	-0.041709463	0.295362546	1.135220087	0.959148408	1.343613392			

GCST90199935	CAC	outcome	Glycohyocholate levels	Weighted mode	17	0.0621628	0.138271173	0.659049887	-0.208848698	0.333174298	1.064135572	0.81151801	1.395390492			
GCST90199935	CAC	outcome	Glycohyocholate levels	Contamination mixture method	17	0.211878457	0.117346939	0.02672115	-0.018121543	0.441878457	1.235997649	0.982041665	1.555626654			
GCST90199935	CAC	outcome	Glycohyocholate levels	Robust adjusted profile score (RAPS)	17	0.165268839	0.068910962	0.016471579	0.030203353	0.300334325	1.179710228	1.030664101	1.350310175			
GCST90199945	CAC	outcome	Sphingomyelin (d18:1/14:0, d16:1/16:0) levels	Inverse variance weighted	26	-0.096896517	0.044515607	0.029503972	-0.184147106	-0.009645928	0.907649928	0.83181343	0.990400445	0.341240068	0.820678728	
GCST90199945	CAC	outcome	Sphingomyelin (d18:1/14:0, d16:1/16:0) levels	MR Egger	26	-0.034210649	0.078418753	0.666550336	-0.187911404	0.119490107	0.966367919	0.828688122	1.126922095		0.824185046	
GCST90199945	CAC	outcome	Sphingomyelin (d18:1/14:0, d16:1/16:0) levels	Weighted median	26	-0.036119785	0.062583215	0.563839405	-0.158782886	0.086543317	0.964524751	0.853181576	1.0903986			
GCST90199945	CAC	outcome	Sphingomyelin (d18:1/14:0, d16:1/16:0) levels	Weighted mode	26	-0.047573816	0.060773116	0.441091244	-0.166689124	0.071541491	0.953540084	0.846462716	1.074162718			
GCST90199945	CAC	outcome	Sphingomyelin (d18:1/14:0, d16:1/16:0) levels	Contamination mixture method	26	-0.09876196	0.084183673	0.074071366	-0.26376196	0.06623804	0.905958336	0.76815637	1.068481028			
GCST90199945	CAC	outcome	Sphingomyelin (d18:1/14:0, d16:1/16:0) levels	Robust adjusted profile score (RAPS)	26	-0.09589265	0.047160548	0.042019852	-0.188327324	-0.003457976	0.908561545	0.828343526	0.996547996			

GCST90200003	CAC	outcome	P-cresol glucuronide levels	Inverse variance weighted	23	-0.135823173	0.061931809	0.028299567	-0.257209518	-0.014436827	0.872996989	0.773206196	0.985666884	0.019902261	0.463566187	
GCST90200003	CAC	outcome	P-cresol glucuronide levels	MR Egger	23	-0.501234718	0.157681282	0.004520509	-0.810290031	-0.192179406	0.605782227	0.444729062	0.825158817		0.792306142	
GCST90200003	CAC	outcome	P-cresol glucuronide levels	Weighted median	23	-0.089688627	0.092922414	0.334445115	-0.271816559	0.092439304	0.914215803	0.76199403	1.096846566			
GCST90200003	CAC	outcome	P-cresol glucuronide levels	Weighted mode	23	-0.060057655	0.140794547	0.673843176	-0.336014968	0.215899657	0.941710237	0.71461241	1.240977849			
GCST90200003	CAC	outcome	P-cresol glucuronide levels	Contamination mixture method	23	-0.248444488	0.137755102	0.042887991	-0.518444488	0.021555512	0.78001316	0.595446052	1.02178951			
GCST90200003	CAC	outcome	P-cresol glucuronide levels	Robust adjusted profile score (RAPS)	23	-0.157206082	0.07126983	0.0273989	-0.296894949	-0.017517215	0.854527938	0.743122074	0.982635319			
GCST90200012	CAC	outcome	3-hydroxyhexanoate levels	Inverse variance weighted	18	-0.208251535	0.077748909	0.007394966	-0.360639396	-0.055863674	0.812002764	0.697230378	0.945668046	0.794259537	0.271285642	
GCST90200012	CAC	outcome	3-hydroxyhexanoate levels	MR Egger	18	-0.257459961	0.202065017	0.220808971	-0.653507395	0.138587473	0.773012577	0.520217963	1.148650154		0.221575192	
GCST90200012	CAC	outcome	3-hydroxyhexanoate levels	Weighted median	18	-0.119174208	0.106277903	0.262140987	-0.327478899	0.089130482	0.887653151	0.7207385	1.093223293			

GCST90200012	CAC	outcome	3-hydroxyhexanoate levels	Weighted mode	18	-0.025130785	0.130379455	0.849437843	-0.280674517	0.230412947	0.975182365	0.755274125	1.259119853			
GCST90200012	CAC	outcome	3-hydroxyhexanoate levels	Contamination mixture method	18	-0.04513865	0.216836735	0.583046149	-0.47013865	0.37986135	0.955864942	0.624915618	1.462081858			
GCST90200012	CAC	outcome	3-hydroxyhexanoate levels	Robust adjusted profile score (RAPS)	18	-0.214853049	0.085443697	0.011918203	-0.382322695	-0.047383402	0.806659971	0.682274851	0.953721668			
GCST90200035	CAC	outcome	Palmitoyl dihydrosphingomyelin (d18:0/16:0) levels	Inverse variance weighted	31	0.159167705	0.055827859	0.004357589	0.049745101	0.268590309	1.17253457	1.051003162	1.308119107	0.47880865	0.333172837	
GCST90200035	CAC	outcome	Palmitoyl dihydrosphingomyelin (d18:0/16:0) levels	MR Egger	31	0.261185873	0.152921543	0.098326478	-0.038540351	0.560912097	1.298468993	0.962192878	1.752270012		0.311772676	
GCST90200035	CAC	outcome	Palmitoyl dihydrosphingomyelin (d18:0/16:0) levels	Weighted median	31	0.062426059	0.078419365	0.426000577	-0.091275896	0.216128013	1.064415752	0.912765848	1.241261267			
GCST90200035	CAC	outcome	Palmitoyl dihydrosphingomyelin (d18:0/16:0) levels	Weighted mode	31	0.058089267	0.108548065	0.596492506	-0.15466494	0.270843475	1.059809598	0.856702176	1.311069839			
GCST90200035	CAC	outcome	Palmitoyl dihydrosphingomyelin (d18:0/16:0) levels	Contamination mixture method	31	0.095630893	0.084183673	0.200102734	-0.069369107	0.260630893	1.100352841	0.932982246	1.297748568			
GCST90200035	CAC	outcome	Palmitoyl dihydrosphingomyelin (d18:0/16:0) levels	Robust adjusted profile score (RAPS)	31	0.134481963	0.057310411	0.01894793	0.022153557	0.246810368	1.143944025	1.02240077	1.279936373			

GCST90200045	CAC	outcome	1-stearoyl-2-arachidonoyl-GPE (18:0/20:4) levels	Inverse variance weighted	29	0.07084297	0.031554305	0.024760899	0.008996533	0.132689407	1.073412655	1.009037123	1.141895279	0.975712047	0.492169498	
GCST90200045	CAC	outcome	1-stearoyl-2-arachidonoyl-GPE (18:0/20:4) levels	MR Egger	29	0.072316963	0.057570269	0.219822039	-0.040520764	0.185154689	1.074996024	0.960289225	1.20340458		0.438122328	
GCST90200045	CAC	outcome	1-stearoyl-2-arachidonoyl-GPE (18:0/20:4) levels	Weighted median	29	0.061868904	0.041336775	0.134470378	-0.019151176	0.142888983	1.063822872	0.981031043	1.153601726			
GCST90200045	CAC	outcome	1-stearoyl-2-arachidonoyl-GPE (18:0/20:4) levels	Weighted mode	29	0.063352642	0.036224237	0.091264378	-0.007646862	0.134352147	1.065402479	0.992382301	1.143795532			
GCST90200045	CAC	outcome	1-stearoyl-2-arachidonoyl-GPE (18:0/20:4) levels	Contamination mixture method	29	0.067782308	0.038265306	0.153733033	-0.007217692	0.142782308	1.070132324	0.992808293	1.153478671			
GCST90200045	CAC	outcome	1-stearoyl-2-arachidonoyl-GPE (18:0/20:4) levels	Robust adjusted profile score (RAPS)	29	0.068109436	0.035154765	0.0526944	-0.000793903	0.137012776	1.070482452	0.999206412	1.1468428			
GCST90200050	CAC	outcome	5-hydroxyindole sulfate levels	Inverse variance weighted	16	0.129420193	0.060752271	0.033147356	0.010345742	0.248494644	1.138168274	1.010399444	1.282093955	0.71588921	0.618091524	
GCST90200050	CAC	outcome	5-hydroxyindole sulfate levels	MR Egger	16	0.191514065	0.177878246	0.299836859	-0.157127297	0.540155428	1.211081867	0.854595264	1.716273598		0.55364946	
GCST90200050	CAC	outcome	5-hydroxyindole sulfate levels	Weighted median	16	0.125107425	0.086002885	0.145755459	-0.043458228	0.293673079	1.133270189	0.957472549	1.341345318			

GCST90200050	CAC	outcome	5-hydroxyindole sulfate levels	Weighted mode	16	0.171908664	0.12407854	0.18616211	-0.071285274	0.415102602	1.187569361	0.931196208	1.514526127			
GCST90200050	CAC	outcome	5-hydroxyindole sulfate levels	Contamination mixture method	16	0.225466358	0.081632653	0.023042201	0.065466358	0.385466358	1.252906883	1.067656819	1.470299848			
GCST90200050	CAC	outcome	5-hydroxyindole sulfate levels	Robust adjusted profile score (RAPS)	16	0.138534592	0.065904701	0.035549408	0.009361377	0.267707806	1.148589412	1.009405332	1.306965197			
GCST90200059	CAC	outcome	Behenoyl dihydrosphingomyelin (d18:0/22:0) levels	Inverse variance weighted	36	0.140167657	0.045028887	0.001852973	0.05191104	0.228424275	1.150466667	1.053282038	1.256618364	0.043393098	0.549209641	
GCST90200059	CAC	outcome	Behenoyl dihydrosphingomyelin (d18:0/22:0) levels	MR Egger	36	0.351939488	0.110519632	0.003099681	0.135321009	0.568557967	1.421822484	1.14490425	1.76571899		0.714869945	
GCST90200059	CAC	outcome	Behenoyl dihydrosphingomyelin (d18:0/22:0) levels	Weighted median	36	0.139620544	0.06404959	0.029266215	0.014083347	0.265157741	1.149837404	1.014182985	1.303636597			
GCST90200059	CAC	outcome	Behenoyl dihydrosphingomyelin (d18:0/22:0) levels	Weighted mode	36	0.219285492	0.111041936	0.056221238	0.001643296	0.436927687	1.245186716	1.001644647	1.547944136			
GCST90200059	CAC	outcome	Behenoyl dihydrosphingomyelin (d18:0/22:0) levels	Contamination mixture method	36	0.221012014	0.066326531	0.003158923	0.091012014	0.351012014	1.247338415	1.095282163	1.420504391			
GCST90200059	CAC	outcome	Behenoyl dihydrosphingomyelin (d18:0/22:0) levels	Robust adjusted profile score (RAPS)	36	0.151949682	0.048652172	0.001789059	0.056591424	0.24730794	1.16410166	1.058223358	1.280573392			

GCST90200071	CAC	outcome	Palmitoylcholine levels	Inverse variance weighted	23	-0.125711233	0.063856497	0.048993044	-0.250869968	-0.000552499	0.881869464	0.778123546	0.999447654	0.665311075	0.995289266	
GCST90200071	CAC	outcome	Palmitoylcholine levels	MR Egger	23	-0.187201988	0.154006732	0.237647712	-0.489055182	0.114651206	0.829276216	0.613205488	1.121482203		0.993316982	
GCST90200071	CAC	outcome	Palmitoylcholine levels	Weighted median	23	-0.143049978	0.088604817	0.106425127	-0.316715419	0.030615462	0.866710751	0.728538054	1.031088935			
GCST90200071	CAC	outcome	Palmitoylcholine levels	Weighted mode	23	-0.200222963	0.144305004	0.179185283	-0.483060771	0.082614846	0.818548227	0.616892333	1.086123403			
GCST90200071	CAC	outcome	Palmitoylcholine levels	Contamination mixture method	23	-0.189273041	0.102040816	0.042369332	-0.389273041	0.010726959	0.827560518	0.677549246	1.010784699			
GCST90200071	CAC	outcome	Palmitoylcholine levels	Robust adjusted profile score (RAPS)	23	-0.128257794	0.069889717	0.066484148	-0.26524164	0.008726052	0.879626588	0.767020585	1.008764235			
GCST90200119	CAC	outcome	Glycosyl ceramide (d18:1/23:1, d17:1/24:1) levels	Inverse variance weighted	20	-0.118130648	0.052335482	0.023996774	-0.220708192	-0.015553105	0.888579953	0.801950662	0.98456722	0.169538484	0.618178382	
GCST90200119	CAC	outcome	Glycosyl ceramide (d18:1/23:1, d17:1/24:1) levels	MR Egger	20	-0.364152054	0.17970202	0.057801209	-0.716368014	-0.011936094	0.694785542	0.488523348	0.988134859		0.693692077	
GCST90200119	CAC	outcome	Glycosyl ceramide (d18:1/23:1, d17:1/24:1) levels	Weighted median	20	-0.093177298	0.074923059	0.213632164	-0.240026494	0.053671898	0.911031962	0.78660702	1.055138353			

GCST90200119	CAC	outcome	Glycosyl ceramide (d18:1/23:1, d17:1/24:1) levels	Weighted mode	20	-0.096959258	0.095335749	0.321916092	-0.283817326	0.089898811	0.907592983	0.752904168	1.09406357			
GCST90200119	CAC	outcome	Glycosyl ceramide (d18:1/23:1, d17:1/24:1) levels	Contamination mixture method	20	-0.100504817	0.081632653	0.14622989	-0.260504817	0.059495183	0.904380756	0.770662444	1.061300648			
GCST90200119	CAC	outcome	Glycosyl ceramide (d18:1/23:1, d17:1/24:1) levels	Robust adjusted profile score (RAPS)	20	-0.118894231	0.05643085	0.035126136	-0.229498697	-0.008289765	0.887901708	0.794932005	0.9917445			
GCST90200130	CAC	outcome	Linoleoylcholine levels	Inverse variance weighted	19	-0.231133965	0.067286191	0.000592362	-0.3630149	-0.099253031	0.793633139	0.695576069	0.905513556	0.651434036	0.989956598	
GCST90200130	CAC	outcome	Linoleoylcholine levels	MR Egger	19	-0.307815611	0.179809271	0.105095475	-0.660241782	0.044610561	0.735050841	0.516726384	1.045620575		0.985895516	
GCST90200130	CAC	outcome	Linoleoylcholine levels	Weighted median	19	-0.231606987	0.092458407	0.012245634	-0.412825464	-0.05038851	0.793257822	0.661777777	0.950859934			
GCST90200130	CAC	outcome	Linoleoylcholine levels	Weighted mode	19	-0.245559636	0.140055571	0.096563232	-0.520068556	0.028949284	0.782266631	0.594479792	1.029372387			
GCST90200130	CAC	outcome	Linoleoylcholine levels	Contamination mixture method	19	-0.310073634	0.089285714	0.001342204	-0.485073634	-0.135073634	0.733392952	0.615651862	0.873651579			
GCST90200130	CAC	outcome	Linoleoylcholine levels	Robust adjusted profile score (RAPS)	19	-0.235353409	0.074554178	0.00159505	-0.381479597	-0.089227221	0.790291504	0.682850318	0.914637725			

GCST90200145	CAC	outcome	Glycosyl ceramide (d18:1/20:0, d16:1/22:0) levels	Inverse variance weighted (multiplicative random effects)	30	-0.1202976	0.052515573	0.021980576	-0.223228124	-0.017367076	0.886656528	0.799932345	0.982782862	0.067849914	0.016057869	
GCST90200145	CAC	outcome	Glycosyl ceramide (d18:1/20:0, d16:1/22:0) levels	MR Egger	30	-0.328052166	0.120388197	0.010958583	-0.564013033	-0.092091299	0.720325442	0.568921377	0.912021879		0.041591185	
GCST90200145	CAC	outcome	Glycosyl ceramide (d18:1/20:0, d16:1/22:0) levels	Weighted median	30	-0.090137345	0.065164971	0.166598105	-0.217860688	0.037585998	0.91380567	0.804237474	1.038301285			
GCST90200145	CAC	outcome	Glycosyl ceramide (d18:1/20:0, d16:1/22:0) levels	Weighted mode	30	-0.128891686	0.077308041	0.106230372	-0.280415446	0.022632073	0.879069176	0.755469819	1.022890122			
GCST90200145	CAC	outcome	Glycosyl ceramide (d18:1/20:0, d16:1/22:0) levels	Contamination mixture method	30	-0.200705799	0.068877551	0.001728767	-0.335705799	-0.065705799	0.818153098	0.714833379	0.936406315			
GCST90200145	CAC	outcome	Glycosyl ceramide (d18:1/20:0, d16:1/22:0) levels	Robust adjusted profile score (RAPS)	30	-0.147609762	0.056352416	0.008808348	-0.258060498	-0.037159027	0.862767734	0.772548493	0.963522897			
GCST90200162	CAC	outcome	3-hydroxyphenylacetoylglutamine levels	Inverse variance weighted (multiplicative random effects)	16	0.194389684	0.087505555	0.026320136	0.022878795	0.365900573	1.214569489	1.023142522	1.44181188	0.651185582	0.038476489	
GCST90200162	CAC	outcome	3-hydroxyphenylacetoylglutamine levels	MR Egger	16	0.271786214	0.190122934	0.174786918	-0.100854737	0.644427164	1.312306418	0.904064351	1.904895524		0.029363096	

GCST90200162	CAC	outcome	3-hydroxyphenylacetoylglutamine levels	Weighted median	16	0.113126897	0.103116906	0.272608955	-0.088982238	0.315236033	1.11977402	0.914861823	1.370582775			
GCST90200162	CAC	outcome	3-hydroxyphenylacetoylglutamine levels	Weighted mode	16	0.10383332	0.149721071	0.498587703	-0.189619979	0.39728662	1.109415522	0.827273455	1.487782297			
GCST90200162	CAC	outcome	3-hydroxyphenylacetoylglutamine levels	Contamination mixture method	16	0.186897393	0.119897959	0.062426696	-0.048102607	0.421897393	1.205503586	0.953035994	1.524852056			
GCST90200162	CAC	outcome	3-hydroxyphenylacetoylglutamine levels	Robust adjusted profile score (RAPS)	16	0.195813694	0.105370993	0.063122683	-0.010713452	0.40234084	1.21630028	0.989343732	1.495320912			
GCST90200213	CAC	outcome	2-naphthol sulfate levels	Inverse variance weighted	21	0.132166646	0.067077126	0.048796168	0.00069548	0.263637812	1.141298497	1.000695722	1.301656667	0.89671218	0.669712581	
GCST90200213	CAC	outcome	2-naphthol sulfate levels	MR Egger	21	0.149095261	0.145106171	0.317093364	-0.135312834	0.433503356	1.160783561	0.873442626	1.542652529		0.608571161	
GCST90200213	CAC	outcome	2-naphthol sulfate levels	Weighted median	21	0.168499186	0.095471706	0.077578509	-0.018625357	0.355623729	1.183527263	0.981547023	1.427070482			
GCST90200213	CAC	outcome	2-naphthol sulfate levels	Weighted mode	21	0.300894534	0.164949931	0.083106747	-0.02240733	0.624196398	1.351066842	0.977841849	1.866745235			
GCST90200213	CAC	outcome	2-naphthol sulfate levels	Contamination mixture method	21	0.237240574	0.112244898	0.011305428	0.017240574	0.457240574	1.267746068	1.01739005	1.579708875			

GCST90200213	CAC	outcome	2-naphthol sulfate levels	Robust adjusted profile score (RAPS)	21	0.152148186	0.073086316	0.037364376	0.008899007	0.295397366	1.164332762	1.008938721	1.343660177			
GCST90200228	CAC	outcome	3-hydroxyhexanoylcarnitine (1) levels	Inverse variance weighted	18	-0.141117252	0.061326815	0.021387715	-0.26131781	-0.020916694	0.868387486	0.770036156	0.979300543	0.115302292	0.849692891	
GCST90200228	CAC	outcome	3-hydroxyhexanoylcarnitine (1) levels	MR Egger	18	-0.425463091	0.181423778	0.032244562	-0.781053696	-0.069872486	0.65346671	0.457923245	0.932512721		0.937510515	
GCST90200228	CAC	outcome	3-hydroxyhexanoylcarnitine (1) levels	Weighted median	18	-0.234207869	0.087186561	0.007225164	-0.405093529	-0.063322209	0.791197333	0.666914432	0.938640986			
GCST90200228	CAC	outcome	3-hydroxyhexanoylcarnitine (1) levels	Weighted mode	18	-0.217675117	0.108912634	0.061900634	-0.431143879	-0.004206354	0.804386731	0.649765417	0.99580248			
GCST90200228	CAC	outcome	3-hydroxyhexanoylcarnitine (1) levels	Contamination mixture method	18	-0.177909011	0.086734694	0.013604962	-0.347909011	-0.007909011	0.837018579	0.706163126	0.992122183			
GCST90200228	CAC	outcome	3-hydroxyhexanoylcarnitine (1) levels	Robust adjusted profile score (RAPS)	18	-0.151723448	0.066096137	0.02170469	-0.281271877	-0.022175019	0.859225868	0.754823089	0.978069039			
GCST90200278	CAC	outcome	3-hydroxyoctanoylcarnitine (2) levels	Inverse variance weighted	18	-0.185645513	0.067171783	0.005714261	-0.317302207	-0.053988819	0.830567968	0.728110682	0.9474427	0.482925906	0.569114301	
GCST90200278	CAC	outcome	3-hydroxyoctanoylcarnitine (2) levels	MR Egger	18	-0.323767845	0.203683455	0.131495526	-0.722987416	0.075451727	0.723418168	0.485300294	1.07837117		0.535625033	

GCST90200278	CAC	outcome	3-hydroxyoctanoylcarnitine (2) levels	Weighted median	18	-0.118038097	0.095107642	0.214568799	-0.304449074	0.068372881	0.888662197	0.737529587	1.070764502			
GCST90200278	CAC	outcome	3-hydroxyoctanoylcarnitine (2) levels	Weighted mode	18	-0.093286932	0.143970183	0.525666215	-0.37546849	0.188894627	0.910932088	0.686967366	1.207913664			
GCST90200278	CAC	outcome	3-hydroxyoctanoylcarnitine (2) levels	Contamination mixture method	18	-0.288390171	0.132653061	0.030504001	-0.548390171	-0.028390171	0.749469114	0.577879349	0.972009043			
GCST90200278	CAC	outcome	3-hydroxyoctanoylcarnitine (2) levels	Robust adjusted profile score (RAPS)	18	-0.198028442	0.075059116	0.008332291	-0.34514431	-0.050912574	0.82034652	0.708118157	0.950361753			
GCST90200305	CAC	outcome	2-aminobutyrate levels	Inverse variance weighted	19	-0.161536115	0.06514975	0.013158196	-0.289229626	-0.033842605	0.850835803	0.748840233	0.96672365	0.041624079	0.332908166	
GCST90200305	CAC	outcome	2-aminobutyrate levels	MR Egger	19	-0.495885838	0.163831644	0.007607033	-0.816995859	-0.174775816	0.609031165	0.441756763	0.839645235		0.585230723	
GCST90200305	CAC	outcome	2-aminobutyrate levels	Weighted median	19	-0.194027365	0.089783536	0.030690915	-0.370003096	-0.018051633	0.823635365	0.690732192	0.982110321			
GCST90200305	CAC	outcome	2-aminobutyrate levels	Weighted mode	19	-0.234117095	0.109918184	0.047231268	-0.449556736	-0.018677454	0.791269157	0.637910852	0.981495889			
GCST90200305	CAC	outcome	2-aminobutyrate levels	Contamination mixture method	19	-0.283030475	0.086734694	0.013864078	-0.453030475	-0.113030475	0.753496824	0.63569876	0.893123441			

GCST90200305	CAC	outcome	2-aminobutyrate levels	Robust adjusted profile score (RAPS)	19	-0.176430112	0.073273327	0.016047414	-0.320045833	-0.03281439	0.838257361	0.726115756	0.967718161			
GCST90200311	CAC	outcome	4-acetamidobutanoate levels	Inverse variance weighted	18	0.133889469	0.050658462	0.008217909	0.034598884	0.233180054	1.143266446	1.035204388	1.262608797	0.183740727	0.513082704	
GCST90200311	CAC	outcome	4-acetamidobutanoate levels	MR Egger	18	0.028750488	0.091062881	0.756295037	-0.149732759	0.207233735	1.029167773	0.860938024	1.230270095		0.582210795	
GCST90200311	CAC	outcome	4-acetamidobutanoate levels	Weighted median	18	0.107094317	0.065305437	0.10102618	-0.020904339	0.235092972	1.113039228	0.979312642	1.265026376			
GCST90200311	CAC	outcome	4-acetamidobutanoate levels	Weighted mode	18	0.114198465	0.074313529	0.142766901	-0.031456052	0.259852983	1.120974577	0.969033542	1.296739429			
GCST90200311	CAC	outcome	4-acetamidobutanoate levels	Contamination mixture method	18	0.169591449	0.117346939	0.025320166	-0.060408551	0.399591449	1.184820692	0.941379853	1.491215335			
GCST90200311	CAC	outcome	4-acetamidobutanoate levels	Robust adjusted profile score (RAPS)	18	0.138731746	0.053599304	0.009644746	0.03367711	0.243786383	1.148815884	1.034250603	1.276071711			
GCST90200341	CAC	outcome	N-stearoyl-sphingosine (d18:1/18:0) levels	Inverse variance weighted	23	-0.13013481	0.057391219	0.023359464	-0.242621599	-0.017648021	0.877977063	0.784568339	0.982506794	0.897808694	0.306422074	
GCST90200341	CAC	outcome	N-stearoyl-sphingosine (d18:1/18:0) levels	MR Egger	23	-0.151685058	0.175871079	0.398166856	-0.496392372	0.193022257	0.859258855	0.608722749	1.212909788		0.256531139	

GCST90200341	CAC	outcome	N-stearoyl-sphingosine (d18:1/18:0) levels	Weighted median	23	-0.17684758	0.077918626	0.023229162	-0.329568087	-0.024127073	0.837907489	0.719234313	0.976161658			
GCST90200341	CAC	outcome	N-stearoyl-sphingosine (d18:1/18:0) levels	Weighted mode	23	-0.19500784	0.099662599	0.063195944	-0.390346535	0.000330854	0.822828207	0.676822291	1.000330909			
GCST90200341	CAC	outcome	N-stearoyl-sphingosine (d18:1/18:0) levels	Contamination mixture method	23	-0.219117501	0.073979592	0.008280751	-0.364117501	-0.074117501	0.803227333	0.694809549	0.928562581			
GCST90200341	CAC	outcome	N-stearoyl-sphingosine (d18:1/18:0) levels	Robust adjusted profile score (RAPS)	23	-0.141538266	0.059011211	0.016462471	-0.25720024	-0.025876292	0.86802196	0.77321337	0.974455631			
GCST90200371	CAC	outcome	3-(4-hydroxyphenyl)lactate levels	Inverse variance weighted	27	-0.14795607	0.056831683	0.009230198	-0.259346168	-0.036565972	0.862469002	0.771555889	0.964094489	0.372796347	0.494529352	
GCST90200371	CAC	outcome	3-(4-hydroxyphenyl)lactate levels	MR Egger	27	-0.013344428	0.158843628	0.933717717	-0.324677939	0.297989084	0.986744214	0.722760089	1.347147082		0.484369662	
GCST90200371	CAC	outcome	3-(4-hydroxyphenyl)lactate levels	Weighted median	27	-0.055082869	0.084962947	0.516780349	-0.221610246	0.111444507	0.946406717	0.801227585	1.117891707			
GCST90200371	CAC	outcome	3-(4-hydroxyphenyl)lactate levels	Weighted mode	27	-0.025591933	0.128353744	0.843511984	-0.277165271	0.225981405	0.974732765	0.757929223	1.253552355			
GCST90200371	CAC	outcome	3-(4-hydroxyphenyl)lactate levels	Contamination mixture method	27	-0.204508914	0.165816327	0.283072323	-0.529508914	0.120491086	0.815047477	0.588894097	1.128050686			

GCST90200371	CAC	outcome	3-(4-hydroxyphenyl)lactate levels	Robust adjusted profile score (RAPS)	27	-0.151405738	0.062260958	0.015024307	-0.273437215	-0.029374261	0.859498897	0.760760099	0.97105297			
GCST90200376	CAC	outcome	Flavin adenine dinucleotide (FAD) levels	Inverse variance weighted	23	0.109810612	0.039248072	0.00514424	0.032884392	0.186736833	1.116066681	1.033431059	1.205310046	0.636261922	0.848767188	
GCST90200376	CAC	outcome	Flavin adenine dinucleotide (FAD) levels	MR Egger	23	0.137218533	0.069298022	0.060943269	0.00139441	0.273042655	1.147078796	1.001395383	1.313956291		0.819125912	
GCST90200376	CAC	outcome	Flavin adenine dinucleotide (FAD) levels	Weighted median	23	0.118260794	0.055949629	0.034540803	0.008599521	0.227922068	1.125537607	1.008636603	1.25598744			
GCST90200376	CAC	outcome	Flavin adenine dinucleotide (FAD) levels	Weighted mode	23	0.115283182	0.057841622	0.058799632	0.001913603	0.228652762	1.122191177	1.001915435	1.256905517			
GCST90200376	CAC	outcome	Flavin adenine dinucleotide (FAD) levels	Contamination mixture method	23	0.142121163	0.06122449	0.008672278	0.022121163	0.262121163	1.152716307	1.02236765	1.299684007			
GCST90200376	CAC	outcome	Flavin adenine dinucleotide (FAD) levels	Robust adjusted profile score (RAPS)	23	0.112921367	0.041655812	0.006711724	0.031275977	0.194566758	1.119543897	1.031770209	1.214784577			
GCST90200399	CAC	outcome	Isoleucine levels	Inverse variance weighted	21	0.249610324	0.067981104	0.000240885	0.11636736	0.382853289	1.283525161	1.123408492	1.466462867	0.591441291	0.9939024	
GCST90200399	CAC	outcome	Isoleucine levels	MR Egger	21	0.155982932	0.184471697	0.408317953	-0.205581594	0.517547457	1.168806253	0.81417366	1.67790746		0.992037134	

GCST90200399	CAC	outcome	Isoleucine levels	Weighted median	21	0.280521794	0.094020131	0.002848459	0.096242338	0.464801251	1.323820394	1.101025852	1.591697808			
GCST90200399	CAC	outcome	Isoleucine levels	Weighted mode	21	0.306382042	0.163053209	0.074893494	-0.013202248	0.625966331	1.358501212	0.986884519	1.870052175			
GCST90200399	CAC	outcome	Isoleucine levels	Contamination mixture method	21	0.287472837	0.094387755	0.00064897	0.102472837	0.472472837	1.333054382	1.107907207	1.603955614			
GCST90200399	CAC	outcome	Isoleucine levels	Robust adjusted profile score (RAPS)	21	0.253341802	0.075617287	0.000807194	0.105131919	0.401551684	1.288323553	1.110857144	1.494141335			
GCST90200409	CAC	outcome	Pristanate levels	Inverse variance weighted	14	0.189852535	0.074919925	0.011274534	0.043009482	0.336695588	1.209071289	1.043947794	1.400312726	0.778539572	0.707795787	
GCST90200409	CAC	outcome	Pristanate levels	MR Egger	14	0.238585448	0.185256437	0.222074266	-0.12451717	0.601688065	1.269452173	0.882923102	1.825197253		0.638145889	
GCST90200409	CAC	outcome	Pristanate levels	Weighted median	14	0.136588597	0.098999561	0.167682445	-0.057450542	0.330627737	1.146356438	0.944168586	1.391841564			
GCST90200409	CAC	outcome	Pristanate levels	Weighted mode	14	0.104329341	0.150876082	0.501420559	-0.19138778	0.400046462	1.109965952	0.825812293	1.491894012			
GCST90200409	CAC	outcome	Pristanate levels	Contamination mixture method	14	0.213945901	0.096938776	0.020026255	0.023945901	0.403945901	1.238555649	1.024234906	1.49772292			
GCST90200409	CAC	outcome	Pristanate levels	Robust adjusted profile score (RAPS)	14	0.199835774	0.08270489	0.015681247	0.037734189	0.361937358	1.221202188	1.038455164	1.436108979			

GCST90200489	CAC	outcome	X-12407 levels	Inverse variance weighted	23	0.150112856	0.059467091	0.011592851	0.033557358	0.266668354	1.16196537	1.034126758	1.305607375	0.466555858	0.920497686	
GCST90200489	CAC	outcome	X-12407 levels	MR Egger	23	0.045294324	0.153345306	0.770608347	-0.255262475	0.345851124	1.046335777	0.774713129	1.413192209		0.912796212	
GCST90200489	CAC	outcome	X-12407 levels	Weighted median	23	0.162737202	0.08323608	0.050567948	-0.000405515	0.325879919	1.176727407	0.999594567	1.385249016			
GCST90200489	CAC	outcome	X-12407 levels	Weighted mode	23	0.218591176	0.157935609	0.180223618	-0.090962618	0.528144969	1.244322463	0.913051842	1.695783657			
GCST90200489	CAC	outcome	X-12407 levels	Contamination mixture method	23	0.231480125	0.091836735	0.008980566	0.051480125	0.411480125	1.260464275	1.052828261	1.509049715			
GCST90200489	CAC	outcome	X-12407 levels	Robust adjusted profile score (RAPS)	23	0.162474165	0.065445391	0.01304306	0.034201199	0.29074713	1.176417925	1.034792785	1.337426347			
GCST90200515	CAC	outcome	X-12849 levels	Inverse variance weighted	22	-0.206612197	0.072358341	0.00429823	-0.348434544	-0.064789849	0.813335003	0.705792112	0.93726441	0.579672458	0.057515555	
GCST90200515	CAC	outcome	X-12849 levels	MR Egger	22	-0.304779037	0.189238681	0.122947573	-0.675686851	0.066128777	0.737286269	0.508806826	1.068364289		0.048032061	
GCST90200515	CAC	outcome	X-12849 levels	Weighted median	22	-0.281465965	0.08676069	0.001177956	-0.451516917	-0.111415012	0.754676601	0.636661656	0.894567415			

GCST90200515	CAC	outcome	X-12849 levels	Weighted mode	22	-0.38760989	0.166380401	0.029882276	-0.713715476	-0.061504305	0.67867705	0.489820895	0.940348898			
GCST90200515	CAC	outcome	X-12849 levels	Contamination mixture method	22	-0.424732025	0.084183673	0.000324998	-0.589732025	-0.259732025	0.653945002	0.55447585	0.771258236			
GCST90200515	CAC	outcome	X-12849 levels	Robust adjusted profile score (RAPS)	22	-0.252411619	0.075737157	0.000859978	-0.400856446	-0.103966792	0.776924875	0.669746199	0.901255226			
GCST90200531	CAC	outcome	X-16964 levels	Inverse variance weighted	16	-0.136490285	0.069140948	0.048371789	-0.272006543	-0.000974026	0.872414796	0.761849276	0.999026448	0.510786488	0.436454623	
GCST90200531	CAC	outcome	X-16964 levels	MR Egger	16	-0.033056746	0.168684944	0.847451668	-0.363679237	0.297565744	0.967483657	0.695114126	1.346576901		0.396850055	
GCST90200531	CAC	outcome	X-16964 levels	Weighted median	16	-0.220558397	0.096338694	0.022055767	-0.409382238	-0.031734556	0.802070799	0.664060354	0.9687637			
GCST90200531	CAC	outcome	X-16964 levels	Weighted mode	16	-0.283251383	0.148374446	0.075577917	-0.574065298	0.007562531	0.753330389	0.563231076	1.007591199			
GCST90200531	CAC	outcome	X-16964 levels	Contamination mixture method	16	-0.25364125	0.079081633	0.004185885	-0.40864125	-0.09864125	0.775970132	0.664552598	0.906067702			
GCST90200531	CAC	outcome	X-16964 levels	Robust adjusted profile score (RAPS)	16	-0.174447448	0.074918195	0.019885264	-0.32128711	-0.027607786	0.839920993	0.725215004	0.972769826			

GCST90200570	CAC	outcome	X-18921 levels	Inverse variance weighted	16	-0.087040111	0.040906014	0.033353071	-0.167215897	-0.006864324	0.916640328	0.846016939	0.993159182	0.388023172	0.909268032	
GCST90200570	CAC	outcome	X-18921 levels	MR Egger	16	-0.135695315	0.068233106	0.066645329	-0.269432203	-0.001958427	0.873108615	0.763813062	0.99804349		0.91143464	
GCST90200570	CAC	outcome	X-18921 levels	Weighted median	16	-0.121493624	0.049158277	0.013455564	-0.217843847	-0.025143402	0.88559667	0.804251018	0.975170061			
GCST90200570	CAC	outcome	X-18921 levels	Weighted mode	16	-0.124588851	0.051717969	0.029304343	-0.225956069	-0.023221632	0.882859815	0.797753147	0.977045915			
GCST90200570	CAC	outcome	X-18921 levels	Contamination mixture method	16	-0.08370869	0.079081633	0.149246718	-0.23870869	0.07129131	0.919699134	0.787644297	1.073894016			
GCST90200570	CAC	outcome	X-18921 levels	Robust adjusted profile score (RAPS)	16	-0.08764561	0.042889749	0.041002027	-0.171709517	-0.003581702	0.916085471	0.842223789	0.996424705			
GCST90200585	CAC	outcome	X-21829 levels	Inverse variance weighted	27	0.134743174	0.053524099	0.011821445	0.02983594	0.239650408	1.144242875	1.030285491	1.270804809	0.211888181	0.961657653	
GCST90200585	CAC	outcome	X-21829 levels	MR Egger	27	0.297535911	0.137877858	0.040733047	0.027295309	0.567776512	1.346536729	1.027671238	1.764339699		0.975083174	
GCST90200585	CAC	outcome	X-21829 levels	Weighted median	27	0.11007104	0.074871005	0.141523186	-0.036676129	0.25681821	1.116357374	0.963988292	1.292810085			

GCST90200585	CAC	outcome	X-21829 levels	Weighted mode	27	0.112498805	0.108875186	0.310987864	-0.100896561	0.32589417	1.119070919	0.90402654	1.385268758			
GCST90200585	CAC	outcome	X-21829 levels	Contamination mixture method	27	0.165301416	0.081632653	0.091161992	0.005301416	0.325301416	1.17974866	1.005315493	1.384447877			
GCST90200585	CAC	outcome	X-21829 levels	Robust adjusted profile score (RAPS)	27	0.125576398	0.057662231	0.029421458	0.012558425	0.238594371	1.133801786	1.012637613	1.269463501			
GCST90200588	CAC	outcome	X-21742 levels	Inverse variance weighted	19	-0.149293159	0.068856154	0.030144306	-0.284251222	-0.014335097	0.861316575	0.752577557	0.985767161	0.645094823	0.155708704	
GCST90200588	CAC	outcome	X-21742 levels	MR Egger	19	-0.212160126	0.15143069	0.179193301	-0.508964278	0.084644026	0.808835171	0.601117847	1.08832958		0.128593545	
GCST90200588	CAC	outcome	X-21742 levels	Weighted median	19	-0.111251624	0.085688311	0.194174287	-0.279200713	0.056697466	0.894713589	0.756388071	1.058335579			
GCST90200588	CAC	outcome	X-21742 levels	Weighted mode	19	-0.031141497	0.15430153	0.842318968	-0.333572496	0.271289501	0.969338404	0.716359963	1.311654741			
GCST90200588	CAC	outcome	X-21742 levels	Contamination mixture method	19	-0.156398197	0.135204082	0.036888257	-0.421398197	0.108601803	0.855218577	0.656128781	1.114718384			
GCST90200588	CAC	outcome	X-21742 levels	Robust adjusted profile score (RAPS)	19	-0.167420439	0.06875745	0.014894155	-0.302185042	-0.032655837	0.845843911	0.739201269	0.967871608			

GCST90200591	CAC	outcome	X-21383 levels	Inverse variance weighted	21	-0.228855397	0.060749605	0.000165094	-0.347924622	-0.109786172	0.795443549	0.706152102	0.89602571	0.282278157	0.917567733	
GCST90200591	CAC	outcome	X-21383 levels	MR Egger	21	-0.067229447	0.158185572	0.675608394	-0.377273168	0.242814274	0.934980648	0.685728729	1.274831832		0.932529337	
GCST90200591	CAC	outcome	X-21383 levels	Weighted median	21	-0.238068118	0.080320546	0.003036926	-0.395496388	-0.080639848	0.788149002	0.673345716	0.922525881			
GCST90200591	CAC	outcome	X-21383 levels	Weighted mode	21	-0.264782302	0.138727181	0.070762921	-0.536687577	0.007122972	0.767372987	0.584681762	1.007148401			
GCST90200591	CAC	outcome	X-21383 levels	Contamination mixture method	21	-0.291541737	0.079081633	0.000530091	-0.446541737	-0.136541737	0.747110831	0.639837055	0.87236991			
GCST90200591	CAC	outcome	X-21383 levels	Robust adjusted profile score (RAPS)	21	-0.237843711	0.067194535	0.00040069	-0.369545	-0.106142421	0.788325888	0.691048686	0.89929656			
GCST90200647	CAC	outcome	X-24736 levels	Inverse variance weighted	29	-0.082728178	0.039027658	0.034028645	-0.159222387	-0.006233969	0.920601353	0.852806685	0.993785422	0.481120386	0.187429687	
GCST90200647	CAC	outcome	X-24736 levels	MR Egger	29	-0.142493682	0.092462317	0.134932163	-0.323719824	0.03873246	0.867193033	0.723452908	1.03949234		0.172533616	
GCST90200647	CAC	outcome	X-24736 levels	Weighted median	29	-0.104067415	0.054949029	0.058239615	-0.211767512	0.003632681	0.901164543	0.809152794	1.003639287			

GCST90200647	CAC	outcome	X-24736 levels	Weighted mode	29	-0.115159336	0.059848137	0.064548742	-0.232461685	0.002143012	0.891224128	0.792580117	1.00214531			
GCST90200647	CAC	outcome	X-24736 levels	Contamination mixture method	29	-0.135616311	0.045918367	0.006097401	-0.225616311	-0.045616311	0.873177597	0.798024236	0.955408471			
GCST90200647	CAC	outcome	X-24736 levels	Robust adjusted profile score (RAPS)	29	-0.082007312	0.047172376	0.082129702	-0.174465168	0.010450544	0.921265222	0.839906109	1.010505342			
GCST90200669	CAC	outcome	X-25519 levels	Inverse variance weighted	19	-0.188454296	0.066681569	0.004710639	-0.319150171	-0.057758421	0.828238356	0.726766402	0.943877941	0.50814897	0.584224069	
GCST90200669	CAC	outcome	X-25519 levels	MR Egger	19	-0.283780917	0.155991435	0.086538179	-0.589524129	0.021962295	0.752931581	0.554591136	1.022205241		0.54788661	
GCST90200669	CAC	outcome	X-25519 levels	Weighted median	19	-0.214066994	0.087578222	0.014513561	-0.385720309	-0.042413678	0.807294299	0.679960678	0.958473199			
GCST90200669	CAC	outcome	X-25519 levels	Weighted mode	19	-0.249406397	0.158942942	0.134023106	-0.560934563	0.062121769	0.779263219	0.570675483	1.06409191			
GCST90200669	CAC	outcome	X-25519 levels	Contamination mixture method	19	-0.312133376	0.091836735	0.009778907	-0.492133376	-0.132133376	0.731883906	0.611320825	0.87622412			
GCST90200669	CAC	outcome	X-25519 levels	Robust adjusted profile score (RAPS)	19	-0.203821279	0.072858482	0.005150048	-0.346623903	-0.061018654	0.815608125	0.707071205	0.94080569			

GCST90200704	CAC	outcome	Bilirubin (z,z) levels	Inverse variance weighted	28	-0.062319648	0.02820369	0.02713103	-0.117598881	-0.007040415	0.939582503	0.889052597	0.992984311	0.202240173	0.494962508	
GCST90200704	CAC	outcome	Bilirubin (z,z) levels	MR Egger	28	-0.026718652	0.039191708	0.501430072	-0.1035344	0.050097095	0.973635133	0.901645006	1.051373175		0.535070227	
GCST90200704	CAC	outcome	Bilirubin (z,z) levels	Weighted median	28	-0.03910514	0.031714058	0.217555687	-0.101264693	0.023054413	0.961649596	0.903693799	1.02332222			
GCST90200704	CAC	outcome	Bilirubin (z,z) levels	Weighted mode	28	-0.040237298	0.03217709	0.221847872	-0.103304395	0.022829799	0.960561473	0.901852413	1.023092394			
GCST90200704	CAC	outcome	Bilirubin (z,z) levels	Contamination mixture method	28	-0.050641497	0.025510204	0.093677443	-0.100641497	-0.000641497	0.95061941	0.904257154	0.999358709			
GCST90200704	CAC	outcome	Bilirubin (z,z) levels	Robust adjusted profile score (RAPS)	28	-0.055239857	0.029218228	0.058678336	-0.112507584	0.002027871	0.946258154	0.89359057	1.002029929			
GCST90200715	CAC	outcome	Alpha-ketoglutarate to glutamate ratio	Inverse variance weighted	30	-0.124421097	0.063377562	0.049626114	-0.248641119	-0.000201076	0.88300793	0.7798598	0.999798944	0.316437712	0.343863364	
GCST90200715	CAC	outcome	Alpha-ketoglutarate to glutamate ratio	MR Egger	30	-0.306911091	0.189782418	0.117053073	-0.678884631	0.065062448	0.73571601	0.507182373	1.067225668		0.347286451	
GCST90200715	CAC	outcome	Alpha-ketoglutarate to glutamate ratio	Weighted median	30	-0.110893931	0.08770441	0.206084549	-0.282794575	0.061006712	0.895033679	0.753674595	1.062906049			

GCST90200715	CAC	outcome	Alpha-ketoglutarate to glutamate ratio	Weighted mode	30	-0.144177424	0.154641739	0.358862756	-0.447275232	0.158920384	0.865734133	0.63936791	1.172244614			
GCST90200715	CAC	outcome	Alpha-ketoglutarate to glutamate ratio	Contamination mixture method	30	-0.165029688	0.089285714	0.036391685	-0.340029688	0.009970312	0.847868533	0.711749192	1.010020182			
GCST90200715	CAC	outcome	Alpha-ketoglutarate to glutamate ratio	Robust adjusted profile score (RAPS)	30	-0.13362433	0.066903366	0.045795465	-0.264754927	-0.002493732	0.874918684	0.767393995	0.997509374			
GCST90200737	CAC	outcome	Adenosine 5'-diphosphate (ADP) to 2'-deoxyuridine ratio	Inverse variance weighted	21	0.101953786	0.04985601	0.040858328	0.004236006	0.199671565	1.107332296	1.004244991	1.221001673	0.639185931	0.948532643	
GCST90200737	CAC	outcome	Adenosine 5'-diphosphate (ADP) to 2'-deoxyuridine ratio	MR Egger	21	0.157197506	0.126212541	0.228090137	-0.090179074	0.404574086	1.170226718	0.913767539	1.498664062		0.934053359	
GCST90200737	CAC	outcome	Adenosine 5'-diphosphate (ADP) to 2'-deoxyuridine ratio	Weighted median	21	0.112289897	0.068275611	0.100040761	-0.021530301	0.246110094	1.118837161	0.978699822	1.279040381			
GCST90200737	CAC	outcome	Adenosine 5'-diphosphate (ADP) to 2'-deoxyuridine ratio	Weighted mode	21	0.108517119	0.12357982	0.390309529	-0.133699328	0.350733566	1.11462399	0.874853069	1.42010891			
GCST90200737	CAC	outcome	Adenosine 5'-diphosphate (ADP) to 2'-deoxyuridine ratio	Contamination mixture method	21	0.168646345	0.076530612	0.028252346	0.018646345	0.318646345	1.183701443	1.018821274	1.37526487			
GCST90200737	CAC	outcome	Adenosine 5'-diphosphate (ADP) to 2'-deoxyuridine ratio	Robust adjusted profile score (RAPS)	21	0.106224128	0.054667575	0.052005041	-0.000924318	0.213372574	1.112071095	0.999076109	1.237845755			

GCST90200846	CAC	outcome	Phenylalanine to phosphate ratio	Inverse variance weighted (multiplicative random effects)	20	-0.277103979	0.081243481	0.000647763	-0.436341201	-0.117866757	0.75797568	0.646397137	0.888814473	0.974662412	0.041582523	
GCST90200846	CAC	outcome	Phenylalanine to phosphate ratio	MR Egger	20	-0.283987442	0.229453803	0.231741537	-0.733716896	0.165742013	0.752776098	0.480121109	1.180268568		0.029711158	
GCST90200846	CAC	outcome	Phenylalanine to phosphate ratio	Weighted median	20	-0.270214325	0.097262346	0.005466083	-0.460848524	-0.079580126	0.763215901	0.630748213	0.923504021			
GCST90200846	CAC	outcome	Phenylalanine to phosphate ratio	Weighted mode	20	-0.216265653	0.158574563	0.188567805	-0.527071797	0.094540492	0.805521285	0.590331051	1.099153669			
GCST90200846	CAC	outcome	Phenylalanine to phosphate ratio	Contamination mixture method	20	-0.201818375	0.160714286	0.042589689	-0.516818375	0.113181625	0.817243347	0.596415102	1.119835305			
GCST90200846	CAC	outcome	Phenylalanine to phosphate ratio	Robust adjusted profile score (RAPS)	20	-0.261753854	0.119906456	0.029037001	-0.496770508	-0.0267372	0.769700459	0.608492612	0.973617074			
GCST90200861	CAC	outcome	Histidine to phosphate ratio	Inverse variance weighted (multiplicative random effects)	29	-0.178558818	0.073858139	0.0156236	-0.32332077	-0.033796867	0.836474855	0.723741662	0.966767868	0.618423493	0.006875416	
GCST90200861	CAC	outcome	Histidine to phosphate ratio	MR Egger	29	-0.261701887	0.181190007	0.160145764	-0.6168343	0.093430527	0.76974046	0.539650107	1.097934324		0.005479001	

GCST90200861	CAC	outcome	Histidine to phosphate ratio	Weighted median	29	-0.06801427	0.083976342	0.417985122	-0.2326079	0.09657936	0.934247142	0.792464238	1.101396985			
GCST90200861	CAC	outcome	Histidine to phosphate ratio	Weighted mode	29	-0.004473464	0.130013171	0.972796106	-0.259299278	0.250352351	0.995536527	0.771592068	1.284477924			
GCST90200861	CAC	outcome	Histidine to phosphate ratio	Contamination mixture method	29	-0.023985715	0.076530612	0.669075472	-0.173985715	0.126014285	0.976299656	0.840308902	1.134298372			
GCST90200861	CAC	outcome	Histidine to phosphate ratio	Robust adjusted profile score (RAPS)	29	-0.149470715	0.088410346	0.090904179	-0.322754992	0.023813562	0.861163657	0.724151255	1.02409937			
GCST90200862	CAC	outcome	Adenosine 5'-monophosphate (AMP) to methionine ratio	Inverse variance weighted	19	0.157112719	0.073400359	0.032315204	0.013248016	0.300977422	1.170127502	1.01333616	1.351178834	0.542922236	0.958913974	
GCST90200862	CAC	outcome	Adenosine 5'-monophosphate (AMP) to methionine ratio	MR Egger	19	0.270936984	0.197478801	0.187904676	-0.116121465	0.657995433	1.311192442	0.890367068	1.930917799		0.950604912	
GCST90200862	CAC	outcome	Adenosine 5'-monophosphate (AMP) to methionine ratio	Weighted median	19	0.078963652	0.098124419	0.420975571	-0.113360208	0.271287512	1.082164987	0.892828998	1.311652132			
GCST90200862	CAC	outcome	Adenosine 5'-monophosphate (AMP) to methionine ratio	Weighted mode	19	0.070848467	0.148149629	0.638248651	-0.219524805	0.36122174	1.073418555	0.802900241	1.43508164			
GCST90200862	CAC	outcome	Adenosine 5'-monophosphate (AMP) to methionine ratio	Contamination mixture method	19	0.179737458	0.12244898	0.1789447	-0.060262542	0.419737458	1.196903085	0.941517314	1.52156203			

GCST90200862	CAC	outcome	Adenosine 5'-monophosphate (AMP) to methionine ratio	Robust adjusted profile score (RAPS)	19	0.155545936	0.080461393	0.053214671	-0.002158395	0.313250266	1.168295601	0.997843933	1.367863819			
GCST90200863	CAC	outcome	Phosphate to serine ratio	Inverse variance weighted	24	0.121433034	0.056620051	0.031976827	0.010457733	0.232408335	1.129113751	1.010512607	1.261634793	0.621725125	0.15583691	
GCST90200863	CAC	outcome	Phosphate to serine ratio	MR Egger	24	0.047518748	0.158516511	0.767165385	-0.263173614	0.35821111	1.048665861	0.768608445	1.430767638		0.132607999	
GCST90200863	CAC	outcome	Phosphate to serine ratio	Weighted median	24	0.120661817	0.070993361	0.089202215	-0.018485172	0.259808805	1.128243295	0.981684631	1.296682144			
GCST90200863	CAC	outcome	Phosphate to serine ratio	Weighted mode	24	0.09979682	0.075638209	0.200020822	-0.04845407	0.248047711	1.104946393	0.952701096	1.281521073			
GCST90200863	CAC	outcome	Phosphate to serine ratio	Contamination mixture method	24	0.093715568	0.06122449	0.108236396	-0.026284432	0.213715568	1.098247325	0.974057997	1.238270401			
GCST90200863	CAC	outcome	Phosphate to serine ratio	Robust adjusted profile score (RAPS)	24	0.112940934	0.055982681	0.043651044	0.003214879	0.22266699	1.119565803	1.003220052	1.24940444			
GCST90200864	CAC	outcome	Methionine to phosphate ratio	Inverse variance weighted	13	-0.264499082	0.101631892	0.009254128	-0.46369759	-0.065300574	0.767590353	0.628953727	0.936785848	0.572736645	0.171822019	
GCST90200864	CAC	outcome	Methionine to phosphate ratio	MR Egger	13	-0.107439751	0.28969821	0.717779145	-0.675248242	0.46036874	0.898130632	0.509030042	1.584658203		0.14292721	

GCST90200864	CAC	outcome	Methionine to phosphate ratio	Weighted median	13	-0.114493791	0.119242583	0.33696696	-0.348209253	0.119221671	0.891817476	0.705951139	1.126619629			
GCST90200864	CAC	outcome	Methionine to phosphate ratio	Weighted mode	13	-0.053854581	0.185479983	0.776503183	-0.417395347	0.309686186	0.947569892	0.65876043	1.362997319			
GCST90200864	CAC	outcome	Methionine to phosphate ratio	Contamination mixture method	13	-0.129524996	0.086734694	0.428472209	-0.299524996	0.040475004	0.878512629	0.741170196	1.041305281			
GCST90200864	CAC	outcome	Methionine to phosphate ratio	Robust adjusted profile score (RAPS)	13	-0.219705257	0.102190828	0.031559102	-0.419999279	-0.019411235	0.80275537	0.657047293	0.98077595			
GCST90200892	CAC	outcome	Hypotaurine to taurine ratio	Inverse variance weighted	25	-0.180570725	0.074151397	0.014885091	-0.325907464	-0.035233986	0.834793638	0.721871984	0.965379505	0.778928082	0.085965566	
GCST90200892	CAC	outcome	Hypotaurine to taurine ratio	MR Egger	25	-0.252094735	0.262925154	0.347626877	-0.767428038	0.263238567	0.777171109	0.464205453	1.301137091		0.06797189	
GCST90200892	CAC	outcome	Hypotaurine to taurine ratio	Weighted median	25	-0.267879143	0.092196255	0.003666297	-0.448583802	-0.087174484	0.765000231	0.638531799	0.916517164			
GCST90200892	CAC	outcome	Hypotaurine to taurine ratio	Weighted mode	25	-0.279497375	0.14398504	0.064070671	-0.561708053	0.002713304	0.756163713	0.570234241	1.002716988			
GCST90200892	CAC	outcome	Hypotaurine to taurine ratio	Contamination mixture method	25	-0.333179174	0.102040816	0.006285933	-0.533179174	-0.133179174	0.716641779	0.586736664	0.875308246			

GCST90200892	CAC	outcome	Hypotaurine to taurine ratio	Robust adjusted profile score (RAPS)	25	-0.202140501	0.083452953	0.015426472	-0.365708288	-0.038572713	0.816980134	0.693705134	0.962161741			
GCST90200894	CAC	outcome	Inosine to theophylline ratio	Inverse variance weighted	21	-0.113953076	0.056948186	0.045392854	-0.225571521	-0.00233463	0.892299826	0.79805998	0.997668093	0.978994529	0.219927315	
GCST90200894	CAC	outcome	Inosine to theophylline ratio	MR Egger	21	-0.110635432	0.137398687	0.430657833	-0.379936858	0.158665994	0.895265074	0.683904591	1.171946444		0.176564712	
GCST90200894	CAC	outcome	Inosine to theophylline ratio	Weighted median	21	-0.13793487	0.072142467	0.05587891	-0.279334106	0.003464366	0.871155428	0.756287181	1.003470374			
GCST90200894	CAC	outcome	Inosine to theophylline ratio	Weighted mode	21	-0.222255947	0.130656059	0.104425734	-0.478341823	0.033829929	0.800710399	0.619810296	1.034408669			
GCST90200894	CAC	outcome	Inosine to theophylline ratio	Contamination mixture method	21	-0.200894868	0.076530612	0.009221528	-0.350894868	-0.050894868	0.817998425	0.704057769	0.950378581			
GCST90200894	CAC	outcome	Inosine to theophylline ratio	Robust adjusted profile score (RAPS)	21	-0.126414023	0.059292432	0.033003301	-0.242627191	-0.010200856	0.881249913	0.784563952	0.989850997			
GCST90200910	CAC	outcome	Retinol (Vitamin A) to oleoyl-linoleoyl-glycerol (18:1 to 18:2) [2] ratio	Inverse variance weighted	32	-0.130058588	0.045703377	0.004431243	-0.219637206	-0.04047997	0.878043986	0.80281	0.960328399	0.119950435	0.882940186	
GCST90200910	CAC	outcome	Retinol (Vitamin A) to oleoyl-linoleoyl-glycerol (18:1 to 18:2) [2] ratio	MR Egger	32	-0.293283659	0.111751985	0.013521082	-0.51231755	-0.074249767	0.745810555	0.599105512	0.928439771		0.930300762	

GCST90200910	CAC	outcome	Retinol (Vitamin A) to oleoyl-linoleoyl-glycerol (18:1 to 18:2) [2] ratio	Weighted median	32	-0.149038139	0.066664813	0.025375738	-0.279701172	-0.018375106	0.861536256	0.756009624	0.981792687			
GCST90200910	CAC	outcome	Retinol (Vitamin A) to oleoyl-linoleoyl-glycerol (18:1 to 18:2) [2] ratio	Weighted mode	32	-0.21284173	0.099455347	0.040327003	-0.407774211	-0.017909249	0.808284055	0.665129041	0.982250169			
GCST90200910	CAC	outcome	Retinol (Vitamin A) to oleoyl-linoleoyl-glycerol (18:1 to 18:2) [2] ratio	Contamination mixture method	32	-0.218674017	0.06377551	0.006707301	-0.343674017	-0.093674017	0.80358363	0.709160064	0.910579547			
GCST90200910	CAC	outcome	Retinol (Vitamin A) to oleoyl-linoleoyl-glycerol (18:1 to 18:2) [2] ratio	Robust adjusted profile score (RAPS)	32	-0.135499983	0.049047206	0.005733522	-0.231632507	-0.039367459	0.873279178	0.793237579	0.96139737			
GCST90200942	CAC	outcome	Glutamate to pyruvate ratio	Inverse variance weighted	25	0.212785519	0.07393008	0.003999521	0.067882563	0.357688476	1.237119284	1.070239615	1.430020065	0.987888637	0.097695807	
GCST90200942	CAC	outcome	Glutamate to pyruvate ratio	MR Egger	25	0.215296466	0.180211379	0.244383645	-0.137917836	0.568510769	1.240229529	0.871170268	1.765635653		0.07581761	
GCST90200942	CAC	outcome	Glutamate to pyruvate ratio	Weighted median	25	0.167660419	0.096280612	0.081618291	-0.021049582	0.356370419	1.182534976	0.979170415	1.42813646			
GCST90200942	CAC	outcome	Glutamate to pyruvate ratio	Weighted mode	25	0.086283604	0.183422377	0.64230953	-0.273224254	0.445791463	1.090115446	0.760922129	1.561725754			
GCST90200942	CAC	outcome	Glutamate to pyruvate ratio	Contamination mixture method	25	0.417793106	0.130102041	0.002928499	0.162793106	0.672793106	1.518606451	1.176793192	1.959703342			

GCST90200942	CAC	outcome	Glutamate to pyruvate ratio	Robust adjusted profile score (RAPS)	25	0.240354547	0.082083204	0.003409506	0.079471466	0.401237627	1.271699947	1.082714665	1.493672163			
GCST90200964	CAC	outcome	Adenosine 5'-diphosphate (ADP) to 5-oxoproline ratio	Inverse variance weighted	16	-0.122135918	0.053641399	0.022792403	-0.22727306	-0.016998775	0.885028069	0.796703204	0.983144889	0.368334183	0.800333522	
GCST90200964	CAC	outcome	Adenosine 5'-diphosphate (ADP) to 5-oxoproline ratio	MR Egger	16	-0.009205985	0.132799031	0.945713207	-0.269492085	0.251080115	0.99083626	0.763767325	1.285413061		0.802029202	
GCST90200964	CAC	outcome	Adenosine 5'-diphosphate (ADP) to 5-oxoproline ratio	Weighted median	16	-0.14510126	0.073430114	0.048149602	-0.289024283	-0.001178237	0.864934705	0.748994017	0.998822457			
GCST90200964	CAC	outcome	Adenosine 5'-diphosphate (ADP) to 5-oxoproline ratio	Weighted mode	16	-0.136041962	0.109796692	0.234370751	-0.351243478	0.079159554	0.872806007	0.70381237	1.082377006			
GCST90200964	CAC	outcome	Adenosine 5'-diphosphate (ADP) to 5-oxoproline ratio	Contamination mixture method	16	-0.130448834	0.086734694	0.077397916	-0.300448834	0.039551166	0.8777014	0.740485791	1.040343728			
GCST90200964	CAC	outcome	Adenosine 5'-diphosphate (ADP) to 5-oxoproline ratio	Robust adjusted profile score (RAPS)	16	-0.124638441	0.058319858	0.032585574	-0.238945362	-0.01033152	0.882816035	0.787457907	0.989721667			
GCST90200966	CAC	outcome	Adenosine 5'-diphosphate (ADP) to uridine ratio	Inverse variance weighted	13	-0.126982808	0.060227567	0.034997832	-0.245028839	-0.008936776	0.880748815	0.782681966	0.991103038	0.991725928	0.988544483	
GCST90200966	CAC	outcome	Adenosine 5'-diphosphate (ADP) to uridine ratio	MR Egger	13	-0.129328009	0.229129932	0.583786675	-0.578422675	0.319766657	0.878685701	0.560782205	1.376806458		0.978365508	

GCST90200966	CAC	outcome	Adenosine 5'-diphosphate (ADP) to uridine ratio	Weighted median	13	-0.145211945	0.07603232	0.056149786	-0.294235292	0.003811401	0.864838975	0.745101155	1.003818674			
GCST90200966	CAC	outcome	Adenosine 5'-diphosphate (ADP) to uridine ratio	Weighted mode	13	-0.205996411	0.129712145	0.138248058	-0.460232215	0.048239394	0.813835997	0.631137069	1.04942185			
GCST90200966	CAC	outcome	Adenosine 5'-diphosphate (ADP) to uridine ratio	Contamination mixture method	13	-0.167282359	0.081632653	0.021412522	-0.327282359	-0.007282359	0.845960713	0.720880167	0.992744093			
GCST90200966	CAC	outcome	Adenosine 5'-diphosphate (ADP) to uridine ratio	Robust adjusted profile score (RAPS)	13	-0.128789059	0.066571333	0.05303911	-0.259268873	0.001690754	0.879159397	0.771615529	1.001692185			
GCST90201012	CAC	outcome	3-methyl-2-oxovalerate to 3-methyl-2-oxobutyrate ratio	Inverse variance weighted	20	0.173982936	0.069536259	0.012347779	0.037691869	0.310274003	1.190035259	1.038411217	1.363798748	0.865279644	0.958356844	
GCST90201012	CAC	outcome	3-methyl-2-oxovalerate to 3-methyl-2-oxobutyrate ratio	MR Egger	20	0.198160674	0.156754367	0.222302557	-0.109077886	0.505399234	1.219158265	0.896660577	1.657647178		0.940003276	
GCST90201012	CAC	outcome	3-methyl-2-oxovalerate to 3-methyl-2-oxobutyrate ratio	Weighted median	20	0.184455913	0.089735888	0.039826656	0.008573572	0.360338253	1.202563962	1.00861043	1.433814325			
GCST90201012	CAC	outcome	3-methyl-2-oxovalerate to 3-methyl-2-oxobutyrate ratio	Weighted mode	20	0.227167451	0.1453098	0.134477468	-0.057639758	0.51197466	1.255040008	0.943989951	1.668582827			
GCST90201012	CAC	outcome	3-methyl-2-oxovalerate to 3-methyl-2-oxobutyrate ratio	Contamination mixture method	20	0.231105018	0.104591837	0.022939339	0.026105018	0.436105018	1.259991554	1.026448738	1.546671215			

GCST90201012	CAC	outcome	3-methyl-2-oxovalerate to 3-methyl-2-oxobutyrate ratio	Robust adjusted profile score (RAPS)	20	0.172826748	0.076516103	0.023902194	0.022855186	0.322798311	1.18866015	1.023118367	1.380986792		

nsnp: number of single nucleotide polymorphisms, se: standard error, lo_ci: lower confidence interval, up_ci: upper confidence interval, or: odds ratio, or_lci95: odds ratio lower 95% confidence interval limit, or_uci95: odds ratio upper 95% confidence interval limit, p-FDR: p-value after False Discovery Rate correction

Table S5 Mendelian Randomization analysis on the causal effect between coronary artery calcification and circulating metabolites.

id.exposure	id.outcome	outcome	exposure	method	nsnp	b	se	pval	lo_ci	up_ci	or	or_lci95	or_uci95	pleiotropy	heterogeneity	p_fdr
CAC	ebi-met1400-GCST90199623	Maltotriose levels || id:ebi-met1400-GCST90199623	exposure	Inverse variance weighted	5	0.134128	0.063028	0.033332	0.010593	0.257662	1.143539	1.010649	1.293902	0.200179726	0.345300973	0.935072545
CAC	ebi-met1400-GCST90199623	Maltotriose levels || id:ebi-met1400-GCST90199623	exposure	Robust adjusted profile score (RAPS)	5	0.130544	0.063504	0.039812	0.006077	0.255011	1.139448	1.006096	1.290476		0.615475427	0.988238216
CAC	ebi-met1400-GCST90199623	Maltotriose levels || id:ebi-met1400-GCST90199623	exposure	MR Egger	5	0.421011	0.185113	0.107493	0.05819	0.783832	1.523501	1.059916	2.189847			0.995501177
CAC	ebi-met1400-GCST90199623	Maltotriose levels || id:ebi-met1400-GCST90199623	exposure	Contamination mixture method	5	0.308071	0.158163	0.241991	-0.00193	0.618071	1.360798	0.998073	1.855346			0.71490601
CAC	ebi-met1400-GCST90199623	Maltotriose levels || id:ebi-met1400-GCST90199623	exposure	Weighted median	5	0.048972	0.07706	0.525098	-0.10207	0.200009	1.050191	0.902971	1.221414			0.999387558
CAC	ebi-met1400-	Maltotriose levels || id:ebi-met1400-GCST90199623	exposure	Weighted mode	5	0.047345	0.091605	0.632534	-0.1322	0.22689	1.048483	0.876165	1.254692			0.998768904

	GCST90199623															
CAC	ebi-met1400-GCST90199625	Methionine sulfoxide levels || id:ebi-met1400-GCST90199625	exposure	Inverse variance weighted	5	-0.10281	0.048984	0.035836	-0.19881	-0.0068	0.902302	0.819703	0.993225	0.556576519	0.300705975	0.935072545
CAC	ebi-met1400-GCST90199625	Methionine sulfoxide levels || id:ebi-met1400-GCST90199625	exposure	Robust adjusted profile score (RAPS)	5	-0.10338	0.053994	0.055536	-0.20921	0.002448	0.901784	0.811226	1.002451		0.235250225	0.988238216
CAC	ebi-met1400-GCST90199625	Methionine sulfoxide levels || id:ebi-met1400-GCST90199625	exposure	Contamination mixture method	5	-0.24591	0.119898	0.235758	-0.48091	-0.01091	0.781995	0.618223	0.989153			0.712811518
CAC	ebi-met1400-GCST90199625	Methionine sulfoxide levels || id:ebi-met1400-GCST90199625	exposure	Weighted median	5	-0.05778	0.057266	0.313	-0.17002	0.054463	0.943859	0.843647	1.055974			0.999387558
CAC	ebi-met1400-GCST90199625	Methionine sulfoxide levels || id:ebi-met1400-GCST90199625	exposure	Weighted mode	5	-0.04006	0.066106	0.577237	-0.16963	0.089509	0.960733	0.84398	1.093638			0.998768904
CAC	ebi-met1400-GCST90199625	Methionine sulfoxide levels || id:ebi-met1400-GCST90199625	exposure	MR Egger	5	-0.00049	0.163856	0.997799	-0.32165	0.320667	0.99951	0.724953	1.378047			0.998512373

CAC	ebi-met1400-GCST90199633	Suberate (C8-DC) levels || id:ebi-met1400-GCST90199633	exposure	Robust adjusted profile score (RAPS)	5	-0.11908	0.050552	0.018492	-0.21816	-0.02	0.887736	0.803995	0.980199	0.722821683	0.375546719	0.988238216
CAC	ebi-met1400-GCST90199633	Suberate (C8-DC) levels || id:ebi-met1400-GCST90199633	exposure	Contamination mixture method	5	-0.18507	0.063776	0.022937	-0.31007	-0.06007	0.831049	0.733398	0.941702		0.258462941	0.712811518
CAC	ebi-met1400-GCST90199633	Suberate (C8-DC) levels || id:ebi-met1400-GCST90199633	exposure	Inverse variance weighted	5	-0.108	0.049023	0.027586	-0.20409	-0.01192	0.897623	0.815389	0.988152			0.934078042
CAC	ebi-met1400-GCST90199633	Suberate (C8-DC) levels || id:ebi-met1400-GCST90199633	exposure	Weighted median	5	-0.13802	0.065695	0.035648	-0.26678	-0.00926	0.871082	0.765841	0.990785			0.999387558
CAC	ebi-met1400-GCST90199633	Suberate (C8-DC) levels || id:ebi-met1400-GCST90199633	exposure	Weighted mode	5	-0.1859	0.080336	0.081673	-0.34336	-0.02844	0.830359	0.709386	0.971962			0.998768904
CAC	ebi-met1400-GCST90199633	Suberate (C8-DC) levels || id:ebi-met1400-GCST90199633	exposure	MR Egger	5	-0.17098	0.170799	0.390568	-0.50574	0.163788	0.84284	0.603056	1.177964			0.995501177

CAC	ebi-met1400-GCST90199638	Glycerol 3-phosphate levels || id:ebi-met1400-GCST90199638	exposure	Inverse variance weighted	5	-0.09237	0.044534	0.038071	-0.17966	-0.00508	0.911769	0.835558	0.994932	0.772456978	0.987646672	0.935072545
CAC	ebi-met1400-GCST90199638	Glycerol 3-phosphate levels || id:ebi-met1400-GCST90199638	exposure	Contamination mixture method	5	-0.0905	0.053571	0.047072	-0.1955	0.014501	0.913475	0.822424	1.014607		0.972258127	0.712811518
CAC	ebi-met1400-GCST90199638	Glycerol 3-phosphate levels || id:ebi-met1400-GCST90199638	exposure	Robust adjusted profile score (RAPS)	5	-0.09252	0.047436	0.051139	-0.18549	0.00046	0.911635	0.830697	1.00046			0.988238216
CAC	ebi-met1400-GCST90199638	Glycerol 3-phosphate levels || id:ebi-met1400-GCST90199638	exposure	Weighted median	5	-0.09607	0.05306	0.0702	-0.20007	0.007926	0.908399	0.818674	1.007958			0.999387558
CAC	ebi-met1400-GCST90199638	Glycerol 3-phosphate levels || id:ebi-met1400-GCST90199638	exposure	Weighted mode	5	-0.10103	0.065706	0.198956	-0.22982	0.027752	0.903904	0.794679	1.02814			0.998768904
CAC	ebi-met1400-GCST90199638	Glycerol 3-phosphate levels || id:ebi-met1400-GCST90199638	exposure	MR Egger	5	-0.05104	0.138026	0.736123	-0.32157	0.219495	0.950244	0.725012	1.245447			0.995501177

CAC	ebi-met1400-GCST90199640	4-guanidinobutanoate levels || id:ebi-met1400-GCST90199640	exposure	Inverse variance weighted	5	-0.11316	0.054101	0.036467	-0.2192	-0.00712	0.893005	0.80316	0.992901	0.695118378	0.171074515	0.935072545
CAC	ebi-met1400-GCST90199640	4-guanidinobutanoate levels || id:ebi-met1400-GCST90199640	exposure	Contamination mixture method	5	-0.15238	0.102041	0.067923	-0.35238	0.04762	0.858662	0.703013	1.048773		0.110277924	0.712811518
CAC	ebi-met1400-GCST90199640	4-guanidinobutanoate levels || id:ebi-met1400-GCST90199640	exposure	Weighted median	5	-0.0949	0.056594	0.09358	-0.20582	0.016027	0.909467	0.813979	1.016156			0.999387558
CAC	ebi-met1400-GCST90199640	4-guanidinobutanoate levels || id:ebi-met1400-GCST90199640	exposure	Robust adjusted profile score (RAPS)	5	-0.11439	0.094587	0.226521	-0.29978	0.071	0.891909	0.74098	1.073581			0.988238216
CAC	ebi-met1400-GCST90199640	4-guanidinobutanoate levels || id:ebi-met1400-GCST90199640	exposure	Weighted mode	5	-0.09571	0.075261	0.272397	-0.24322	0.051804	0.90873	0.7841	1.053169			0.998768904
CAC	ebi-met1400-GCST90199640	4-guanidinobutanoate levels || id:ebi-met1400-GCST90199640	exposure	MR Egger	5	-0.03645	0.187768	0.858492	-0.40447	0.331578	0.964209	0.667328	1.393165			0.995501177

CAC	ebi-met1400-GCST90199688	N-acetylglycine levels || id:ebi-met1400-GCST90199688	exposure	Inverse variance weighted	5	0.098564	0.043	0.021896	0.014283	0.182844	1.103585	1.014386	1.200627	0.59277333	0.404022369	0.934078042
CAC	ebi-met1400-GCST90199688	N-acetylglycine levels || id:ebi-met1400-GCST90199688	exposure	Robust adjusted profile score (RAPS)	5	0.093675	0.045242	0.038401	0.005001	0.182348	1.098203	1.005014	1.200032		0.309422031	0.988238216
CAC	ebi-met1400-GCST90199688	N-acetylglycine levels || id:ebi-met1400-GCST90199688	exposure	Contamination mixture method	5	0.143253	0.122449	0.154124	-0.09675	0.383253	1.154022	0.907786	1.467049			0.712811518
CAC	ebi-met1400-GCST90199688	N-acetylglycine levels || id:ebi-met1400-GCST90199688	exposure	Weighted median	5	0.068784	0.052031	0.186177	-0.0332	0.170766	1.071205	0.967348	1.186213			0.999387558
CAC	ebi-met1400-GCST90199688	N-acetylglycine levels || id:ebi-met1400-GCST90199688	exposure	MR Egger	5	0.180663	0.145391	0.30229	-0.1043	0.465629	1.198011	0.900952	1.593015			0.995501177
CAC	ebi-met1400-GCST90199688	N-acetylglycine levels || id:ebi-met1400-GCST90199688	exposure	Weighted mode	5	0.044188	0.059004	0.495553	-0.07146	0.159836	1.045179	0.931034	1.173318			0.998768904

CAC	ebi-met1400-GCST90199689	Glycolithocholate levels || id:ebi-met1400-GCST90199689	exposure	Inverse variance weighted	5	-0.11478	0.047757	0.016248	-0.20838	-0.02117	0.891566	0.811898	0.979052	0.728909937	0.69445212	0.923524134
CAC	ebi-met1400-GCST90199689	Glycolithocholate levels || id:ebi-met1400-GCST90199689	exposure	Robust adjusted profile score (RAPS)	5	-0.11578	0.050869	0.022846	-0.21548	-0.01607	0.890674	0.806154	0.984054		0.555923441	0.988238216
CAC	ebi-met1400-GCST90199689	Glycolithocholate levels || id:ebi-met1400-GCST90199689	exposure	Contamination mixture method	5	-0.11398	0.084184	0.036342	-0.27898	0.051024	0.892279	0.756558	1.052348			0.712811518
CAC	ebi-met1400-GCST90199689	Glycolithocholate levels || id:ebi-met1400-GCST90199689	exposure	Weighted median	5	-0.099	0.055569	0.074826	-0.20791	0.009918	0.905745	0.812278	1.009967			0.999387558
CAC	ebi-met1400-GCST90199689	Glycolithocholate levels || id:ebi-met1400-GCST90199689	exposure	Weighted mode	5	-0.09577	0.065451	0.217223	-0.22406	0.03251	0.908669	0.799269	1.033044			0.998768904
CAC	ebi-met1400-GCST90199689	Glycolithocholate levels || id:ebi-met1400-GCST90199689	exposure	MR Egger	5	-0.1679	0.14755	0.337813	-0.4571	0.121301	0.84544	0.633119	1.128964			0.995501177

CAC	ebi-met1400-GCST90199708	Propionylcarnitine (c3) levels || id:ebi-met1400-GCST90199708	exposure	Inverse variance weighted	5	-0.11999	0.042575	0.004829	-0.20343	-0.03654	0.886932	0.815924	0.96412	0.430422289	0.610509401	0.763614945
CAC	ebi-met1400-GCST90199708	Propionylcarnitine (c3) levels || id:ebi-met1400-GCST90199708	exposure	Robust adjusted profile score (RAPS)	5	-0.12087	0.045381	0.007735	-0.20982	-0.03192	0.88615	0.810733	0.968582		0.600561636	0.988238216
CAC	ebi-met1400-GCST90199708	Propionylcarnitine (c3) levels || id:ebi-met1400-GCST90199708	exposure	Contamination mixture method	5	-0.14461	0.048469	0.01096	-0.23961	-0.04961	0.865363	0.786937	0.951604			0.712811518
CAC	ebi-met1400-GCST90199708	Propionylcarnitine (c3) levels || id:ebi-met1400-GCST90199708	exposure	Weighted median	5	-0.11768	0.052014	0.023664	-0.21963	-0.01574	0.888978	0.802817	0.984387			0.999387558
CAC	ebi-met1400-GCST90199708	Propionylcarnitine (c3) levels || id:ebi-met1400-GCST90199708	exposure	Weighted mode	5	-0.12464	0.062274	0.115929	-0.24669	-0.00258	0.882818	0.781379	0.997424			0.998768904
CAC	ebi-met1400-GCST90199708	Propionylcarnitine (c3) levels || id:ebi-met1400-GCST90199708	exposure	MR Egger	5	-0.23342	0.131871	0.174851	-0.49189	0.025044	0.791818	0.61147	1.02536			0.995501177

CAC	ebi-met1400-GCST90199709	Adrenate (22:4n6) levels || id:ebi-met1400-GCST90199709	exposure	Inverse variance weighted	5	0.093913	0.043701	0.031637	0.008258	0.179568	1.098464	1.008293	1.1967	0.629220563	0.774019227	0.935072545
CAC	ebi-met1400-GCST90199709	Adrenate (22:4n6) levels || id:ebi-met1400-GCST90199709	exposure	Robust adjusted profile score (RAPS)	5	0.094458	0.046452	0.042005	0.003413	0.185503	1.099063	1.003419	1.203824		0.681250081	0.988238216
CAC	ebi-met1400-GCST90199709	Adrenate (22:4n6) levels || id:ebi-met1400-GCST90199709	exposure	Contamination mixture method	5	0.09403	0.076531	0.049802	-0.05597	0.24403	1.098592	0.945567	1.276382			0.712811518
CAC	ebi-met1400-GCST90199709	Adrenate (22:4n6) levels || id:ebi-met1400-GCST90199709	exposure	Weighted median	5	0.087152	0.052684	0.098075	-0.01611	0.190412	1.091063	0.984021	1.209748			0.999387558
CAC	ebi-met1400-GCST90199709	Adrenate (22:4n6) levels || id:ebi-met1400-GCST90199709	exposure	Weighted mode	5	0.069624	0.062798	0.329731	-0.05346	0.192707	1.072105	0.947944	1.212528			0.998768904
CAC	ebi-met1400-GCST90199709	Adrenate (22:4n6) levels || id:ebi-met1400-GCST90199709	exposure	MR Egger	5	0.025258	0.135363	0.863883	-0.24005	0.290569	1.02558	0.786586	1.337188			0.995501177

CAC	ebi-met1400-GCST90199793	1-linoleoyl-GPE (18:2) levels || id:ebi-met1400-GCST90199793	exposure	Inverse variance weighted	5	-0.10226	0.044145	0.020531	-0.18879	-0.01574	0.902792	0.827962	0.984385	0.480657755	0.618409523	0.934078042
CAC	ebi-met1400-GCST90199793	1-linoleoyl-GPE (18:2) levels || id:ebi-met1400-GCST90199793	exposure	Robust adjusted profile score (RAPS)	5	-0.10344	0.046938	0.027538	-0.19544	-0.01144	0.901728	0.822472	0.988622		0.571851902	0.988238216
CAC	ebi-met1400-GCST90199793	1-linoleoyl-GPE (18:2) levels || id:ebi-met1400-GCST90199793	exposure	Weighted median	5	-0.09807	0.053394	0.066254	-0.20272	0.006584	0.906587	0.816506	1.006605			0.999387558
CAC	ebi-met1400-GCST90199793	1-linoleoyl-GPE (18:2) levels || id:ebi-met1400-GCST90199793	exposure	Weighted mode	5	-0.16068	0.069046	0.0805	-0.29601	-0.02535	0.851563	0.743779	0.974966			0.998768904
CAC	ebi-met1400-GCST90199793	1-linoleoyl-GPE (18:2) levels || id:ebi-met1400-GCST90199793	exposure	Contamination mixture method	5	-0.15951	0.081633	0.103914	-0.31951	0.000489	0.852561	0.726505	1.00049			0.712811518
CAC	ebi-met1400-GCST90199793	1-linoleoyl-GPE (18:2) levels || id:ebi-met1400-GCST90199793	exposure	MR Egger	5	-0.20624	0.136796	0.228748	-0.47437	0.061875	0.813634	0.62228	1.06383			0.995501177

CAC	ebi-met1400-GCST90199799	Glutarylcarnitine (c5-dc) levels || id:ebi-met1400-GCST90199799	exposure	Inverse variance weighted	5	0.147768	0.043258	0.000636	0.062982	0.232554	1.159244	1.065007	1.261818	0.831037845	0.896227333	0.513600644
CAC	ebi-met1400-GCST90199799	Glutarylcarnitine (c5-dc) levels || id:ebi-met1400-GCST90199799	exposure	Robust adjusted profile score (RAPS)	5	0.148505	0.046702	0.001474	0.056969	0.240041	1.160099	1.058623	1.271302		0.793136181	0.988238216
CAC	ebi-met1400-GCST90199799	Glutarylcarnitine (c5-dc) levels || id:ebi-met1400-GCST90199799	exposure	Contamination mixture method	5	0.144633	0.040816	0.004006	0.064633	0.224633	1.155615	1.066767	1.251863			0.712811518
CAC	ebi-met1400-GCST90199799	Glutarylcarnitine (c5-dc) levels || id:ebi-met1400-GCST90199799	exposure	Weighted median	5	0.132022	0.051289	0.010051	0.031495	0.23255	1.141134	1.031996	1.261813			0.999387558
CAC	ebi-met1400-GCST90199799	Glutarylcarnitine (c5-dc) levels || id:ebi-met1400-GCST90199799	exposure	Weighted mode	5	0.116634	0.056646	0.108577	0.005607	0.22766	1.123708	1.005623	1.255659			0.998768904
CAC	ebi-met1400-GCST90199799	Glutarylcarnitine (c5-dc) levels || id:ebi-met1400-GCST90199799	exposure	MR Egger	5	0.118248	0.134083	0.442771	-0.14455	0.38105	1.125523	0.865407	1.463821			0.995501177

CAC	ebi-met1400-GCST90199826	N6-acetyllysine levels || id:ebi-met1400-GCST90199826	exposure	Inverse variance weighted	5	-0.09571	0.043497	0.027781	-0.18096	-0.01046	0.908729	0.834467	0.989599	0.579847122	0.954954398	0.934078042
CAC	ebi-met1400-GCST90199826	N6-acetyllysine levels || id:ebi-met1400-GCST90199826	exposure	Robust adjusted profile score (RAPS)	5	-0.09595	0.046307	0.038258	-0.18671	-0.00519	0.908508	0.829682	0.994824		0.96240906	0.988238216
CAC	ebi-met1400-GCST90199826	N6-acetyllysine levels || id:ebi-met1400-GCST90199826	exposure	Contamination mixture method	5	-0.09103	0.068878	0.040275	-0.22603	0.043974	0.912994	0.797697	1.044955			0.712811518
CAC	ebi-met1400-GCST90199826	N6-acetyllysine levels || id:ebi-met1400-GCST90199826	exposure	Weighted median	5	-0.09269	0.051342	0.071016	-0.19332	0.007939	0.911475	0.824217	1.00797			0.999387558
CAC	ebi-met1400-GCST90199826	N6-acetyllysine levels || id:ebi-met1400-GCST90199826	exposure	Weighted mode	5	-0.08633	0.061398	0.232443	-0.20667	0.034014	0.917294	0.81329	1.034599			0.998768904
CAC	ebi-met1400-GCST90199826	N6-acetyllysine levels || id:ebi-met1400-GCST90199826	exposure	MR Egger	5	-0.17464	0.134756	0.285694	-0.43876	0.089488	0.839763	0.644837	1.093614			0.995501177

<table>
<tr><td>CAC</td><td>ebi-met1400-GCST90199844</td><td>1-palmitoyl-2-linoleoyl-GPE (16:0/18:2) levels || id:ebi-met1400-GCST90199844</td><td>exposure</td><td>Inverse variance weighted</td><td>5</td><td>-0.11553</td><td>0.043961</td><td>0.008588</td><td>-0.20169</td><td>-0.02937</td><td>0.890893</td><td>0.817345</td><td>0.971059</td><td>0.644004769</td><td>0.527536953</td><td>0.801502092</td></tr>
<tr><td>CAC</td><td>ebi-met1400-GCST90199844</td><td>1-palmitoyl-2-linoleoyl-GPE (16:0/18:2) levels || id:ebi-met1400-GCST90199844</td><td>exposure</td><td>Robust adjusted profile score (RAPS)</td><td>5</td><td>-0.11286</td><td>0.046691</td><td>0.015644</td><td>-0.20437</td><td>-0.02134</td><td>0.893279</td><td>0.81516</td><td>0.978883</td><td></td><td>0.403836674</td><td>0.988238216</td></tr>
<tr><td>CAC</td><td>ebi-met1400-GCST90199844</td><td>1-palmitoyl-2-linoleoyl-GPE (16:0/18:2) levels || id:ebi-met1400-GCST90199844</td><td>exposure</td><td>Contamination mixture method</td><td>5</td><td>-0.11434</td><td>0.066327</td><td>0.066693</td><td>-0.24434</td><td>0.015663</td><td>0.891958</td><td>0.783224</td><td>1.015787</td><td></td><td></td><td>0.712811518</td></tr>
<tr><td>CAC</td><td>ebi-met1400-GCST90199844</td><td>1-palmitoyl-2-linoleoyl-GPE (16:0/18:2) levels || id:ebi-met1400-GCST90199844</td><td>exposure</td><td>Weighted median</td><td>5</td><td>-0.09322</td><td>0.054642</td><td>0.088006</td><td>-0.20032</td><td>0.013878</td><td>0.910993</td><td>0.818471</td><td>1.013975</td><td></td><td></td><td>0.999387558</td></tr>
<tr><td>CAC</td><td>ebi-met1400-GCST90199844</td><td>1-palmitoyl-2-linoleoyl-GPE (16:0/18:2) levels || id:ebi-met1400-GCST90199844</td><td>exposure</td><td>Weighted mode</td><td>5</td><td>-0.09131</td><td>0.063188</td><td>0.221959</td><td>-0.21516</td><td>0.032539</td><td>0.912735</td><td>0.806414</td><td>1.033074</td><td></td><td></td><td>0.998768904</td></tr>
<tr><td>CAC</td><td>ebi-met1400-GCST90199844</td><td>1-palmitoyl-2-linoleoyl-GPE (16:0/18:2) levels || id:ebi-met1400-GCST90199844</td><td>exposure</td><td>MR Egger</td><td>5</td><td>-0.1815</td><td>0.13616</td><td>0.27472</td><td>-0.44838</td><td>0.085372</td><td>0.834016</td><td>0.638665</td><td>1.089122</td><td></td><td></td><td>0.995501177</td></tr>
</table>

<table>
<tr><td>CAC</td><td>ebi-met1400-GCST90199864</td><td>Gamma-CEHC levels || id:ebi-met1400-GCST90199864</td><td>exposure</td><td>Inverse variance weighted</td><td>5</td><td>-0.09598</td><td>0.044343</td><td>0.030421</td><td>-0.18289</td><td>-0.00907</td><td>0.90848</td><td>0.832856</td><td>0.99097</td><td>0.797731588</td><td>0.910779051</td><td>0.935072545</td></tr>
<tr><td>CAC</td><td>ebi-met1400-GCST90199864</td><td>Gamma-CEHC levels || id:ebi-met1400-GCST90199864</td><td>exposure</td><td>Robust adjusted profile score (RAPS)</td><td>5</td><td>-0.09629</td><td>0.047175</td><td>0.041242</td><td>-0.18875</td><td>-0.00383</td><td>0.908201</td><td>0.827991</td><td>0.996182</td><td></td><td>0.821769775</td><td>0.988238216</td></tr>
<tr><td>CAC</td><td>ebi-met1400-GCST90199864</td><td>Gamma-CEHC levels || id:ebi-met1400-GCST90199864</td><td>exposure</td><td>Contamination mixture method</td><td>5</td><td>-0.10854</td><td>0.063776</td><td>0.043667</td><td>-0.23354</td><td>0.01646</td><td>0.897143</td><td>0.791726</td><td>1.016596</td><td></td><td></td><td>0.712811518</td></tr>
<tr><td>CAC</td><td>ebi-met1400-GCST90199864</td><td>Gamma-CEHC levels || id:ebi-met1400-GCST90199864</td><td>exposure</td><td>Weighted median</td><td>5</td><td>-0.09733</td><td>0.054352</td><td>0.073346</td><td>-0.20386</td><td>0.009203</td><td>0.907259</td><td>0.815578</td><td>1.009246</td><td></td><td></td><td>0.999387558</td></tr>
<tr><td>CAC</td><td>ebi-met1400-GCST90199864</td><td>Gamma-CEHC levels || id:ebi-met1400-GCST90199864</td><td>exposure</td><td>Weighted mode</td><td>5</td><td>-0.09458</td><td>0.060651</td><td>0.193909</td><td>-0.21346</td><td>0.024296</td><td>0.909755</td><td>0.807788</td><td>1.024594</td><td></td><td></td><td>0.998768904</td></tr>
<tr><td>CAC</td><td>ebi-met1400-GCST90199864</td><td>Gamma-CEHC levels || id:ebi-met1400-GCST90199864</td><td>exposure</td><td>MR Egger</td><td>5</td><td>-0.13237</td><td>0.137344</td><td>0.406255</td><td>-0.40156</td><td>0.136826</td><td>0.876018</td><td>0.669272</td><td>1.146629</td><td></td><td></td><td>0.995501177</td></tr>
</table>

CAC	ebi-met1400-GCST90199889	2R,3R-dihydroxybutyrate levels || id:ebi-met1400-GCST90199889	exposure	Inverse variance weighted	5	-0.09987	0.043767	0.022495	-0.18566	-0.01409	0.904953	0.830559	0.98601	0.456382319	0.573509624	0.934078042
CAC	ebi-met1400-GCST90199889	2R,3R-dihydroxybutyrate levels || id:ebi-met1400-GCST90199889	exposure	Robust adjusted profile score (RAPS)	5	-0.10074	0.046385	0.029876	-0.19165	-0.00982	0.904172	0.825595	0.990227		0.535998597	0.988238216
CAC	ebi-met1400-GCST90199889	2R,3R-dihydroxybutyrate levels || id:ebi-met1400-GCST90199889	exposure	Contamination mixture method	5	-0.11689	0.071429	0.046547	-0.25689	0.023112	0.889685	0.773455	1.023381			0.712811518
CAC	ebi-met1400-GCST90199889	2R,3R-dihydroxybutyrate levels || id:ebi-met1400-GCST90199889	exposure	Weighted median	5	-0.10263	0.053444	0.054825	-0.20738	0.002124	0.902463	0.812712	1.002126			0.999387558
CAC	ebi-met1400-GCST90199889	2R,3R-dihydroxybutyrate levels || id:ebi-met1400-GCST90199889	exposure	MR Egger	5	-0.20926	0.135515	0.220236	-0.47487	0.056346	0.811182	0.621965	1.057963			0.995501177
CAC	ebi-met1400-GCST90199889	2R,3R-dihydroxybutyrate levels || id:ebi-met1400-GCST90199889	exposure	Weighted mode	5	-0.10134	0.071303	0.228278	-0.2411	0.038411	0.903622	0.785764	1.039158			0.998768904

CAC	ebi-met1400-GCST90199890	4-hydroxyglutamate levels || id:ebi-met1400-GCST90199890	exposure	Inverse variance weighted	5	-0.10348	0.045893	0.024144	-0.19343	-0.01353	0.901694	0.824127	0.986561	0.283521231	0.430765615	0.934078042
CAC	ebi-met1400-GCST90199890	4-hydroxyglutamate levels || id:ebi-met1400-GCST90199890	exposure	Robust adjusted profile score (RAPS)	5	-0.09766	0.048374	0.0435	-0.19247	-0.00285	0.906957	0.824917	0.997156		0.547304951	0.988238216
CAC	ebi-met1400-GCST90199890	4-hydroxyglutamate levels || id:ebi-met1400-GCST90199890	exposure	MR Egger	5	-0.27823	0.141735	0.144423	-0.55603	-0.00043	0.757121	0.573479	0.999569			0.995501177
CAC	ebi-met1400-GCST90199890	4-hydroxyglutamate levels || id:ebi-met1400-GCST90199890	exposure	Contamination mixture method	5	-0.07168	0.127551	0.181392	-0.32168	0.178324	0.930832	0.724933	1.195212			0.712811518
CAC	ebi-met1400-GCST90199890	4-hydroxyglutamate levels || id:ebi-met1400-GCST90199890	exposure	Weighted median	5	-0.05239	0.057035	0.358289	-0.16418	0.059395	0.948955	0.848586	1.061194			0.999387558
CAC	ebi-met1400-GCST90199890	4-hydroxyglutamate levels || id:ebi-met1400-GCST90199890	exposure	Weighted mode	5	-0.04937	0.064646	0.487645	-0.17607	0.077341	0.951833	0.838557	1.080411			0.998768904

CAC	ebi-met1400-GCST90199911	Beta-citrylglutamate levels || id:ebi-met1400-GCST90199911	exposure	Inverse variance weighted	5	-0.11604	0.043824	0.008101	-0.20193	-0.03014	0.890441	0.81715	0.970306	0.614581545	0.53974003	0.801502092
CAC	ebi-met1400-GCST90199911	Beta-citrylglutamate levels || id:ebi-met1400-GCST90199911	exposure	Robust adjusted profile score (RAPS)	5	-0.11719	0.046592	0.011891	-0.20851	-0.02588	0.889412	0.811789	0.974457		0.424242331	0.988238216
CAC	ebi-met1400-GCST90199911	Beta-citrylglutamate levels || id:ebi-met1400-GCST90199911	exposure	Contamination mixture method	5	-0.16493	0.086735	0.040309	-0.33493	0.005074	0.847957	0.715391	1.005087			0.712811518
CAC	ebi-met1400-GCST90199911	Beta-citrylglutamate levels || id:ebi-met1400-GCST90199911	exposure	Weighted median	5	-0.08057	0.054962	0.142675	-0.18829	0.027157	0.922591	0.828371	1.027529			0.999387558
CAC	ebi-met1400-GCST90199911	Beta-citrylglutamate levels || id:ebi-met1400-GCST90199911	exposure	MR Egger	5	-0.18796	0.135699	0.26005	-0.45393	0.078015	0.828651	0.635129	1.081138			0.995501177
CAC	ebi-met1400-GCST90199911	Beta-citrylglutamate levels || id:ebi-met1400-GCST90199911	exposure	Weighted mode	5	-0.06665	0.065505	0.366482	-0.19504	0.061743	0.935525	0.822804	1.063689			0.998768904

CAC	ebi-met1400-GCST90199950	2-aminophenol sulfate levels || id:ebi-met1400-GCST90199950	exposure	Inverse variance weighted	5	-0.14768	0.043736	0.000734	-0.23341	-0.06196	0.862704	0.791832	0.939921	0.841459306	0.533851387	0.513600644
CAC	ebi-met1400-GCST90199950	2-aminophenol sulfate levels || id:ebi-met1400-GCST90199950	exposure	Robust adjusted profile score (RAPS)	5	-0.14983	0.046942	0.001414	-0.24184	-0.05783	0.860853	0.785183	0.943815		0.377054248	0.988238216
CAC	ebi-met1400-GCST90199950	2-aminophenol sulfate levels || id:ebi-met1400-GCST90199950	exposure	Weighted median	5	-0.15305	0.054126	0.00469	-0.25914	-0.04696	0.858089	0.771718	0.954126			0.999387558
CAC	ebi-met1400-GCST90199950	2-aminophenol sulfate levels || id:ebi-met1400-GCST90199950	exposure	Contamination mixture method	5	-0.17852	0.05102	0.007293	-0.27852	-0.07852	0.836511	0.756907	0.924488			0.712811518
CAC	ebi-met1400-GCST90199950	2-aminophenol sulfate levels || id:ebi-met1400-GCST90199950	exposure	Weighted mode	5	-0.15702	0.069271	0.086031	-0.2928	-0.02125	0.854683	0.746174	0.978972			0.998768904
CAC	ebi-met1400-GCST90199950	2-aminophenol sulfate levels || id:ebi-met1400-GCST90199950	exposure	MR Egger	5	-0.11929	0.137631	0.449835	-0.38905	0.150462	0.887547	0.6777	1.162371			0.995501177

CAC	ebi-met1400-GCST90199961	Methionine sulfone levels || id:ebi-met1400-GCST90199961	exposure	Inverse variance weighted	5	-0.09426	0.041612	0.023493	-0.17582	-0.01271	0.910043	0.838767	0.987375	0.626337776	0.590498567	0.934078042
CAC	ebi-met1400-GCST90199961	Methionine sulfone levels || id:ebi-met1400-GCST90199961	exposure	Robust adjusted profile score (RAPS)	5	-0.09519	0.0441	0.030892	-0.18163	-0.00875	0.909201	0.833913	0.991286		0.472499772	0.988238216
CAC	ebi-met1400-GCST90199961	Methionine sulfone levels || id:ebi-met1400-GCST90199961	exposure	Contamination mixture method	5	-0.09171	0.07398	0.099079	-0.23671	0.05329	0.91237	0.78922	1.054736			0.712811518
CAC	ebi-met1400-GCST90199961	Methionine sulfone levels || id:ebi-met1400-GCST90199961	exposure	Weighted median	5	-0.05527	0.05084	0.277006	-0.15491	0.04438	0.946232	0.856489	1.04538			0.999387558
CAC	ebi-met1400-GCST90199961	Methionine sulfone levels || id:ebi-met1400-GCST90199961	exposure	Weighted mode	5	-0.04675	0.06739	0.526014	-0.17884	0.085332	0.954323	0.836242	1.089079			0.998768904
CAC	ebi-met1400-GCST90199961	Methionine sulfone levels || id:ebi-met1400-GCST90199961	exposure	MR Egger	5	-0.02831	0.128893	0.840222	-0.28095	0.224316	0.972082	0.755069	1.251467			0.995501177

CAC	ebi-met1400-GCST90199965	Fructosyllysine levels || id:ebi-met1400-GCST90199965	exposure	Inverse variance weighted	5	-0.10509	0.049406	0.033416	-0.20192	-0.00825	0.900244	0.817157	0.991781	0.612001095	0.29071136	0.935072545
CAC	ebi-met1400-GCST90199965	Fructosyllysine levels || id:ebi-met1400-GCST90199965	exposure	Robust adjusted profile score (RAPS)	5	-0.11075	0.053488	0.038406	-0.21558	-0.00591	0.895166	0.806073	0.994107		0.213156253	0.988238216
CAC	ebi-met1400-GCST90199965	Fructosyllysine levels || id:ebi-met1400-GCST90199965	exposure	Contamination mixture method	5	-0.21743	0.091837	0.058464	-0.39743	-0.03743	0.804584	0.672045	0.963262			0.712811518
CAC	ebi-met1400-GCST90199965	Fructosyllysine levels || id:ebi-met1400-GCST90199965	exposure	Weighted median	5	-0.09209	0.058053	0.112686	-0.20587	0.021698	0.912027	0.81394	1.021935			0.999387558
CAC	ebi-met1400-GCST90199965	Fructosyllysine levels || id:ebi-met1400-GCST90199965	exposure	Weighted mode	5	-0.02251	0.069773	0.763114	-0.15927	0.114242	0.977739	0.852768	1.121024			0.998768904
CAC	ebi-met1400-GCST90199965	Fructosyllysine levels || id:ebi-met1400-GCST90199965	exposure	MR Egger	5	-0.01531	0.168101	0.933182	-0.34479	0.31417	0.984809	0.708373	1.369122			0.995501177

CAC	ebi-met1400-GCST90200027	4-hydroxyphenylacetoylcarnitine levels || id:ebi-met1400-GCST90200027	exposure	Weighted median	5	-0.14864	0.054471	0.006356	-0.2554	-0.04188	0.861878	0.774604	0.958985	0.494071602	0.540123624	0.999387558
CAC	ebi-met1400-GCST90200027	4-hydroxyphenylacetoylcarnitine levels || id:ebi-met1400-GCST90200027	exposure	Inverse variance weighted	5	-0.11601	0.045404	0.010614	-0.20501	-0.02702	0.890462	0.814642	0.973339		0.474596776	0.874066694
CAC	ebi-met1400-GCST90200027	4-hydroxyphenylacetoylcarnitine levels || id:ebi-met1400-GCST90200027	exposure	Robust adjusted profile score (RAPS)	5	-0.11966	0.048377	0.013378	-0.21448	-0.02484	0.887221	0.806961	0.975463			0.988238216
CAC	ebi-met1400-GCST90200027	4-hydroxyphenylacetoylcarnitine levels || id:ebi-met1400-GCST90200027	exposure	Contamination mixture method	5	-0.14387	0.05102	0.026434	-0.24387	-0.04387	0.865998	0.783587	0.957076			0.712811518
CAC	ebi-met1400-GCST90200027	4-hydroxyphenylacetoylcarnitine levels || id:ebi-met1400-GCST90200027	exposure	Weighted mode	5	-0.15141	0.071638	0.102083	-0.29182	-0.011	0.859496	0.746903	0.989062			0.998768904
CAC	ebi-met1400-GCST90200027	4-hydroxyphenylacetoylcarnitine levels || id:ebi-met1400-GCST90200027	exposure	MR Egger	5	-0.21962	0.140948	0.217074	-0.49588	0.056636	0.802822	0.609035	1.05827			0.995501177

CAC	ebi-met1400-GCST90200031	Nonanoylcarnitine (C9) levels || id:ebi-met1400-GCST90200031	exposure	Inverse variance weighted	5	0.139535	0.044948	0.001907	0.051436	0.227634	1.149739	1.052781	1.255625	0.735357717	0.504360766	0.763614945
CAC	ebi-met1400-GCST90200031	Nonanoylcarnitine (C9) levels || id:ebi-met1400-GCST90200031	exposure	Robust adjusted profile score (RAPS)	5	0.137984	0.048084	0.004109	0.043739	0.232229	1.147957	1.04471	1.261409		0.364264313	0.988238216
CAC	ebi-met1400-GCST90200031	Nonanoylcarnitine (C9) levels || id:ebi-met1400-GCST90200031	exposure	Contamination mixture method	5	0.144192	0.079082	0.015044	-0.01081	0.299192	1.155106	0.989251	1.348769			0.712811518
CAC	ebi-met1400-GCST90200031	Nonanoylcarnitine (C9) levels || id:ebi-met1400-GCST90200031	exposure	Weighted median	5	0.112647	0.059493	0.058298	-0.00396	0.229254	1.119237	0.996048	1.257661			0.999387558
CAC	ebi-met1400-GCST90200031	Nonanoylcarnitine (C9) levels || id:ebi-met1400-GCST90200031	exposure	Weighted mode	5	0.108082	0.06205	0.156499	-0.01354	0.2297	1.114139	0.986555	1.258223			0.998768904
CAC	ebi-met1400-GCST90200031	Nonanoylcarnitine (C9) levels || id:ebi-met1400-GCST90200031	exposure	MR Egger	5	0.089143	0.143538	0.578564	-0.19219	0.370477	1.093237	0.825149	1.448425			0.995501177

CAC	ebi-met1400-GCST90200039	1-stearoyl-2-linoleoyl-GPE (18:0/18:2) levels || id:ebi-met1400-GCST90200039	exposure	Inverse variance weighted	5	-0.12781	0.044037	0.003704	-0.21412	-0.0415	0.880021	0.80725	0.959353	0.899484107	0.812384031	0.763614945
CAC	ebi-met1400-GCST90200039	1-stearoyl-2-linoleoyl-GPE (18:0/18:2) levels || id:ebi-met1400-GCST90200039	exposure	Robust adjusted profile score (RAPS)	5	-0.12856	0.04713	0.006375	-0.22094	-0.03619	0.879358	0.801766	0.964459		0.668233718	0.988238216
CAC	ebi-met1400-GCST90200039	1-stearoyl-2-linoleoyl-GPE (18:0/18:2) levels || id:ebi-met1400-GCST90200039	exposure	Contamination mixture method	5	-0.12817	0.061224	0.010353	-0.24817	-0.00817	0.879706	0.780229	0.991866			0.712811518
CAC	ebi-met1400-GCST90200039	1-stearoyl-2-linoleoyl-GPE (18:0/18:2) levels || id:ebi-met1400-GCST90200039	exposure	Weighted median	5	-0.12303	0.053465	0.02139	-0.22782	-0.01823	0.884242	0.79627	0.981932			0.999387558
CAC	ebi-met1400-GCST90200039	1-stearoyl-2-linoleoyl-GPE (18:0/18:2) levels || id:ebi-met1400-GCST90200039	exposure	Weighted mode	5	-0.08824	0.064619	0.243843	-0.21489	0.038416	0.915544	0.80663	1.039163			0.998768904
CAC	ebi-met1400-GCST90200039	1-stearoyl-2-linoleoyl-GPE (18:0/18:2) levels || id:ebi-met1400-GCST90200039	exposure	MR Egger	5	-0.11008	0.136452	0.478865	-0.37752	0.157371	0.895766	0.685558	1.17043			0.995501177

CAC	ebi-met1400-GCST90200042	Oleate/vaccenate (18:1) levels || id:ebi-met1400-GCST90200042	exposure	Inverse variance weighted	5	0.084971	0.042925	0.047757	0.000838	0.169104	1.088685	1.000839	1.184243	0.98490638	0.856844544	0.997470883
CAC	ebi-met1400-GCST90200042	Oleate/vaccenate (18:1) levels || id:ebi-met1400-GCST90200042	exposure	Contamination mixture method	5	0.085573	0.081633	0.06005	-0.07443	0.245573	1.089341	0.928275	1.278354		0.722923564	0.712811518
CAC	ebi-met1400-GCST90200042	Oleate/vaccenate (18:1) levels || id:ebi-met1400-GCST90200042	exposure	Robust adjusted profile score (RAPS)	5	0.085331	0.045604	0.061324	-0.00405	0.174715	1.089078	0.995956	1.190907			0.988238216
CAC	ebi-met1400-GCST90200042	Oleate/vaccenate (18:1) levels || id:ebi-met1400-GCST90200042	exposure	Weighted median	5	0.08252	0.051901	0.111845	-0.01921	0.184246	1.086021	0.980978	1.202312			0.999387558
CAC	ebi-met1400-GCST90200042	Oleate/vaccenate (18:1) levels || id:ebi-met1400-GCST90200042	exposure	Weighted mode	5	0.050584	0.063169	0.468131	-0.07323	0.174395	1.051885	0.929389	1.190526			0.998768904
CAC	ebi-met1400-GCST90200042	Oleate/vaccenate (18:1) levels || id:ebi-met1400-GCST90200042	exposure	MR Egger	5	0.082388	0.1329	0.579208	-0.1781	0.342872	1.085877	0.836862	1.408989			0.995501177

CAC	ebi-met1400-GCST90200068	1,2-dilinoleoyl-GPE (18:2/18:2) levels || id:ebi-met1400-GCST90200068	exposure	Inverse variance weighted	5	-0.11966	0.048408	0.013437	-0.21455	-0.02478	0.887218	0.806908	0.97552	0.766119715	0.911761965	0.923524134
CAC	ebi-met1400-GCST90200068	1,2-dilinoleoyl-GPE (18:2/18:2) levels || id:ebi-met1400-GCST90200068	exposure	Robust adjusted profile score (RAPS)	5	-0.12026	0.051706	0.020029	-0.2216	-0.01891	0.886692	0.801235	0.981264		0.830014918	0.988238216
CAC	ebi-met1400-GCST90200068	1,2-dilinoleoyl-GPE (18:2/18:2) levels || id:ebi-met1400-GCST90200068	exposure	Weighted median	5	-0.13082	0.057795	0.023601	-0.2441	-0.01754	0.877374	0.78341	0.982609			0.999387558
CAC	ebi-met1400-GCST90200068	1,2-dilinoleoyl-GPE (18:2/18:2) levels || id:ebi-met1400-GCST90200068	exposure	Contamination mixture method	5	-0.14005	0.058673	0.029546	-0.25505	-0.02505	0.869315	0.774878	0.975261			0.712811518
CAC	ebi-met1400-GCST90200068	1,2-dilinoleoyl-GPE (18:2/18:2) levels || id:ebi-met1400-GCST90200068	exposure	Weighted mode	5	-0.14527	0.069765	0.105745	-0.28201	-0.00853	0.864786	0.754264	0.991503			0.998768904
CAC	ebi-met1400-GCST90200068	1,2-dilinoleoyl-GPE (18:2/18:2) levels || id:ebi-met1400-GCST90200068	exposure	MR Egger	5	-0.16582	0.149781	0.34906	-0.45939	0.127755	0.847203	0.631672	1.136275			0.995501177

CAC	ebi-met1400-GCST90200134	Sphingomyelin (d18:1/25:0, d19:0/24:1, d20:1/23:0, d19:1/24:0) levels || id:ebi-met1400-GCST90200134	exposure	Inverse variance weighted	5	0.096815	0.044011	0.027823	0.010553	0.183076	1.101656	1.010609	1.200906	0.421382863	0.498574152	0.934078042
CAC	ebi-met1400-GCST90200134	Sphingomyelin (d18:1/25:0, d19:0/24:1, d20:1/23:0, d19:1/24:0) levels || id:ebi-met1400-GCST90200134	exposure	Robust adjusted profile score (RAPS)	5	0.097708	0.046652	0.036226	0.006269	0.189147	1.102641	1.006289	1.208219		0.47481578	0.988238216
CAC	ebi-met1400-GCST90200134	Sphingomyelin (d18:1/25:0, d19:0/24:1, d20:1/23:0, d19:1/24:0) levels || id:ebi-met1400-GCST90200134	exposure	Contamination mixture method	5	0.100756	0.079082	0.142783	-0.05424	0.255756	1.106007	0.947201	1.291438			0.712811518
CAC	ebi-met1400-GCST90200134	Sphingomyelin (d18:1/25:0, d19:0/24:1, d20:1/23:0, d19:1/24:0) levels || id:ebi-met1400-GCST90200134	exposure	MR Egger	5	0.216703	0.13634	0.210181	-0.05052	0.48393	1.241975	0.950732	1.622438			0.995501177
CAC	ebi-met1400-GCST90200134	Sphingomyelin (d18:1/25:0, d19:0/24:1, d20:1/23:0, d19:1/24:0) levels || id:ebi-met1400-GCST90200134	exposure	Weighted median	5	0.061583	0.055387	0.266197	-0.04698	0.170142	1.063519	0.95411	1.185473			0.999387558
CAC	ebi-met1400-GCST90200134	Sphingomyelin (d18:1/25:0, d19:0/24:1, d20:1/23:0, d19:1/24:0) levels || id:ebi-met1400-GCST90200134	exposure	Weighted mode	5	0.05225	0.06742	0.481608	-0.07989	0.184392	1.053639	0.923215	1.202487			0.998768904

CAC	ebi-met1400-GCST90200150	Arachidonoylcarnitine (C20:4) levels || id:ebi-met1400-GCST90200150	exposure	Inverse variance weighted	5	0.083369	0.042513	0.049877	4.32E-05	0.166694	1.086943	1.000043	1.181393	0.958774485	0.963747081	0.997470883
CAC	ebi-met1400-GCST90200150	Arachidonoylcarnitine (C20:4) levels || id:ebi-met1400-GCST90200150	exposure	Contamination mixture method	5	0.086419	0.063776	0.056166	-0.03858	0.211419	1.090263	0.962154	1.23543		0.898563004	0.712811518
CAC	ebi-met1400-GCST90200150	Arachidonoylcarnitine (C20:4) levels || id:ebi-met1400-GCST90200150	exposure	Robust adjusted profile score (RAPS)	5	0.083609	0.04523	0.064523	-0.00504	0.17226	1.087204	0.994972	1.187986			0.988238216
CAC	ebi-met1400-GCST90200150	Arachidonoylcarnitine (C20:4) levels || id:ebi-met1400-GCST90200150	exposure	Weighted median	5	0.084663	0.049237	0.085526	-0.01184	0.181167	1.08835	0.988228	1.198616			0.999387558
CAC	ebi-met1400-GCST90200150	Arachidonoylcarnitine (C20:4) levels || id:ebi-met1400-GCST90200150	exposure	Weighted mode	5	0.087428	0.058382	0.208609	-0.027	0.201857	1.091364	0.973361	1.223673			0.998768904
CAC	ebi-met1400-GCST90200150	Arachidonoylcarnitine (C20:4) levels || id:ebi-met1400-GCST90200150	exposure	MR Egger	5	0.090366	0.131727	0.541969	-0.16782	0.34855	1.094575	0.845507	1.417011			0.995501177

CAC	ebi-met1400-GCST90200226	5-hydroxy-2-methylpyridine sulfate levels || id:ebi-met1400-GCST90200226	exposure	Inverse variance weighted	5	-0.10524	0.047927	0.028102	-0.19918	-0.0113	0.900108	0.819404	0.988759	0.900753677	0.460885827	0.934078042
CAC	ebi-met1400-GCST90200226	5-hydroxy-2-methylpyridine sulfate levels || id:ebi-met1400-GCST90200226	exposure	Robust adjusted profile score (RAPS)	5	-0.10995	0.050888	0.030717	-0.2097	-0.01021	0.895874	0.810831	0.989838		0.309132989	0.988238216
CAC	ebi-met1400-GCST90200226	5-hydroxy-2-methylpyridine sulfate levels || id:ebi-met1400-GCST90200226	exposure	Weighted median	5	-0.10618	0.058229	0.068232	-0.22031	0.00795	0.899264	0.802272	1.007982			0.999387558
CAC	ebi-met1400-GCST90200226	5-hydroxy-2-methylpyridine sulfate levels || id:ebi-met1400-GCST90200226	exposure	Contamination mixture method	5	-0.13383	0.063776	0.068398	-0.25883	-0.00883	0.87474	0.771955	0.99121			0.712811518
CAC	ebi-met1400-GCST90200226	5-hydroxy-2-methylpyridine sulfate levels || id:ebi-met1400-GCST90200226	exposure	Weighted mode	5	-0.1326	0.074747	0.150746	-0.2791	0.013908	0.875818	0.756464	1.014005			0.998768904
CAC	ebi-met1400-GCST90200226	5-hydroxy-2-methylpyridine sulfate levels || id:ebi-met1400-GCST90200226	exposure	MR Egger	5	-0.12605	0.162182	0.493705	-0.44392	0.191831	0.881575	0.641515	1.211466			0.995501177

CAC	ebi-met1400-GCST90200282	Picolinate levels || id:ebi-met1400-GCST90200282	exposure	Inverse variance weighted	5	-0.10789	0.047321	0.022608	-0.20064	-0.01514	0.897725	0.818206	0.984972	0.613170611	0.964865455	0.934078042
CAC	ebi-met1400-GCST90200282	Picolinate levels || id:ebi-met1400-GCST90200282	exposure	Contamination mixture method	5	-0.10401	0.058673	0.028482	-0.21901	0.010988	0.901214	0.803312	1.011049		0.966036144	0.712811518
CAC	ebi-met1400-GCST90200282	Picolinate levels || id:ebi-met1400-GCST90200282	exposure	Robust adjusted profile score (RAPS)	5	-0.10814	0.050441	0.032038	-0.207	-0.00928	0.897501	0.813016	0.990765			0.988238216
CAC	ebi-met1400-GCST90200282	Picolinate levels || id:ebi-met1400-GCST90200282	exposure	Weighted median	5	-0.11044	0.056032	0.04872	-0.22026	-0.00062	0.89544	0.802308	0.999382			0.999387558
CAC	ebi-met1400-GCST90200282	Picolinate levels || id:ebi-met1400-GCST90200282	exposure	Weighted mode	5	-0.11447	0.064751	0.151833	-0.24138	0.012447	0.891843	0.785545	1.012525			0.998768904
CAC	ebi-met1400-GCST90200282	Picolinate levels || id:ebi-met1400-GCST90200282	exposure	MR Egger	5	-0.03008	0.146243	0.850207	-0.31672	0.256557	0.970369	0.728538	1.292472			0.995501177

CAC	ebi-met1400-GCST90200285	Eicosenedioate (C20:1-DC) levels || id:ebi-met1400-GCST90200285	exposure	Inverse variance weighted	5	0.091563	0.044503	0.039642	0.004337	0.178789	1.095886	1.004347	1.195769	0.722424233	0.855151885	0.935837484
CAC	ebi-met1400-GCST90200285	Eicosenedioate (C20:1-DC) levels || id:ebi-met1400-GCST90200285	exposure	Robust adjusted profile score (RAPS)	5	0.092108	0.047355	0.05177	-0.00071	0.184925	1.096484	0.999292	1.203128		0.756789669	0.988238216
CAC	ebi-met1400-GCST90200285	Eicosenedioate (C20:1-DC) levels || id:ebi-met1400-GCST90200285	exposure	Contamination mixture method	5	0.094344	0.068878	0.076932	-0.04066	0.229344	1.098938	0.960159	1.257775			0.712811518
CAC	ebi-met1400-GCST90200285	Eicosenedioate (C20:1-DC) levels || id:ebi-met1400-GCST90200285	exposure	Weighted median	5	0.072228	0.051232	0.158589	-0.02819	0.172642	1.074901	0.972208	1.188441			0.999387558
CAC	ebi-met1400-GCST90200285	Eicosenedioate (C20:1-DC) levels || id:ebi-met1400-GCST90200285	exposure	Weighted mode	5	0.049704	0.07054	0.519906	-0.08855	0.187962	1.05096	0.915254	1.206788			0.998768904
CAC	ebi-met1400-GCST90200285	Eicosenedioate (C20:1-DC) levels || id:ebi-met1400-GCST90200285	exposure	MR Egger	5	0.040703	0.137721	0.78685	-0.22923	0.310637	1.041543	0.795145	1.364294			0.995501177

CAC	ebi-met1400-GCST90200297	Ursodeoxycholate levels || id:ebi-met1400-GCST90200297	exposure	Inverse variance weighted	5	-0.14204	0.051802	0.006109	-0.24357	-0.0405	0.86759	0.783826	0.960306	0.196162735	0.401701553	0.763614945
CAC	ebi-met1400-GCST90200297	Ursodeoxycholate levels || id:ebi-met1400-GCST90200297	exposure	Robust adjusted profile score (RAPS)	5	-0.13816	0.054658	0.011481	-0.24529	-0.03103	0.87096	0.782478	0.969447		0.732137754	0.988238216
CAC	ebi-met1400-GCST90200297	Ursodeoxycholate levels || id:ebi-met1400-GCST90200297	exposure	Contamination mixture method	5	-0.13868	0.135204	0.078756	-0.40368	0.126323	0.870509	0.66786	1.134648			0.712811518
CAC	ebi-met1400-GCST90200297	Ursodeoxycholate levels || id:ebi-met1400-GCST90200297	exposure	MR Egger	5	-0.39311	0.160097	0.091234	-0.7069	-0.07932	0.674952	0.493169	0.923742			0.995501177
CAC	ebi-met1400-GCST90200297	Ursodeoxycholate levels || id:ebi-met1400-GCST90200297	exposure	Weighted median	5	-0.10119	0.064883	0.118845	-0.22836	0.025976	0.903758	0.795835	1.026317			0.999387558
CAC	ebi-met1400-GCST90200297	Ursodeoxycholate levels || id:ebi-met1400-GCST90200297	exposure	Weighted mode	5	-0.08102	0.079735	0.367023	-0.2373	0.075258	0.922172	0.788751	1.078162			0.998768904

CAC	ebi-met1400-GCST90200310	3-aminoisobutyrate levels || id:ebi-met1400-GCST90200310	exposure	Inverse variance weighted	5	0.124675	0.042497	0.003349	0.04138	0.207969	1.13278	1.042248	1.231175	0.837639251	0.420208309	0.763614945
CAC	ebi-met1400-GCST90200310	3-aminoisobutyrate levels || id:ebi-met1400-GCST90200310	exposure	Robust adjusted profile score (RAPS)	5	0.127052	0.045438	0.005171	0.037994	0.21611	1.135476	1.038725	1.24124		0.280106283	0.988238216
CAC	ebi-met1400-GCST90200310	3-aminoisobutyrate levels || id:ebi-met1400-GCST90200310	exposure	Weighted median	5	0.131033	0.054585	0.016372	0.024045	0.23802	1.140005	1.024337	1.268735			0.999387558
CAC	ebi-met1400-GCST90200310	3-aminoisobutyrate levels || id:ebi-met1400-GCST90200310	exposure	Contamination mixture method	5	0.151466	0.056122	0.02642	0.041466	0.261466	1.163538	1.042337	1.298832			0.712811518
CAC	ebi-met1400-GCST90200310	3-aminoisobutyrate levels || id:ebi-met1400-GCST90200310	exposure	Weighted mode	5	0.137628	0.065399	0.103136	0.009446	0.26581	1.147548	1.009491	1.304487			0.998768904
CAC	ebi-met1400-GCST90200310	3-aminoisobutyrate levels || id:ebi-met1400-GCST90200310	exposure	MR Egger	5	0.09323	0.14878	0.575344	-0.19838	0.384839	1.097715	0.820059	1.469378			0.995501177

CAC	ebi-met1400-GCST90200326	Gamma-glutamylleucine levels || id:ebi-met1400-GCST90200326	exposure	Inverse variance weighted	5	-0.09154	0.043993	0.037446	-0.17777	-0.00532	0.91252	0.837133	0.994696	0.746636712	0.805026575	0.935072545
CAC	ebi-met1400-GCST90200326	Gamma-glutamylleucine levels || id:ebi-met1400-GCST90200326	exposure	Robust adjusted profile score (RAPS)	5	-0.0921	0.0467	0.048598	-0.18363	-0.00057	0.912016	0.832244	0.999435		0.683313499	0.988238216
CAC	ebi-met1400-GCST90200326	Gamma-glutamylleucine levels || id:ebi-met1400-GCST90200326	exposure	Contamination mixture method	5	-0.08722	0.066327	0.06679	-0.21722	0.042776	0.916471	0.804749	1.043704			0.712811518
CAC	ebi-met1400-GCST90200326	Gamma-glutamylleucine levels || id:ebi-met1400-GCST90200326	exposure	Weighted median	5	-0.07764	0.054689	0.155708	-0.18483	0.029551	0.925297	0.831244	1.029992			0.999387558
CAC	ebi-met1400-GCST90200326	Gamma-glutamylleucine levels || id:ebi-met1400-GCST90200326	exposure	Weighted mode	5	-0.04995	0.071037	0.520724	-0.18918	0.089282	0.951276	0.827634	1.093389			0.998768904
CAC	ebi-met1400-GCST90200326	Gamma-glutamylleucine levels || id:ebi-met1400-GCST90200326	exposure	MR Egger	5	-0.04576	0.136549	0.759596	-0.3134	0.221874	0.95527	0.73096	1.248415			0.995501177

CAC	ebi-met1400-GCST90200332	1-palmitoyl-2-linoleoyl-GPI (16:0/18:2) levels || id:ebi-met1400-GCST90200332	exposure	Robust adjusted profile score (RAPS)	5	-0.12687	0.054199	0.019246	-0.2331	-0.02064	0.880852	0.792078	0.979576	0.158874738	0.195641772	0.988238216
CAC	ebi-met1400-GCST90200332	1-palmitoyl-2-linoleoyl-GPI (16:0/18:2) levels || id:ebi-met1400-GCST90200332	exposure	Inverse variance weighted	5	-0.12118	0.054444	0.02603	-0.22789	-0.01447	0.885874	0.796211	0.985635		0.463623246	0.934078042
CAC	ebi-met1400-GCST90200332	1-palmitoyl-2-linoleoyl-GPI (16:0/18:2) levels || id:ebi-met1400-GCST90200332	exposure	MR Egger	5	-0.36309	0.136989	0.076963	-0.63159	-0.09459	0.695526	0.531748	0.909748			0.995501177
CAC	ebi-met1400-GCST90200332	1-palmitoyl-2-linoleoyl-GPI (16:0/18:2) levels || id:ebi-met1400-GCST90200332	exposure	Contamination mixture method	5	-0.07067	0.104592	0.13915	-0.27567	0.134331	0.93177	0.759064	1.143771			0.712811518
CAC	ebi-met1400-GCST90200332	1-palmitoyl-2-linoleoyl-GPI (16:0/18:2) levels || id:ebi-met1400-GCST90200332	exposure	Weighted median	5	-0.08255	0.057169	0.148725	-0.19461	0.029496	0.920761	0.823159	1.029936			0.999387558
CAC	ebi-met1400-GCST90200332	1-palmitoyl-2-linoleoyl-GPI (16:0/18:2) levels || id:ebi-met1400-GCST90200332	exposure	Weighted mode	5	-0.05933	0.070622	0.448113	-0.19775	0.079087	0.942393	0.820573	1.082298			0.998768904

CAC	ebi-met1400-GCST90200342	Glycochenodeoxycholate levels || id:ebi-met1400-GCST90200342	exposure	Inverse variance weighted	5	-0.10838	0.044175	0.014149	-0.19496	-0.0218	0.897285	0.822864	0.978438	0.272312935	0.44858795	0.923524134
CAC	ebi-met1400-GCST90200342	Glycochenodeoxycholate levels || id:ebi-met1400-GCST90200342	exposure	Robust adjusted profile score (RAPS)	5	-0.10342	0.046682	0.026729	-0.19492	-0.01192	0.901748	0.822903	0.988146		0.593926707	0.988238216
CAC	ebi-met1400-GCST90200342	Glycochenodeoxycholate levels || id:ebi-met1400-GCST90200342	exposure	Weighted median	5	-0.08762	0.055936	0.117268	-0.19725	0.02202	0.916113	0.820985	1.022264			0.999387558
CAC	ebi-met1400-GCST90200342	Glycochenodeoxycholate levels || id:ebi-met1400-GCST90200342	exposure	Contamination mixture method	5	-0.11234	0.117347	0.118098	-0.34234	0.117657	0.893737	0.710104	1.124858			0.712811518
CAC	ebi-met1400-GCST90200342	Glycochenodeoxycholate levels || id:ebi-met1400-GCST90200342	exposure	MR Egger	5	-0.28244	0.137081	0.131442	-0.55112	-0.01377	0.753938	0.576302	0.986328			0.995501177
CAC	ebi-met1400-GCST90200342	Glycochenodeoxycholate levels || id:ebi-met1400-GCST90200342	exposure	Weighted mode	5	-0.08774	0.06347	0.239015	-0.21214	0.036659	0.915997	0.808849	1.037339			0.998768904

CAC	ebi-met1400-GCST90200345	S-adenosylhomocysteine (SAH) levels || id:ebi-met1400-GCST90200345	exposure	Inverse variance weighted	5	-0.12334	0.047398	0.00926	-0.21624	-0.03044	0.883961	0.80554	0.970015	0.699991325	0.7302011	0.81024884
CAC	ebi-met1400-GCST90200345	S-adenosylhomocysteine (SAH) levels || id:ebi-met1400-GCST90200345	exposure	Robust adjusted profile score (RAPS)	5	-0.12415	0.050494	0.013941	-0.22312	-0.02519	0.883244	0.800017	0.975129		0.604059748	0.988238216
CAC	ebi-met1400-GCST90200345	S-adenosylhomocysteine (SAH) levels || id:ebi-met1400-GCST90200345	exposure	Contamination mixture method	5	-0.16453	0.084184	0.022974	-0.32953	0.000474	0.848296	0.719265	1.000474			0.712811518
CAC	ebi-met1400-GCST90200345	S-adenosylhomocysteine (SAH) levels || id:ebi-met1400-GCST90200345	exposure	Weighted median	5	-0.1215	0.061104	0.046765	-0.24127	-0.00174	0.88559	0.785633	0.998265			0.999387558
CAC	ebi-met1400-GCST90200345	S-adenosylhomocysteine (SAH) levels || id:ebi-met1400-GCST90200345	exposure	MR Egger	5	-0.18245	0.147179	0.303233	-0.47092	0.106018	0.833225	0.624426	1.111842			0.995501177
CAC	ebi-met1400-GCST90200345	S-adenosylhomocysteine (SAH) levels || id:ebi-met1400-GCST90200345	exposure	Weighted mode	5	-0.05696	0.069623	0.459238	-0.19342	0.079504	0.944634	0.824136	1.082749			0.998768904

CAC	ebi-met1400-GCST90200358	Arachidonate (20:4n6) levels || id:ebi-met1400-GCST90200358	exposure	Inverse variance weighted	5	0.090924	0.043422	0.036266	0.005816	0.176031	1.095185	1.005833	1.192475	0.880518206	0.915071367	0.935072545
CAC	ebi-met1400-GCST90200358	Arachidonate (20:4n6) levels || id:ebi-met1400-GCST90200358	exposure	Robust adjusted profile score (RAPS)	5	0.091339	0.046235	0.048206	0.000719	0.181959	1.09564	1.000719	1.199565		0.816209652	0.988238216
CAC	ebi-met1400-GCST90200358	Arachidonate (20:4n6) levels || id:ebi-met1400-GCST90200358	exposure	Contamination mixture method	5	0.091019	0.071429	0.056785	-0.04898	0.231019	1.09529	0.9522	1.259884			0.712811518
CAC	ebi-met1400-GCST90200358	Arachidonate (20:4n6) levels || id:ebi-met1400-GCST90200358	exposure	Weighted median	5	0.094661	0.05084	0.062613	-0.00499	0.194308	1.099286	0.995026	1.21447			0.999387558
CAC	ebi-met1400-GCST90200358	Arachidonate (20:4n6) levels || id:ebi-met1400-GCST90200358	exposure	Weighted mode	5	0.09813	0.065877	0.210574	-0.03099	0.22725	1.103107	0.969486	1.255144			0.998768904
CAC	ebi-met1400-GCST90200358	Arachidonate (20:4n6) levels || id:ebi-met1400-GCST90200358	exposure	MR Egger	5	0.111732	0.134469	0.466975	-0.15183	0.375291	1.118213	0.859136	1.455415			0.995501177

CAC	ebi-met1400-GCST90200386	Deoxycholate levels || id:ebi-met1400-GCST90200386	exposure	Inverse variance weighted	5	-0.1286	0.045877	0.005059	-0.21852	-0.03869	0.879321	0.803705	0.962052	0.675994683	0.931413208	0.763614945
CAC	ebi-met1400-GCST90200386	Deoxycholate levels || id:ebi-met1400-GCST90200386	exposure	Robust adjusted profile score (RAPS)	5	-0.12907	0.049136	0.008621	-0.22537	-0.03276	0.878914	0.798217	0.96777		0.887510219	0.988238216
CAC	ebi-met1400-GCST90200386	Deoxycholate levels || id:ebi-met1400-GCST90200386	exposure	Contamination mixture method	5	-0.1276	0.05102	0.015182	-0.2276	-0.0276	0.880206	0.796444	0.972778			0.712811518
CAC	ebi-met1400-GCST90200386	Deoxycholate levels || id:ebi-met1400-GCST90200386	exposure	Weighted median	5	-0.1163	0.05689	0.040928	-0.2278	-0.00479	0.890209	0.79628	0.995218			0.999387558
CAC	ebi-met1400-GCST90200386	Deoxycholate levels || id:ebi-met1400-GCST90200386	exposure	Weighted mode	5	-0.13729	0.070493	0.123301	-0.27546	0.000876	0.871717	0.759225	1.000877			0.998768904
CAC	ebi-met1400-GCST90200386	Deoxycholate levels || id:ebi-met1400-GCST90200386	exposure	MR Egger	5	-0.19051	0.141836	0.271786	-0.46851	0.087487	0.826536	0.625933	1.091428			0.995501177

CAC	ebi-met1400-GCST90200467	X-11852 levels || id:ebi-met1400-GCST90200467	exposure	Inverse variance weighted	5	-0.09602	0.04707	0.041355	-0.18828	-0.00376	0.908445	0.828385	0.996244	0.590221686	0.464285526	0.949129987
CAC	ebi-met1400-GCST90200467	X-11852 levels || id:ebi-met1400-GCST90200467	exposure	Robust adjusted profile score (RAPS)	5	-0.09725	0.049805	0.050866	-0.19487	0.000368	0.907329	0.822943	1.000368		0.361161786	0.988238216
CAC	ebi-met1400-GCST90200467	X-11852 levels || id:ebi-met1400-GCST90200467	exposure	Weighted median	5	-0.09944	0.057623	0.08441	-0.21238	0.013504	0.905347	0.808659	1.013595			0.999387558
CAC	ebi-met1400-GCST90200467	X-11852 levels || id:ebi-met1400-GCST90200467	exposure	Contamination mixture method	5	-0.11882	0.084184	0.112089	-0.28382	0.046183	0.88797	0.752904	1.047266			0.712811518
CAC	ebi-met1400-GCST90200467	X-11852 levels || id:ebi-met1400-GCST90200467	exposure	Weighted mode	5	-0.09222	0.070626	0.261687	-0.23064	0.046211	0.911909	0.794024	1.047295			0.998768904
CAC	ebi-met1400-GCST90200467	X-11852 levels || id:ebi-met1400-GCST90200467	exposure	MR Egger	5	-0.18174	0.150706	0.31427	-0.47713	0.11364	0.833814	0.620562	1.120348			0.995501177

CAC	ebi-met1400-GCST90200493	X-12013 levels || id:ebi-met1400-GCST90200493	exposure	Inverse variance weighted	5	0.10178	0.051579	0.048464	0.000685	0.202875	1.10714	1.000685	1.22492	0.822745098	0.875677819	0.997470883
CAC	ebi-met1400-GCST90200493	X-12013 levels || id:ebi-met1400-GCST90200493	exposure	Robust adjusted profile score (RAPS)	5	0.102143	0.054733	0.062013	-0.00513	0.209419	1.107541	0.994879	1.232962		0.763821135	0.988238216
CAC	ebi-met1400-GCST90200493	X-12013 levels || id:ebi-met1400-GCST90200493	exposure	Contamination mixture method	5	0.097363	0.086735	0.06462	-0.07264	0.267363	1.10226	0.929938	1.306514			0.712811518
CAC	ebi-met1400-GCST90200493	X-12013 levels || id:ebi-met1400-GCST90200493	exposure	Weighted median	5	0.092109	0.061025	0.13121	-0.0275	0.211718	1.096484	0.972874	1.2358			0.999387558
CAC	ebi-met1400-GCST90200493	X-12013 levels || id:ebi-met1400-GCST90200493	exposure	Weighted mode	5	0.075347	0.072875	0.359581	-0.06749	0.218182	1.078258	0.93474	1.243813			0.998768904
CAC	ebi-met1400-GCST90200493	X-12013 levels || id:ebi-met1400-GCST90200493	exposure	MR Egger	5	0.06527	0.158093	0.707445	-0.24459	0.375131	1.067447	0.783024	1.455182			0.995501177

CAC	ebi-met1400-GCST90200507	X-12822 levels || id:ebi-met1400-GCST90200507	exposure	Inverse variance weighted	5	-0.11995	0.044113	0.006545	-0.20641	-0.03349	0.886965	0.813499	0.967067	0.80558387	0.995845003	0.763614945
CAC	ebi-met1400-GCST90200507	X-12822 levels || id:ebi-met1400-GCST90200507	exposure	Contamination mixture method	5	-0.11596	0.045918	0.008865	-0.20596	-0.02596	0.89051	0.813864	0.974373		0.989862699	0.712811518
CAC	ebi-met1400-GCST90200507	X-12822 levels || id:ebi-met1400-GCST90200507	exposure	Robust adjusted profile score (RAPS)	5	-0.12006	0.047257	0.011069	-0.21268	-0.02743	0.886869	0.808413	0.972939			0.988238216
CAC	ebi-met1400-GCST90200507	X-12822 levels || id:ebi-met1400-GCST90200507	exposure	Weighted median	5	-0.12433	0.052427	0.017722	-0.22708	-0.02157	0.883092	0.796854	0.978663			0.999387558
CAC	ebi-met1400-GCST90200507	X-12822 levels || id:ebi-met1400-GCST90200507	exposure	Weighted mode	5	-0.13087	0.069051	0.13096	-0.26621	0.004474	0.877335	0.766281	1.004484			0.998768904
CAC	ebi-met1400-GCST90200507	X-12822 levels || id:ebi-met1400-GCST90200507	exposure	MR Egger	5	-0.15475	0.136809	0.340259	-0.42289	0.1134	0.856633	0.65515	1.12008			0.995501177

CAC	ebi-met1400-GCST90200515	X-12849 levels || id:ebi-met1400-GCST90200515	exposure	Inverse variance weighted	5	-0.12426	0.046493	0.007525	-0.21539	-0.03314	0.883148	0.806228	0.967408	0.822824649	0.541757032	0.801502092
CAC	ebi-met1400-GCST90200515	X-12849 levels || id:ebi-met1400-GCST90200515	exposure	Robust adjusted profile score (RAPS)	5	-0.12473	0.049407	0.011583	-0.22157	-0.0279	0.882733	0.80126	0.97249		0.386038917	0.988238216
CAC	ebi-met1400-GCST90200515	X-12849 levels || id:ebi-met1400-GCST90200515	exposure	Contamination mixture method	5	-0.14597	0.102041	0.04256	-0.34597	0.054032	0.864185	0.707535	1.055518			0.712811518
CAC	ebi-met1400-GCST90200515	X-12849 levels || id:ebi-met1400-GCST90200515	exposure	Weighted median	5	-0.11535	0.057555	0.045052	-0.22816	-0.00254	0.891054	0.795997	0.997461			0.999387558
CAC	ebi-met1400-GCST90200515	X-12849 levels || id:ebi-met1400-GCST90200515	exposure	Weighted mode	5	-0.11551	0.069145	0.170129	-0.25103	0.020013	0.890911	0.777996	1.020215			0.998768904
CAC	ebi-met1400-GCST90200515	X-12849 levels || id:ebi-met1400-GCST90200515	exposure	MR Egger	5	-0.15774	0.144838	0.355792	-0.44162	0.126146	0.854075	0.642995	1.134448			0.995501177

CAC	ebi-met1400-GCST90200547	X-17654 levels || id:ebi-met1400-GCST90200547	exposure	Inverse variance weighted	5	-0.10692	0.044591	0.016492	-0.19432	-0.01952	0.898594	0.823392	0.980665	0.376177963	0.603894761	0.923524134
CAC	ebi-met1400-GCST90200547	X-17654 levels || id:ebi-met1400-GCST90200547	exposure	Robust adjusted profile score (RAPS)	5	-0.10814	0.04737	0.022437	-0.20099	-0.0153	0.897501	0.817923	0.984821		0.646737005	0.988238216
CAC	ebi-met1400-GCST90200547	X-17654 levels || id:ebi-met1400-GCST90200547	exposure	Contamination mixture method	5	-0.13055	0.089286	0.050736	-0.30555	0.044455	0.877617	0.736722	1.045458			0.712811518
CAC	ebi-met1400-GCST90200547	X-17654 levels || id:ebi-met1400-GCST90200547	exposure	Weighted median	5	-0.08723	0.055586	0.116585	-0.19618	0.021719	0.916467	0.821866	1.021957			0.999387558
CAC	ebi-met1400-GCST90200547	X-17654 levels || id:ebi-met1400-GCST90200547	exposure	MR Egger	5	-0.24237	0.138077	0.177465	-0.513	0.028257	0.784763	0.598695	1.02866			0.995501177
CAC	ebi-met1400-GCST90200547	X-17654 levels || id:ebi-met1400-GCST90200547	exposure	Weighted mode	5	-0.08131	0.064025	0.272924	-0.2068	0.044175	0.921905	0.813181	1.045165			0.998768904
CAC	ebi-met1400-	X-17653 levels || id:ebi-met1400-GCST90200553	exposure	Robust adjusted	5	-0.108	0.047194	0.022113	-0.2005	-0.0155	0.897627	0.81832	0.98462	0.167685165	0.396685593	0.988238216

	GCST90200553			profile score (RAPS)												
CAC	ebi-met1400-GCST90200553	X-17653 levels || id:ebi-met1400-GCST90200553	exposure	Inverse variance weighted	5	-0.10099	0.044855	0.024352	-0.18891	-0.01308	0.90394	0.827863	0.987009		0.852626756	0.934078042
CAC	ebi-met1400-GCST90200553	X-17653 levels || id:ebi-met1400-GCST90200553	exposure	Contamination mixture method	5	-0.13026	0.061224	0.031198	-0.25026	-0.01026	0.877865	0.778597	0.98979			0.712811518
CAC	ebi-met1400-GCST90200553	X-17653 levels || id:ebi-met1400-GCST90200553	exposure	Weighted median	5	-0.10998	0.056011	0.049577	-0.21977	-0.0002	0.895849	0.802707	0.999798			0.999387558
CAC	ebi-met1400-GCST90200553	X-17653 levels || id:ebi-met1400-GCST90200553	exposure	MR Egger	5	-0.33743	0.137868	0.091883	-0.60765	-0.06721	0.7136	0.544628	0.934998			0.995501177
CAC	ebi-met1400-GCST90200553	X-17653 levels || id:ebi-met1400-GCST90200553	exposure	Weighted mode	5	-0.10935	0.068693	0.186626	-0.24399	0.025287	0.896416	0.783497	1.02561			0.998768904
CAC	ebi-met1400-GCST90200569	X-18913 levels || id:ebi-met1400-GCST90200569	exposure	Inverse variance weighted	5	-0.0878	0.044066	0.046323	-0.17417	-0.00143	0.915945	0.840156	0.998571	0.731341785	0.992254028	0.997470883

CAC	ebi-met1400-GCST90200569	X-18913 levels || id:ebi-met1400-GCST90200569	exposure	Contamination mixture method	5	-0.09266	0.063776	0.046772	-0.21766	0.032336	0.9115	0.804396	1.032865		0.989612119	0.712811518
CAC	ebi-met1400-GCST90200569	X-18913 levels || id:ebi-met1400-GCST90200569	exposure	Robust adjusted profile score (RAPS)	5	-0.08788	0.0469	0.060953	-0.17981	0.004041	0.915869	0.835433	1.004049			0.988238216
CAC	ebi-met1400-GCST90200569	X-18913 levels || id:ebi-met1400-GCST90200569	exposure	Weighted median	5	-0.07681	0.052486	0.143367	-0.17968	0.026066	0.92607	0.835539	1.026409			0.999387558
CAC	ebi-met1400-GCST90200569	X-18913 levels || id:ebi-met1400-GCST90200569	exposure	Weighted mode	5	-0.07484	0.064855	0.312796	-0.20195	0.05228	0.927897	0.817136	1.053671			0.998768904
CAC	ebi-met1400-GCST90200569	X-18913 levels || id:ebi-met1400-GCST90200569	exposure	MR Egger	5	-0.03912	0.136464	0.793007	-0.30659	0.228345	0.961632	0.735951	1.256519			0.995501177
CAC	ebi-met1400-GCST90200597	X-22834 levels || id:ebi-met1400-GCST90200597	exposure	Inverse variance weighted	5	-0.13525	0.049723	0.006526	-0.23271	-0.03779	0.873497	0.792385	0.962911	0.844379345	0.872036815	0.763614945

CAC	ebi-met1400-GCST90200597	X-22834 levels || id:ebi-met1400-GCST90200597	exposure	Robust adjusted profile score (RAPS)	5	-0.13596	0.053168	0.010551	-0.24017	-0.03175	0.872875	0.786492	0.968745		0.755194701	0.988238216
CAC	ebi-met1400-GCST90200597	X-22834 levels || id:ebi-met1400-GCST90200597	exposure	Contamination mixture method	5	-0.13907	0.068878	0.018302	-0.27407	-0.00407	0.87017	0.760282	0.995942			0.712811518
CAC	ebi-met1400-GCST90200597	X-22834 levels || id:ebi-met1400-GCST90200597	exposure	Weighted median	5	-0.12451	0.059411	0.036109	-0.24095	-0.00806	0.882931	0.785878	0.991971			0.999387558
CAC	ebi-met1400-GCST90200597	X-22834 levels || id:ebi-met1400-GCST90200597	exposure	Weighted mode	5	-0.12476	0.075363	0.173175	-0.27247	0.022952	0.882709	0.761495	1.023217			0.998768904
CAC	ebi-met1400-GCST90200597	X-22834 levels || id:ebi-met1400-GCST90200597	exposure	MR Egger	5	-0.16646	0.154186	0.359374	-0.46866	0.135743	0.846656	0.625837	1.145387			0.995501177
CAC	ebi-met1400-GCST90200602	X-22520 levels || id:ebi-met1400-GCST90200602	exposure	Inverse variance weighted	5	-0.11556	0.05414	0.032802	-0.22168	-0.00945	0.890866	0.801175	0.990597	0.96351828	0.588933256	0.935072545

CAC	ebi-met1400-GCST90200602	X-22520 levels || id:ebi-met1400-GCST90200602	exposure	Robust adjusted profile score (RAPS)	5	-0.11685	0.057526	0.042226	-0.2296	-0.0041	0.889717	0.794849	0.995908		0.421142829	0.988238216
CAC	ebi-met1400-GCST90200602	X-22520 levels || id:ebi-met1400-GCST90200602	exposure	Weighted median	5	-0.13196	0.065892	0.045209	-0.26111	-0.00281	0.876374	0.770196	0.99719			0.999387558
CAC	ebi-met1400-GCST90200602	X-22520 levels || id:ebi-met1400-GCST90200602	exposure	Contamination mixture method	5	-0.11621	0.112245	0.089532	-0.33621	0.103794	0.890292	0.714476	1.109372			0.712811518
CAC	ebi-met1400-GCST90200602	X-22520 levels || id:ebi-met1400-GCST90200602	exposure	Weighted mode	5	-0.10546	0.077523	0.245316	-0.25741	0.046482	0.899908	0.773053	1.047579			0.998768904
CAC	ebi-met1400-GCST90200602	X-22520 levels || id:ebi-met1400-GCST90200602	exposure	MR Egger	5	-0.12338	0.166415	0.512228	-0.44955	0.202797	0.883932	0.637916	1.224824			0.995501177
CAC	ebi-met1400-GCST90200606	X-22509 levels || id:ebi-met1400-GCST90200606	exposure	Inverse variance weighted	5	0.133287	0.053902	0.013407	0.027639	0.238935	1.142578	1.028025	1.269896	0.776347439	0.814397189	0.923524134
CAC	ebi-met1400-	X-22509 levels || id:ebi-met1400-GCST90200606	exposure	Robust adjusted	5	0.134027	0.057399	0.019543	0.021525	0.246529	1.143424	1.021758	1.279577		0.688700958	0.988238216

	GCST90200606			profile score (RAPS)												
CAC	ebi-met1400-GCST90200606	X-22509 levels || id:ebi-met1400-GCST90200606	exposure	Contamination mixture method	5	0.131722	0.091837	0.026069	-0.04828	0.311722	1.140791	0.952869	1.365775			0.712811518
CAC	ebi-met1400-GCST90200606	X-22509 levels || id:ebi-met1400-GCST90200606	exposure	Weighted median	5	0.097203	0.067206	0.148081	-0.03452	0.228926	1.102084	0.966068	1.257249			0.999387558
CAC	ebi-met1400-GCST90200606	X-22509 levels || id:ebi-met1400-GCST90200606	exposure	Weighted mode	5	0.095659	0.077633	0.285354	-0.0565	0.24782	1.100384	0.945065	1.28123			0.998768904
CAC	ebi-met1400-GCST90200606	X-22509 levels || id:ebi-met1400-GCST90200606	exposure	MR Egger	5	0.182013	0.16582	0.352559	-0.14299	0.50702	1.19963	0.86676	1.660336			0.995501177
CAC	ebi-met1400-GCST90200693	Glycochenodeoxycholate glucuronide (1) levels || id:ebi-met1400-GCST90200693	exposure	Inverse variance weighted	5	-0.11885	0.043544	0.006345	-0.20419	-0.0335	0.887942	0.815304	0.967052	0.760630386	0.401448835	0.763614945
CAC	ebi-met1400-GCST90200693	Glycochenodeoxycholate glucuronide (1) levels || id:ebi-met1400-GCST90200693	exposure	Robust adjusted profile	5	-0.116	0.046118	0.011893	-0.20639	-0.02561	0.890476	0.813516	0.974716		0.273643939	0.988238216

				score (RAPS)												
CAC	ebi-met1400-GCST90200693	Glycochenodeoxycholate glucuronide (1) levels || id:ebi-met1400-GCST90200693	exposure	Weighted median	5	-0.08845	0.05786	0.126331	-0.20186	0.024953	0.915346	0.81721	1.025267			0.999387558
CAC	ebi-met1400-GCST90200693	Glycochenodeoxycholate glucuronide (1) levels || id:ebi-met1400-GCST90200693	exposure	Contamination mixture method	5	-0.18699	0.107143	0.227622	-0.39699	0.023008	0.82945	0.672339	1.023274			0.712811518
CAC	ebi-met1400-GCST90200693	Glycochenodeoxycholate glucuronide (1) levels || id:ebi-met1400-GCST90200693	exposure	MR Egger	5	-0.16716	0.152991	0.354456	-0.46702	0.132704	0.846066	0.626868	1.141912			0.995501177
CAC	ebi-met1400-GCST90200693	Glycochenodeoxycholate glucuronide (1) levels || id:ebi-met1400-GCST90200693	exposure	Weighted mode	5	-0.04781	0.089401	0.621122	-0.22304	0.127413	0.953312	0.800084	1.135886			0.998768904
CAC	ebi-met1400-GCST90200723	S-adenosylhomocysteine (SAH) to 5-methyluridine (ribothymidine) ratio || id:ebi-met1400-GCST90200723	exposure	Inverse variance weighted	5	-0.11828	0.049311	0.016455	-0.21493	-0.02163	0.888446	0.806597	0.978601	0.553783989	0.553189566	0.923524134
CAC	ebi-met1400-GCST90200723	S-adenosylhomocysteine (SAH) to 5-methyluridine (ribothymidine) ratio || id:ebi-met1400-GCST90200723	exposure	Robust adjusted profile score (RAPS)	5	-0.11947	0.052332	0.022432	-0.22204	-0.0169	0.887389	0.800881	0.98324		0.45995464	0.988238216

CAC	ebi-met1400-GCST90200723	S-adenosylhomocysteine (SAH) to 5-methyluridine (ribothymidine) ratio || id:ebi-met1400-GCST90200723	exposure	Contamination mixture method	5	-0.18808	0.07398	0.024106	-0.33308	-0.04308	0.828549	0.716713	0.957835			0.712811518
CAC	ebi-met1400-GCST90200723	S-adenosylhomocysteine (SAH) to 5-methyluridine (ribothymidine) ratio || id:ebi-met1400-GCST90200723	exposure	Weighted median	5	-0.13237	0.062998	0.035632	-0.25584	-0.00889	0.876021	0.774265	0.99115			0.999387558
CAC	ebi-met1400-GCST90200723	S-adenosylhomocysteine (SAH) to 5-methyluridine (ribothymidine) ratio || id:ebi-met1400-GCST90200723	exposure	MR Egger	5	-0.21469	0.153195	0.255632	-0.51495	0.085575	0.806794	0.597531	1.089343			0.995501177
CAC	ebi-met1400-GCST90200723	S-adenosylhomocysteine (SAH) to 5-methyluridine (ribothymidine) ratio || id:ebi-met1400-GCST90200723	exposure	Weighted mode	5	-0.023	0.079221	0.786	-0.17827	0.132273	0.977263	0.836714	1.14142			0.998768904
CAC	ebi-met1400-GCST90200749	Arachidonate (20:4n6) to pyruvate ratio || id:ebi-met1400-GCST90200749	exposure	Inverse variance weighted	5	0.106515	0.043515	0.014373	0.021226	0.191804	1.112395	1.021453	1.211433	0.960421783	0.998063993	0.923524134
CAC	ebi-met1400-GCST90200749	Arachidonate (20:4n6) to pyruvate ratio || id:ebi-met1400-GCST90200749	exposure	Contamination mixture method	5	0.109827	0.043367	0.015144	0.024827	0.194827	1.116085	1.025137	1.2151		0.988782209	0.712811518
CAC	ebi-met1400-	Arachidonate (20:4n6) to pyruvate ratio || id:ebi-met1400-GCST90200749	exposure	Robust adjusted profile	5	0.106544	0.046484	0.021903	0.015435	0.197653	1.112427	1.015555	1.21854			0.988238216

	GCST90200749			score (RAPS)												
CAC	ebi-met1400-GCST90200749	Arachidonate (20:4n6) to pyruvate ratio || id:ebi-met1400-GCST90200749	exposure	Weighted median	5	0.111412	0.050298	0.026758	0.012828	0.209997	1.117856	1.01291	1.233674			0.999387558
CAC	ebi-met1400-GCST90200749	Arachidonate (20:4n6) to pyruvate ratio || id:ebi-met1400-GCST90200749	exposure	Weighted mode	5	0.117095	0.064185	0.142157	-0.00871	0.242897	1.124226	0.991331	1.274937			0.998768904
CAC	ebi-met1400-GCST90200749	Arachidonate (20:4n6) to pyruvate ratio || id:ebi-met1400-GCST90200749	exposure	MR Egger	5	0.11339	0.134834	0.462111	-0.15088	0.377664	1.120069	0.859948	1.458873			0.995501177
CAC	ebi-met1400-GCST90200934	Acetylcarnitine (C2) to propionylcarnitine (C3) ratio || id:ebi-met1400-GCST90200934	exposure	Inverse variance weighted	5	0.102463	0.042627	0.016229	0.018914	0.186012	1.107896	1.019094	1.204437	0.845453179	0.902662097	0.923524134
CAC	ebi-met1400-GCST90200934	Acetylcarnitine (C2) to propionylcarnitine (C3) ratio || id:ebi-met1400-GCST90200934	exposure	Robust adjusted profile score (RAPS)	5	0.102881	0.045453	0.023607	0.013793	0.191969	1.10836	1.013889	1.211633		0.800905514	0.988238216
CAC	ebi-met1400-GCST90200934	Acetylcarnitine (C2) to propionylcarnitine (C3) ratio || id:ebi-met1400-GCST90200934	exposure	Contamination mixture method	5	0.103663	0.066327	0.02509	-0.02634	0.233663	1.109226	0.974006	1.263218			0.712811518

CAC	ebi-met1400-GCST90200934	Acetylcarnitine (C2) to propionylcarnitine (C3) ratio || id:ebi-met1400-GCST90200934	exposure	Weighted median	5	0.084655	0.053638	0.114503	-0.02048	0.189786	1.088342	0.979733	1.20899			0.999387558
CAC	ebi-met1400-GCST90200934	Acetylcarnitine (C2) to propionylcarnitine (C3) ratio || id:ebi-met1400-GCST90200934	exposure	Weighted mode	5	0.077835	0.064262	0.292475	-0.04812	0.203789	1.080945	0.953021	1.22604			0.998768904
CAC	ebi-met1400-GCST90200934	Acetylcarnitine (C2) to propionylcarnitine (C3) ratio || id:ebi-met1400-GCST90200934	exposure	MR Egger	5	0.128988	0.131992	0.400521	-0.12972	0.387693	1.137677	0.878344	1.473577			0.995501177
CAC	ebi-met1400-GCST90200935	Carnitine to propionylcarnitine (C3) ratio || id:ebi-met1400-GCST90200935	exposure	Inverse variance weighted	5	0.100665	0.043114	0.01955	0.016162	0.185167	1.105906	1.016293	1.20342	0.682535254	0.428745538	0.934078042
CAC	ebi-met1400-GCST90200935	Carnitine to propionylcarnitine (C3) ratio || id:ebi-met1400-GCST90200935	exposure	Robust adjusted profile score (RAPS)	5	0.101948	0.045598	0.025365	0.012576	0.191319	1.107326	1.012655	1.210846		0.309056977	0.988238216
CAC	ebi-met1400-GCST90200935	Carnitine to propionylcarnitine (C3) ratio || id:ebi-met1400-GCST90200935	exposure	Contamination mixture method	5	0.179633	0.081633	0.051287	0.019633	0.339633	1.196778	1.019827	1.404432			0.712811518

CAC	ebi-met1400-GCST90200935	Carnitine to propionylcarnitine (C3) ratio || id:ebi-met1400-GCST90200935	exposure	Weighted median	5	0.066624	0.056859	0.241294	-0.04482	0.178067	1.068894	0.956171	1.194906			0.999387558
CAC	ebi-met1400-GCST90200935	Carnitine to propionylcarnitine (C3) ratio || id:ebi-met1400-GCST90200935	exposure	MR Egger	5	0.163029	0.146079	0.345749	-0.12329	0.449344	1.177071	0.884011	1.567283			0.995501177
CAC	ebi-met1400-GCST90200935	Carnitine to propionylcarnitine (C3) ratio || id:ebi-met1400-GCST90200935	exposure	Weighted mode	5	0.031072	0.066085	0.662719	-0.09846	0.160598	1.031559	0.906236	1.174213			0.998768904
CAC	ebi-met1400-GCST90200973	Proline to glutamate ratio || id:ebi-met1400-GCST90200973	exposure	Inverse variance weighted	5	0.08978	0.043668	0.039788	0.00419	0.17537	1.093933	1.004198	1.191687	0.275058237	0.395600713	0.935837484
CAC	ebi-met1400-GCST90200973	Proline to glutamate ratio || id:ebi-met1400-GCST90200973	exposure	Robust adjusted profile score (RAPS)	5	0.082302	0.045548	0.070773	-0.00697	0.171575	1.085783	0.993052	1.187173		0.511785599	0.988238216
CAC	ebi-met1400-GCST90200973	Proline to glutamate ratio || id:ebi-met1400-GCST90200973	exposure	MR Egger	5	0.258734	0.134028	0.149101	-0.00396	0.521428	1.295289	0.996047	1.684432			0.995501177

CAC	ebi-met1400-GCST90200973	Proline to glutamate ratio || id:ebi-met1400-GCST90200973	exposure	Contamination mixture method	5	0.047647	0.091837	0.290099	-0.13235	0.227647	1.048801	0.876032	1.255643			0.738246269
CAC	ebi-met1400-GCST90200973	Proline to glutamate ratio || id:ebi-met1400-GCST90200973	exposure	Weighted median	5	0.045092	0.053257	0.397164	-0.05929	0.149475	1.046124	0.942433	1.161225			0.999387558
CAC	ebi-met1400-GCST90200973	Proline to glutamate ratio || id:ebi-met1400-GCST90200973	exposure	Weighted mode	5	0.041574	0.064881	0.556537	-0.08559	0.168741	1.04245	0.917968	1.183814			0.998768904
CAC	ebi-met1400-GCST90200990	Benzoate to linoleoyl-arachidonoyl-glycerol (18:2 to 20:4) [2] ratio || id:ebi-met1400-GCST90200990	exposure	Inverse variance weighted	4	0.116903	0.053421	0.028646	0.012197	0.221608	1.12401	1.012272	1.248082	0.73190322	0.447044175	0.934078042
CAC	ebi-met1400-GCST90200990	Benzoate to linoleoyl-arachidonoyl-glycerol (18:2 to 20:4) [2] ratio || id:ebi-met1400-GCST90200990	exposure	Robust adjusted profile score (RAPS)	4	0.118203	0.056564	0.036643	0.007337	0.229068	1.125472	1.007364	1.257428		0.290989355	0.988238216
CAC	ebi-met1400-GCST90200990	Benzoate to linoleoyl-arachidonoyl-glycerol (18:2 to 20:4) [2] ratio || id:ebi-met1400-GCST90200990	exposure	Contamination mixture method	4	0.195441	0.109694	0.060535	-0.01956	0.410441	1.215848	0.980632	1.507483			0.712811518

CAC	ebi-met1400-GCST90200990	Benzoate to linoleoyl-arachidonoyl-glycerol (18:2 to 20:4) [2] ratio || id:ebi-met1400-GCST90200990	exposure	Weighted median	4	0.114318	0.064059	0.074329	-0.01124	0.239873	1.121109	0.988826	1.271088			0.999387558
CAC	ebi-met1400-GCST90200990	Benzoate to linoleoyl-arachidonoyl-glycerol (18:2 to 20:4) [2] ratio || id:ebi-met1400-GCST90200990	exposure	MR Egger	4	0.195327	0.207923	0.446684	-0.2122	0.602856	1.215708	0.808801	1.82733			0.995501177
CAC	ebi-met1400-GCST90200990	Benzoate to linoleoyl-arachidonoyl-glycerol (18:2 to 20:4) [2] ratio || id:ebi-met1400-GCST90200990	exposure	Weighted mode	4	0.055468	0.072988	0.502541	-0.08759	0.198524	1.057035	0.916138	1.219602			0.998768904
CAC	ebi-met1400-GCST90200994	Glutamine to alanine ratio || id:ebi-met1400-GCST90200994	exposure	Inverse variance weighted	5	0.093045	0.044425	0.036222	0.005972	0.180118	1.097511	1.00599	1.197359	0.378449439	0.691614487	0.935072545
CAC	ebi-met1400-GCST90200994	Glutamine to alanine ratio || id:ebi-met1400-GCST90200994	exposure	Robust adjusted profile score (RAPS)	5	0.093726	0.047079	0.046498	0.001452	0.186001	1.098259	1.001453	1.204423		0.758302625	0.988238216
CAC	ebi-met1400-GCST90200994	Glutamine to alanine ratio || id:ebi-met1400-GCST90200994	exposure	Contamination mixture method	5	0.108043	0.081633	0.067249	-0.05196	0.268043	1.114096	0.94937	1.307403			0.712811518

CAC	ebi-met1400-GCST90200994	Glutamine to alanine ratio || id:ebi-met1400-GCST90200994	exposure	Weighted median	5	0.071424	0.055013	0.194182	-0.0364	0.179251	1.074037	0.964253	1.196321			0.999387558
CAC	ebi-met1400-GCST90200994	Glutamine to alanine ratio || id:ebi-met1400-GCST90200994	exposure	MR Egger	5	0.227371	0.137673	0.197197	-0.04247	0.497209	1.255295	0.958421	1.644126			0.995501177
CAC	ebi-met1400-GCST90200994	Glutamine to alanine ratio || id:ebi-met1400-GCST90200994	exposure	Weighted mode	5	0.079922	0.065574	0.289878	-0.0486	0.208446	1.083202	0.95256	1.231763			0.998768904

nsnp: number of single nucleotide polymorphisms, se: standard error, lo_ci: lower confidence interval, up_ci: upper confidence interval, or: odds ratio, or_lci95: odds ratio lower 95% confidence interval limit, or_uci95: odds ratio upper 95% confidence interval limit, p-FDR: p-value after False Discovery Rate correction

Table S6 Mendelian Randomization analysis on the causal effect between immune cells and coronary artery calcification.

id.exposure	id.outcome	outcome	exposure	method	nsnp	b	se	pval	lo_ci	up_ci	or	or_lci95	or_uci95	pleiotropy	heterogeneity
ebi-a-GCST90001400	CAC	outcome	|| IgD+ B cell Absolute Count id:ebi-a-GCST90001400	Inverse variance weighted	17	0.06102	0.021358427	0.00428	0.019157044	0.102882078	1.062919706	1.019341718	1.1083607	0.525836095	0.828760335
ebi-a-GCST90001400	CAC	outcome	|| id:ebi-a-GCST90001400	MR Egger	17	0.050078	0.02720222	0.08549	-0.003238108	0.103394593	1.051353354	0.99676713	1.1089289		0.803158615
ebi-a-GCST90001400	CAC	outcome	|| id:ebi-a-GCST90001400	Weighted median	17	0.045794	0.032912495	0.16411	-0.018714647	0.110302334	1.046858572	0.981459385	1.1166156		
ebi-a-GCST90001400	CAC	outcome	|| id:ebi-a-GCST90001400	Weighted mode	17	0.045607	0.028438183	0.12833	-0.010132208	0.10134547	1.046662605	0.98991895	1.1066589		
ebi-a-GCST90001400	CAC	outcome	|| id:ebi-a-GCST90001400	Contamination mixture method	17	0.050328	0.033163265	0.03705	-0.014672131	0.115327869	1.051615832	0.98543498	1.1222413		
ebi-a-GCST90001400	CAC	outcome	|| id:ebi-a-GCST90001400	Robust adjusted profile score (RAPS)	17	0.061414	0.024724433	0.01299	0.012954194	0.10987397	1.063339133	1.013038463	1.1161374		
ebi-a-GCST90001410	CAC	outcome	IgD- CD38+ B cell %B cell || id:ebi-a-GCST90001410	Inverse variance weighted	17	0.091208	0.039417754	0.02067	0.0139488	0.168466397	1.095496406	1.014046539	1.1834885	0.415342901	0.707145718

ebi-a-GCST90001410	CAC	outcome	|| id:ebi-a-GCST90001410	MR Egger	17	0.033298	0.079576591	0.68155	-0.122671648	0.189268589	1.03385907	0.88455406	1.2083655		0.692384239
ebi-a-GCST90001410	CAC	outcome	|| id:ebi-a-GCST90001410	Weighted median	17	0.109837	0.052956451	0.03807	0.006042285	0.213631572	1.116096052	1.006060577	1.2381664		
ebi-a-GCST90001410	CAC	outcome	|| id:ebi-a-GCST90001410	Weighted mode	17	0.113865	0.081898697	0.18347	-0.046656149	0.274386744	1.120601167	0.954415518	1.3157236		
ebi-a-GCST90001410	CAC	outcome	|| id:ebi-a-GCST90001410	Contamination mixture method	17	0.102391	0.051020408	0.02809	0.002391304	0.202391304	1.10781688	1.002394166	1.224327		
ebi-a-GCST90001410	CAC	outcome	|| id:ebi-a-GCST90001410	Robust adjusted profile score (RAPS)	17	0.098743	0.043112929	0.022	0.014242062	0.183244745	1.103783036	1.014343963	1.2011083		
ebi-a-GCST90001457	CAC	outcome	CD11c+ HLA DR++ monocyte %monocyte|| id:ebi-a-GCST90001457	Inverse variance weighted	15	-0.05121	0.024384987	0.03573	-0.099003412	-0.003414261	0.950080238	0.905739617	0.9965916	0.517443318	0.474538471
ebi-a-GCST90001457	CAC	outcome	|| id:ebi-a-GCST90001457	MR Egger	15	-0.06293	0.030249464	0.05783	-0.122219092	-0.003641194	0.939009068	0.884954461	0.9963654		0.430946525
ebi-a-GCST90001457	CAC	outcome	|| id:ebi-a-GCST90001457	Weighted median	15	-0.05816	0.034253449	0.08953	-0.125295573	0.008977947	0.943500095	0.882236098	1.0090184		

ebi-a-GCST90001457	CAC	outcome	|| id:ebi-a-GCST90001457	Weighted mode	15	-0.05807	0.032794642	0.09834	-0.122352103	0.006202894	0.943579549	0.88483676	1.0062222		
ebi-a-GCST90001457	CAC	outcome	|| id:ebi-a-GCST90001457	Contamination mixture method	15	-0.04945	0.022959184	0.02856	-0.094449686	-0.004449686	0.951753044	0.909873513	0.9955602		
ebi-a-GCST90001457	CAC	outcome	|| id:ebi-a-GCST90001457	Robust adjusted profile score (RAPS)	15	-0.0518	0.027649162	0.06101	-0.105991266	0.00239345	0.949519788	0.899432503	1.0023963		
ebi-a-GCST90001458	CAC	outcome	Myeloid Dendritic Cell Absolute Count|| id:ebi-a-GCST90001458	Inverse variance weighted	22	-0.05728	0.024161457	0.01775	-0.104635814	-0.009922903	0.944330226	0.900652468	0.9901262	0.060097733	0.399579267
ebi-a-GCST90001458	CAC	outcome	|| id:ebi-a-GCST90001458	MR Egger	22	-0.09695	0.030877705	0.00516	-0.157465577	-0.036424974	0.907605673	0.854306221	0.9642304		0.585615002
ebi-a-GCST90001458	CAC	outcome	|| id:ebi-a-GCST90001458	Weighted median	22	-0.08723	0.038180119	0.02234	-0.162060052	-0.012393985	0.916469016	0.850390135	0.9876825		
ebi-a-GCST90001458	CAC	outcome	|| id:ebi-a-GCST90001458	Weighted mode	22	-0.08945	0.038679104	0.03098	-0.165258578	-0.01363649	0.91443624	0.847674485	0.9864561		
ebi-a-GCST90001458	CAC	outcome	|| id:ebi-a-GCST90001458	Contamination mixture method	22	-0.09593	0.030612245	0.03581	-0.15593361	-0.03593361	0.908524331	0.855615993	0.9647043		

ebi-a-GCST90001458	CAC	outcome	|| id:ebi-a-GCST90001458	Robust adjusted profile score (RAPS)	22	-0.0582	0.025072218	0.02027	-0.107340809	-0.009057715	0.943461933	0.8982195	0.9909832		
ebi-a-GCST90001476	CAC	outcome	HLA DR++ monocyte %leukocyte|| id:ebi-a-GCST90001476	Inverse variance weighted	8	-0.15992	0.06735718	0.01759	-0.291940824	-0.02790068	0.852211322	0.746812728	0.9724849	0.525409637	0.59352559
ebi-a-GCST90001476	CAC	outcome	|| id:ebi-a-GCST90001476	MR Egger	8	-0.2245	0.117123223	0.10373	-0.454063156	0.005059877	0.798914267	0.635042625	1.0050727		0.531982337
ebi-a-GCST90001476	CAC	outcome	|| id:ebi-a-GCST90001476	Weighted median	8	-0.14237	0.089601786	0.11208	-0.31798977	0.033249232	0.867300063	0.727610232	1.0338082		
ebi-a-GCST90001476	CAC	outcome	|| id:ebi-a-GCST90001476	Weighted mode	8	-0.14137	0.100974325	0.20423	-0.339278788	0.056540568	0.868168803	0.712283845	1.0581695		
ebi-a-GCST90001476	CAC	outcome	|| id:ebi-a-GCST90001476	Contamination mixture method	8	-0.18736	0.089285714	0.07158	-0.362359017	-0.012359017	0.829146005	0.696032435	0.987717		
ebi-a-GCST90001476	CAC	outcome	|| id:ebi-a-GCST90001476	Robust adjusted profile score (RAPS)	8	-0.16217	0.07391683	0.02824	-0.307043779	-0.017289804	0.85029937	0.735618396	0.9828588		

ebi-a-GCST90001480	CAC	outcome	Resting CD4 regulatory T cell Absolute Count || id:ebi-a-GCST90001480	Inverse variance weighted	24	0.055323	0.027109544	0.04128	0.002188597	0.10845801	1.056882253	1.002190994	1.1145581	0.364588127	0.584285592
ebi-a-GCST90001480	CAC	outcome	|| id:ebi-a-GCST90001480	MR Egger	24	0.024457	0.042970644	0.57502	-0.059765685	0.10867924	1.024758298	0.941985229	1.1148047		0.577378528
ebi-a-GCST90001480	CAC	outcome	|| id:ebi-a-GCST90001480	Weighted median	24	0.037105	0.038421203	0.33418	-0.038200869	0.112410245	1.037801661	0.962519581	1.1189718		
ebi-a-GCST90001480	CAC	outcome	|| id:ebi-a-GCST90001480	Weighted mode	24	0.039346	0.037493876	0.3049	-0.034142058	0.112833937	1.040130243	0.966434205	1.119446		
ebi-a-GCST90001480	CAC	outcome	|| id:ebi-a-GCST90001480	Contamination mixture method	24	0.058701	0.051020408	0.13193	-0.041298838	0.158701162	1.060458288	0.959542339	1.1719877		
ebi-a-GCST90001480	CAC	outcome	|| id:ebi-a-GCST90001480	Robust adjusted profile score (RAPS)	24	0.056773	0.029183515	0.05173	-0.000426414	0.113972966	1.058415815	0.999573677	1.1207218		
ebi-a-GCST90001507	CAC	outcome	CD25++ CD45RA+ CD4 not regulatory T cell Absolute Count|| id:ebi-a-GCST90001507	Inverse variance weighted	26	0.050858	0.017690226	0.00404	0.016184944	0.08553063	1.05217325	1.01631663	1.0892949	0.721340376	0.258782039
ebi-a-GCST90001507	CAC	outcome	|| id:ebi-a-GCST90001507	MR Egger	26	0.055886	0.022767663	0.02173	0.011261603	0.10051084	1.057477359	1.011325253	1.1057356		0.221397475

ebi-a-GCST90001507	CAC	outcome	|| id:ebi-a-GCST90001507	Weighted median	26	0.052671	0.026765276	0.04908	0.000210953	0.105130835	1.054082683	1.000210975	1.1108559		
ebi-a-GCST90001507	CAC	outcome	|| id:ebi-a-GCST90001507	Weighted mode	26	0.055748	0.021107158	0.01404	0.014377563	0.09711762	1.057330771	1.014481417	1.10199		
ebi-a-GCST90001507	CAC	outcome	|| id:ebi-a-GCST90001507	Contamination mixture method	26	0.070151	0.017857143	0.00128	0.035151455	0.105151455	1.07267063	1.03577657	1.1108788		
ebi-a-GCST90001507	CAC	outcome	|| id:ebi-a-GCST90001507	Robust adjusted profile score (RAPS)	26	0.056309	0.01869587	0.0026	0.019664609	0.092952421	1.057924019	1.019859231	1.0974095		
ebi-a-GCST90001521	CAC	outcome	CD33+ HLA DR+ CD14dim %CD33+ HLA DR+|| id:ebi-a-GCST90001521	Inverse variance weighted	24	-0.06477	0.023622585	0.00611	-0.111065295	-0.018464762	0.937287674	0.894880316	0.9817047	0.970513087	0.614366982
ebi-a-GCST90001521	CAC	outcome	|| id:ebi-a-GCST90001521	MR Egger	24	-0.06603	0.041349297	0.12454	-0.147078486	0.015010759	0.936099164	0.863226224	1.015124		0.554724707
ebi-a-GCST90001521	CAC	outcome	|| id:ebi-a-GCST90001521	Weighted median	24	-0.07803	0.035732678	0.02898	-0.148067792	-0.007995695	0.924935065	0.862372651	0.9920362		
ebi-a-GCST90001521	CAC	outcome	|| id:ebi-a-GCST90001521	Weighted mode	24	-0.08087	0.05612346	0.16306	-0.19087639	0.029127572	0.922309518	0.826234712	1.0295559		

ebi-a-GCST90001521	CAC	outcome	|| id:ebi-a-GCST90001521	Contamination mixture method	24	-0.10697	0.043367347	0.02528	-0.191972395	-0.021972395	0.898550477	0.825329651	0.9782672		
ebi-a-GCST90001521	CAC	outcome	|| id:ebi-a-GCST90001521	Robust adjusted profile score (RAPS)	24	-0.06627	0.02573009	0.01001	-0.116698974	-0.015837022	0.935880017	0.889853021	0.9842877		
ebi-a-GCST90001543	CAC	outcome	Effector Memory CD4+ T cell %CD4+ T cell|| id:ebi-a-GCST90001543	Inverse variance weighted	27	-0.05443	0.026875253	0.04282	-0.107109259	-0.001758266	0.947021235	0.898427507	0.9982433	0.399353173	0.493252352
ebi-a-GCST90001543	CAC	outcome	|| id:ebi-a-GCST90001543	MR Egger	27	-0.07797	0.038418927	0.05317	-0.153274914	-0.002672719	0.924988646	0.857893842	0.9973308		0.478059517
ebi-a-GCST90001543	CAC	outcome	|| id:ebi-a-GCST90001543	Weighted median	27	-0.07082	0.039148868	0.07047	-0.147548234	0.00591533	0.931632876	0.86282082	1.0059329		
ebi-a-GCST90001543	CAC	outcome	|| id:ebi-a-GCST90001543	Weighted mode	27	-0.08538	0.041349075	0.04904	-0.166425693	-0.004337318	0.918161934	0.846685729	0.9956721		
ebi-a-GCST90001543	CAC	outcome	|| id:ebi-a-GCST90001543	Contamination mixture method	27	-0.09283	0.030612245	0.00686	-0.152831472	-0.032831472	0.911347075	0.858274353	0.9677016		
ebi-a-GCST90001543	CAC	outcome	|| id:ebi-a-GCST90001543	Robust adjusted profile	27	-0.06117	0.029078463	0.03542	-0.118162396	-0.004174821	0.940664622	0.888551744	0.9958339		

				score (RAPS)											
ebi-a-GCST90001560	CAC	outcome	CD45RA+ CD8+ T cell Absolute Count|| id:ebi-a-GCST90001560	Inverse variance weighted	26	-0.02359	0.010142078	0.02002	-0.043468957	-0.003712011	0.976685596	0.957462276	0.9962949	0.255650682	0.632338993
ebi-a-GCST90001560	CAC	outcome	|| id:ebi-a-GCST90001560	MR Egger	26	-0.02822	0.010892884	0.01604	-0.049568492	-0.006868388	0.972175981	0.951639976	0.9931551		0.656191392
ebi-a-GCST90001560	CAC	outcome	|| id:ebi-a-GCST90001560	Weighted median	26	-0.02782	0.014360368	0.05267	-0.055971246	0.000321395	0.972558623	0.945566325	1.0003214		
ebi-a-GCST90001560	CAC	outcome	|| id:ebi-a-GCST90001560	Weighted mode	26	-0.02479	0.010850875	0.03112	-0.046052765	-0.003517334	0.975519578	0.954991571	0.9964888		
ebi-a-GCST90001560	CAC	outcome	|| id:ebi-a-GCST90001560	Contamination mixture method	26	-0.03163	0.010204082	0.08427	-0.051629073	-0.011629073	0.968865894	0.949681064	0.9884383		
ebi-a-GCST90001560	CAC	outcome	|| id:ebi-a-GCST90001560	Robust adjusted profile score (RAPS)	26	-0.0235	0.013246595	0.07604	-0.04946445	0.002462201	0.976772876	0.951738992	1.0024652		
ebi-a-GCST90001596	CAC	outcome	CD8dim T cell Absolute Count|| id:ebi-a-GCST90001596	Inverse variance weighted	27	-0.06097	0.029070843	0.03597	-0.117947929	-0.003990223	0.940852334	0.888742329	0.9960177	0.399016012	0.583588266

ebi-a-GCST90001596	CAC	outcome	|| id:ebi-a-GCST90001596	MR Egger	27	-0.08832	0.043145492	0.05129	-0.172890061	-0.003759734	0.915463396	0.841230094	0.9962473		0.569947056
ebi-a-GCST90001596	CAC	outcome	|| id:ebi-a-GCST90001596	Weighted median	27	-0.09038	0.045323876	0.04615	-0.179211208	-0.001541615	0.913587236	0.835929326	0.9984596		
ebi-a-GCST90001596	CAC	outcome	|| id:ebi-a-GCST90001596	Weighted mode	27	-0.08796	0.047446637	0.07515	-0.180952114	0.005038704	0.915800526	0.834475318	1.0050514		
ebi-a-GCST90001596	CAC	outcome	|| id:ebi-a-GCST90001596	Contamination mixture method	27	-0.09324	0.035714286	0.02213	-0.163240418	-0.023240418	0.910974459	0.849386956	0.9770276		
ebi-a-GCST90001596	CAC	outcome	|| id:ebi-a-GCST90001596	Robust adjusted profile score (RAPS)	27	-0.06543	0.03161223	0.03847	-0.12739022	-0.00347028	0.936664377	0.880390059	0.9965357		
ebi-a-GCST90001661	CAC	outcome	CD28- CD8dim T cell %T cell|| id:ebi-a-GCST90001661	Inverse variance weighted	23	-0.06955	0.032299686	0.0313	-0.132855787	-0.006241017	0.932814982	0.875591354	0.9937784	0.274459898	0.193119208
ebi-a-GCST90001661	CAC	outcome	|| id:ebi-a-GCST90001661	MR Egger	23	-0.02594	0.05040891	0.61217	-0.124744878	0.072858049	0.974390225	0.882722076	1.0755778		0.208858229
ebi-a-GCST90001661	CAC	outcome	|| id:ebi-a-GCST90001661	Weighted median	23	-0.06002	0.04947729	0.22512	-0.15699212	0.036958858	0.941748871	0.854710794	1.0376503		

ebi-a-GCST90001661	CAC	outcome	|| id:ebi-a-GCST90001661	Weighted mode	23	-0.05492	0.052169465	0.30393	-0.15716827	0.047336033	0.946564544	0.85456025	1.0484743		
ebi-a-GCST90001661	CAC	outcome	|| id:ebi-a-GCST90001661	Contamination mixture method	23	-0.11167	0.06122449	0.046	-0.231670516	0.008329484	0.89433888	0.79320743	1.0083643		
ebi-a-GCST90001661	CAC	outcome	|| id:ebi-a-GCST90001661	Robust adjusted profile score (RAPS)	23	-0.07123	0.034722485	0.04022	-0.139287299	-0.00317516	0.931246536	0.869978049	0.9968299		
ebi-a-GCST90001677	CAC	outcome	CD28- CD25++ CD8+ T cell %CD8+ T cell|| id:ebi-a-GCST90001677	Inverse variance weighted	23	0.072095	0.036023256	0.04536	0.001488984	0.142700147	1.074756974	1.001490093	1.1533839	0.242095416	0.845547274
ebi-a-GCST90001677	CAC	outcome	|| id:ebi-a-GCST90001677	MR Egger	23	0.006714	0.0651756	0.91893	-0.121029835	0.134458515	1.006736932	0.886007525	1.1439172		0.872659057
ebi-a-GCST90001677	CAC	outcome	|| id:ebi-a-GCST90001677	Weighted median	23	0.066471	0.048342358	0.16913	-0.028279672	0.161222371	1.068730344	0.972116455	1.1749462		
ebi-a-GCST90001677	CAC	outcome	|| id:ebi-a-GCST90001677	Weighted mode	23	0.015816	0.066651167	0.81462	-0.114819807	0.146452768	1.015942223	0.891526776	1.1577202		
ebi-a-GCST90001677	CAC	outcome	|| id:ebi-a-GCST90001677	Contamination mixture method	23	0.082354	0.058673469	0.0683	-0.032645601	0.197354399	1.085840562	0.967881515	1.2181757		

ebi-a-GCST90001677	CAC	outcome	|| id:ebi-a-GCST90001677	Robust adjusted profile score (RAPS)	23	0.077493	0.039704479	0.05097	-0.000328004	0.155313552	1.080574424	0.99967205	1.1680241		
ebi-a-GCST90001678	CAC	outcome	CD28- CD25++ CD8+ T cell Absolute Count|| id:ebi-a-GCST90001678	Inverse variance weighted	24	0.062551	0.024831364	0.01177	0.013881452	0.1112204	1.064548671	1.013978247	1.1176412	0.173700209	0.435121566
ebi-a-GCST90001678	CAC	outcome	|| id:ebi-a-GCST90001678	MR Egger	24	0.092257	0.032424168	0.00941	0.028705194	0.155807934	1.096646145	1.029121158	1.1686017		0.492058608
ebi-a-GCST90001678	CAC	outcome	|| id:ebi-a-GCST90001678	Weighted median	24	0.053652	0.037682419	0.1545	-0.020205306	0.127509778	1.055117606	0.979997453	1.135996		
ebi-a-GCST90001678	CAC	outcome	|| id:ebi-a-GCST90001678	Weighted mode	24	0.042413	0.033599738	0.21949	-0.023441995	0.10826898	1.043325797	0.976830634	1.1143474		
ebi-a-GCST90001678	CAC	outcome	|| id:ebi-a-GCST90001678	Contamination mixture method	24	0.040487	0.038265306	0.20158	-0.034513034	0.115486966	1.041317738	0.966075748	1.1224199		
ebi-a-GCST90001678	CAC	outcome	|| id:ebi-a-GCST90001678	Robust adjusted profile score (RAPS)	24	0.058896	0.027331148	0.03117	0.005327017	0.112465119	1.060664998	1.005341231	1.1190332		

ebi-a-GCST90001760	CAC	outcome	CD20 on unswitched memory B cell|| id:ebi-a-GCST90001760	Inverse variance weighted	20	-0.08558	0.034224214	0.0124	-0.152655018	-0.018496101	0.917983778	0.858425812	0.9816739	0.249114768	0.369613097
ebi-a-GCST90001760	CAC	outcome	|| id:ebi-a-GCST90001760	MR Egger	20	-0.02402	0.06178109	0.70195	-0.145114194	0.097067679	0.976263004	0.864923519	1.1019349		0.396150932
ebi-a-GCST90001760	CAC	outcome	|| id:ebi-a-GCST90001760	Weighted median	20	-0.10428	0.046016727	0.02344	-0.19447741	-0.014091839	0.900968823	0.823264775	0.986007		
ebi-a-GCST90001760	CAC	outcome	|| id:ebi-a-GCST90001760	Weighted mode	20	-0.08722	0.052064487	0.11026	-0.189267961	0.014824829	0.916474014	0.827564722	1.0149353		
ebi-a-GCST90001760	CAC	outcome	|| id:ebi-a-GCST90001760	Contamination mixture method	20	-0.09459	0.06122449	0.06137	-0.214592934	0.025407066	0.909743185	0.806869823	1.0257326		
ebi-a-GCST90001760	CAC	outcome	|| id:ebi-a-GCST90001760	Robust adjusted profile score (RAPS)	20	-0.08562	0.035758839	0.01665	-0.15570856	-0.015533913	0.917941848	0.855808571	0.9845861		
ebi-a-GCST90001797	CAC	outcome	CD27 on CD20- CD38- B cell|| id:ebi-a-GCST90001797	Inverse variance weighted	18	-0.08857	0.036960062	0.01656	-0.161010217	-0.016126774	0.915240419	0.851283374	0.9840026	0.308367956	0.30666575
ebi-a-GCST90001797	CAC	outcome	|| id:ebi-a-GCST90001797	MR Egger	18	-0.04504	0.055398261	0.42812	-0.153622312	0.06353887	0.955957598	0.857595864	1.0656009		0.316088975

ebi-a-GCST90001797	CAC	outcome	|| id:ebi-a-GCST90001797	Weighted median	18	-0.08338	0.052996863	0.11566	-0.187251358	0.020496344	0.920003774	0.829235275	1.0207078		
ebi-a-GCST90001797	CAC	outcome	|| id:ebi-a-GCST90001797	Weighted mode	18	-0.08496	0.055456515	0.14394	-0.193649941	0.023739598	0.918553461	0.823946283	1.0240236		
ebi-a-GCST90001797	CAC	outcome	|| id:ebi-a-GCST90001797	Contamination mixture method	18	-0.10369	0.043367347	0.04514	-0.188691275	-0.018691275	0.901503571	0.828042104	0.9814823		
ebi-a-GCST90001797	CAC	outcome	|| id:ebi-a-GCST90001797	Robust adjusted profile score (RAPS)	18	-0.08598	0.038585584	0.02586	-0.161607151	-0.010351663	0.917613127	0.850775365	0.9897017		
ebi-a-GCST90001883	CAC	outcome	CD16-CD56 on Natural Killer T|| id:ebi-a-GCST90001883	Inverse variance weighted	27	0.06153	0.029130661	0.03467	0.004434026	0.118626216	1.063462529	1.004443871	1.125949	0.408208429	0.34222894
ebi-a-GCST90001883	CAC	outcome	|| id:ebi-a-GCST90001883	MR Egger	27	0.09798	0.052304648	0.07277	-0.004537247	0.200496972	1.102940574	0.995473031	1.2220099		0.328792279
ebi-a-GCST90001883	CAC	outcome	|| id:ebi-a-GCST90001883	Weighted median	27	0.024195	0.040721453	0.55241	-0.055619375	0.104008719	1.024489738	0.9458991	1.1096101		
ebi-a-GCST90001883	CAC	outcome	|| id:ebi-a-GCST90001883	Weighted mode	27	0.00172	0.0524102	0.97406	-0.101003552	0.104444433	1.001721921	0.903929822	1.1100937		

ebi-a-GCST90001883	CAC	outcome	|| id:ebi-a-GCST90001883	Contamination mixture method	27	0.038942	0.028061224	0.18419	-0.016057651	0.093942349	1.039710542	0.984070586	1.0984964		
ebi-a-GCST90001883	CAC	outcome	|| id:ebi-a-GCST90001883	Robust adjusted profile score (RAPS)	27	0.056692	0.02978147	0.05696	-0.001679426	0.115063938	1.058330066	0.998321983	1.1219452		
ebi-a-GCST90001975	CAC	outcome	FSC-A on HLA DR+ T cell|| id:ebi-a-GCST90001975	Inverse variance weighted	14	-0.11048	0.045389044	0.01493	-0.199443766	-0.021518715	0.895403128	0.819186286	0.9787112	0.850351716	0.283079408
ebi-a-GCST90001975	CAC	outcome	|| id:ebi-a-GCST90001975	MR Egger	14	-0.09698	0.084435989	0.27312	-0.262474668	0.068514409	0.90757404	0.769145848	1.0709161		0.222749911
ebi-a-GCST90001975	CAC	outcome	|| id:ebi-a-GCST90001975	Weighted median	14	-0.12177	0.063418917	0.05484	-0.246074437	0.002527718	0.885349002	0.781864023	1.0025309		
ebi-a-GCST90001975	CAC	outcome	|| id:ebi-a-GCST90001975	Weighted mode	14	-0.13322	0.074273442	0.09616	-0.27879387	0.012358023	0.875274328	0.756695865	1.0124347		
ebi-a-GCST90001975	CAC	outcome	|| id:ebi-a-GCST90001975	Contamination mixture method	14	-0.19054	0.081632653	0.01019	-0.350538441	-0.030538441	0.826513985	0.704308759	0.9699231		
ebi-a-GCST90001975	CAC	outcome	|| id:ebi-a-GCST90001975	Robust adjusted profile	14	-0.11693	0.047080781	0.01301	-0.209209676	-0.024653012	0.88964627	0.811225124	0.9756484		

				score (RAPS)											
ebi-a-GCST90002020	CAC	outcome	CD14 on CD33+ HLA DR+ CD14dim|| id:ebi-a-GCST90002020	Inverse variance weighted	19	0.076386	0.02587582	0.00316	0.025669511	0.127102726	1.079379262	1.02600181	1.1355337	0.902321089	0.731376652
ebi-a-GCST90002020	CAC	outcome	|| id:ebi-a-GCST90002020	MR Egger	19	0.082025	0.052137151	0.13409	-0.020164071	0.184213561	1.08548267	0.980037864	1.2022726		0.670681938
ebi-a-GCST90002020	CAC	outcome	|| id:ebi-a-GCST90002020	Weighted median	19	0.079372	0.03708346	0.03233	0.006688448	0.15205561	1.082607008	1.006710865	1.164225		
ebi-a-GCST90002020	CAC	outcome	|| id:ebi-a-GCST90002020	Weighted mode	19	0.072936	0.047737719	0.14393	-0.020630112	0.166501748	1.075661496	0.979581233	1.1811656		
ebi-a-GCST90002020	CAC	outcome	|| id:ebi-a-GCST90002020	Contamination mixture method	19	0.094629	0.028061224	0.00384	0.039629345	0.149629345	1.099251336	1.040425064	1.1614037		
ebi-a-GCST90002020	CAC	outcome	|| id:ebi-a-GCST90002020	Robust adjusted profile score (RAPS)	19	0.082908	0.028760263	0.00394	0.026538146	0.139278376	1.086442135	1.026893418	1.149444		
ebi-a-GCST90002039	CAC	outcome	CD80 on monocyte|| id:ebi-a-GCST90002039	Inverse variance weighted	21	-0.07378	0.028705483	0.01017	-0.130037967	-0.017512475	0.928880463	0.878062093	0.98264	0.699026594	0.618921967

ebi-a-GCST90002039	CAC	outcome	|| id:ebi-a-GCST90002039	MR Egger	21	-0.05942	0.046489796	0.2166	-0.150540321	0.031699679	0.942310613	0.860243043	1.0322075		0.565017487
ebi-a-GCST90002039	CAC	outcome	|| id:ebi-a-GCST90002039	Weighted median	21	-0.04582	0.043646494	0.29381	-0.131367615	0.039726643	0.955213421	0.876895355	1.0405263		
ebi-a-GCST90002039	CAC	outcome	|| id:ebi-a-GCST90002039	Weighted mode	21	-0.03104	0.05789319	0.59773	-0.144513484	0.082427821	0.96943405	0.865443243	1.0859203		
ebi-a-GCST90002039	CAC	outcome	|| id:ebi-a-GCST90002039	Contamination mixture method	21	-0.13027	0.058673469	0.0716	-0.24526952	-0.01526952	0.877858798	0.782493612	0.9848465		
ebi-a-GCST90002039	CAC	outcome	|| id:ebi-a-GCST90002039	Robust adjusted profile score (RAPS)	21	-0.07658	0.030626589	0.01241	-0.136607096	-0.016550869	0.926279751	0.872312894	0.9835853		

nsnp: number of single nucleotide polymorphisms, se: standard error, lo_ci: lower confidence interval, up_ci: upper confidence interval, or: odds ratio, or_lci95: odds ratio lower 95% confidence interval limit, or_uci95: odds ratio upper 95% confidence interval limit, p-FDR: p-value after False Discovery Rate correction

Table S7 Mendelian Randomization analysis on the causal effect between coronary artery calcification and immune cells.

id.exposure	id.outcome	outcome	exposure	method	nsnp	b	se	pval	lo_ci	up_ci	or	or_lci95	or_uci95	pleiotropy	heterogeneity
CAC	ebi-a-GCST90001398	Unswitched memory B cell Absolute Count || id:ebi-a-GCST90001398	exposure	Inverse variance weighted	5	-0.1580924	0.0805416	0.04966171	-0.31595393	-0.0002309	0.8537709	0.72909304	0.99976917	0.648790606	0.962317309
CAC	ebi-a-GCST90001398	Unswitched memory B cell Absolute Count || id:ebi-a-GCST90001398	exposure	MR Egger	5	-0.0351249	0.2568192	0.89987595	-0.53849065	0.4682408	0.96548479	0.58362849	1.59718196		0.949885217
CAC	ebi-a-GCST90001398	Unswitched memory B cell Absolute Count || id:ebi-a-GCST90001398	exposure	Weighted median	5	-0.1599139	0.0946417	0.09108978	-0.34541172	0.02558384	0.85221713	0.70792883	1.02591392		
CAC	ebi-a-GCST90001398	Unswitched memory B cell Absolute Count || id:ebi-a-GCST90001398	exposure	Weighted mode	5	-0.1627479	0.1103702	0.2143472	-0.37907345	0.05357757	0.84980536	0.68449534	1.05503883		
CAC	ebi-a-GCST90001398	Unswitched memory B cell Absolute Count || id:ebi-a-GCST90001398	exposure	Contamination mixture method	5	-0.1569069	0.1071429	0.05424985	-0.36690695	0.05309305	0.8547836	0.69287412	1.05452777		
CAC	ebi-a-GCST90001398	Unswitched memory B cell Absolute Count || id:ebi-a-GCST90001398	exposure	Robust adjusted profile score (RAPS)	5	-0.1587385	0.0857936	0.06427946	-0.32689408	0.009417	0.85321942	0.72116013	1.00946148		
CAC	ebi-a-GCST90001466	CD86+ plasmacytoid Dendritic Cell Absolute	exposure	Inverse variance weighted	5	0.16277585	0.0799205	0.04167795	0.006131631	0.31942006	1.17677288	1.00615047	1.37632935	0.51991365	0.908466221

		Count || id:ebi-a-GCST90001466													
CAC	ebi-a-GCST90001466	CD86+ plasmacytoid Dendritic Cell Absolute Count || id:ebi-a-GCST90001466	exposure	MR Egger	5	0.34124523	0.2582257	0.27807735	-0.16487708	0.84736755	1.40669817	0.84799793	2.33349594		0.92315915
CAC	ebi-a-GCST90001466	CD86+ plasmacytoid Dendritic Cell Absolute Count || id:ebi-a-GCST90001466	exposure	Weighted median	5	0.13993258	0.09789	0.15286399	-0.0519318	0.33179697	1.15019626	0.94939361	1.39346991		
CAC	ebi-a-GCST90001466	CD86+ plasmacytoid Dendritic Cell Absolute Count || id:ebi-a-GCST90001466	exposure	Weighted mode	5	0.10819718	0.1182323	0.41190293	-0.12353822	0.33993258	1.11426743	0.88378787	1.40485287		
CAC	ebi-a-GCST90001466	CD86+ plasmacytoid Dendritic Cell Absolute Count || id:ebi-a-GCST90001466	exposure	Contamination mixture method	5	0.16116331	0.127551	0.05321896	-0.08883669	0.41116331	1.17487682	0.91499499	1.5085717		
CAC	ebi-a-GCST90001466	CD86+ plasmacytoid Dendritic Cell Absolute Count || id:ebi-a-GCST90001466	exposure	Robust adjusted profile score (RAPS)	5	0.1633838	0.0849606	0.05447328	-0.00313896	0.32990655	1.17748852	0.99686596	1.39083815		
CAC	ebi-a-GCST90001486	Activated CD4 regulatory T cell Absolute Count || id:ebi-a-GCST90001486	exposure	Inverse variance weighted	5	-0.17083	0.0816844	0.03649751	-0.33093155	-0.0107285	0.84296484	0.71825433	0.98932883	0.295477021	0.531417389

CAC	ebi-a-GCST90001486	Activated CD4 regulatory T cell Absolute Count || id:ebi-a-GCST90001486	exposure	MR Egger	5	-0.4873546	0.2633787	0.16136133	-1.00357688	0.02886761	0.61424916	0.36656593	1.02928832		0.668037393
CAC	ebi-a-GCST90001486	Activated CD4 regulatory T cell Absolute Count || id:ebi-a-GCST90001486	exposure	Weighted median	5	-0.2058701	0.1026994	0.04500698	-0.40716096	-0.0045793	0.81393878	0.66553705	0.99543118		
CAC	ebi-a-GCST90001486	Activated CD4 regulatory T cell Absolute Count || id:ebi-a-GCST90001486	exposure	Weighted mode	5	-0.2115786	0.1224198	0.15899771	-0.45152151	0.02836422	0.80930563	0.63665873	1.02877031		
CAC	ebi-a-GCST90001486	Activated CD4 regulatory T cell Absolute Count || id:ebi-a-GCST90001486	exposure	Contamination mixture method	5	-0.2340629	0.1045918	0.02224853	-0.43906291	-0.0290629	0.79131203	0.64464022	0.97135535		
CAC	ebi-a-GCST90001486	Activated CD4 regulatory T cell Absolute Count || id:ebi-a-GCST90001486	exposure	Robust adjusted profile score (RAPS)	5	-0.1788086	0.0866661	0.03909453	-0.34867405	-0.0089431	0.83626596	0.70562309	0.99109676		
CAC	ebi-a-GCST90001489	CD39+ activated CD4 regulatory T cell Absolute Count || id:ebi-a-GCST90001489	exposure	Inverse variance weighted	5	-0.1666568	0.0840324	0.04734009	-0.33136029	-0.0019533	0.84649007	0.71794645	0.99804859	0.392741241	0.730871744
CAC	ebi-a-GCST90001489	CD39+ activated CD4 regulatory T cell Absolute Count || id:ebi-a-GCST90001489	exposure	MR Egger	5	-0.4210339	0.2689158	0.21539851	-0.94810899	0.10604114	0.65636784	0.38747305	1.11186762		0.792796875

CAC	ebi-a-GCST90001489	CD39+ activated CD4 regulatory T cell Absolute Count || id:ebi-a-GCST90001489	exposure	Weighted median	5	-0.1294232	0.1067224	0.22524151	-0.33859916	0.07975284	0.8786021	0.7127681	1.08301936		
CAC	ebi-a-GCST90001489	CD39+ activated CD4 regulatory T cell Absolute Count || id:ebi-a-GCST90001489	exposure	Weighted mode	5	-0.1101064	0.1282429	0.43897849	-0.36146243	0.14124962	0.89573882	0.69665677	1.15171211		
CAC	ebi-a-GCST90001489	CD39+ activated CD4 regulatory T cell Absolute Count || id:ebi-a-GCST90001489	exposure	Contamination mixture method	5	-0.1684666	0.1887755	0.12347909	-0.53846658	0.20153342	0.8449595	0.58364254	1.22327712		
CAC	ebi-a-GCST90001489	CD39+ activated CD4 regulatory T cell Absolute Count || id:ebi-a-GCST90001489	exposure	Robust adjusted profile score (RAPS)	5	-0.1680244	0.0888203	0.05852631	-0.34211206	0.00606332	0.84533323	0.7102686	1.00608174		
CAC	ebi-a-GCST90001498	Activated & resting CD4 regulatory T cell Absolute Count || id:ebi-a-GCST90001498	exposure	Inverse variance weighted	5	-0.1644308	0.0803643	0.04074968	-0.32194488	-0.0069168	0.84837643	0.72473813	0.99310707	0.828465526	0.757301181
CAC	ebi-a-GCST90001498	Activated & resting CD4 regulatory T cell Absolute Count || id:ebi-a-GCST90001498	exposure	MR Egger	5	-0.1064994	0.2580672	0.70756058	-0.61231116	0.39931231	0.89897557	0.54209655	1.49079914		0.609074717

<table>
<tr><td>CAC</td><td>ebi-a-GCST90001498</td><td>Activated & resting CD4 regulatory T cell Absolute Count || id:ebi-a-GCST90001498</td><td>exposure</td><td>Weighted median</td><td>5</td><td>-0.1602911</td><td>0.0947082</td><td>0.09055563</td><td>-0.34591914</td><td>0.02533689</td><td>0.85189575</td><td>0.7075697</td><td>1.0256606</td><td></td><td></td></tr>
<tr><td>CAC</td><td>ebi-a-GCST90001498</td><td>Activated & resting CD4 regulatory T cell Absolute Count || id:ebi-a-GCST90001498</td><td>exposure</td><td>Weighted mode</td><td>5</td><td>-0.2542644</td><td>0.1125225</td><td>0.08671149</td><td>-0.47480845</td><td>-0.0337203</td><td>0.77548674</td><td>0.62200419</td><td>0.96684186</td><td></td><td></td></tr>
<tr><td>CAC</td><td>ebi-a-GCST90001498</td><td>Activated & resting CD4 regulatory T cell Absolute Count || id:ebi-a-GCST90001498</td><td>exposure</td><td>Contamination mixture method</td><td>5</td><td>-0.24</td><td>0.1530612</td><td>0.113395</td><td>-0.54</td><td>0.06</td><td>0.78662786</td><td>0.58274825</td><td>1.06183655</td><td></td><td></td></tr>
<tr><td>CAC</td><td>ebi-a-GCST90001498</td><td>Activated & resting CD4 regulatory T cell Absolute Count || id:ebi-a-GCST90001498</td><td>exposure</td><td>Robust adjusted profile score (RAPS)</td><td>5</td><td>-0.1661658</td><td>0.0854548</td><td>0.05183671</td><td>-0.3336573</td><td>0.00132563</td><td>0.84690578</td><td>0.71629922</td><td>1.00132651</td><td></td><td></td></tr>
<tr><td>CAC</td><td>ebi-a-GCST90001530</td><td>Monocytic Myeloid-Derived Suppressor Cells Absolute Count || id:ebi-a-GCST90001530</td><td>exposure</td><td>Inverse variance weighted</td><td>5</td><td>0.23781235</td><td>0.1065262</td><td>0.02558653</td><td>0.029020984</td><td>0.44660372</td><td>1.26847115</td><td>1.0294462</td><td>1.56299479</td><td>0.830352027</td><td>0.636107874</td></tr>
<tr><td>CAC</td><td>ebi-a-GCST90001530</td><td>Monocytic Myeloid-Derived Suppressor Cells Absolute Count || id:ebi-a-GCST90001530</td><td>exposure</td><td>MR Egger</td><td>5</td><td>0.31456969</td><td>0.3454657</td><td>0.42965899</td><td>-0.36254317</td><td>0.99168255</td><td>1.36966981</td><td>0.69590427</td><td>2.69576643</td><td></td><td>0.476523438</td></tr>
</table>

CAC	ebi-a-GCST90001530	Monocytic Myeloid-Derived Suppressor Cells Absolute Count || id:ebi-a-GCST90001530	exposure	Weighted median	5	0.2713862	0.1276316	0.03347624	0.021228282	0.52154411	1.31178158	1.0214552	1.6846269		
CAC	ebi-a-GCST90001530	Monocytic Myeloid-Derived Suppressor Cells Absolute Count || id:ebi-a-GCST90001530	exposure	Weighted mode	5	0.3102931	0.1393144	0.08988131	0.03723697	0.58334924	1.3638248	1.03793895	1.79203033		
CAC	ebi-a-GCST90001530	Monocytic Myeloid-Derived Suppressor Cells Absolute Count || id:ebi-a-GCST90001530	exposure	Contamination mixture method	5	0.32437746	0.1887755	0.09790273	-0.04562254	0.69437746	1.3831693	0.95540252	2.00246207		
CAC	ebi-a-GCST90001530	Monocytic Myeloid-Derived Suppressor Cells Absolute Count || id:ebi-a-GCST90001530	exposure	Robust adjusted profile score (RAPS)	5	0.23931934	0.1131404	0.03440931	0.01756416	0.46107452	1.27038416	1.01771932	1.58577702		
CAC	ebi-a-GCST90001551	Naive CD8+ T cell Absolute Count || id:ebi-a-GCST90001551	exposure	Inverse variance weighted	4	-0.199065	0.0615642	0.00122302	-0.31973081	-0.0783993	0.81949659	0.72634454	0.92459518	0.831677755	0.815387722
CAC	ebi-a-GCST90001551	Naive CD8+ T cell Absolute Count || id:ebi-a-GCST90001551	exposure	MR Egger	4	-0.2573603	0.2491257	0.41013592	-0.74564655	0.23092601	0.77308964	0.47442746	1.25976603		0.642991826
CAC	ebi-a-GCST90001551	Naive CD8+ T cell Absolute Count || id:ebi-a-GCST90001551	exposure	Weighted median	4	-0.2196105	0.0724681	0.00244196	-0.36164805	-0.0775729	0.80283145	0.69652747	0.92535955		

CAC	ebi-a-GCST90001551	Naive CD8+ T cell Absolute Count || id:ebi-a-GCST90001551	exposure	Weighted mode	4	-0.2264444	0.0778001	0.06196676	-0.37893259	-0.0739563	0.79736364	0.68459176	0.92871229		
CAC	ebi-a-GCST90001551	Naive CD8+ T cell Absolute Count || id:ebi-a-GCST90001551	exposure	Contamination mixture method	4	-0.2253915	0.0663265	0.01241908	-0.3553915	-0.0953915	0.79820366	0.70089899	0.90901699		
CAC	ebi-a-GCST90001551	Naive CD8+ T cell Absolute Count || id:ebi-a-GCST90001551	exposure	Robust adjusted profile score (RAPS)	4	-0.1997331	0.0661571	0.00253551	-0.32940108	-0.0700651	0.81894929	0.71935444	0.93233309		
CAC	ebi-a-GCST90001552	Naive CD8+ T cell %CD8+ T cell || id:ebi-a-GCST90001552	exposure	Inverse variance weighted	5	-0.1907771	0.0604132	0.00158926	-0.30918707	-0.0723672	0.82631672	0.73404344	0.93018926	0.694317961	0.956516737
CAC	ebi-a-GCST90001552	Naive CD8+ T cell %CD8+ T cell || id:ebi-a-GCST90001552	exposure	MR Egger	5	-0.2705603	0.1939457	0.2573473	-0.65069376	0.10957324	0.76295193	0.52168373	1.11580179		0.925506236
CAC	ebi-a-GCST90001552	Naive CD8+ T cell %CD8+ T cell || id:ebi-a-GCST90001552	exposure	Weighted median	5	-0.1927032	0.073279	0.00854557	-0.33633017	-0.0490763	0.82472668	0.7143872	0.95210848		
CAC	ebi-a-GCST90001552	Naive CD8+ T cell %CD8+ T cell || id:ebi-a-GCST90001552	exposure	Weighted mode	5	-0.1959261	0.088065	0.09013111	-0.36853358	-0.0233187	0.82207296	0.69174798	0.97695109		
CAC	ebi-a-GCST90001552	Naive CD8+ T cell %CD8+ T cell || id:ebi-a-GCST90001552	exposure	Contamination mixture method	5	-0.1956881	0.0663265	0.00625918	-0.32568807	-0.0656881	0.82226868	0.72203037	0.93642291		

CAC	ebi-a-GCST90001552	Naive CD8+ T cell %CD8+ T cell || id:ebi-a-GCST90001552	exposure	Robust adjusted profile score (RAPS)	5	-0.1909767	0.0649044	0.00325648	-0.31818932	-0.063764	0.82615187	0.72746505	0.9382264		
CAC	ebi-a-GCST90001553	Naive CD8+ T cell %T cell || id:ebi-a-GCST90001553	exposure	Inverse variance weighted	4	-0.1576745	0.0617026	0.01060669	-0.27861149	-0.0367375	0.85412777	0.75683389	0.96392916	0.988922775	0.740260878
CAC	ebi-a-GCST90001553	Naive CD8+ T cell %T cell || id:ebi-a-GCST90001553	exposure	MR Egger	4	-0.1614588	0.2493086	0.58364028	-0.65010371	0.32718618	0.85090161	0.52199164	1.38705969		0.534455432
CAC	ebi-a-GCST90001553	Naive CD8+ T cell %T cell || id:ebi-a-GCST90001553	exposure	Weighted median	4	-0.1512792	0.0687898	0.0278672	-0.28610734	-0.0164512	0.85960763	0.75118198	0.98368343		
CAC	ebi-a-GCST90001553	Naive CD8+ T cell %T cell || id:ebi-a-GCST90001553	exposure	Weighted mode	4	-0.1351889	0.083225	0.20275984	-0.29830989	0.02793204	0.87355086	0.74207135	1.0283258		
CAC	ebi-a-GCST90001553	Naive CD8+ T cell %T cell || id:ebi-a-GCST90001553	exposure	Contamination mixture method	4	-0.1561521	0.0637755	0.02658305	-0.28115213	-0.0311521	0.85542905	0.75491349	0.9693281		
CAC	ebi-a-GCST90001553	Naive CD8+ T cell %T cell || id:ebi-a-GCST90001553	exposure	Robust adjusted profile score (RAPS)	4	-0.1584919	0.0657766	0.0159723	-0.28741406	-0.0295697	0.85342988	0.75020104	0.97086317		

CAC	ebi-a-GCST90001555	Effector Memory CD8+ T cell %CD8+ T cell || id:ebi-a-GCST90001555	exposure	Inverse variance weighted	5	0.213269	0.0689375	0.00197707	0.078151431	0.34838658	1.23771756	1.08128639	1.41677984	0.719352889	0.855942347
CAC	ebi-a-GCST90001555	Effector Memory CD8+ T cell %CD8+ T cell || id:ebi-a-GCST90001555	exposure	MR Egger	5	0.12992457	0.2220518	0.59957378	-0.30529699	0.56514613	1.13874248	0.73690449	1.75970491		0.758769961
CAC	ebi-a-GCST90001555	Effector Memory CD8+ T cell %CD8+ T cell || id:ebi-a-GCST90001555	exposure	Weighted median	5	0.19874936	0.0844093	0.01854306	0.033307205	0.36419152	1.21987618	1.0338681	1.43934986		
CAC	ebi-a-GCST90001555	Effector Memory CD8+ T cell %CD8+ T cell || id:ebi-a-GCST90001555	exposure	Weighted mode	5	0.28555054	0.097274	0.04257843	0.094893517	0.47620756	1.33049431	1.09954177	1.60995714		
CAC	ebi-a-GCST90001555	Effector Memory CD8+ T cell %CD8+ T cell || id:ebi-a-GCST90001555	exposure	Contamination mixture method	5	0.23259502	0.0816327	0.01489808	0.07259502	0.39259502	1.26187034	1.07529498	1.48081857		
CAC	ebi-a-GCST90001555	Effector Memory CD8+ T cell %CD8+ T cell || id:ebi-a-GCST90001555	exposure	Robust adjusted profile score (RAPS)	5	0.21415471	0.0740855	0.00384454	0.06894717	0.35936225	1.2388143	1.07137961	1.4324156		
CAC	ebi-a-GCST90001560	CD45RA+ CD8+ T cell Absolute Count || id:ebi-a-GCST90001560	exposure	Inverse variance weighted	5	-0.2340732	0.0693786	0.00074124	-0.37005524	-0.0980912	0.79130387	0.69069617	0.90656622	0.687901055	0.571235305
CAC	ebi-a-GCST90001560	CD45RA+ CD8+ T cell Absolute Count || id:ebi-a-GCST90001560	exposure	MR Egger	5	-0.139843	0.2238352	0.57642106	-0.57855993	0.29887403	0.86949478	0.56070524	1.34833976		0.436108747

CAC	ebi-a-GCST90001560	CD45RA+ CD8+ T cell Absolute Count || id:ebi-a-GCST90001560	exposure	Weighted median	5	-0.2628221	0.08564	0.00214837	-0.43067658	-0.0949676	0.76887867	0.65006912	0.90940239		
CAC	ebi-a-GCST90001560	CD45RA+ CD8+ T cell Absolute Count || id:ebi-a-GCST90001560	exposure	Weighted mode	5	-0.2906846	0.1017699	0.04610168	-0.49015352	-0.0912157	0.74775148	0.61253235	0.91282081		
CAC	ebi-a-GCST90001560	CD45RA+ CD8+ T cell Absolute Count || id:ebi-a-GCST90001560	exposure	Contamination mixture method	5	-0.2719239	0.0867347	0.01840238	-0.44192394	-0.1019239	0.76191221	0.64279853	0.90309824		
CAC	ebi-a-GCST90001560	CD45RA+ CD8+ T cell Absolute Count || id:ebi-a-GCST90001560	exposure	Robust adjusted profile score (RAPS)	5	-0.2361062	0.0745444	0.00153848	-0.38221318	-0.0899993	0.78969679	0.68234958	0.91393186		
CAC	ebi-a-GCST90001561	CD45RA+ CD8+ T cell %CD8+ T cell || id:ebi-a-GCST90001561	exposure	Inverse variance weighted	5	-0.2354177	0.0691725	0.00066567	-0.37099589	-0.0998395	0.7902407	0.69004678	0.90498265	0.622532619	0.767114911
CAC	ebi-a-GCST90001561	CD45RA+ CD8+ T cell %CD8+ T cell || id:ebi-a-GCST90001561	exposure	MR Egger	5	-0.1195998	0.2228009	0.62866145	-0.55628957	0.31708999	0.88727546	0.57333243	1.37312613		0.675301948
CAC	ebi-a-GCST90001561	CD45RA+ CD8+ T cell %CD8+ T cell || id:ebi-a-GCST90001561	exposure	Weighted median	5	-0.2356627	0.0850259	0.00557717	-0.4023135	-0.0690119	0.7900471	0.66877105	0.93331555		
CAC	ebi-a-GCST90001561	CD45RA+ CD8+ T cell %CD8+ T cell || id:ebi-a-GCST90001561	exposure	Weighted mode	5	-0.3212092	0.0997558	0.0322819	-0.5167306	-0.1256878	0.7252715	0.59646745	0.88189011		

CAC	ebi-a-GCST90001561	CD45RA+ CD8+ T cell %CD8+ T cell || id:ebi-a-GCST90001561	exposure	Contamination mixture method	5	-0.2515884	0.0816327	0.01228659	-0.41158837	-0.0915884	0.77756474	0.66259697	0.91248068		
CAC	ebi-a-GCST90001561	CD45RA+ CD8+ T cell %CD8+ T cell || id:ebi-a-GCST90001561	exposure	Robust adjusted profile score (RAPS)	5	-0.2366607	0.0745604	0.00150311	-0.38279905	-0.0905224	0.78925902	0.68194992	0.91345387		
CAC	ebi-a-GCST90001562	CD45RA+ CD8+ T cell %T cell || id:ebi-a-GCST90001562	exposure	Inverse variance weighted	5	-0.1981891	0.0717358	0.00573142	-0.33879122	-0.0575869	0.82021476	0.71263122	0.94403984	0.837387212	0.552327487
CAC	ebi-a-GCST90001562	CD45RA+ CD8+ T cell %T cell || id:ebi-a-GCST90001562	exposure	MR Egger	5	-0.1490903	0.2309481	0.56454149	-0.60174846	0.30356791	0.86149134	0.5478529	1.35468358		0.394266005
CAC	ebi-a-GCST90001562	CD45RA+ CD8+ T cell %T cell || id:ebi-a-GCST90001562	exposure	Weighted median	5	-0.2001442	0.086209	0.02025368	-0.36911386	-0.0311745	0.81861273	0.69134669	0.96930645		
CAC	ebi-a-GCST90001562	CD45RA+ CD8+ T cell %T cell || id:ebi-a-GCST90001562	exposure	Weighted mode	5	-0.2070191	0.1008729	0.10940723	-0.40473011	-0.0093082	0.8130041	0.66715685	0.99073504		
CAC	ebi-a-GCST90001562	CD45RA+ CD8+ T cell %T cell || id:ebi-a-GCST90001562	exposure	Contamination mixture method	5	-0.2401048	0.0816327	0.01269403	-0.40010485	-0.0801048	0.78654539	0.67024977	0.92301956		
CAC	ebi-a-GCST90001562	CD45RA+ CD8+ T cell %T cell || id:ebi-a-GCST90001562	exposure	Robust adjusted profile	5	-0.2022479	0.076552	0.00824262	-0.35228995	-0.0522059	0.81689236	0.70307624	0.94913338		

				score (RAPS)											
CAC	ebi-a-GCST90001563	Central Memory CD4-CD8- T cell Absolute Count || id:ebi-a-GCST90001563	exposure	Inverse variance weighted	5	-0.1735125	0.0679181	0.01062703	-0.30663208	-0.0403929	0.84070663	0.73592131	0.96041197	0.725821648	0.677992043
CAC	ebi-a-GCST90001563	Central Memory CD4-CD8- T cell Absolute Count || id:ebi-a-GCST90001563	exposure	MR Egger	5	-0.0931303	0.2194852	0.69992739	-0.52332138	0.33706069	0.91107474	0.5925492	1.40082407		0.53849095
CAC	ebi-a-GCST90001563	Central Memory CD4-CD8- T cell Absolute Count || id:ebi-a-GCST90001563	exposure	Weighted median	5	-0.1992103	0.0858516	0.02031903	-0.36747938	-0.0309413	0.81937755	0.69247761	0.96953253		
CAC	ebi-a-GCST90001563	Central Memory CD4-CD8- T cell Absolute Count || id:ebi-a-GCST90001563	exposure	Weighted mode	5	-0.059546	0.0949309	0.56451686	-0.24561062	0.12651868	0.94219222	0.78222675	1.13487065		
CAC	ebi-a-GCST90001563	Central Memory CD4-CD8- T cell Absolute Count || id:ebi-a-GCST90001563	exposure	Contamination mixture method	5	-0.2592005	0.0969388	0.01389659	-0.44920052	-0.0692005	0.77166827	0.63813812	0.93313954		
CAC	ebi-a-GCST90001563	Central Memory CD4-CD8- T cell Absolute Count || id:ebi-a-GCST90001563	exposure	Robust adjusted profile score (RAPS)	5	-0.1749065	0.0723385	0.01561091	-0.31669	-0.0331229	0.83953555	0.72855657	0.96741966		
CAC	ebi-a-GCST90001570	Effector Memory CD4-CD8- T cell %CD4-CD8- T cell || id:ebi-a-GCST90001570	exposure	Inverse variance weighted	5	0.22225434	0.0811586	0.00617168	0.063183452	0.38132522	1.24888897	1.06522224	1.46422372	0.526407012	0.512862991

CAC	ebi-a-GCST90001570	Effector Memory CD4-CD8- T cell %CD4-CD8- T cell || id:ebi-a-GCST90001570	exposure	MR Egger	5	0.04424374	0.2619703	0.87662954	-0.4692181	0.55770559	1.04523709	0.62549115	1.74666035		0.429376886
CAC	ebi-a-GCST90001570	Effector Memory CD4-CD8- T cell %CD4-CD8- T cell || id:ebi-a-GCST90001570	exposure	Weighted median	5	0.23621559	0.1034742	0.0224395	0.033406128	0.43902505	1.26644731	1.03397038	1.55119414		
CAC	ebi-a-GCST90001570	Effector Memory CD4-CD8- T cell %CD4-CD8- T cell || id:ebi-a-GCST90001570	exposure	Weighted mode	5	0.10942976	0.1177149	0.40519098	-0.12129154	0.34015105	1.1156417	0.88577568	1.40515982		
CAC	ebi-a-GCST90001570	Effector Memory CD4-CD8- T cell %CD4-CD8- T cell || id:ebi-a-GCST90001570	exposure	Contamination mixture method	5	0.33908277	0.1352041	0.01686895	0.074082774	0.60408277	1.40365953	1.07689594	1.82957331		
CAC	ebi-a-GCST90001570	Effector Memory CD4-CD8- T cell %CD4-CD8- T cell || id:ebi-a-GCST90001570	exposure	Robust adjusted profile score (RAPS)	5	0.22478993	0.0864712	0.00933343	0.055306386	0.39427348	1.25205967	1.05686437	1.48330614		
CAC	ebi-a-GCST90001572	Terminally Differentiated CD4-CD8- T cell Absolute Count || id:ebi-a-GCST90001572	exposure	Inverse variance weighted	5	-0.1918452	0.0808511	0.01765274	-0.3503133	-0.0333771	0.82543462	0.70446735	0.96717373	0.630075441	0.654516567

CAC	ebi-a-GCST90001572	Terminally Differentiated CD4-CD8- T cell Absolute Count || id:ebi-a-GCST90001572	exposure	MR Egger	5	-0.3241449	0.260394	0.30157903	-0.83451718	0.18622742	0.72314547	0.43408402	1.20469621		0.540012275
CAC	ebi-a-GCST90001572	Terminally Differentiated CD4-CD8- T cell Absolute Count || id:ebi-a-GCST90001572	exposure	Weighted median	5	-0.1517799	0.1025935	0.13902523	-0.35286316	0.04930327	0.85917733	0.70267334	1.0505389		
CAC	ebi-a-GCST90001572	Terminally Differentiated CD4-CD8- T cell Absolute Count || id:ebi-a-GCST90001572	exposure	Weighted mode	5	-0.1501686	0.1180024	0.27210446	-0.38145337	0.08111623	0.8605629	0.68286823	1.08449694		
CAC	ebi-a-GCST90001572	Terminally Differentiated CD4-CD8- T cell Absolute Count || id:ebi-a-GCST90001572	exposure	Contamination mixture method	5	-0.2231848	0.1479592	0.05464075	-0.5131848	0.0668152	0.799967	0.59858616	1.06909789		
CAC	ebi-a-GCST90001572	Terminally Differentiated CD4-CD8- T cell Absolute Count || id:ebi-a-GCST90001572	exposure	Robust adjusted profile score (RAPS)	5	-0.193391	0.0856579	0.02396348	-0.36128049	-0.0255016	0.82415964	0.69678353	0.97482085		
CAC	ebi-a-GCST90001602	Lymphocyte %leukocyte || id:ebi-a-GCST90001602	exposure	Inverse variance weighted	5	-0.1941532	0.0833988	0.01991151	-0.35761481	-0.0306917	0.8235317	0.6993424	0.96977454	0.973084939	0.361742366
CAC	ebi-a-GCST90001602	Lymphocyte %leukocyte || id:ebi-a-GCST90001602	exposure	MR Egger	5	-0.1833884	0.3092905	0.59495344	-0.78959776	0.42282105	0.83244481	0.45402739	1.52626115		0.227044057

CAC	ebi-a-GCST90001602	Lymphocyte %leukocyte || id:ebi-a-GCST90001602	exposure	Weighted median	5	-0.2291377	0.1062657	0.03106247	-0.43741847	-0.0208569	0.79521902	0.64570117	0.97935908		
CAC	ebi-a-GCST90001602	Lymphocyte %leukocyte || id:ebi-a-GCST90001602	exposure	Weighted mode	5	-0.3581217	0.1265148	0.04731285	-0.60609069	-0.1101527	0.698988	0.54547915	0.89569734		
CAC	ebi-a-GCST90001602	Lymphocyte %leukocyte || id:ebi-a-GCST90001602	exposure	Contamination mixture method	5	-0.3443848	0.1811224	0.18756495	-0.69938479	0.01061521	0.70865619	0.4968909	1.01067175		
CAC	ebi-a-GCST90001602	Lymphocyte %leukocyte || id:ebi-a-GCST90001602	exposure	Robust adjusted profile score (RAPS)	5	-0.1930989	0.0884446	0.02901546	-0.36645031	-0.0197476	0.8244004	0.69319058	0.98044612		
CAC	ebi-a-GCST90001605	T cell %leukocyte || id:ebi-a-GCST90001605	exposure	Inverse variance weighted	5	-0.2198929	0.0961832	0.02224329	-0.40841196	-0.0313738	0.80260478	0.66470499	0.96911328	0.640952216	0.189984431
CAC	ebi-a-GCST90001605	T cell %leukocyte || id:ebi-a-GCST90001605	exposure	MR Egger	5	-0.0519406	0.3419409	0.88890669	-0.72214464	0.61826353	0.9493853	0.48570947	1.85570287		0.131380178
CAC	ebi-a-GCST90001605	T cell %leukocyte || id:ebi-a-GCST90001605	exposure	Weighted median	5	-0.2960246	0.1079469	0.00610075	-0.50760057	-0.0844487	0.74376911	0.60193816	0.91901881		
CAC	ebi-a-GCST90001605	T cell %leukocyte || id:ebi-a-GCST90001605	exposure	Weighted mode	5	-0.3618121	0.1299728	0.04962772	-0.61655872	-0.1070655	0.69641321	0.53979885	0.89846684		

CAC	ebi-a-GCST90001605	T cell %leukocyte || id:ebi-a-GCST90001605	exposure	Contamination mixture method	5	-0.3923043	0.1887755	0.19731564	-0.76230425	-0.0223043	0.67549856	0.46659005	0.97794265		
CAC	ebi-a-GCST90001605	T cell %leukocyte || id:ebi-a-GCST90001605	exposure	Robust adjusted profile score (RAPS)	5	-0.1939322	0.0984887	0.04894394	-0.38697014	-0.0008942	0.82371376	0.67911137	0.99910615		
CAC	ebi-a-GCST90001615	TCRgd T cell Absolute Count || id:ebi-a-GCST90001615	exposure	Inverse variance weighted	5	-0.166203	0.0774465	0.03187004	-0.31799813	-0.0144079	0.84687429	0.72760415	0.98569539	0.564719976	0.71107539
CAC	ebi-a-GCST90001615	TCRgd T cell Absolute Count || id:ebi-a-GCST90001615	exposure	MR Egger	5	-0.3194805	0.249856	0.29096298	-0.80919827	0.17023725	0.72652637	0.44521487	1.1855861		0.632949883
CAC	ebi-a-GCST90001615	TCRgd T cell Absolute Count || id:ebi-a-GCST90001615	exposure	Weighted median	5	-0.1739566	0.0943716	0.06528312	-0.35892495	0.01101181	0.84033339	0.69842676	1.01107267		
CAC	ebi-a-GCST90001615	TCRgd T cell Absolute Count || id:ebi-a-GCST90001615	exposure	Weighted mode	5	-0.1736973	0.1123763	0.19707765	-0.39395479	0.04656023	0.84055131	0.67438454	1.04766117		
CAC	ebi-a-GCST90001615	TCRgd T cell Absolute Count || id:ebi-a-GCST90001615	exposure	Contamination mixture method	5	-0.1985845	0.1147959	0.03888268	-0.42358453	0.02641547	0.81989046	0.65469583	1.02676745		
CAC	ebi-a-GCST90001615	TCRgd T cell Absolute Count || id:ebi-a-GCST90001615	exposure	Robust adjusted profile	5	-0.1673398	0.0821735	0.0417081	-0.32839989	-0.0062798	0.84591209	0.72007501	0.9937399		

				score (RAPS)											
CAC	ebi-a-GCST90001624	HLA DR+ CD4+ T cell Absolute Count || id:ebi-a-GCST90001624	exposure	Inverse variance weighted	5	-0.1889547	0.0777366	0.01506946	-0.34131831	-0.036591	0.82782403	0.71083261	0.96407034	0.935967491	0.953644887
CAC	ebi-a-GCST90001624	HLA DR+ CD4+ T cell Absolute Count || id:ebi-a-GCST90001624	exposure	MR Egger	5	-0.1681824	0.250437	0.54995315	-0.65903903	0.32267418	0.84519963	0.51734825	1.38081538		0.879432939
CAC	ebi-a-GCST90001624	HLA DR+ CD4+ T cell Absolute Count || id:ebi-a-GCST90001624	exposure	Weighted median	5	-0.1718228	0.0941657	0.06804835	-0.35638755	0.01274188	0.84212836	0.7002012	1.01282341		
CAC	ebi-a-GCST90001624	HLA DR+ CD4+ T cell Absolute Count || id:ebi-a-GCST90001624	exposure	Weighted mode	5	-0.2424113	0.1119271	0.09626203	-0.46178834	-0.0230342	0.78473337	0.6301557	0.97722906		
CAC	ebi-a-GCST90001624	HLA DR+ CD4+ T cell Absolute Count || id:ebi-a-GCST90001624	exposure	Contamination mixture method	5	-0.192595	0.0994898	0.0353829	-0.38759502	0.00240498	0.82481594	0.67868714	1.00240787		
CAC	ebi-a-GCST90001624	HLA DR+ CD4+ T cell Absolute Count || id:ebi-a-GCST90001624	exposure	Robust adjusted profile score (RAPS)	5	-0.1893532	0.0830106	0.02254411	-0.35205396	-0.0266525	0.82749416	0.70324217	0.97369956		
CAC	ebi-a-GCST90001626	HLA DR+ CD4+ T cell %lymphocyte || id:ebi-a-GCST90001626	exposure	Inverse variance weighted	5	-0.1465185	0.0711853	0.0395644	-0.28604165	-0.0069952	0.8637098	0.75123132	0.99302916	0.693408757	0.952163331

CAC	ebi-a-GCST90001626	HLA DR+ CD4+ T cell %lymphocyte || id:ebi-a-GCST90001626	exposure	MR Egger	5	-0.0515133	0.230044	0.83719651	-0.50239953	0.39937297	0.94979104	0.60507701	1.49088958		0.917881196
CAC	ebi-a-GCST90001626	HLA DR+ CD4+ T cell %lymphocyte || id:ebi-a-GCST90001626	exposure	Weighted median	5	-0.1205284	0.0872286	0.16704774	-0.29149654	0.05043976	0.88645192	0.7471446	1.0517335		
CAC	ebi-a-GCST90001626	HLA DR+ CD4+ T cell %lymphocyte || id:ebi-a-GCST90001626	exposure	Weighted mode	5	-0.1185986	0.1015997	0.30792345	-0.31773405	0.08053679	0.88816421	0.72779632	1.08386872		
CAC	ebi-a-GCST90001626	HLA DR+ CD4+ T cell %lymphocyte || id:ebi-a-GCST90001626	exposure	Contamination mixture method	5	-0.1507602	0.1122449	0.04752084	-0.37076016	0.06923984	0.86005395	0.69020946	1.07169322		
CAC	ebi-a-GCST90001626	HLA DR+ CD4+ T cell %lymphocyte || id:ebi-a-GCST90001626	exposure	Robust adjusted profile score (RAPS)	5	-0.1467492	0.0756624	0.05243729	-0.29504749	0.00154909	0.86351052	0.74449623	1.00155029		
CAC	ebi-a-GCST90001666	CD28+ CD45RA+ CD8dim T cell Absolute Count || id:ebi-a-GCST90001666	exposure	Inverse variance weighted	5	-0.1649312	0.0711584	0.02046002	-0.30440176	-0.0254607	0.84795201	0.73756448	0.97486068	0.616881877	0.703754904
CAC	ebi-a-GCST90001666	CD28+ CD45RA+ CD8dim T cell Absolute Count || id:ebi-a-GCST90001666	exposure	MR Egger	5	-0.2861787	0.229322	0.3005868	-0.73564971	0.16329234	0.75112839	0.47919402	1.17738083		0.600920801

CAC	ebi-a-GCST90001666	CD28+ CD45RA+ CD8dim T cell Absolute Count || id:ebi-a-GCST90001666	exposure	Weighted median	5	-0.091522	0.0901744	0.31013246	-0.26826382	0.08521985	0.91254125	0.76470601	1.08895645		
CAC	ebi-a-GCST90001666	CD28+ CD45RA+ CD8dim T cell Absolute Count || id:ebi-a-GCST90001666	exposure	Weighted mode	5	-0.083448	0.1009911	0.45507568	-0.28139056	0.11449454	0.91993891	0.75473351	1.12130651		
CAC	ebi-a-GCST90001666	CD28+ CD45RA+ CD8dim T cell Absolute Count || id:ebi-a-GCST90001666	exposure	Contamination mixture method	5	-0.1643119	0.127551	0.04751471	-0.41431193	0.08568807	0.84847732	0.6607948	1.08946644		
CAC	ebi-a-GCST90001666	CD28+ CD45RA+ CD8dim T cell Absolute Count || id:ebi-a-GCST90001666	exposure	Robust adjusted profile score (RAPS)	5	-0.166129	0.0755928	0.02797164	-0.3142909	-0.0179672	0.84693693	0.73030655	0.98219326		
CAC	ebi-a-GCST90001688	CD28+ CD45RA+ CD8+ T cell %T cell || id:ebi-a-GCST90001688	exposure	Inverse variance weighted	5	-0.2062551	0.0566404	0.00027107	-0.31727026	-0.0952399	0.8136255	0.72813394	0.90915479	0.664943999	0.93230823
CAC	ebi-a-GCST90001688	CD28+ CD45RA+ CD8+ T cell %T cell || id:ebi-a-GCST90001688	exposure	MR Egger	5	-0.2891252	0.182182	0.21070501	-0.64620183	0.06795148	0.74891846	0.52403237	1.07031338		0.892759893
CAC	ebi-a-GCST90001688	CD28+ CD45RA+ CD8+ T cell %T cell || id:ebi-a-GCST90001688	exposure	Weighted median	5	-0.1995887	0.0683686	0.00350813	-0.33359108	-0.0655863	0.81906757	0.71634665	0.93651822		

CAC	ebi-a-GCST90001688	CD28+ CD45RA+ CD8+ T cell %T cell || id:ebi-a-GCST90001688	exposure	Weighted mode	5	-0.1819089	0.0786192	0.08169083	-0.33600248	-0.0278153	0.83367729	0.71462133	0.97256798		
CAC	ebi-a-GCST90001688	CD28+ CD45RA+ CD8+ T cell %T cell || id:ebi-a-GCST90001688	exposure	Contamination mixture method	5	-0.2019004	0.0637755	0.00178013	-0.32690039	-0.0769004	0.81717632	0.72115557	0.92598208		
CAC	ebi-a-GCST90001688	CD28+ CD45RA+ CD8+ T cell %T cell || id:ebi-a-GCST90001688	exposure	Robust adjusted profile score (RAPS)	5	-0.2065988	0.0612182	0.00073871	-0.32658654	-0.0866111	0.81334587	0.72138195	0.91703364		
CAC	ebi-a-GCST90001689	CD28+ CD45RA+ CD8+ T cell %CD8+ T cell || id:ebi-a-GCST90001689	exposure	Inverse variance weighted	5	-0.217013	0.0609885	0.00037332	-0.33655049	-0.0974756	0.80491948	0.71422982	0.9071245	0.816638381	0.879704526
CAC	ebi-a-GCST90001689	CD28+ CD45RA+ CD8+ T cell %CD8+ T cell || id:ebi-a-GCST90001689	exposure	MR Egger	5	-0.2642753	0.1965353	0.27135983	-0.64948456	0.12093395	0.76776214	0.52231493	1.12855037		0.770744048
CAC	ebi-a-GCST90001689	CD28+ CD45RA+ CD8+ T cell %CD8+ T cell || id:ebi-a-GCST90001689	exposure	Weighted median	5	-0.2389239	0.0737565	0.00119798	-0.38348665	-0.0943612	0.7874748	0.68148118	0.90995404		
CAC	ebi-a-GCST90001689	CD28+ CD45RA+ CD8+ T cell %CD8+ T cell || id:ebi-a-GCST90001689	exposure	Weighted mode	5	-0.2490632	0.0842313	0.04168273	-0.41415656	-0.0839698	0.77953071	0.66089747	0.919459		
CAC	ebi-a-GCST90001689	CD28+ CD45RA+ CD8+ T cell %CD8+ T cell || id:ebi-a-GCST90001689	exposure	Contamination mixture method	5	-0.2176933	0.0714286	0.00637679	-0.35769332	-0.0776933	0.80437209	0.6992875	0.92524814		

CAC	ebi-a-GCST90001689	CD28+ CD45RA+ CD8+ T cell %CD8+ T cell || id:ebi-a-GCST90001689	exposure	Robust adjusted profile score (RAPS)	5	-0.2178207	0.0659143	0.00095111	-0.34701274	-0.0886286	0.80426964	0.70679632	0.91518538		
CAC	ebi-a-GCST90001690	CD28+ CD45RA+ CD8+ T cell Absolute Count || id:ebi-a-GCST90001690	exposure	Inverse variance weighted	5	-0.2320936	0.0566731	4.22E-05	-0.34317286	-0.1210144	0.79287187	0.70951555	0.88602117	0.776988392	0.915729074
CAC	ebi-a-GCST90001690	CD28+ CD45RA+ CD8+ T cell Absolute Count || id:ebi-a-GCST90001690	exposure	MR Egger	5	-0.2858322	0.1824874	0.21525597	-0.64350746	0.07184307	0.75138869	0.5254462	1.07448671		0.833985234
CAC	ebi-a-GCST90001690	CD28+ CD45RA+ CD8+ T cell Absolute Count || id:ebi-a-GCST90001690	exposure	Weighted median	5	-0.2570351	0.0677319	0.00014771	-0.38978958	-0.1242807	0.77334103	0.67719935	0.8831319		
CAC	ebi-a-GCST90001690	CD28+ CD45RA+ CD8+ T cell Absolute Count || id:ebi-a-GCST90001690	exposure	Weighted mode	5	-0.2597996	0.0817117	0.03355379	-0.41995105	-0.099641	0.77120888	0.65707899	0.90516231		
CAC	ebi-a-GCST90001690	CD28+ CD45RA+ CD8+ T cell Absolute Count || id:ebi-a-GCST90001690	exposure	Contamination mixture method	5	-0.2475229	0.0586735	0.00133517	-0.36252294	-0.1325229	0.78073231	0.69591835	0.87588284		
CAC	ebi-a-GCST90001690	CD28+ CD45RA+ CD8+ T cell Absolute Count || id:ebi-a-GCST90001690	exposure	Robust adjusted profile score (RAPS)	5	-0.2324793	0.0616795	0.0001638	-0.35337107	-0.1115876	0.79256613	0.70231654	0.89441304		

CAC	ebi-a-GCST90001763	CD20 on transitional B cell || id:ebi-a-GCST90001763	exposure	Inverse variance weighted	5	-0.1829964	0.0777503	0.01859047	-0.33538702	-0.0306058	0.83277113	0.71506129	0.96985778	0.300589032	0.468597624
CAC	ebi-a-GCST90001763	CD20 on transitional B cell || id:ebi-a-GCST90001763	exposure	MR Egger	5	-0.4817286	0.2516927	0.15153519	-0.9750462	0.0115899	0.61771469	0.37717493	1.01165641		0.571551989
CAC	ebi-a-GCST90001763	CD20 on transitional B cell || id:ebi-a-GCST90001763	exposure	Weighted median	5	-0.1718128	0.0957764	0.07283005	-0.35953464	0.01590902	0.8421368	0.69800107	1.01603624		
CAC	ebi-a-GCST90001763	CD20 on transitional B cell || id:ebi-a-GCST90001763	exposure	Weighted mode	5	-0.1713985	0.11186	0.20022365	-0.39064402	0.04784701	0.84248577	0.67662098	1.04901016		
CAC	ebi-a-GCST90001763	CD20 on transitional B cell || id:ebi-a-GCST90001763	exposure	Contamination mixture method	5	-0.2309043	0.1428571	0.04209781	-0.51090433	0.04909567	0.79381541	0.59995278	1.05032084		
CAC	ebi-a-GCST90001763	CD20 on transitional B cell || id:ebi-a-GCST90001763	exposure	Robust adjusted profile score (RAPS)	5	-0.1855093	0.0828777	0.02519817	-0.3479496	-0.0230691	0.83068107	0.70613446	0.97719496		
CAC	ebi-a-GCST90001781	CD25 on IgD+ CD38- naive B cell || id:ebi-a-GCST90001781	exposure	Inverse variance weighted	5	0.21947555	0.1068638	0.03999618	0.010022573	0.42892853	1.2454234	1.01007297	1.53561128	0.460876127	0.911657669
CAC	ebi-a-GCST90001781	CD25 on IgD+ CD38- naive B cell || id:ebi-a-GCST90001781	exposure	MR Egger	5	0.49590204	0.3446844	0.24582101	-0.1796793	1.17148339	1.64197871	0.83553813	3.22677564		0.964452775

CAC	ebi-a-GCST90001781	CD25 on IgD+ CD38- naive B cell || id:ebi-a-GCST90001781	exposure	Weighted median	5	0.20843468	0.1361331	0.12574228	-0.05838615	0.4752555	1.23174846	0.94328563	1.60842511		
CAC	ebi-a-GCST90001781	CD25 on IgD+ CD38- naive B cell || id:ebi-a-GCST90001781	exposure	Weighted mode	5	0.16910666	0.1550992	0.3368553	-0.13488778	0.4731011	1.18424644	0.87381396	1.60496363		
CAC	ebi-a-GCST90001781	CD25 on IgD+ CD38- naive B cell || id:ebi-a-GCST90001781	exposure	Contamination mixture method	5	0.22246085	0.1683673	0.05200639	-0.10753915	0.55246085	1.24914691	0.89804136	1.73752355		
CAC	ebi-a-GCST90001781	CD25 on IgD+ CD38- naive B cell || id:ebi-a-GCST90001781	exposure	Robust adjusted profile score (RAPS)	5	0.22042046	0.1136414	0.05242665	-0.00231673	0.44315765	1.24660077	0.99768595	1.55761787		
CAC	ebi-a-GCST90001788	CD25 on IgD- CD38+ B cell || id:ebi-a-GCST90001788	exposure	Inverse variance weighted	5	0.17404323	0.0765786	0.02304186	0.023949176	0.32413727	1.19010701	1.02423826	1.38283712	0.264585435	0.725097307
CAC	ebi-a-GCST90001788	CD25 on IgD- CD38+ B cell || id:ebi-a-GCST90001788	exposure	MR Egger	5	0.49705399	0.248118	0.13888164	0.010742778	0.9833652	1.64387127	1.01080069	2.67343778		0.980015242
CAC	ebi-a-GCST90001788	CD25 on IgD- CD38+ B cell || id:ebi-a-GCST90001788	exposure	Weighted median	5	0.15994108	0.0936572	0.08768675	-0.02362705	0.34350922	1.17344173	0.97664988	1.40988652		
CAC	ebi-a-GCST90001788	CD25 on IgD- CD38+ B cell || id:ebi-a-GCST90001788	exposure	Weighted mode	5	0.16092573	0.1109631	0.22059407	-0.05656189	0.37841335	1.17459773	0.94500799	1.4599663		

CAC	ebi-a-GCST90001788	CD25 on IgD- CD38+ B cell || id:ebi-a-GCST90001788	exposure	Contamination mixture method	5	0.20731544	0.130102	0.03928806	-0.04768456	0.46231544	1.23037061	0.95343449	1.58774606		
CAC	ebi-a-GCST90001788	CD25 on IgD- CD38+ B cell || id:ebi-a-GCST90001788	exposure	Robust adjusted profile score (RAPS)	5	0.17566912	0.0813828	0.0308848	0.016158863	0.33517937	1.19204357	1.01629012	1.39819116		
CAC	ebi-a-GCST90001803	CD27 on IgD- CD38+ B cell || id:ebi-a-GCST90001803	exposure	Inverse variance weighted	5	-0.1786386	0.0778807	0.02180503	-0.33128477	-0.0259925	0.83640811	0.71800067	0.97434244	0.771238593	0.950976338
CAC	ebi-a-GCST90001803	CD27 on IgD- CD38+ B cell || id:ebi-a-GCST90001803	exposure	MR Egger	5	-0.2550564	0.2525116	0.38685593	-0.74997908	0.23986628	0.77487279	0.47237644	1.27107917		0.896056394
CAC	ebi-a-GCST90001803	CD27 on IgD- CD38+ B cell || id:ebi-a-GCST90001803	exposure	Weighted median	5	-0.1631837	0.0923584	0.07725296	-0.34420617	0.01783881	0.84943515	0.70878278	1.01799887		
CAC	ebi-a-GCST90001803	CD27 on IgD- CD38+ B cell || id:ebi-a-GCST90001803	exposure	Weighted mode	5	-0.1577886	0.1088462	0.22075494	-0.37112724	0.05554998	0.85403028	0.68995614	1.05712185		
CAC	ebi-a-GCST90001803	CD27 on IgD- CD38+ B cell || id:ebi-a-GCST90001803	exposure	Contamination mixture method	5	-0.1759895	0.1122449	0.0288435	-0.39598952	0.04401048	0.83862678	0.67301375	1.04499331		
CAC	ebi-a-GCST90001803	CD27 on IgD- CD38+ B cell || id:ebi-a-GCST90001803	exposure	Robust adjusted profile	5	-0.1791485	0.0830691	0.03103521	-0.3419639	-0.0163331	0.83598174	0.71037385	0.98379954		

				score (RAPS)											
CAC	ebi-a-GCST90001814	CD38 on IgD- CD38+ B cell || id:ebi-a-GCST90001814	exposure	Inverse variance weighted	5	-0.1711221	0.0759712	0.02429317	-0.32002568	-0.0222186	0.84271864	0.72613039	0.97802642	0.461644637	0.552839862
CAC	ebi-a-GCST90001814	CD38 on IgD- CD38+ B cell || id:ebi-a-GCST90001814	exposure	MR Egger	5	0.02600013	0.2461471	0.92254389	-0.45644826	0.50844852	1.02634108	0.63352979	1.66270953		0.508507782
CAC	ebi-a-GCST90001814	CD38 on IgD- CD38+ B cell || id:ebi-a-GCST90001814	exposure	Weighted median	5	-0.1271615	0.0926481	0.16990115	-0.3087517	0.05442875	0.88059147	0.73436309	1.05593724		
CAC	ebi-a-GCST90001814	CD38 on IgD- CD38+ B cell || id:ebi-a-GCST90001814	exposure	Weighted mode	5	-0.0708333	0.1083884	0.54908899	-0.28327428	0.14160825	0.93161745	0.75331314	1.15212521		
CAC	ebi-a-GCST90001814	CD38 on IgD- CD38+ B cell || id:ebi-a-GCST90001814	exposure	Contamination mixture method	5	-0.2591355	0.1530612	0.09203797	-0.55913499	0.04086501	0.77171884	0.57170338	1.04171147		
CAC	ebi-a-GCST90001814	CD38 on IgD- CD38+ B cell || id:ebi-a-GCST90001814	exposure	Robust adjusted profile score (RAPS)	5	-0.1713283	0.0804035	0.03310088	-0.32891915	-0.0137375	0.84254488	0.7197012	0.98635639		
CAC	ebi-a-GCST90001823	IgD on IgD+ CD38- unswitched memory B cell || id:ebi-a-GCST90001823	exposure	Inverse variance weighted	5	0.27077428	0.1067782	0.01121724	0.061489004	0.48005955	1.31097912	1.0634188	1.61617064	0.667884951	0.793746567

CAC	ebi-a-GCST90001823	IgD on IgD+ CD38- unswitched memory B cell || id:ebi-a-GCST90001823	exposure	MR Egger	5	0.11533088	0.3449088	0.76010455	-0.56069036	0.79135212	1.12224471	0.57081486	2.20637771		0.691841459
CAC	ebi-a-GCST90001823	IgD on IgD+ CD38- unswitched memory B cell || id:ebi-a-GCST90001823	exposure	Weighted median	5	0.18019113	0.1341994	0.17936541	-0.08283977	0.44322204	1.19744621	0.92049863	1.55771817		
CAC	ebi-a-GCST90001823	IgD on IgD+ CD38- unswitched memory B cell || id:ebi-a-GCST90001823	exposure	Weighted mode	5	0.1985652	0.1553699	0.27036986	-0.10595987	0.50309027	1.21965155	0.89946075	1.65382415		
CAC	ebi-a-GCST90001823	IgD on IgD+ CD38- unswitched memory B cell || id:ebi-a-GCST90001823	exposure	Contamination mixture method	5	0.26669725	0.1530612	0.02040189	-0.03330275	0.56669725	1.3056451	0.96724568	1.76243654		
CAC	ebi-a-GCST90001823	IgD on IgD+ CD38- unswitched memory B cell || id:ebi-a-GCST90001823	exposure	Robust adjusted profile score (RAPS)	5	0.27272091	0.1141364	0.01687454	0.049013583	0.49642823	1.31353359	1.05023462	1.64284292		
CAC	ebi-a-GCST90001880	HVEM on CD45RA- CD4+ T cell || id:ebi-a-GCST90001880	exposure	Inverse variance weighted	4	0.30075837	0.1417873	0.03390542	0.02285535	0.57866139	1.35088289	1.02311854	1.78364923	0.666335131	0.946478909
CAC	ebi-a-GCST90001880	HVEM on CD45RA- CD4+ T cell || id:ebi-a-GCST90001880	exposure	MR Egger	4	0.59004241	0.5950602	0.42590908	-0.5762756	1.75636041	1.80406492	0.56198754	5.79132097		0.942288201
CAC	ebi-a-GCST90001880	HVEM on CD45RA- CD4+ T cell || id:ebi-a-GCST90001880	exposure	Weighted median	4	0.27824568	0.1609579	0.08386524	-0.03723175	0.59372311	1.32081065	0.96345283	1.81071737		

CAC	ebi-a-GCST90001880	HVEM on CD45RA- CD4+ T cell || id:ebi-a-GCST90001880	exposure	Weighted mode	4	0.29272549	0.1783852	0.19933937	-0.05690943	0.64236041	1.34007487	0.94467962	1.90096264		
CAC	ebi-a-GCST90001880	HVEM on CD45RA- CD4+ T cell || id:ebi-a-GCST90001880	exposure	Contamination mixture method	4	0.29995526	0.1734694	0.04928479	-0.04004474	0.63995526	1.34979841	0.96074645	1.89639603		
CAC	ebi-a-GCST90001880	HVEM on CD45RA- CD4+ T cell || id:ebi-a-GCST90001880	exposure	Robust adjusted profile score (RAPS)	4	0.30142813	0.1510013	0.04591267	0.005465541	0.59739072	1.35178796	1.0054805	1.81737057		
CAC	ebi-a-GCST90001930	CD127 on CD28- CD8+ T cell || id:ebi-a-GCST90001930	exposure	Inverse variance weighted	4	0.26766407	0.0954108	0.00502566	0.080658836	0.4546693	1.30690803	1.08400101	1.57565223	0.754958943	0.763903847
CAC	ebi-a-GCST90001930	CD127 on CD28- CD8+ T cell || id:ebi-a-GCST90001930	exposure	MR Egger	4	0.13258381	0.3897708	0.76614139	-0.63136693	0.89653455	1.1417747	0.53186428	2.45109423		0.598435263
CAC	ebi-a-GCST90001930	CD127 on CD28- CD8+ T cell || id:ebi-a-GCST90001930	exposure	Weighted median	4	0.29920982	0.1144482	0.00893924	0.074891356	0.52352828	1.3487926	1.07776705	1.6879728		
CAC	ebi-a-GCST90001930	CD127 on CD28- CD8+ T cell || id:ebi-a-GCST90001930	exposure	Weighted mode	4	0.32250519	0.1364535	0.09908697	0.055056329	0.58995406	1.38058206	1.05660013	1.80390554		
CAC	ebi-a-GCST90001930	CD127 on CD28- CD8+ T cell || id:ebi-a-GCST90001930	exposure	Contamination mixture method	4	0.3303132	0.122449	0.0412074	0.090313199	0.5703132	1.39140385	1.09451703	1.76882096		

CAC	ebi-a-GCST90001930	CD127 on CD28- CD8+ T cell || id:ebi-a-GCST90001930	exposure	Robust adjusted profile score (RAPS)	4	0.26837294	0.1018177	0.00839349	0.068810191	0.46793568	1.30783479	1.07123286	1.59669471		
CAC	ebi-a-GCST90001935	CD25 on CD39+ CD4 regulatory T cell || id:ebi-a-GCST90001935	exposure	Inverse variance weighted	5	-0.1555857	0.0785161	0.04752672	-0.30947726	-0.0016941	0.85591376	0.73383046	0.99830737	0.612241466	0.708563589
CAC	ebi-a-GCST90001935	CD25 on CD39+ CD4 regulatory T cell || id:ebi-a-GCST90001935	exposure	MR Egger	5	-0.2928455	0.2557714	0.3352946	-0.79415745	0.20846641	0.7461374	0.45196188	1.23178755		0.608423595
CAC	ebi-a-GCST90001935	CD25 on CD39+ CD4 regulatory T cell || id:ebi-a-GCST90001935	exposure	Weighted median	5	-0.1288388	0.0967139	0.18280616	-0.31839809	0.06072043	0.87911564	0.72731319	1.0626018		
CAC	ebi-a-GCST90001935	CD25 on CD39+ CD4 regulatory T cell || id:ebi-a-GCST90001935	exposure	Weighted mode	5	-0.1225426	0.1070546	0.31618321	-0.33236962	0.08728441	0.88466821	0.71722217	1.09120699		
CAC	ebi-a-GCST90001935	CD25 on CD39+ CD4 regulatory T cell || id:ebi-a-GCST90001935	exposure	Contamination mixture method	5	-0.1531062	0.119898	0.07699712	-0.38810616	0.08189384	0.85803863	0.67834033	1.08534058		
CAC	ebi-a-GCST90001935	CD25 on CD39+ CD4 regulatory T cell || id:ebi-a-GCST90001935	exposure	Robust adjusted profile score (RAPS)	5	-0.1568891	0.0835859	0.06052076	-0.32071753	0.00693926	0.85479882	0.72562819	1.00696339		

CAC	ebi-a-GCST90001938	CD25 on CD39+ resting CD4 regulatory T cell || id:ebi-a-GCST90001938	exposure	Inverse variance weighted	5	-0.1805031	0.079917	0.02390621	-0.3371404	-0.0238659	0.83485007	0.71380861	0.97641668	0.818839119	0.883886251
CAC	ebi-a-GCST90001938	CD25 on CD39+ resting CD4 regulatory T cell || id:ebi-a-GCST90001938	exposure	MR Egger	5	-0.2424742	0.2605954	0.42076658	-0.75324128	0.26829289	0.78468399	0.47083796	1.3077301		0.776539988
CAC	ebi-a-GCST90001938	CD25 on CD39+ resting CD4 regulatory T cell || id:ebi-a-GCST90001938	exposure	Weighted median	5	-0.2089405	0.0981446	0.03326207	-0.40130393	-0.0165772	0.81144348	0.66944657	0.98355948		
CAC	ebi-a-GCST90001938	CD25 on CD39+ resting CD4 regulatory T cell || id:ebi-a-GCST90001938	exposure	Weighted mode	5	-0.1946812	0.1178951	0.17401994	-0.42575552	0.03639318	0.82309704	0.65327604	1.03706351		
CAC	ebi-a-GCST90001938	CD25 on CD39+ resting CD4 regulatory T cell || id:ebi-a-GCST90001938	exposure	Contamination mixture method	5	-0.2071953	0.0918367	0.02627218	-0.38719528	-0.0271953	0.8128609	0.67895849	0.97317118		
CAC	ebi-a-GCST90001938	CD25 on CD39+ resting CD4 regulatory T cell || id:ebi-a-GCST90001938	exposure	Robust adjusted profile score (RAPS)	5	-0.1811613	0.0850767	0.03322217	-0.34791162	-0.0144111	0.83430078	0.70616129	0.98569237		
CAC	ebi-a-GCST90001940	CD25 on CD39+ activated CD4 regulatory T cell || id:ebi-a-GCST90001940	exposure	Inverse variance weighted	5	-0.1614724	0.0790811	0.04116557	-0.31647135	-0.0064735	0.85089001	0.72871589	0.99354745	0.445094462	0.519157432
CAC	ebi-a-GCST90001940	CD25 on CD39+ activated CD4 regulatory T cell || id:ebi-a-GCST90001940	exposure	MR Egger	5	-0.376373	0.257512	0.2400125	-0.8810964	0.12835047	0.6863463	0.41432839	1.1369514		0.481300287

CAC	ebi-a-GCST90001940	CD25 on CD39+ activated CD4 regulatory T cell || id:ebi-a-GCST90001940	exposure	Weighted median	5	-0.1417594	0.0949884	0.13559772	-0.32793663	0.0444178	0.86783002	0.72040867	1.04541904		
CAC	ebi-a-GCST90001940	CD25 on CD39+ activated CD4 regulatory T cell || id:ebi-a-GCST90001940	exposure	Weighted mode	5	-0.1560166	0.1098641	0.22860648	-0.37135018	0.05931699	0.85554499	0.68980234	1.06111155		
CAC	ebi-a-GCST90001940	CD25 on CD39+ activated CD4 regulatory T cell || id:ebi-a-GCST90001940	exposure	Contamination mixture method	5	-0.1653866	0	0.13907016	-0.16538663	-0.1653866	0.84756594	0.84756594	0.84756594		
CAC	ebi-a-GCST90001940	CD25 on CD39+ activated CD4 regulatory T cell || id:ebi-a-GCST90001940	exposure	Robust adjusted profile score (RAPS)	5	-0.1568047	0.084091	0.06222302	-0.32162308	0.00801361	0.85487097	0.7249714	1.0080458		
CAC	ebi-a-GCST90002073	SSC-A on monocyte || id:ebi-a-GCST90002073	exposure	Inverse variance weighted	5	0.17210421	0.0870777	0.04810451	0.001431906	0.34277651	1.1878016	1.00143293	1.40885386	0.349691092	0.653704581
CAC	ebi-a-GCST90002073	SSC-A on monocyte || id:ebi-a-GCST90002073	exposure	MR Egger	5	0.46918255	0.282518	0.19535444	-0.08455269	1.02291779	1.59868681	0.91892324	2.78129818		0.746374239
CAC	ebi-a-GCST90002073	SSC-A on monocyte || id:ebi-a-GCST90002073	exposure	Weighted median	5	0.10562641	0.1065851	0.32168305	-0.10328043	0.31453326	1.11140659	0.90187402	1.36961991		
CAC	ebi-a-GCST90002073	SSC-A on monocyte || id:ebi-a-GCST90002073	exposure	Weighted mode	5	0.09354641	0.1247871	0.49514933	-0.15103626	0.33812909	1.09806157	0.85981652	1.40232152		

CAC	ebi-a-GCST90002073	SSC-A on monocyte || id:ebi-a-GCST90002073	exposure	Contamination mixture method	5	0.16796421	0.2015306	0.15939906	-0.22703579	0.56296421	1.18289427	0.79689226	1.75586955		
CAC	ebi-a-GCST90002073	SSC-A on monocyte || id:ebi-a-GCST90002073	exposure	Robust adjusted profile score (RAPS)	5	0.17388658	0.0920112	0.05877923	-0.00645542	0.35422858	1.1899206	0.99356538	1.42508089		

nsnp: number of single nucleotide polymorphisms, se: standard error, lo_ci: lower confidence interval, up_ci: upper confidence interval, or: odds ratio, or_lci95: odds ratio lower 95% confidence interval limit, or_uci95: odds ratio upper 95% confidence interval limit, p-FDR: p-value after False Discovery Rate correction

Table S8 Mendelian Randomization analysis on the causal effect between inflammation-related proteins and coronary artery calcification.

id.exposure	id.outcome	outcome	exposure	method	nsnp	b	se	pval	lo_ci	up_ci	or	or_lci95	or_uci95	pleiotropy	heterogeneity
GCST90274761	CAC	outcome	Axin-1 levels	Inverse variance weighted	13	-0.19851	0.093433	0.033619	-0.38164	-0.01538	0.819952	0.682742	0.984737	0.48611909	0.679268003
GCST90274761	CAC	outcome	Axin-1 levels	MR Egger	13	-0.03067	0.250917	0.904905	-0.52247	0.461122	0.969791	0.593053	1.585853		0.644430546
GCST90274761	CAC	outcome	Axin-1 levels	Weighted median	13	-0.22876	0.127763	0.073377	-0.47917	0.021658	0.795522	0.619295	1.021895		
GCST90274761	CAC	outcome	Axin-1 levels	Weighted mode	13	-0.30541	0.209993	0.171487	-0.717	0.106172	0.736818	0.488214	1.112013		
GCST90274761	CAC	outcome	Axin-1 levels	Contamination mixture method	13	-0.34424	0.168367	0.052329	-0.67424	-0.01424	0.70876	0.509544	0.985862		
GCST90274761	CAC	outcome	Axin-1 levels	Robust adjusted profile score (RAPS)	13	-0.2047	0.102012	0.044787	-0.40465	-0.00476	0.81489	0.667213	0.995252		
GCST90274778	CAC	outcome	Fractalkine levels	Inverse variance weighted (multiplicative random effects)	30	-0.16978	0.086051	0.048493	-0.33844	-0.00112	0.843851	0.712883	0.99888	0.69164132	0.009672785

GCST90274778	CAC	outcome	Fractalkine levels	MR Egger	30	-0.26225	0.246722	0.296884	-0.74583	0.22132	0.769315	0.474341	1.247723		0.007464572
GCST90274778	CAC	outcome	Fractalkine levels	Weighted median	30	-0.18354	0.097099	0.058732	-0.37385	0.006778	0.832321	0.688079	1.006801		
GCST90274778	CAC	outcome	Fractalkine levels	Weighted mode	30	-0.20346	0.205776	0.330976	-0.60678	0.199866	0.815906	0.545105	1.221239		
GCST90274778	CAC	outcome	Fractalkine levels	Contamination mixture method	30	-0.20099	0.089286	0.037687	-0.37599	-0.02599	0.81792	0.686609	0.974344		
GCST90274778	CAC	outcome	Fractalkine levels	Robust adjusted profile score (RAPS)	30	-0.13661	0.09413	0.146702	-0.3211	0.047886	0.872311	0.725348	1.049051		
GCST90274796	CAC	outcome	Interleukin-10 receptor subunit alpha levels	Inverse variance weighted	18	0.203221	0.078179	0.009338	0.049991	0.356452	1.225343	1.051261	1.428252	0.69198505	0.891458121
GCST90274796	CAC	outcome	Interleukin-10 receptor subunit alpha levels	MR Egger	18	0.106884	0.251276	0.676236	-0.38562	0.599386	1.112806	0.680031	1.821		0.860431576
GCST90274796	CAC	outcome	Interleukin-10 receptor subunit alpha levels	Weighted median	18	0.168629	0.107173	0.115619	-0.04143	0.378688	1.183681	0.959417	1.460367		
GCST90274796	CAC	outcome	Interleukin-10 receptor subunit alpha levels	Weighted mode	18	0.151811	0.1773	0.403774	-0.1957	0.499319	1.16394	0.822261	1.647599		

GCST90274796	CAC	outcome	Interleukin-10 receptor subunit alpha levels	Contamination mixture method	18	0.314212	0.160714	0.06792	-0.00079	0.629212	1.369179	0.999212	1.876131		
GCST90274796	CAC	outcome	Interleukin-10 receptor subunit alpha levels	Robust adjusted profile score (RAPS)	18	0.208542	0.085968	0.015274	0.040046	0.377039	1.231881	1.040858	1.457961		
GCST90274815	CAC	outcome	Interleukin-6 levels	Inverse variance weighted	10	-0.18814	0.091789	0.040394	-0.36804	-0.00823	0.8285	0.692086	0.991801	0.60927043	0.382519606
GCST90274815	CAC	outcome	Interleukin-6 levels	MR Egger	10	-0.28604	0.207453	0.205274	-0.69265	0.120569	0.751233	0.50025	1.128138		0.318675901
GCST90274815	CAC	outcome	Interleukin-6 levels	Weighted median	10	-0.25607	0.107498	0.017216	-0.46676	-0.04537	0.77409	0.627028	0.955643		
GCST90274815	CAC	outcome	Interleukin-6 levels	Weighted mode	10	-0.26178	0.110039	0.041298	-0.47746	-0.0461	0.769681	0.62036	0.954944		
GCST90274815	CAC	outcome	Interleukin-6 levels	Contamination mixture method	10	-0.28436	0.096939	0.017107	-0.47436	-0.09436	0.752496	0.622284	0.909956		
GCST90274815	CAC	outcome	Interleukin-6 levels	Robust adjusted profile score (RAPS)	10	-0.20201	0.094145	0.031898	-0.38653	-0.01748	0.81709	0.67941	0.98267		
GCST90274820	CAC	outcome	Leukemia inhibitory factor receptor levels	Inverse variance weighted	29	-0.15081	0.06024	0.012298	-0.26888	-0.03274	0.860011	0.764234	0.96779	0.11686689	0.140672946

GCST90274820	CAC	outcome	Leukemia inhibitory factor receptor levels	MR Egger	29	-0.29898	0.10861	0.010434	-0.51186	-0.08611	0.741572	0.59938	0.917497		0.201050713
GCST90274820	CAC	outcome	Leukemia inhibitory factor receptor levels	Weighted median	29	-0.2365	0.075075	0.001632	-0.38364	-0.08935	0.789388	0.681374	0.914526		
GCST90274820	CAC	outcome	Leukemia inhibitory factor receptor levels	Weighted mode	29	-0.29675	0.074056	0.000412	-0.4419	-0.1516	0.743231	0.642816	0.859333		
GCST90274820	CAC	outcome	Leukemia inhibitory factor receptor levels	Contamination mixture method	29	-0.24185	0.081633	0.018752	-0.40185	-0.08185	0.785177	0.669083	0.921413		
GCST90274820	CAC	outcome	Leukemia inhibitory factor receptor levels	Robust adjusted profile score (RAPS)	29	-0.1608	0.061826	0.009299	-0.28198	-0.03962	0.85146	0.754287	0.961152		
GCST90274840	CAC	outcome	TNF-beta levels	Inverse variance weighted	7	-0.11143	0.047217	0.018273	-0.20398	-0.01889	0.89455	0.815479	0.981289	0.47846477	0.296377689
GCST90274840	CAC	outcome	TNF-beta levels	MR Egger	7	-0.16008	0.080193	0.102444	-0.31725	-0.0029	0.852079	0.728146	0.997107		0.259756585
GCST90274840	CAC	outcome	TNF-beta levels	Weighted median	7	-0.13291	0.050908	0.009031	-0.23269	-0.03313	0.875541	0.792397	0.967408		
GCST90274840	CAC	outcome	TNF-beta levels	Weighted mode	7	-0.13348	0.04866	0.033593	-0.22886	-0.03811	0.875041	0.795441	0.962607		

GCST90274840	CAC	outcome	TNF-beta levels	Contamination mixture method	7	-0.12443	0.09949	0.142374	-0.31943	0.070575	0.883004	0.726566	1.073125		
GCST90274840	CAC	outcome	TNF-beta levels	Robust adjusted profile score (RAPS)	7	-0.11365	0.045556	0.012606	-0.20294	-0.02436	0.892571	0.816328	0.975935		

nsnp: number of single nucleotide polymorphisms, se: standard error, lo_ci: lower confidence interval, up_ci: upper confidence interval, or: odds ratio, or_lci95: odds ratio lower 95% confidence interval limit, or_uci95: odds ratio upper 95% confidence interval limit, p-FDR: p-value after False Discovery Rate correction

Table S9 Mendelian Randomization analysis on the causal effect between coronary artery calcification and inflammation-related proteins.

id.exposure	id.outcome	outcome	exposure	method	nsnp	b	se	pval	lo_ci	up_ci	or	or_lci95	or_uci95	pleiotropy	heterogeneity	p_fdr
CAC	GCST90274760	outcome	exposure	Inverse variance weighted	6	0.107777	0.048943	0.027659	0.011849	0.203706	1.1138	1.011919	1.225938	0.947321071	0.109444245	0.994596934
CAC	GCST90274760	outcome	exposure	MR Egger	6	0.119341	0.173315	0.528946	-0.22036	0.459038	1.126754	0.802233	1.582551		0.061616746	0.999686382
CAC	GCST90274760	outcome	exposure	Weighted median	6	0.137199	0.045719	0.002692	0.04759	0.226807	1.147056	1.048741	1.254588			0.244927789
CAC	GCST90274760	outcome	exposure	Weighted mode	6	0.132867	0.050827	0.047436	0.033245	0.232488	1.142098	1.033804	1.261736			0.964399448
CAC	GCST90274760	outcome	exposure	Contamination mixture method	6	0.138585	0.043367	0.001307	0.053585	0.223585	1.148647	1.055046	1.250551			0.118973425
CAC	GCST90274760	outcome	exposure	Robust adjusted profile score (RAPS)	6	0.125066	0.039012	0.001347	0.048602	0.201529	1.133223	1.049802	1.223272			0.12255662
CAC	GCST90274802	outcome	exposure	Inverse variance weighted	6	0.077297	0.038388	0.044052	0.002057	0.152537	1.080363	1.002059	1.164786	0.91549635	0.354115754	0.994596934
CAC	GCST90274802	outcome	exposure	MR Egger	6	0.091847	0.13573	0.535717	-0.17418	0.357878	1.096197	0.840142	1.430291		0.238198552	0.999686382
CAC	GCST90274802	outcome	exposure	Weighted median	6	0.057686	0.04623	0.212108	-0.03293	0.148298	1.059382	0.96761	1.159858			0.998382474
CAC	GCST90274802	outcome	exposure	Weighted mode	6	0.048087	0.055249	0.423908	-0.0602	0.156375	1.049262	0.941575	1.169265			0.964399448

CAC	GCST90274802	outcome	exposure	Contamination mixture method	6	0.098389	0.096939	0.215577	-0.09161	0.288389	1.103392	0.91246	1.334277			0.618394276
CAC	GCST90274802	outcome	exposure	Robust adjusted profile score (RAPS)	6	0.083343	0.0557	0.134577	-0.02583	0.192515	1.086915	0.974502	1.212295			0.97878598

nsnp: number of single nucleotide polymorphisms, se: standard error, lo_ci: lower confidence interval, up_ci: upper confidence interval, or: odds ratio, or_lci95: odds ratio lower 95% confidence interval limit, or_uci95: odds ratio upper 95% confidence interval limit, p-FDR: p-value after False Discovery Rate correction

Table S10 Mendelian Randomization analysis on the causal effect between inflammatory proteins and aortic stenosis.

exposure	id.exposure	id.outcome	method	nsnp	b	se	pval	lo_ci	up_ci	or	or_lci95	or_uci95	p_fdr	Egger intercept	P for pleiotropy	P for heterogeneity	P for Global Test
Caspase 8 levels	GCST90274763	AS	Inverse variance weighted	16	-0.1476	0.06537	0.02394	-0.2757	-0.0195	0.86276	0.75901	0.9807	0.62888			0.634243926	0.6583333
Caspase 8 levels	GCST90274763	AS	MR Egger	16	-0.1827	0.16366	0.28306	-0.5035	0.13807	0.83302	0.60443	1.14806		0.003248072	0.81847616	0.563717426	
Caspase 8 levels	GCST90274763	AS	Weighted median	16	-0.118	0.09177	0.19863	-0.2978	0.0619	0.88873	0.74243	1.06386					
Caspase 8 levels	GCST90274763	AS	Weighted mode	16	-0.0607	0.15514	0.70127	-0.3647	0.24341	0.94114	0.69438	1.27559					
C-C motif chemokine 25 levels	GCST90274768	AS	Inverse variance weighted	13	-0.0537	0.02512	0.0327	-0.1029	-0.0044	0.94776	0.90222	0.99559	0.62888			0.310525116	0.1796667
C-C motif chemokine 25 levels	GCST90274768	AS	MR Egger	13	-0.068	0.03997	0.11716	-0.1463	0.01038	0.9343	0.8639	1.01044		0.005053367	0.647028069	0.257440008	
C-C motif chemokine 25 levels	GCST90274768	AS	Weighted median	13	-0.0442	0.02889	0.12624	-0.1008	0.01245	0.95679	0.90412	1.01253					
C-C motif chemokine 25 levels	GCST90274768	AS	Weighted mode	13	-0.0448	0.02503	0.09836	-0.0939	0.0042	0.95614	0.91037	1.00421					
T-cell surface glycoprotein CD6 isoform levels	GCST90274774	AS	Inverse variance weighted	5	0.1888	0.09433	0.04533	0.00392	0.37368	1.2078	1.00393	1.45307	0.62888			0.215315779	0.1313333
T-cell surface glycoprotein CD6 isoform levels	GCST90274774	AS	MR Egger	5	0.62402	0.28979	0.12033	0.05604	1.192	1.86641	1.05764	3.29365		-0.039403288	0.215781943	0.363242684	
T-cell surface glycoprotein CD6 isoform levels	GCST90274774	AS	Weighted median	5	0.18294	0.10306	0.07587	-0.019	0.38494	1.20075	0.98113	1.46952					
T-cell surface glycoprotein CD6 isoform levels	GCST90274774	AS	Weighted mode	5	0.16859	0.13007	0.26466	-0.0864	0.42353	1.18363	0.91727	1.52734					
Interleukin-18 levels	GCST90274803	AS	Inverse variance weighted	25	0.11482	0.03871	0.00302	0.03895	0.19068	1.12167	1.03971	1.21008	0.62888			0.728375756	0.7703333
Interleukin-18 levels	GCST90274803	AS	MR Egger	25	0.06177	0.0803	0.44962	-0.0956	0.21916	1.06371	0.9088	1.24503		0.007198706	0.458512806	0.708908087	
Interleukin-18 levels	GCST90274803	AS	Weighted median	25	0.10195	0.05307	0.0547	-0.0021	0.20596	1.10733	0.99794	1.2287					
Interleukin-18 levels	GCST90274803	AS	Weighted mode	25	0.10691	0.0541	0.05975	0.00087	0.21294	1.11283	1.00087	1.23731					
Interleukin-6 levels	GCST90274815	AS	Inverse variance weighted	13	-0.1938	0.06151	0.00163	-0.3144	-0.0733	0.82381	0.73025	0.92936	0.62888			0.280835092	0.2653333
Interleukin-6 levels	GCST90274815	AS	MR Egger	13	-0.3929	0.11225	0.00497	-0.6129	-0.1729	0.6751	0.54178	0.84124		0.026423822	0.064962898	0.520028192	
Interleukin-6 levels	GCST90274815	AS	Weighted median	13	-0.3165	0.08166	0.00011	-0.4765	-0.1564	0.72872	0.62093	0.85521					
Interleukin-6 levels	GCST90274815	AS	Weighted mode	13	-0.2896	0.08091	0.00379	-0.4481	-0.131	0.7486	0.63882	0.87724					
Neurturin levels	GCST90274828	AS	Inverse variance weighted	18	-0.1324	0.06691	0.04789	-0.2635	-0.0012	0.87601	0.76834	0.99877	0.62888			0.155471102	0.1666667
Neurturin levels	GCST90274828	AS	MR Egger	18	-0.035	0.12737	0.78698	-0.2846	0.21464	0.9656	0.75228	1.23942		-0.0120192	0.381272417	0.15273877	
Neurturin levels	GCST90274828	AS	Weighted median	18	-0.1159	0.08458	0.17062	-0.2817	0.04989	0.89057	0.75452	1.05115					
Neurturin levels	GCST90274828	AS	Weighted mode	18	-0.0929	0.12747	0.47595	-0.3427	0.15692	0.91127	0.70982	1.1699					

Table S11 Secondary analysis results.

Mendelian Randomization analysis on the causal effect between circulating metabolites and aortic stenosis.

exposure	id.exposure	id.outcome	method	nsnp	b	se	pval	lo_ci	up_ci	or	or_lci95	or_uci95	p_fdr	P for pleiotropy	P for heterogeneity	P for Global Test
1-stearoyl-2-arachidonoyl-GPI (18:0/20:4) levels	ebi-met1400-GCST90199649	AS	Inverse variance weighted	4	0.16495	0.064342	0.010359	0.038839	0.291061	1.179334	1.039603	1.337846	0.318813	0.949624825	0.135976385	0.526667
1-stearoyl-2-arachidonoyl-GPI (18:0/20:4) levels	ebi-met1400-GCST90199649	AS	MR Egger	4	0.153738	0.175782	0.474023	-0.190794	0.49827	1.166185	0.826303	1.645872	0.988256			
1-stearoyl-2-arachidonoyl-GPI (18:0/20:4) levels	ebi-met1400-GCST90199649	AS	Weighted median	4	0.205647	0.055516	0.000212	0.096835	0.314459	1.228319	1.101678	1.369518	0.013357			
1-stearoyl-2-arachidonoyl-GPI (18:0/20:4) levels	ebi-met1400-GCST90199649	AS	Weighted mode	4	0.216164	0.056672	0.031696	0.105087	0.327242	1.241306	1.110807	1.387137	0.833876			
Campesterol levels	ebi-met1400-GCST90199725	AS	Inverse variance weighted	3	0.155648	0.035824	1.39E-05	0.085434	0.225863	1.168415	1.08919	1.253404	0.002574	0.527219451	0.563393444	0.774
Campesterol levels	ebi-met1400-GCST90199725	AS	MR Egger	3	0.0598	0.110391	0.683948	-0.156567	0.276166	1.061624	0.855074	1.318067	0.988256			
Campesterol levels	ebi-met1400-GCST90199725	AS	Weighted median	3	0.14634	0.03827	0.000131	0.071332	0.221349	1.15759	1.073938	1.247759	0.010343			
Campesterol levels	ebi-met1400-GCST90199725	AS	Weighted mode	3	0.141754	0.040367	0.072396	0.062635	0.220874	1.152294	1.064638	1.247166	0.833876			
Erythronate levels	ebi-met1400-GCST90199746	AS	Inverse variance weighted	4	-0.224291	0.095996	0.019467	-0.412443	-0.036139	0.799083	0.662031	0.964507	0.431395	0.558692347	0.173201385	0.7863333
Erythronate levels	ebi-met1400-GCST90199746	AS	MR Egger	4	-0.067951	0.248317	0.810026	-0.554652	0.41875	0.934306	0.574272	1.52006	0.988256			
Erythronate levels	ebi-met1400-GCST90199746	AS	Weighted median	4	-0.170527	0.091854	0.063383	-0.350561	0.009507	0.84322	0.704293	1.009553	0.570347			
Erythronate levels	ebi-met1400-GCST90199746	AS	Weighted mode	4	-0.133611	0.099975	0.273727	-0.329562	0.062341	0.874931	0.719239	1.064325	0.833876			
Androstenediol (3beta,17beta) monosulfate (2) levels	ebi-met1400-GCST90199871	AS	Inverse variance weighted	4	0.159238	0.068922	0.020865	0.024152	0.294325	1.172617	1.024446	1.34222	0.444581	0.73442862	0.704778548	0.573333
Androstenediol (3beta,17beta) monosulfate (2) levels	ebi-met1400-GCST90199871	AS	MR Egger	4	0.108347	0.147704	0.539563	-0.181153	0.397846	1.114434	0.834308	1.488615	0.988256			
Androstenediol (3beta,17beta) monosulfate (2) levels	ebi-met1400-GCST90199871	AS	Weighted median	4	0.12468	0.08077	0.122677	-0.03363	0.28299	1.132786	0.966929	1.327092	0.604613			
Androstenediol (3beta,17beta) monosulfate (2) levels	ebi-met1400-GCST90199871	AS	Weighted mode	4	0.117714	0.102218	0.332936	-0.082634	0.318063	1.124923	0.920688	1.374462	0.833876			
N-palmitoylglycine levels	ebi-met1400-GCST90199923	AS	Inverse variance weighted	3	-0.086181	0.040287	0.032423	-0.165143	-0.007218	0.917429	0.847772	0.992808	0.519004	0.735807996	0.870936323	0.736667
N-palmitoylglycine levels	ebi-met1400-GCST90199923	AS	MR Egger	3	-0.030151	0.133402	0.858493	-0.291619	0.231318	0.970299	0.747053	1.260259	0.988256			
N-palmitoylglycine levels	ebi-met1400-GCST90199923	AS	Weighted median	3	-0.085984	0.042461	0.042867	-0.169207	-0.00276	0.917609	0.844334	0.997244	0.570347			
N-palmitoylglycine levels	ebi-met1400-GCST90199923	AS	Weighted mode	3	-0.073668	0.049144	0.272617	-0.169991	0.022655	0.92898	0.843672	1.022913	0.833876			
1-stearoyl-2-arachidonoyl-GPE (18:0/20:4) levels	ebi-met1400-GCST90200045	AS	Inverse variance weighted	5	0.163962	0.046508	0.000423	0.072806	0.255117	1.178169	1.075522	1.290613	0.039034	0.789256849	0.133259559	0.753
1-stearoyl-2-arachidonoyl-GPE (18:0/20:4) levels	ebi-met1400-GCST90200045	AS	MR Egger	5	0.201055	0.137594	0.240101	-0.068629	0.470739	1.222692	0.933673	1.601177	0.988256			
1-stearoyl-2-arachidonoyl-GPE (18:0/20:4) levels	ebi-met1400-GCST90200045	AS	Weighted median	5	0.166592	0.041673	6.40E-05	0.084913	0.248271	1.181272	1.088623	1.281807	0.006718			
1-stearoyl-2-arachidonoyl-GPE (18:0/20:4) levels	ebi-met1400-GCST90200045	AS	Weighted mode	5	0.167954	0.04217	0.016363	0.0853	0.250607	1.182882	1.089044	1.284806	0.833876			
1-palmitoyl-2-arachidonoyl-GPE (16:0/20:4) levels	ebi-met1400-GCST90200054	AS	Inverse variance weighted	4	0.12066	0.044939	0.007253	0.03258	0.208739	1.128241	1.033116	1.232124	0.287028	0.75770876	0.246228804	0.6873333
1-palmitoyl-2-arachidonoyl-GPE (16:0/20:4) levels	ebi-met1400-GCST90200054	AS	MR Egger	4	0.151444	0.10222	0.276648	-0.048908	0.351796	1.163513	0.952269	1.421618	0.988256			
1-palmitoyl-2-arachidonoyl-GPE (16:0/20:4) levels	ebi-met1400-GCST90200054	AS	Weighted median	4	0.12142	0.040396	0.00265	0.042243	0.200596	1.129099	1.043148	1.222131	0.092733			
1-palmitoyl-2-arachidonoyl-GPE (16:0/20:4) levels	ebi-met1400-GCST90200054	AS	Weighted mode	4	0.123826	0.037748	0.046415	0.049839	0.197813	1.131819	1.051102	1.218735	0.833876			
1-linoleoyl-2-arachidonoyl-GPC (18:2/20:4n6) levels	ebi-met1400-GCST90200073	AS	Inverse variance weighted	5	0.169115	0.043381	9.68E-05	0.084088	0.254143	1.184257	1.087725	1.289356	0.010731	0.449639613	0.305710695	0.456667
1-linoleoyl-2-arachidonoyl-GPC (18:2/20:4n6) levels	ebi-met1400-GCST90200073	AS	MR Egger	5	0.236642	0.089832	0.078039	0.06057	0.412714	1.266987	1.062442	1.510912	0.988256			
1-linoleoyl-2-arachidonoyl-GPC (18:2/20:4n6) levels	ebi-met1400-GCST90200073	AS	Weighted median	5	0.187355	0.043537	1.68E-05	0.102023	0.272687	1.206055	1.107409	1.313489	0.002649			
1-linoleoyl-2-arachidonoyl-GPC (18:2/20:4n6) levels	ebi-met1400-GCST90200073	AS	Weighted mode	5	0.188091	0.045904	0.014883	0.09812	0.278063	1.206944	1.103095	1.320569	0.833876			
1-(1-enyl-stearoyl)-2-linoleoyl-GPE (p-18:0/18:2) levels	ebi-met1400-GCST90200076	AS	Inverse variance weighted	3	-0.244367	0.075858	0.001276	-0.393048	-0.095686	0.7832	0.674997	0.908749	0.088341	0.383075327	0.322207052	0.8456667

1-(1-enyl-stearoyl)-2-linoleoyl-GPE (p-18:0/18:2) levels	ebi-met1400-GCST90200076	AS	MR Egger	3	-0.577731	0.239757	0.250426	-1.047655	-0.107808	0.56117	0.350759	0.8978	0.988256			
1-(1-enyl-stearoyl)-2-linoleoyl-GPE (p-18:0/18:2) levels	ebi-met1400-GCST90200076	AS	Weighted median	3	-0.268118	0.083487	0.001321	-0.431753	-0.104483	0.764817	0.649369	0.90079	0.051996			
1-(1-enyl-stearoyl)-2-linoleoyl-GPE (p-18:0/18:2) levels	ebi-met1400-GCST90200076	AS	Weighted mode	3	-0.290634	0.087524	0.079963	-0.462181	-0.119088	0.747789	0.629908	0.88773	0.833876			
1-oleoyl-2-arachidonoyl-GPE (18:1/20:4) levels	ebi-met1400-GCST90200079	AS	Inverse variance weighted	3	0.112489	0.041798	0.007118	0.030565	0.194412	1.119059	1.031037	1.214596	0.287028	0.428074634	0.407582452	0.563333
1-oleoyl-2-arachidonoyl-GPE (18:1/20:4) levels	ebi-met1400-GCST90200079	AS	MR Egger	3	0.213253	0.090463	0.255411	0.035945	0.390561	1.237698	1.036599	1.47781	0.988256			
1-oleoyl-2-arachidonoyl-GPE (18:1/20:4) levels	ebi-met1400-GCST90200079	AS	Weighted median	3	0.122002	0.044318	0.005908	0.035138	0.208865	1.129756	1.035763	1.232279	0.186096			
1-oleoyl-2-arachidonoyl-GPE (18:1/20:4) levels	ebi-met1400-GCST90200079	AS	Weighted mode	3	0.129021	0.043792	0.09848	0.043189	0.214853	1.137714	1.044135	1.23968	0.833876			
Cis-3,4-methyleneheptanoylglycine levels	ebi-met1400-GCST90200274	AS	Inverse variance weighted	3	-0.080955	0.038662	0.03627	-0.156733	-0.005177	0.922236	0.854933	0.994837	0.528774	0.671302691	0.756133254	0.423333
Cis-3,4-methyleneheptanoylglycine levels	ebi-met1400-GCST90200274	AS	MR Egger	3	-0.039785	0.082185	0.712989	-0.200867	0.121298	0.960996	0.818021	1.128961	0.988256			
Cis-3,4-methyleneheptanoylglycine levels	ebi-met1400-GCST90200274	AS	Weighted median	3	-0.073972	0.040021	0.064551	-0.152412	0.004468	0.928698	0.858634	1.004478	0.570347			
Cis-3,4-methyleneheptanoylglycine levels	ebi-met1400-GCST90200274	AS	Weighted mode	3	-0.071582	0.043678	0.242916	-0.157191	0.014028	0.93092	0.854541	1.014127	0.833876			
1-palmitoyl-2-oleoyl-GPE (16:0/18:1) levels	ebi-met1400-GCST90200331	AS	Inverse variance weighted	5	0.16359	0.047743	0.000612	0.070013	0.257167	1.177731	1.072523	1.293261	0.048396	0.97392284	0.113401086	0.657
1-palmitoyl-2-oleoyl-GPE (16:0/18:1) levels	ebi-met1400-GCST90200331	AS	MR Egger	5	0.167539	0.124179	0.270076	-0.075852	0.41093	1.182391	0.926953	1.508219	0.988256			
1-palmitoyl-2-oleoyl-GPE (16:0/18:1) levels	ebi-met1400-GCST90200331	AS	Weighted median	5	0.184772	0.03962	3.11E-06	0.107117	0.262428	1.202945	1.113065	1.300083	0.000979			
1-palmitoyl-2-oleoyl-GPE (16:0/18:1) levels	ebi-met1400-GCST90200331	AS	Weighted mode	5	0.18208	0.040779	0.011117	0.102152	0.262007	1.19971	1.107552	1.299536	0.833876			
Trans-urocanate levels	ebi-met1400-GCST90200416	AS	Inverse variance weighted	3	-0.114082	0.043369	0.008526	-0.199084	-0.029079	0.892185	0.819481	0.97134	0.306273	0.510954229	0.437344632	0.7643333
Trans-urocanate levels	ebi-met1400-GCST90200416	AS	MR Egger	3	-0.022433	0.104302	0.865135	-0.226865	0.182	0.977817	0.797028	1.199615	0.988256			
Trans-urocanate levels	ebi-met1400-GCST90200416	AS	Weighted median	3	-0.098568	0.046025	0.032226	-0.188778	-0.008358	0.906134	0.82797	0.991677	0.570347			
Trans-urocanate levels	ebi-met1400-GCST90200416	AS	Weighted mode	3	-0.098312	0.043716	0.153471	-0.183996	-0.012628	0.906366	0.831939	0.987451	0.833876			
Arginine to citrulline ratio	ebi-met1400-GCST90200743	AS	Inverse variance weighted	4	0.159884	0.070385	0.023112	0.02193	0.297838	1.173375	1.022172	1.346943	0.448965	0.357404871	0.542694428	0.536667
Arginine to citrulline ratio	ebi-met1400-GCST90200743	AS	MR Egger	4	0.365707	0.187265	0.190069	-0.001333	0.732747	1.441533	0.998668	2.080789	0.988256			
Arginine to citrulline ratio	ebi-met1400-GCST90200743	AS	Weighted median	4	0.202662	0.085603	0.017911	0.03488	0.370444	1.224658	1.035495	1.448377	0.402994			
Arginine to citrulline ratio	ebi-met1400-GCST90200743	AS	Weighted mode	4	0.219783	0.107076	0.132434	0.009915	0.429652	1.245807	1.009964	1.536722	0.833876			
Uridine to pseudouridine ratio	ebi-met1400-GCST90200774	AS	Inverse variance weighted	3	-0.240564	0.092928	0.009633	-0.422703	-0.058426	0.786184	0.655273	0.943248	0.31393	0.354841142	0.276094847	0.7256667
Uridine to pseudouridine ratio	ebi-met1400-GCST90200774	AS	MR Egger	3	-1.965598	1.078347	0.31944	-4.079158	0.147961	0.140072	0.016922	1.159468	0.988256			
Uridine to pseudouridine ratio	ebi-met1400-GCST90200774	AS	Weighted median	3	-0.199005	0.107968	0.065303	-0.410622	0.012613	0.819546	0.663237	1.012693	0.570347			
Uridine to pseudouridine ratio	ebi-met1400-GCST90200774	AS	Weighted mode	3	-0.141048	0.155958	0.461241	-0.446726	0.164629	0.868447	0.639719	1.178956	0.833876			

Mendelian Randomization analysis on the causal effect between immune cells and aortic stenosis.

**exposure**	**id.exposure**	**id.outcome**	**method**	**nsnp**	**b**	**se**	**pval**	**lo_ci**	**up_ci**	**or**	**or_lci95**	**or_uci95**	**p_fdr**	**P for pleiotropy**	**P for heterogeneity**	**P for Global Test**
CD39+ CD8+ T cell %CD8+ T cell	ebi-a-GCST90001671	AS	Inverse variance weighted	7	0.059231	0.028098	0.035033	0.004158	0.114304	1.06102	1.004166	1.121092	0.995138	0.710820977	0.616578626	0.652
CD39+ CD8+ T cell %CD8+ T cell	ebi-a-GCST90001671	AS	MR Egger	7	0.040146	0.056152	0.506626	-0.069913	0.150204	1.040963	0.932475	1.162072	0.985672			
CD39+ CD8+ T cell %CD8+ T cell	ebi-a-GCST90001671	AS	Weighted median	7	0.060345	0.033615	0.072619	-0.005539	0.12623	1.062203	0.994476	1.134543	0.959795			
CD39+ CD8+ T cell %CD8+ T cell	ebi-a-GCST90001671	AS	Weighted mode	7	0.05601	0.042574	0.236338	-0.027434	0.139455	1.057609	0.972939	1.149647	0.999431			
CD25 on CD24+ CD27+ B cell	ebi-a-GCST90001777	AS	Inverse variance weighted	3	0.07502	0.035306	0.033597	0.005821	0.144219	1.077906	1.005838	1.155137	0.995138	0.651438879	0.466914617	0.4453333

CD25 on CD24+ CD27+ B cell	ebi-a-GCST90001777	AS	MR Egger	3	0.122807	0.086761	0.39156	-0.047244	0.292858	1.130667	0.953855	1.340253	0.985672			
CD25 on CD24+ CD27+ B cell	ebi-a-GCST90001777	AS	Weighted median	3	0.080256	0.037952	0.034458	0.00587	0.154642	1.083564	1.005887	1.16724	0.959795			
CD25 on CD24+ CD27+ B cell	ebi-a-GCST90001777	AS	Weighted mode	3	0.09291	0.0418	0.156294	0.010983	0.174838	1.097363	1.011043	1.191054	0.999431			
CD25 on IgD+ CD38- naive B cell	ebi-a-GCST90001781	AS	Inverse variance weighted	3	0.093396	0.047094	0.047348	0.001091	0.1857	1.097896	1.001092	1.204061	0.995138	0.757306915	0.900464928	0.673333
CD25 on IgD+ CD38- naive B cell	ebi-a-GCST90001781	AS	MR Egger	3	0.039218	0.143132	0.829744	-0.24132	0.319757	1.039998	0.78559	1.376793	0.985672			
CD25 on IgD+ CD38- naive B cell	ebi-a-GCST90001781	AS	Weighted median	3	0.094286	0.053708	0.07917	-0.010982	0.199555	1.098874	0.989078	1.220859	0.959795			
CD25 on IgD+ CD38- naive B cell	ebi-a-GCST90001781	AS	Weighted mode	3	0.083461	0.067206	0.340162	-0.048262	0.215185	1.087043	0.952884	1.240091	0.999431			
CD25 on memory B cell	ebi-a-GCST90001790	AS	Inverse variance weighted	3	0.075296	0.035472	0.033781	0.00577	0.144821	1.078203	1.005787	1.155833	0.995138	0.637017199	0.464744064	0.626667
CD25 on memory B cell	ebi-a-GCST90001790	AS	MR Egger	3	0.126366	0.087808	0.386601	-0.045736	0.298469	1.134698	0.955294	1.347794	0.985672			
CD25 on memory B cell	ebi-a-GCST90001790	AS	Weighted median	3	0.081214	0.038129	0.03317	0.006482	0.155947	1.084604	1.006503	1.168764	0.959795			
CD25 on memory B cell	ebi-a-GCST90001790	AS	Weighted mode	3	0.09385	0.038815	0.136808	0.017773	0.169926	1.098395	1.017932	1.185218	0.999431			
CD25 on unswitched memory B cell	ebi-a-GCST90001792	AS	Inverse variance weighted	3	0.080065	0.037032	0.030616	0.007481	0.152649	1.083358	1.00751	1.164916	0.995138	0.601720686	0.505620424	0.513333
CD25 on unswitched memory B cell	ebi-a-GCST90001792	AS	MR Egger	3	0.136492	0.086443	0.359408	-0.032935	0.30592	1.146246	0.967601	1.357874	0.985672			
CD25 on unswitched memory B cell	ebi-a-GCST90001792	AS	Weighted median	3	0.088727	0.040991	0.030424	0.008384	0.16907	1.092782	1.008419	1.184203	0.959795			
CD25 on unswitched memory B cell	ebi-a-GCST90001792	AS	Weighted mode	3	0.097523	0.042967	0.15127	0.013308	0.181739	1.102437	1.013397	1.199301	0.999431			
CD27 on memory B cell	ebi-a-GCST90001805	AS	Inverse variance weighted	7	-0.057799	0.025023	0.020898	-0.106844	-0.008754	0.94384	0.898665	0.991285	0.995138	0.997150319	0.687432664	0.7273333
CD27 on memory B cell	ebi-a-GCST90001805	AS	MR Egger	7	-0.058037	0.068237	0.433866	-0.191783	0.075708	0.943615	0.825486	1.078648	0.985672			
CD27 on memory B cell	ebi-a-GCST90001805	AS	Weighted median	7	-0.045981	0.030126	0.126937	-0.105028	0.013066	0.95506	0.900299	1.013152	0.959795			
CD27 on memory B cell	ebi-a-GCST90001805	AS	Weighted mode	7	-0.048128	0.030667	0.167611	-0.108235	0.011979	0.953012	0.897416	1.012051	0.999431			
CD38 on transitional B cell	ebi-a-GCST90001819	AS	Inverse variance weighted	5	0.073556	0.032868	0.025225	0.009135	0.137977	1.076329	1.009177	1.147949	0.995138	0.381615606	0.698214462	0.7033333
CD38 on transitional B cell	ebi-a-GCST90001819	AS	MR Egger	5	0.032083	0.052191	0.582221	-0.070212	0.134378	1.032603	0.932196	1.143825	0.985672			
CD38 on transitional B cell	ebi-a-GCST90001819	AS	Weighted median	5	0.069051	0.039311	0.078997	-0.007998	0.1461	1.071491	0.992034	1.157312	0.959795			
CD38 on transitional B cell	ebi-a-GCST90001819	AS	Weighted mode	5	0.127214	0.051501	0.068938	0.026271	0.228156	1.13566	1.02662	1.256281	0.999431			
IgD on IgD+ CD24- B cell	ebi-a-GCST90001821	AS	Inverse variance weighted	4	0.06403	0.027746	0.021016	0.009647	0.118413	1.066124	1.009694	1.125709	0.995138	0.54726819	0.651215783	0.644
IgD on IgD+ CD24- B cell	ebi-a-GCST90001821	AS	MR Egger	4	0.032547	0.051886	0.594546	-0.069151	0.134244	1.033082	0.933186	1.143672	0.985672			
IgD on IgD+ CD24- B cell	ebi-a-GCST90001821	AS	Weighted median	4	0.054685	0.031732	0.084827	-0.00751	0.116879	1.056207	0.992518	1.123983	0.959795			
IgD on IgD+ CD24- B cell	ebi-a-GCST90001821	AS	Weighted mode	4	0.04713	0.034318	0.263285	-0.020133	0.114393	1.048258	0.980069	1.121192	0.999431			
IgD on IgD+ CD38+ B cell	ebi-a-GCST90001824	AS	Inverse variance weighted	4	0.053853	0.027172	0.04749	0.000595	0.10711	1.055329	1.000595	1.113057	0.995138	0.41656009	0.720122519	0.7546667
IgD on IgD+ CD38+ B cell	ebi-a-GCST90001824	AS	MR Egger	4	0.008984	0.051854	0.878406	-0.092651	0.110618	1.009024	0.911512	1.116968	0.985672			
IgD on IgD+ CD38+ B cell	ebi-a-GCST90001824	AS	Weighted median	4	0.043191	0.029898	0.14856	-0.015408	0.101791	1.044138	0.98471	1.107152	0.959795			
IgD on IgD+ CD38+ B cell	ebi-a-GCST90001824	AS	Weighted mode	4	0.043323	0.032356	0.273008	-0.020096	0.106742	1.044275	0.980105	1.112647	0.999431			
IgD on IgD+ CD38dim B cell	ebi-a-GCST90001825	AS	Inverse variance weighted	4	0.063431	0.029227	0.029984	0.006147	0.120715	1.065486	1.006165	1.128303	0.995138	0.709730279	0.803841474	0.7896667
IgD on IgD+ CD38dim B cell	ebi-a-GCST90001825	AS	MR Egger	4	0.042134	0.057611	0.540647	-0.070784	0.155051	1.043034	0.931664	1.167717	0.985672			
IgD on IgD+ CD38dim B cell	ebi-a-GCST90001825	AS	Weighted median	4	0.057768	0.032021	0.071221	-0.004993	0.120529	1.059469	0.995019	1.128093	0.959795			
IgD on IgD+ CD38dim B cell	ebi-a-GCST90001825	AS	Weighted mode	4	0.050495	0.03562	0.251319	-0.01932	0.120311	1.051792	0.980865	1.127847	0.999431			
CD64 on CD14+ CD16- monocyte	ebi-a-GCST90001987	AS	Inverse variance weighted	5	-0.06088	0.024134	0.011648	-0.108182	-0.013578	0.940936	0.897465	0.986514	0.995138	0.329991954	0.722271408	0.6593333

CD64 on CD14+ CD16- monocyte	ebi-a-GCST90001987	AS	MR Egger	5	-0.158529	0.087574	0.167952	-0.330173	0.013116	0.853398	0.718799	1.013202	0.985672			
CD64 on CD14+ CD16- monocyte	ebi-a-GCST90001987	AS	Weighted median	5	-0.054878	0.027716	0.047705	-0.109202	-0.000554	0.946601	0.896549	0.999446	0.959795			
CD64 on CD14+ CD16- monocyte	ebi-a-GCST90001987	AS	Weighted mode	5	-0.049821	0.029307	0.164359	-0.107263	0.00762	0.9514	0.89829	1.007649	0.999431			
CD64 on monocyte	ebi-a-GCST90002006	AS	Inverse variance weighted	5	-0.064959	0.025467	0.01075	-0.114875	-0.015044	0.937106	0.891478	0.985068	0.995138	0.352463413	0.748501557	0.7136667
CD64 on monocyte	ebi-a-GCST90002006	AS	MR Egger	5	-0.15411	0.0851	0.167835	-0.320905	0.012686	0.857178	0.725492	1.012767	0.985672			
CD64 on monocyte	ebi-a-GCST90002006	AS	Weighted median	5	-0.058239	0.031801	0.067045	-0.120569	0.004091	0.943424	0.886416	1.004099	0.959795			
CD64 on monocyte	ebi-a-GCST90002006	AS	Weighted mode	5	-0.052109	0.032905	0.188458	-0.116603	0.012386	0.949226	0.889938	1.012463	0.999431			
HLA DR on monocyte	ebi-a-GCST90002010	AS	Inverse variance weighted	3	-0.057042	0.025373	0.024569	-0.106774	-0.00731	0.944554	0.898729	0.992716	0.995138	0.410995272	0.397716444	0.675
HLA DR on monocyte	ebi-a-GCST90002010	AS	MR Egger	3	-0.164076	0.084523	0.302835	-0.329741	0.00159	0.848678	0.71911	1.001591	0.985672			
HLA DR on monocyte	ebi-a-GCST90002010	AS	Weighted median	3	-0.058242	0.025383	0.02176	-0.107992	-0.008492	0.943422	0.897635	0.991544	0.959795			
HLA DR on monocyte	ebi-a-GCST90002010	AS	Weighted mode	3	-0.067359	0.027791	0.136273	-0.121829	-0.012889	0.93486	0.8853	0.987194	0.999431			
HLA DR on CD33+ HLA DR+ CD14dim	ebi-a-GCST90002109	AS	Inverse variance weighted	3	-0.065759	0.032828	0.045167	-0.130102	-0.001415	0.936357	0.878005	0.998586	0.995138	0.591367714	0.305906107	0.6576667
HLA DR on CD33+ HLA DR+ CD14dim	ebi-a-GCST90002109	AS	MR Egger	3	-0.211647	0.198688	0.479901	-0.601076	0.177781	0.80925	0.548222	1.194564	0.985672			
HLA DR on CD33+ HLA DR+ CD14dim	ebi-a-GCST90002109	AS	Weighted median	3	-0.06819	0.032062	0.033437	-0.131032	-0.005348	0.934083	0.87719	0.994666	0.959795			
HLA DR on CD33+ HLA DR+ CD14dim	ebi-a-GCST90002109	AS	Weighted mode	3	-0.07476	0.032442	0.147702	-0.138347	-0.011173	0.927966	0.870796	0.988889	0.999431			

Mendelian Randomization analysis on the causal effect between inflammatory proteins and aortic stenosis.

exposure	id.exposure	id.outcome	method	nsnp	b	se	pval	lo_ci	up_ci	or	or_lci95	or_uci95	p_fdr	P for pleiotropy	P for heterogeneity	P for Global Test
C-C motif chemokine 25 levels	GCST90274768	AS	Inverse variance weighted	5	-0.050865	0.024317	0.036461	-0.098527	-0.003204	0.950407	0.906172	0.996801	0.364607	0.754665135	0.690820164	0.7623333
C-C motif chemokine 25 levels	GCST90274768	AS	MR Egger	5	-0.038236	0.044182	0.450463	-0.124833	0.04836	0.962485	0.882644	1.049548	0.974641			
C-C motif chemokine 25 levels	GCST90274768	AS	Weighted median	5	-0.044158	0.027343	0.106322	-0.09775	0.009435	0.956803	0.906875	1.009479	0.526732			
C-C motif chemokine 25 levels	GCST90274768	AS	Weighted mode	5	-0.042612	0.031776	0.251005	-0.104893	0.019669	0.958283	0.900421	1.019864	0.865048			
C-X-C motif chemokine 11 levels	GCST90274781	AS	Inverse variance weighted	4	0.145971	0.061181	0.017037	0.026057	0.265886	1.157163	1.0264	1.304586	0.31909	0.734421273	0.384509785	0.5846667
C-X-C motif chemokine 11 levels	GCST90274781	AS	MR Egger	4	0.075162	0.195589	0.737777	-0.308193	0.458518	1.078059	0.734774	1.581728	0.974641			
C-X-C motif chemokine 11 levels	GCST90274781	AS	Weighted median	4	0.086445	0.072861	0.23545	-0.056363	0.229252	1.090291	0.945196	1.257659	0.62912			
C-X-C motif chemokine 11 levels	GCST90274781	AS	Weighted mode	4	0.078973	0.082676	0.409937	-0.083071	0.241017	1.082175	0.920286	1.272543	0.865048			
Leukemia inhibitory factor receptor levels	GCST90274820	AS	Inverse variance weighted	3	-0.138074	0.060385	0.022222	-0.256429	-0.019719	0.871034	0.77381	0.980475	0.31909	0.937431896	0.805721116	0.5486667
Leukemia inhibitory factor receptor levels	GCST90274820	AS	MR Egger	3	-0.147459	0.112719	0.415497	-0.368388	0.073471	0.862898	0.691848	1.076238	0.974641			
Leukemia inhibitory factor receptor levels	GCST90274820	AS	Weighted median	3	-0.129624	0.061705	0.035667	-0.250566	-0.008682	0.878426	0.77836	0.991355	0.377219			
Leukemia inhibitory factor receptor levels	GCST90274820	AS	Weighted mode	3	-0.129436	0.065853	0.188277	-0.258508	-0.000364	0.878591	0.772203	0.999636	0.865048			

Table S12 Mendelian Randomization analysis on the causal effect between the two of the identified gut flora, metabolites, immune cell characteristics and inflammatory proteins.

**Mendelian Randomization analysis on the causal effect between gut flora and metabolites.**																		
**exposure**	**id.exposure**	**id.outcome**	**outcome**	**method**	**nsnp**	**b**	**se**	**pval**	**lo_ci**	**up_ci**	**or**	**or_lci95**	**or_uci95**	**p_fdr**	**P for pleiotropy**	**P for heterogeneity**	**P for Global Test**
Gut microbiota abundance (k_Bacteria.p_Actinobacteria.c_Actinobacteria.o_Coriobacteriales.f_Coriobacteriaceae)	ebi-a-GCST90027663	ebi-met1400-GCST90199948	S-allylcysteine levels	Inverse variance weighted	8	0.152268	0.076602	0.0446	0.0036776	0.301168	1.164495	1.003668	1.35213	0.996334814	0.928559823	0.90071738	0.6413333
Gut microbiota abundance (k_Bacteria.p_Actinobacteria.c_Actinobacteria.o_Coriobacteriales.f_Coriobacteriaceae)	ebi-a-GCST90027663	ebi-met1400-GCST90199948	S-allylcysteine levels	MR Egger	8	0.1904	0.410558	0.659188	-0.614133	0.995513	1.209733	0.541	2.70507	0.997716378			
Gut microbiota abundance (k_Bacteria.p_Actinobacteria.c_Actinobacteria.o_Coriobacteriales.f_Coriobacteriaceae)	ebi-a-GCST90027663	ebi-met1400-GCST90199948	S-allylcysteine levels	Weighted median	8	0.105667	0.0978	0.279994	-0.0860224	0.297737	1.111146	0.91757	1.34631	0.997657354			
Gut microbiota abundance (k_Bacteria.p_Actinobacteria.c_Actinobacteria.o_Coriobacteriales.f_Coriobacteriaceae)	ebi-a-GCST90027663	ebi-met1400-GCST90199948	S-allylcysteine levels	Weighted mode	8	0.094472	0.141147	0.5246	-0.1825552	0.371199	1.099935	0.83314	1.45062	0.995193134			
Gut microbiota abundance (k_Bacteria.p_Firmicutes.c_Clostridia.o_Clostridiales.f_Lachnospiraceae)	ebi-a-GCST90027674	ebi-met1400-GCST90199731	10-heptadecenoate (17:1n7) levels	Inverse variance weighted	12	-0.125277	0.058851	0.032229	-0.2399558	-0.010558	0.882226	0.786666	0.98947	0.996334814	0.731561022	0.368588603	0.8123333
Gut microbiota abundance (k_Bacteria.p_Firmicutes.c_Clostridia.o_Clostridiales.f_Lachnospiraceae)	ebi-a-GCST90027674	ebi-met1400-GCST90199731	10-heptadecenoate (17:1n7) levels	MR Egger	12	-0.034425	0.265111	0.899778	-0.5538667	0.485537	0.966633	0.574472	1.62478	0.997716378			
Gut microbiota abundance (k_Bacteria.p_Firmicutes.c_Clostridia.o_Clostridiales.f_Lachnospiraceae)	ebi-a-GCST90027674	ebi-met1400-GCST90199731	10-heptadecenoate (17:1n7) levels	Weighted median	12	-0.173342	0.079947	0.029099	-0.3291722	-0.017666	0.840799	0.719552	0.98249	0.997657354			
Gut microbiota abundance (k_Bacteria.p_Firmicutes.c_Clostridia.o_Clostridiales.f_Lachnospiraceae)	ebi-a-GCST90027674	ebi-met1400-GCST90199731	10-heptadecenoate (17:1n7) levels	Weighted mode	12	-0.220448	0.145545	0.157776	-0.5055565	0.064461	0.802213	0.603316	1.06674	0.995193134			
Gut microbiota abundance (k_Bacteria.p_Firmicutes.c_Clostridia.o_Clostridiales.f_Lachnospiraceae)	ebi-a-GCST90027674	ebi-met1400-GCST90199744	Eicosenoate (20:1) levels	Inverse variance weighted	12	-0.124668	0.057785	0.031115	-0.2380669	-0.011129	0.882278	0.788815	0.98877	0.996334814	0.950014572	0.825418714	0.624
Gut microbiota abundance (k_Bacteria.p_Firmicutes.c_Clostridia.o_Clostridiales.f_Lachnospiraceae)	ebi-a-GCST90027674	ebi-met1400-GCST90199744	Eicosenoate (20:1) levels	MR Egger	12	-0.108895	0.251443	0.673998	-0.6017622	0.383386	0.896777	0.547885	1.46794	0.997716378			
Gut microbiota abundance (k_Bacteria.p_Firmicutes.c_Clostridia.o_Clostridiales.f_Lachnospiraceae)	ebi-a-GCST90027674	ebi-met1400-GCST90199744	Eicosenoate (20:1) levels	Weighted median	12	-0.095777	0.0787	0.223364	-0.2500016	0.058448	0.908667	0.778779	1.06022	0.997657354			
Gut microbiota abundance (k_Bacteria.p_Firmicutes.c_Clostridia.o_Clostridiales.f_Lachnospiraceae)	ebi-a-GCST90027674	ebi-met1400-GCST90199744	Eicosenoate (20:1) levels	Weighted mode	12	-0.090094	0.121111	0.468851	-0.3283313	0.146644	0.913088	0.720014	1.15771	0.995193134			
Gut microbiota abundance (k_Bacteria.p_Firmicutes.c_Clostridia.o_Clostridiales.f_Lachnospiraceae)	ebi-a-GCST90027674	ebi-met1400-GCST90199917	Pregnanediol-3-glucuronide levels	Inverse variance weighted	12	-0.118833	0.060117	0.049921	-0.2362261	-0.000041	0.8884	0.789557	0.99959	0.996334814	0.920508649	0.674605229	0.7236667
Gut microbiota abundance (k_Bacteria.p_Firmicutes.c_Clostridia.o_Clostridiales.f_Lachnospiraceae)	ebi-a-GCST90027674	ebi-met1400-GCST90199917	Pregnanediol-3-glucuronide levels	MR Egger	12	-0.092227	0.261168	0.7317	-0.6051558	0.420062	0.911186	0.545599	1.5229	0.997716378			
Gut microbiota abundance (k_Bacteria.p_Firmicutes.c_Clostridia.o_Clostridiales.f_Lachnospiraceae)	ebi-a-GCST90027674	ebi-met1400-GCST90199917	Pregnanediol-3-glucuronide levels	Weighted median	12	-0.1788	0.082188	0.029557	-0.3398872	-0.017773	0.836627	0.711186	0.98242	0.997657354			
Gut microbiota abundance (k_Bacteria.p_Firmicutes.c_Clostridia.o_Clostridiales.f_Lachnospiraceae)	ebi-a-GCST90027674	ebi-met1400-GCST90199917	Pregnanediol-3-glucuronide levels	Weighted mode	12	-0.230089	0.150118	0.152244	-0.5252251	0.063461	0.793822	0.591421	1.06552	0.995193134			

Gut microbiota abundance (k_Bacteria.p_Firmicutes.c_Clostridia.o_Clostridiales.f_Lachnospiraceae)	ebi-a-GCST90027674	ebi-met1400-GCST90200042	Oleate/vaccenate (18:1) levels	Inverse variance weighted	12	-0.154418	0.057708	0.006691	-0.266052	-0.0423	0.857122	0.7664	0.95858	0.603730981	0.795707811	0.637422774	0.6916667
Gut microbiota abundance (k_Bacteria.p_Firmicutes.c_Clostridia.o_Clostridiales.f_Lachnospiraceae)	ebi-a-GCST90027674	ebi-met1400-GCST90200042	Oleate/vaccenate (18:1) levels	MR Egger	12	-0.089995	0.2482	0.7246	-0.576419	0.396653	0.913398	0.56191	1.48665	0.997716378			
Gut microbiota abundance (k_Bacteria.p_Firmicutes.c_Clostridia.o_Clostridiales.f_Lachnospiraceae)	ebi-a-GCST90027674	ebi-met1400-GCST90200042	Oleate/vaccenate (18:1) levels	Weighted median	12	-0.1278	0.077	0.09698	-0.278716	0.02312	0.88003	0.75675	1.02339	0.997657354			
Gut microbiota abundance (k_Bacteria.p_Firmicutes.c_Clostridia.o_Clostridiales.f_Lachnospiraceae)	ebi-a-GCST90027674	ebi-met1400-GCST90200042	Oleate/vaccenate (18:1) levels	Weighted mode	12	-0.118831	0.1337	0.39514	-0.380372	0.14374	0.88842	0.68361	1.15459	0.995193134			
Gut microbiota abundance (k_Bacteria.p_Firmicutes.c_Clostridia.o_Clostridiales.f_Lachnospiraceae)	ebi-a-GCST90027674	ebi-met1400-GCST90200397	Palmitate (16:0) levels	Inverse variance weighted	12	-0.159955	0.05729	0.00535	-0.271838	-0.04726	0.85253	0.76198	0.95384	0.59972788	0.560952823	0.45262343	0.546667
Gut microbiota abundance (k_Bacteria.p_Firmicutes.c_Clostridia.o_Clostridiales.f_Lachnospiraceae)	ebi-a-GCST90027674	ebi-met1400-GCST90200397	Palmitate (16:0) levels	MR Egger	12	-0.010102	0.25531	0.96917	-0.510534	0.4903	0.98993	0.60018	1.6328	0.997716378			
Gut microbiota abundance (k_Bacteria.p_Firmicutes.c_Clostridia.o_Clostridiales.f_Lachnospiraceae)	ebi-a-GCST90027674	ebi-met1400-GCST90200397	Palmitate (16:0) levels	Weighted median	12	-0.149665	0.08142	0.06605	-0.309224	0.00992	0.86101	0.73402	1.00997	0.997657354			
Gut microbiota abundance (k_Bacteria.p_Firmicutes.c_Clostridia.o_Clostridiales.f_Lachnospiraceae)	ebi-a-GCST90027674	ebi-met1400-GCST90200397	Palmitate (16:0) levels	Weighted mode	12	-0.1325	0.13608	0.35115	-0.39922	0.13422	0.8759	0.67084	1.14365	0.995193134			
Gut microbiota abundance (k_Bacteria.p_Firmicutes.c_Clostridia.o_Clostridiales.f_Oscillospiraceae)	ebi-a-GCST90027675	ebi-met1400-GCST90199917	Pregnanediol-3-glucuronide levels	Inverse variance weighted	7	0.20099	0.08026	0.01227	0.0436889	0.3583	1.22262	1.04466	1.43089	0.719111622	0.287387175	0.879852631	0.823333
Gut microbiota abundance (k_Bacteria.p_Firmicutes.c_Clostridia.o_Clostridiales.f_Oscillospiraceae)	ebi-a-GCST90027675	ebi-met1400-GCST90199917	Pregnanediol-3-glucuronide levels	MR Egger	7	0.79441	0.50499	0.17651	-0.195383	1.78419	2.21313	0.82252	5.95478	0.997716378			
Gut microbiota abundance (k_Bacteria.p_Firmicutes.c_Clostridia.o_Clostridiales.f_Oscillospiraceae)	ebi-a-GCST90027675	ebi-met1400-GCST90199917	Pregnanediol-3-glucuronide levels	Weighted median	7	0.19791	0.10186	0.05202	-0.001736	0.39756	1.21886	0.99827	1.48819	0.997657354			
Gut microbiota abundance (k_Bacteria.p_Firmicutes.c_Clostridia.o_Clostridiales.f_Oscillospiraceae)	ebi-a-GCST90027675	ebi-met1400-GCST90199917	Pregnanediol-3-glucuronide levels	Weighted mode	7	0.194424	0.14481	0.22836	-0.089596	0.47807	1.21438	0.9143	1.61296	0.995193134			

Mendelian Randomization analysis on the causal effect between metabolites and gut flora.

**exposure**	**id.exposure**	**id.outcome**	**outcome**	**method**	**nsnp**	**b**	**se**	**pval**	**lo_ci**	**up_ci**	**or**	**or_lci95**	**or_uci95**	**p_fdr**	**P for pleiotropy**	**P for heterogeneity**	**P for Global Test**
Octanoylcarnitine (c8) levels	ebi-met1400-GCST90199720	ebi-a-GCST90027663	Gut microbiota abundance (k_Bacteria.p_Actinobacteria.c_Actinobacteria.o_Coriobacteriales.f_Coriobacteriaceae)	Inverse variance weighted	13	0.092669	0.037779	0.014417	0.0186247	0.166675	1.097122	1.0188	1.18146	0.970325165	0.474462011	0.30116682	0.6723333
Octanoylcarnitine (c8) levels	ebi-met1400-GCST90199720	ebi-a-GCST90027663	Gut microbiota abundance (k_Bacteria.p_Actinobacteria.c_Actinobacteria.o_Coriobacteriales.f_Coriobacteriaceae)	MR Egger	13	0.050559	0.068666	0.476663	-0.083982	0.185157	1.051889	0.91945	1.20342	0.998600886			
Octanoylcarnitine (c8) levels	ebi-met1400-GCST90199720	ebi-a-GCST90027663	Gut microbiota abundance (k_Bacteria.p_Actinobacteria.c_Actinobacteria.o_Coriobacteriales.f_Coriobacteriaceae)	Weighted median	13	0.079948	0.041977	0.058277	-0.002785	0.161174	1.082722	0.99722	1.17556	0.994136208			
Octanoylcarnitine (c8) levels	ebi-met1400-GCST90199720	ebi-a-GCST90027663	Gut microbiota abundance (k_Bacteria.p_Actinobacteria.c_Actinobacteria.o_Coriobacteriales.f_Coriobacteriaceae)	Weighted mode	13	0.078847	0.042774	0.091221	-0.005291	0.162224	1.081664	0.99472	1.17614	0.987538236			
Sphingomyelin (d18:1/18:1, d18:2/18:0) levels	ebi-met1400-	ebi-a-GCST90027778	Gut microbiota abundance (k_Bacteria.p_Bacteroidetes.c_Bacteroidia.o_Bacteroidales.f_Rikenellaceae.g_Alistipes.s_Alistipes_putredinis)	Inverse variance	16	0.11	0.05	0.04	0.004	0.21	1.11	1.00	1.24218	0.97032	0.197545089	0.270750787	0.7926667

	GCST90199878			weighted		076	414	077	6471	687	712	466		5165			
Sphingomyelin (d18:1/18:1, d18:2/18:0) levels	ebi-met1400-GCST90199878	ebi-a-GCST90027778	Gut microbiota abundance (k_Bacteria.p_Bacteroidetes.c_Bacteroidia.o_Bacteroidales.f_Rikenellaceae.g_Alistipes.s_Alistipes_putredinis)	MR Egger	16	0.30907	0.15577	0.0672	0.003757	0.61438	1.36216	1.00376	1.84851	0.998600886			
Sphingomyelin (d18:1/18:1, d18:2/18:0) levels	ebi-met1400-GCST90199878	ebi-a-GCST90027778	Gut microbiota abundance (k_Bacteria.p_Bacteroidetes.c_Bacteroidia.o_Bacteroidales.f_Rikenellaceae.g_Alistipes.s_Alistipes_putredinis)	Weighted median	16	0.13125	0.06964	0.05946	-0.0052441	0.26774	1.14025	0.99477	1.30701	0.994136208			
Sphingomyelin (d18:1/18:1, d18:2/18:0) levels	ebi-met1400-GCST90199878	ebi-a-GCST90027778	Gut microbiota abundance (k_Bacteria.p_Bacteroidetes.c_Bacteroidia.o_Bacteroidales.f_Rikenellaceae.g_Alistipes.s_Alistipes_putredinis)	Weighted mode	16	0.15525	0.07918	0.06875	5.78E-05	0.31045	1.16795	1.00006	1.36404	0.987538236			
N-acetyltaurine levels	ebi-met1400-GCST90199967	ebi-a-GCST90027674	Gut microbiota abundance (k_Bacteria.p_Firmicutes.c_Clostridia.o_Clostridiales.f_Lachnospiraceae)	Inverse variance weighted	10	-0.14037	0.0685	0.04046	-0.274631	-0.0061	0.86904	0.75985	0.99392	0.970325165	0.383472214	0.293497044	0.5126667
N-acetyltaurine levels	ebi-met1400-GCST90199967	ebi-a-GCST90027674	Gut microbiota abundance (k_Bacteria.p_Firmicutes.c_Clostridia.o_Clostridiales.f_Lachnospiraceae)	MR Egger	10	-0.25697	0.1441	0.11239	-0.539416	0.02547	0.77339	0.58309	1.0258	0.998600886			
N-acetyltaurine levels	ebi-met1400-GCST90199967	ebi-a-GCST90027674	Gut microbiota abundance (k_Bacteria.p_Firmicutes.c_Clostridia.o_Clostridiales.f_Lachnospiraceae)	Weighted median	10	-0.19123	0.08337	0.0218	-0.354635	-0.02783	0.82594	0.70143	0.97255	0.994136208			
N-acetyltaurine levels	ebi-met1400-GCST90199967	ebi-a-GCST90027674	Gut microbiota abundance (k_Bacteria.p_Firmicutes.c_Clostridia.o_Clostridiales.f_Lachnospiraceae)	Weighted mode	10	-0.19257	0.09062	0.06253	-0.370198	-0.01495	0.82483	0.6906	0.98516	0.987538236			
N,N-dimethylalanine levels	ebi-met1400-GCST90200192	ebi-a-GCST90027675	Gut microbiota abundance (k_Bacteria.p_Firmicutes.c_Clostridia.o_Clostridiales.f_Oscillospiraceae)	Inverse variance weighted	11	-0.20317	0.06125	0.00091	-0.323219	-0.08313	0.81614	0.72381	0.92023	0.75548597	0.683122274	0.314881317	0.714
N,N-dimethylalanine levels	ebi-met1400-GCST90200192	ebi-a-GCST90027675	Gut microbiota abundance (k_Bacteria.p_Firmicutes.c_Clostridia.o_Clostridiales.f_Oscillospiraceae)	MR Egger	11	-0.29002	0.21563	0.21154	-0.712657	0.13261	0.74825	0.49034	1.14181	0.998600886			
N,N-dimethylalanine levels	ebi-met1400-GCST90200192	ebi-a-GCST90027675	Gut microbiota abundance (k_Bacteria.p_Firmicutes.c_Clostridia.o_Clostridiales.f_Oscillospiraceae)	Weighted median	11	-0.27037	0.07804	0.00053	-0.423338	-0.11741	0.76309	0.65486	0.88922	0.439984006			
N,N-dimethylalanine levels	ebi-met1400-GCST90200192	ebi-a-GCST90027675	Gut microbiota abundance (k_Bacteria.p_Firmicutes.c_Clostridia.o_Clostridiales.f_Oscillospiraceae)	Weighted mode	11	-0.30637	0.09816	0.01085	-0.49876	-0.11399	0.73611	0.60728	0.89227	0.987538236			
Deoxycholic acid 12-sulfate levels	ebi-met1400-GCST90200250	ebi-a-GCST90027778	Gut microbiota abundance (k_Bacteria.p_Bacteroidetes.c_Bacteroidia.o_Bacteroidales.f_Rikenellaceae.g_Alistipes.s_Alistipes_putredinis)	Inverse variance weighted	15	0.08446	0.03683	0.02183	0.0122748	0.15665	1.08813	1.01235	1.16958	0.970325165	0.355793569	0.9535008	0.4243333
Deoxycholic acid 12-sulfate levels	ebi-met1400-GCST90200250	ebi-a-GCST90027778	Gut microbiota abundance (k_Bacteria.p_Bacteroidetes.c_Bacteroidia.o_Bacteroidales.f_Rikenellaceae.g_Alistipes.s_Alistipes_putredinis)	MR Egger	15	0.14024	0.06892	0.06279	0.0051561	0.27532	1.15055	1.00517	1.31695	0.998600886			
Deoxycholic acid 12-sulfate levels	ebi-met1400-GCST90200250	ebi-a-GCST90027778	Gut microbiota abundance (k_Bacteria.p_Bacteroidetes.c_Bacteroidia.o_Bacteroidales.f_Rikenellaceae.g_Alistipes.s_Alistipes_putredinis)	Weighted median	15	0.09926	0.04878	0.04188	0.0036463	0.19487	1.10435	1.00365	1.21516	0.994136208			
Deoxycholic acid 12-sulfate levels	ebi-met1400-GCST90200250	ebi-a-GCST90027778	Gut microbiota abundance (k_Bacteria.p_Bacteroidetes.c_Bacteroidia.o_Bacteroidales.f_Rikenellaceae.g_Alistipes.s_Alistipes_putredinis)	Weighted mode	15	0.10615	0.05379	0.0685	0.0007299	0.21158	1.11199	1.00073	1.23563	0.987538236			
Trans-4-hydroxyproline levels	ebi-met1400-GCST90200292	ebi-a-GCST90027778	Gut microbiota abundance (k_Bacteria.p_Bacteroidetes.c_Bacteroidia.o_Bacteroidales.f_Rikenellaceae.g_Alistipes.s_Alistipes_putredinis)	Inverse variance weighted	12	0.19531	0.06798	0.00407	0.0620662	0.32856	1.21569	1.06403	1.38897	0.970325165	0.248781135	0.438869039	0.3426667
Trans-4-hydroxyproline levels	ebi-met1400-GCST90200292	ebi-a-GCST90027778	Gut microbiota abundance (k_Bacteria.p_Bacteroidetes.c_Bacteroidia.o_Bacteroidales.f_Rikenellaceae.g_Alistipes.s_Alistipes_putredinis)	MR Egger	12	0.6015	0.33854	0.10599	-0.062047	1.26504	1.82485	0.93984	3.54325	0.998600886			
Trans-4-hydroxyproline levels	ebi-met1400-GCST90200292	ebi-a-GCST90027778	Gut microbiota abundance (k_Bacteria.p_Bacteroidetes.c_Bacteroidia.o_Bacteroidales.f_Rikenellaceae.g_Alistipes.s_Alistipes_putredinis)	Weighted median	12	0.21719	0.094	0.02087	0.0329403	0.40143	1.24258	1.03349	1.49396	0.994136208			
Trans-4-hydroxyproline levels	ebi-met1400-GCST90200292	ebi-a-GCST90027778	Gut microbiota abundance (k_Bacteria.p_Bacteroidetes.c_Bacteroidia.o_Bacteroidales.f_Rikenellaceae.g_Alistipes.s_Alistipes_putredinis)	Weighted mode	12	0.26949	0.17595	0.15385	-0.07537	0.61434	1.30929	0.9274	1.84844	0.987538236			

Mendelian Randomization analysis on the causal effect between gut flora and immune cell characteristics.

exposure	id.exposure	id.outcome	outcome	method	nsnp	b	se	pval	lo_ci	up_ci	or	or_lci95	or_uci95	p_fdr	P for pleiotropy	P for heterogeneity	P for Global Test
Gut bacterial pathway abundance (PWY.7323..superpathway.of.GDP.mannose.derived.O.antigen.building.blocks.biosynthesis)	ebi-a-GCST90027621	ebi-a-GCST90001824	IgD on IgD+ CD38+ B cell || id:ebi-a-GCST90001824	Inverse variance weighted	10	-0.19072	0.094844	0.04432	-0.376603	-0.004484	0.826336	0.686619	0.99517	0.897410303	0.968929525	0.499650817	0.2156667
Gut bacterial pathway abundance (PWY.7323..superpathway.of.GDP.mannose.derived.O.antigen.building.blocks.biosynthesis)	ebi-a-GCST90027621	ebi-a-GCST90001824	IgD on IgD+ CD38+ B cell || id:ebi-a-GCST90001824	MR Egger	10	-0.176667	0.362822	0.639376	-0.887809	0.534446	0.838805	0.411516	1.70653	0.998409511			
Gut bacterial pathway abundance (PWY.7323..superpathway.of.GDP.mannose.derived.O.antigen.building.blocks.biosynthesis)	ebi-a-GCST90027621	ebi-a-GCST90001824	IgD on IgD+ CD38+ B cell || id:ebi-a-GCST90001824	Weighted median	10	-0.107338	0.131519	0.414449	-0.365299	0.150544	0.898188	0.693939	1.16246	0.988438743			
Gut bacterial pathway abundance (PWY.7323..superpathway.of.GDP.mannose.derived.O.antigen.building.blocks.biosynthesis)	ebi-a-GCST90027621	ebi-a-GCST90001824	IgD on IgD+ CD38+ B cell || id:ebi-a-GCST90001824	Weighted mode	10	-0.063335	0.207923	0.767574	-0.470902	0.344419	0.938611	0.624444	1.41085	0.980098828			
Gut microbiota abundance (k_Bacteria.p_Actinobacteria.c_Actinobacteria.o_Coriobacteriales)	ebi-a-GCST90027737	ebi-a-GCST90001744	CD20 on CD20- CD38- B cell || id:ebi-a-GCST90001744	Inverse variance weighted	9	-0.244424	0.115066	0.033778	-0.469755	-0.018872	0.7833	0.625116	0.98146	0.766328158	0.194398968	0.734933682	0.6473333
Gut microbiota abundance (k_Bacteria.p_Actinobacteria.c_Actinobacteria.o_Coriobacteriales)	ebi-a-GCST90027737	ebi-a-GCST90001744	CD20 on CD20- CD38- B cell || id:ebi-a-GCST90001744	MR Egger	9	-1.215523	0.686633	0.119993	-2.560442	0.129998	0.296664	0.077277	1.1388	0.998409511			
Gut microbiota abundance (k_Bacteria.p_Actinobacteria.c_Actinobacteria.o_Coriobacteriales)	ebi-a-GCST90027737	ebi-a-GCST90001744	CD20 on CD20- CD38- B cell || id:ebi-a-GCST90001744	Weighted median	9	-0.293316	0.156003	0.060026	-0.598979	0.012265	0.7459	0.549937	1.01273	0.988438743			
Gut microbiota abundance (k_Bacteria.p_Actinobacteria.c_Actinobacteria.o_Coriobacteriales)	ebi-a-GCST90027737	ebi-a-GCST90001744	CD20 on CD20- CD38- B cell || id:ebi-a-GCST90001744	Weighted mode	9	-0.326605	0.231833	0.197223	-0.780427	0.128333	0.721777	0.458281	1.13693	0.980098828			
Gut microbiota abundance (k_Bacteria.p_Bacteroidetes.c_Bacteroidia.o_Bacteroidales.f_Rikenellaceae.g_Alistipes.s_Alistipes_putredinis)	ebi-a-GCST90027778	ebi-a-GCST90001552	Naive CD8+ T cell %CD8+ T cell || id:ebi-a-GCST90001552	Inverse variance weighted	6	-0.260226	0.111919	0.020013	-0.479755	-0.040776	0.770885	0.618994	0.96006	0.735284816	0.937478429	0.324986523	0.7123333
Gut microbiota abundance (k_Bacteria.p_Bacteroidetes.c_Bacteroidia.o_Bacteroidales.f_Rikenellaceae.g_Alistipes.s_Alistipes_putredinis)	ebi-a-GCST90027778	ebi-a-GCST90001552	Naive CD8+ T cell %CD8+ T cell || id:ebi-a-GCST90001552	MR Egger	6	-0.213	0.579975	0.731994	-1.349308	0.923331	0.808816	0.259442	2.51761	0.998409511			
Gut microbiota abundance (k_Bacteria.p_Bacteroidetes.c_Bacteroidia.o_Bacteroidales.f_Rikenellaceae.g_Alistipes.s_Alistipes_putredinis)	ebi-a-GCST90027778	ebi-a-GCST90001552	Naive CD8+ T cell %CD8+ T cell || id:ebi-a-GCST90001552	Weighted median	6	-0.207714	0.144007	0.150449	-0.489515	0.075223	0.8129	0.612292	1.07813	0.988438743			
Gut microbiota abundance (k_Bacteria.p_Bacteroidetes.c_Bacteroidia.o_Bacteroidales.f_Rikenellaceae.g_Alistipes.s_Alistipes_putredinis)	ebi-a-GCST90027778	ebi-a-GCST90001552	Naive CD8+ T cell %CD8+ T cell || id:ebi-a-GCST90001552	Weighted mode	6	-0.147716	0.200094	0.496682	-0.541013	0.246669	0.863315	0.582216	1.27978	0.980098828			
Gut microbiota abundance (k_Bacteria.p_Bacteroidetes.c_Bacteroidia.o_Bacteroidales.f_Rikenellaceae.g_Alistipes.s_Alistipes_putredinis)	ebi-a-GCST90027778	ebi-a-GCST90001690	CD28+ CD45RA+ CD8+ T cell Absolute Count || id:ebi-a-GCST90001690	Inverse variance weighted	6	-0.278828	0.098861	0.004777	-0.471556	-0.085	0.757709	0.624003	0.91851	0.644284437	0.63979141	0.836150023	0.8466667
Gut microbiota abundance (k_Bacteria.p_Bacteroidetes.c_Bacteroidia.o_Bacteroidales.f_Rikenellaceae.g_Alistipes.s_Alistipes_putredinis)	ebi-a-GCST90027778	ebi-a-GCST90001690	CD28+ CD45RA+ CD8+ T cell Absolute Count || id:ebi-a-GCST90001690	MR Egger	6	-0.052111	0.458815	0.914492	-0.950083	0.845855	0.949222	0.386671	2.32997	0.998409511			
Gut microbiota abundance (k_Bacteria.p_Bacteroidetes.c_Bacteroidia.o_Bacteroidales.f_Rikenellaceae.g_Alistipes.s_Alistipes_putredinis)	ebi-a-GCST90027778	ebi-a-GCST90001690	CD28+ CD45RA+ CD8+ T cell Absolute Count || id:ebi-a-GCST90001690	Weighted median	6	-0.233384	0.137761	0.089928	-0.503564	0.035589	0.791149	0.604437	1.03654	0.988438743			
Gut microbiota abundance (k_Bacteria.p_Bacteroidetes.c_Bacteroidia.o_Bacteroidales.f_Rikenellaceae.g_Alistipes.s_Alistipes_putredinis)	ebi-a-GCST90027778	ebi-a-GCST90001690	CD28+ CD45RA+ CD8+ T cell Absolute Count || id:ebi-a-GCST90001690	Weighted mode	6	-0.183317	0.178815	0.351011	-0.532344	0.166601	0.832263	0.587233	1.18058	0.980098828			

Mendelian Randomization analysis on the causal effect between immune cell characteristics and gut flora.

exposure	id.exposure	id.outcome	outcome	method	nsnp	b	se	pval	lo_ci	up_ci	or	or_lci95	or_uci95	p_fdr	P for pleiotropy	P for heterogeneity	P for Global Test
CD27 on memory B cell	ebi-a-GCST90001805	ebi-a-GCST90027778	Gut microbiota abundance (k_Bacteria.p_Bacteroidetes.c_Bacteroidia.o_Bacteroidales.f_Rikenellaceae.g_Alistipes.s_Alistipes_putredinis)	Inverse variance weighted	11	-0.05792	0.02807	0.03904	-0.11293	-0.00291	0.94373	0.89321	0.99709	0.99011796	0.416837179	0.913074032	0.7126667
CD27 on memory B cell	ebi-a-GCST90001805	ebi-a-GCST90027778	Gut microbiota abundance (k_Bacteria.p_Bacteroidetes.c_Bacteroidia.o_Bacteroidales.f_Rikenellaceae.g_Alistipes.s_Alistipes_putredinis)	MR Egger	11	-0.01693	0.05575	0.76831	-0.126198	0.09234	0.98322	0.88144	1.09674	0.992584069			
CD27 on memory B cell	ebi-a-GCST90001805	ebi-a-GCST90027778	Gut microbiota abundance (k_Bacteria.p_Bacteroidetes.c_Bacteroidia.o_Bacteroidales.f_Rikenellaceae.g_Alistipes.s_Alistipes_putredinis)	Weighted median	11	-0.04813	0.0361	0.18244	-0.118885	0.02262	0.95301	0.88791	1.02288	0.987483487			
CD27 on memory B cell	ebi-a-GCST90001805	ebi-a-GCST90027778	Gut microbiota abundance (k_Bacteria.p_Bacteroidetes.c_Bacteroidia.o_Bacteroidales.f_Rikenellaceae.g_Alistipes.s_Alistipes_putredinis)	Weighted mode	11	-0.03265	0.03625	0.38899	-0.103694	0.0384	0.96788	0.9015	1.03915	0.993569334			
CD38 on IgD+ CD38+ B cell	ebi-a-GCST90001812	ebi-a-GCST90027850	Gut microbiota abundance (k_Bacteria.p_Firmicutes.c_Clostridia.o_Clostridiales.f_Lachnospiraceae.g_Lachnospiraceae_noname.s_Lachnospiraceae_bacterium_3_1_46FAA)	Inverse variance weighted	5	-0.174	0.08379	0.03784	-0.338227	-0.00977	0.8403	0.71303	0.99028	0.99011796	0.744072087	0.992731344	0.534
CD38 on IgD+ CD38+ B cell	ebi-a-GCST90001812	ebi-a-GCST90027850	Gut microbiota abundance (k_Bacteria.p_Firmicutes.c_Clostridia.o_Clostridiales.f_Lachnospiraceae.g_Lachnospiraceae_noname.s_Lachnospiraceae_bacterium_3_1_46FAA)	MR Egger	5	-0.11046	0.19627	0.61288	-0.495156	0.27423	0.89542	0.60948	1.31551	0.992584069			
CD38 on IgD+ CD38+ B cell	ebi-a-GCST90001812	ebi-a-GCST90027850	Gut microbiota abundance (k_Bacteria.p_Firmicutes.c_Clostridia.o_Clostridiales.f_Lachnospiraceae.g_Lachnospiraceae_noname.s_Lachnospiraceae_bacterium_3_1_46FAA)	Weighted median	5	-0.17178	0.10123	0.08969	-0.370183	0.02662	0.84216	0.69061	1.02698	0.987483487			
CD38 on IgD+ CD38+ B cell	ebi-a-GCST90001812	ebi-a-GCST90027850	Gut microbiota abundance (k_Bacteria.p_Firmicutes.c_Clostridia.o_Clostridiales.f_Lachnospiraceae.g_Lachnospiraceae_noname.s_Lachnospiraceae_bacterium_3_1_46FAA)	Weighted mode	5	-0.16754	0.12224	0.24238	-0.407129	0.07205	0.84574	0.66556	1.07471	0.993569334			
CD38 on IgD- CD38dim B cell	ebi-a-GCST90001815	ebi-a-GCST90027674	Gut microbiota abundance (k_Bacteria.p_Firmicutes.c_Clostridia.o_Clostridiales.f_Lachnospiraceae)	Inverse variance weighted	5	0.13478	0.06664	0.04313	0.004161	0.26539	1.14428	1.00417	1.30394	0.99011796	0.478215705	0.373265934	0.7613333
CD38 on IgD- CD38dim B cell	ebi-a-GCST90001815	ebi-a-GCST90027674	Gut microbiota abundance (k_Bacteria.p_Firmicutes.c_Clostridia.o_Clostridiales.f_Lachnospiraceae)	MR Egger	5	0.4461	0.39156	0.33732	-0.32136	1.21357	1.56222	0.72516	3.36548	0.992584069			
CD38 on IgD- CD38dim B cell	ebi-a-GCST90001815	ebi-a-GCST90027674	Gut microbiota abundance (k_Bacteria.p_Firmicutes.c_Clostridia.o_Clostridiales.f_Lachnospiraceae)	Weighted median	5	0.14328	0.08773	0.10242	-0.028666	0.31523	1.15405	0.97174	1.37057	0.987483487			
CD38 on IgD- CD38dim B cell	ebi-a-GCST90001815	ebi-a-GCST90027674	Gut microbiota abundance (k_Bacteria.p_Firmicutes.c_Clostridia.o_Clostridiales.f_Lachnospiraceae)	Weighted mode	5	0.19963	0.12366	0.18175	-0.042741	0.44201	1.22095	0.95816	1.55582	0.993569334			
HVEM on Central Memory CD8+ T cell	ebi-a-GCST90001882	ebi-a-GCST90027674	Gut microbiota abundance (k_Bacteria.p_Firmicutes.c_Clostridia.o_Clostridiales.f_Lachnospiraceae)	Inverse variance weighted	15	0.04176	0.01726	0.01551	0.0079407	0.07558	1.04265	1.00797	1.07851	0.99011796	0.144837234	0.23243876	0.6743333
HVEM on Central Memory CD8+ T cell	ebi-a-GCST90001882	ebi-a-GCST90027674	Gut microbiota abundance (k_Bacteria.p_Firmicutes.c_Clostridia.o_Clostridiales.f_Lachnospiraceae)	MR Egger	15	0.00359	0.0296	0.90522	-0.054416	0.0616	1.0036	0.94704	1.06354	0.992584069			
HVEM on Central Memory CD8+ T cell	ebi-a-GCST90001882	ebi-a-GCST90027674	Gut microbiota abundance (k_Bacteria.p_Firmicutes.c_Clostridia.o_Clostridiales.f_Lachnospiraceae)	Weighted median	15	0.01055	0.02235	0.63691	-0.033253	0.05435	1.0106	0.96729	1.05585	0.987483487			
HVEM on Central Memory CD8+ T cell	ebi-a-GCST90001882	ebi-a-GCST90027674	Gut microbiota abundance (k_Bacteria.p_Firmicutes.c_Clostridia.o_Clostridiales.f_Lachnospiraceae)	Weighted mode	15	0.01101	0.02187	0.62239	-0.031848	0.05387	1.01107	0.96865	1.05535	0.993569334			

**Mendelian Randomization analysis on the causal effect between gut flora and inflammatory proteins.**																		
**exposure**	**id.exposure**	**id.outcome**	**outcome**	**method**	**nsnp**	**b**	**se**	**pval**	**lo_ci**	**up_ci**	**or**	**or_lci95**	**or_uci95**	**p_fdr**	**P for pleiotropy**	**P for heterogeneity**	**P for Global Test**
Gut microbiota abundance (k_Bacteria.p_Firmicutes.c_Clostridia.o_Clostridiales.f_Oscillospiraceae)	ebi-a-GCST90027675	ebi-IPs91-GCST90274828	Neurturin levels	Inverse variance weighted	8	-0.17282	0.06088	0.00453	-0.292149	-0.05348	0.84129	0.74666	0.94792	0.190389322	0.577064907	0.749525798	0.7536667
Gut microbiota abundance (k_Bacteria.p_Firmicutes.c_Clostridia.o_Clostridiales.f_Oscillospiraceae)	ebi-a-GCST90027675	ebi-IPs91-GCST90274828	Neurturin levels	MR Egger	8	-0.33975	0.28967	0.28529	-0.907502	0.228	0.71195	0.40353	1.25609	0.996424849			
Gut microbiota abundance (k_Bacteria.p_Firmicutes.c_Clostridia.o_Clostridiales.f_Oscillospiraceae)	ebi-a-GCST90027675	ebi-IPs91-GCST90274828	Neurturin levels	Weighted median	8	-0.14544	0.08037	0.07034	-0.302954	0.01208	0.86464	0.73863	1.01215	0.924952448			
Gut microbiota abundance (k_Bacteria.p_Firmicutes.c_Clostridia.o_Clostridiales.f_Oscillospiraceae)	ebi-a-GCST90027675	ebi-IPs91-GCST90274828	Neurturin levels	Weighted mode	8	-0.12365	0.12891	0.36943	-0.37631	0.12902	0.88369	0.68639	1.13771	0.998762729			

**Mendelian Randomization analysis on the causal effect between immune cell characteristics and metabolites.**																	
**exposure**	**id.exposure**	**id.outcome**	**outcome**	**method**	**nsnp**	**b**	**se**	**pval**	**lo_ci**	**up_ci**	**or**	**or_lci95**	**or_uci95**	**p_fdr**	**P for pleiotropy**	**P for heterogeneity**	**P for Global Test**
IgD- CD27- B cell %lymphocyte	ebi-a-GCST90001433	ebi-met1400-GCST90199682	Homocitrulline levels	Inverse variance weighted	18	-0.05566	0.02717	0.04054	-0.10892	-0.0024	0.94586	0.8968	0.99761	0.952042913	0.436044707	0.644008413	0.8346667
IgD- CD27- B cell %lymphocyte	ebi-a-GCST90001433	ebi-met1400-GCST90199682	Homocitrulline levels	MR Egger	18	-0.00508	0.0689	0.94219	-0.14012	0.12997	0.99494	0.86925	1.13879	0.999498649			
IgD- CD27- B cell %lymphocyte	ebi-a-GCST90001433	ebi-met1400-GCST90199682	Homocitrulline levels	Weighted median	18	-0.0346	0.03742	0.35511	-0.10795	0.03874	0.96599	0.89767	1.0395	0.999888785			
IgD- CD27- B cell %lymphocyte	ebi-a-GCST90001433	ebi-met1400-GCST90199682	Homocitrulline levels	Weighted mode	18	-0.02445	0.04595	0.60157	-0.114511	0.06561	0.97585	0.8918	1.06781	0.996406102			
IgD- CD27- B cell %lymphocyte	ebi-a-GCST90001433	ebi-met1400-GCST90200116	Ceramide (d18:1/14:0, d16:1/16:0) levels	Inverse variance weighted	18	-0.07723	0.03226	0.01666	-0.140451	-0.014	0.92568	0.86897	0.98609	0.818198593	0.542418405	0.250178062	0.7813333
IgD- CD27- B cell %lymphocyte	ebi-a-GCST90001433	ebi-met1400-GCST90200116	Ceramide (d18:1/14:0, d16:1/16:0) levels	MR Egger	18	-0.12496	0.08342	0.15363	-0.288464	0.03855	0.88254	0.74941	1.0393	0.999498649			
IgD- CD27- B cell %lymphocyte	ebi-a-GCST90001433	ebi-met1400-GCST90200116	Ceramide (d18:1/14:0, d16:1/16:0) levels	Weighted median	18	-0.06129	0.04328	0.15674	-0.146123	0.02354	0.94055	0.86405	1.02382	0.999888785			
IgD- CD27- B cell %lymphocyte	ebi-a-GCST90001433	ebi-met1400-GCST90200116	Ceramide (d18:1/14:0, d16:1/16:0) levels	Weighted mode	18	-0.05861	0.05539	0.30484	-0.167176	0.04996	0.94308	0.84605	1.05123	0.996406102			
CD11c+ monocyte %monocyte	ebi-a-GCST90001449	ebi-met1400-GCST90200295	Gamma-glutamyltyrosine levels	Inverse variance weighted	21	-0.05319	0.02286	0.01995	-0.097995	-0.00839	0.9482	0.90665	0.99164	0.818198593	0.213130425	0.652081344	0.7623333
CD11c+ monocyte %monocyte	ebi-a-GCST90001449	ebi-met1400-GCST90200295	Gamma-glutamyltyrosine levels	MR Egger	21	-0.00636	0.04295	0.88391	-0.090532	0.07782	0.99366	0.91345	1.08093	0.999498649			
CD11c+ monocyte %monocyte	ebi-a-GCST90001449	ebi-met1400-GCST90200295	Gamma-glutamyltyrosine levels	Weighted median	21	-0.02	0.03484	0.47706	-0.093	0.04352	0.97553	0.91114	1.04448	0.999888785			

						477			061								
CD11c+ monocyte %monocyte	ebi-a-GCST90001449	ebi-met1400-GCST90200295	Gamma-glutamyltyrosine levels	Weighted mode	21	-0.008837	0.04064	0.83898	-0.088018	0.07129	0.99167	0.91574	1.07389	0.996406102			
Plasmacytoid Dendritic Cell %Dendritic Cell	ebi-a-GCST90001474	ebi-met1400-GCST90199917	Pregnanediol-3-glucuronide levels	Inverse variance weighted	22	0.04525	0.0166	0.00641	0.0127119	0.07778	1.04629	1.01279	1.08089	0.818198593	0.861750647	0.789131921	0.5346667
Plasmacytoid Dendritic Cell %Dendritic Cell	ebi-a-GCST90001474	ebi-met1400-GCST90199917	Pregnanediol-3-glucuronide levels	MR Egger	22	0.04259	0.02242	0.072	-0.001353	0.08653	1.04351	0.99865	1.09038	0.999498649			
Plasmacytoid Dendritic Cell %Dendritic Cell	ebi-a-GCST90001474	ebi-met1400-GCST90199917	Pregnanediol-3-glucuronide levels	Weighted median	22	0.0394	0.02596	0.12897	-0.011468	0.09028	1.04019	0.9886	1.09448	0.999888785			
Plasmacytoid Dendritic Cell %Dendritic Cell	ebi-a-GCST90001474	ebi-met1400-GCST90199917	Pregnanediol-3-glucuronide levels	Weighted mode	22	0.04368	0.0219	0.05927	0.0007492	0.08661	1.04464	1.00075	1.09047	0.996406102			
CD28+ CD45RA+ CD8+ T cell Absolute Count	ebi-a-GCST90001690	ebi-met1400-GCST90199961	Methionine sulfone levels	Inverse variance weighted	57	0.00594	0.00302	0.04894	2.76E-05	0.01186	1.00596	1.00003	1.01193	0.970389915	0.5859271 3	0.280863406	0.6913333
CD28+ CD45RA+ CD8+ T cell Absolute Count	ebi-a-GCST90001690	ebi-met1400-GCST90199961	Methionine sulfone levels	MR Egger	57	0.00661	0.00327	0.0482	0.0001982	0.01302	1.00663	1.0002	1.0131	0.999498649			
CD28+ CD45RA+ CD8+ T cell Absolute Count	ebi-a-GCST90001690	ebi-met1400-GCST90199961	Methionine sulfone levels	Weighted median	57	0.00102	0.00445	0.81898	-0.007703	0.00974	1.00102	0.99233	1.00979	0.999888785			
CD28+ CD45RA+ CD8+ T cell Absolute Count	ebi-a-GCST90001690	ebi-met1400-GCST90199961	Methionine sulfone levels	Weighted mode	57	0.00171	0.00375	0.65111	-0.00565	0.00906	1.00171	0.99437	1.0091	0.996406102			
CD28+ CD45RA+ CD8+ T cell Absolute Count	ebi-a-GCST90001690	ebi-met1400-GCST90200239	Tetradecadienedioate (C14:2-DC) levels	Inverse variance weighted	57	0.00695	0.00306	0.02334	0.0009429	0.01295	1.00697	1.00094	1.01303	0.818198593	0.5952901 6	0.439098622	0.564
CD28+ CD45RA+ CD8+ T cell Absolute Count	ebi-a-GCST90001690	ebi-met1400-GCST90200239	Tetradecadienedioate (C14:2-DC) levels	MR Egger	57	0.00629	0.00332	0.06324	-0.000213	0.01279	1.00631	0.99979	1.01287	0.999498649			
CD28+ CD45RA+ CD8+ T cell Absolute Count	ebi-a-GCST90001690	ebi-met1400-GCST90200239	Tetradecadienedioate (C14:2-DC) levels	Weighted median	57	0.00263	0.0049	0.59056	-0.006962	0.01223	1.00264	0.99306	1.01231	0.999888785			
CD28+ CD45RA+ CD8+ T cell Absolute Count	ebi-a-GCST90001690	ebi-met1400-GCST90200239	Tetradecadienedioate (C14:2-DC) levels	Weighted mode	57	0.00381	0.00409	0.35549	-0.004201	0.01181	1.00381	0.99581	1.01188	0.996406102			
CD20 on CD20- CD38- B cell	ebi-a-GCST90001744	ebi-met1400-GCST90200234	Picolinoylglycine levels	Inverse variance weighted	16	0.08294	0.03748	0.02689	0.0094854	0.15639	1.08648	1.00953	1.16929	0.84128071	0.8786490 8	0.088106358	0.563333
CD20 on CD20- CD38- B cell	ebi-a-GCST90001744	ebi-met1400-GCST90200234	Picolinoylglycine levels	MR Egger	16	0.0744	0.06719	0.28681	-0.057293	0.2061	1.07724	0.94432	1.22888	0.999498649			
CD20 on CD20- CD38- B cell	ebi-a-GCST90001744	ebi-met1400-GCST90200234	Picolinoylglycine levels	Weighted median	16	0.0792	0.04788	0.0981	-0.014643	0.17303	1.08242	0.98546	1.18891	0.999888785			
CD20 on CD20- CD38- B cell	ebi-a-GCST90001744	ebi-met1400-GCST90200234	Picolinoylglycine levels	Weighted mode	16	0.07871	0.04666	0.11235	-0.012755	0.17017	1.08189	0.98733	1.18551	0.996406102			
IgD on unswitched memory B cell	ebi-a-GCST90001826	ebi-met1400-GCST90200059	Behenoyl dihydrosphingomyelin (d18:0/22:0) levels	Inverse variance weighted	19	-0.06269	0.02247	0.00528	-0.106741	-0.01864	0.93923	0.89876	0.98153	0.818198593	0.544696339	0.549315967	0.736667
IgD on unswitched memory B cell	ebi-a-GCST90001826	ebi-met1400-GCST90200059	Behenoyl dihydrosphingomyelin (d18:0/22:0) levels	MR Egger	19	-0.07	0.03444	0.03518	-0.146	-0.01	0.9242	0.86387	0.98875	0.999498649			

						882			333	132							
IgD on unswitched memory B cell	ebi-a-GCST90001826	ebi-met1400-GCST90200059	Behenoyl dihydrosphingomyelin (d18:0/22:0) levels	Weighted median	19	-0.080809	0.03617	0.02531	-0.151783	-0.01	0.92229	0.85917	0.99005	0.999888785			
IgD on unswitched memory B cell	ebi-a-GCST90001826	ebi-met1400-GCST90200059	Behenoyl dihydrosphingomyelin (d18:0/22:0) levels	Weighted mode	19	-0.087885	0.04085	0.04534	-0.167907	-0.007779	0.9159	0.84543	0.99224	0.996406102			
IgD on unswitched memory B cell	ebi-a-GCST90001826	ebi-met1400-GCST90200295	Gamma-glutamyltyrosine levels	Inverse variance weighted	19	-0.045446	0.02294	0.04753	-0.090435	-0.00049	0.95555	0.91353	0.99951	0.970389915	0.799983001	0.613909747	0.7346667
IgD on unswitched memory B cell	ebi-a-GCST90001826	ebi-met1400-GCST90200295	Gamma-glutamyltyrosine levels	MR Egger	19	-0.052334	0.03521	0.15549	-0.121355	0.01668	0.94901	0.88572	1.01682	0.999498649			
IgD on unswitched memory B cell	ebi-a-GCST90001826	ebi-met1400-GCST90200295	Gamma-glutamyltyrosine levels	Weighted median	19	-0.060445	0.03477	0.08214	-0.128604	0.00771	0.94134	0.87932	1.00774	0.999888785			
IgD on unswitched memory B cell	ebi-a-GCST90001826	ebi-met1400-GCST90200295	Gamma-glutamyltyrosine levels	Weighted mode	19	-0.0557	0.03985	0.17923	-0.133805	0.02241	0.94583	0.87476	1.02267	0.996406102			
CD3 on Effector Memory CD4+ T cell	ebi-a-GCST90001843	ebi-met1400-GCST90199785	Isovalerylglycine levels	Inverse variance weighted	18	0.053733	0.02227	0.01585	0.0100772	0.097739	1.0552	1.01013	1.10229	0.818198593	0.87168764	0.075161633	0.2813333
CD3 on Effector Memory CD4+ T cell	ebi-a-GCST90001843	ebi-met1400-GCST90199785	Isovalerylglycine levels	MR Egger	18	0.05814	0.0353	0.11911	-0.011106	0.127733	1.05986	0.989	1.1358	0.999498649			
CD3 on Effector Memory CD4+ T cell	ebi-a-GCST90001843	ebi-met1400-GCST90199785	Isovalerylglycine levels	Weighted median	18	0.060886	0.02927	0.03757	0.0034968	0.118822	1.06275	1.0035	1.12549	0.999888785			
CD3 on Effector Memory CD4+ T cell	ebi-a-GCST90001843	ebi-met1400-GCST90199785	Isovalerylglycine levels	Weighted mode	18	0.074644	0.0291	0.02008	0.0176605	0.131667	1.07749	1.01776	1.14073	0.996406102			

Mendelian Randomization analysis on the causal effect between metabolites and immune cell characteristics.

exposure	id.exposure	id.outcome	outcome	method	nsnp	b	se	pval	lo_ci	up_ci	or	or_lci95	or_uci95	p_fdr	P for pleiotropy	P for heterogeneity	P for Global Test
Erythronate levels	ebi-met1400-GCST90199746	ebi-a-GCST90001449	CD11c+ monocyte %monocyte	Inverse variance weighted	18	0.284481	0.07937	0.00033	0.1292376	0.440038	1.32951	1.13796	1.5533	0.276672794	0.738422799	0.582183308	0.2753333
Erythronate levels	ebi-met1400-GCST90199746	ebi-a-GCST90001449	CD11c+ monocyte %monocyte	MR Egger	18	0.337793	0.17533	0.07188	-0.005718	0.681159	1.40205	0.9943	1.97701	0.999977204			
Erythronate levels	ebi-met1400-GCST90199746	ebi-a-GCST90001449	CD11c+ monocyte %monocyte	Weighted median	18	0.239901	0.11273	0.03399	0.0180524	0.459996	1.26999	1.01822	1.58401	0.993735727			
Erythronate levels	ebi-met1400-GCST90199746	ebi-a-GCST90001449	CD11c+ monocyte %monocyte	Weighted mode	18	0.151553	0.17199	0.39058	-0.185566	0.488862	1.16361	0.83063	1.63006	0.998913676			
S-allylcysteine levels	ebi-met1400-GCST90199948	ebi-a-GCST90001506	CD25++ CD4+ T cell %T cell	Inverse variance weighted	17	-0.171669	0.08181	0.03585	-0.332032	-0.011134	0.84224	0.71746	0.98872	0.999092064	0.82104983	0.883745142	0.766667
S-allylcysteine levels	ebi-met1400-GCST90199948	ebi-a-GCST90001506	CD25++ CD4+ T cell %T cell	MR Egger	17	-0.124408	0.22241	0.58517	-0.560011	0.311186	0.88331	0.5712	1.36596	0.999977204			
S-allylcysteine levels	ebi-met1400-GCST90199948	ebi-a-GCST90001506	CD25++ CD4+ T cell %T cell	Weighted median	17	-0.19	0.10694	0.07525	-0.399	0.01937	0.82676	0.67043	1.01955	0.993735727			

						023			835								
S-allylcysteine levels	ebi-met1400-GCST90199948	ebi-a-GCST90001506	CD25++ CD4+ T cell %T cell	Weighted mode	17	-0.236657	0.146661	0.126617	-0.523933	0.050779	0.789333	0.592219	1.05211	0.998913676			
4-hydroxychlorothalonil levels	ebi-met1400-GCST90199977	ebi-a-GCST90001449	CD11c+ monocyte %monocyte	Inverse variance weighted	21	-0.128896	0.062262	0.039445	-0.251695	-0.006233	0.879001	0.777748	0.99379	0.999092064	0.338329893	0.45488477	0.642
4-hydroxychlorothalonil levels	ebi-met1400-GCST90199977	ebi-a-GCST90001449	CD11c+ monocyte %monocyte	MR Egger	21	-0.212239	0.105556	0.058861	-0.419276	-0.0055	0.808665	0.657552	0.99451	0.999977204			
4-hydroxychlorothalonil levels	ebi-met1400-GCST90199977	ebi-a-GCST90001449	CD11c+ monocyte %monocyte	Weighted median	21	-0.148876	0.094559	0.115581	-0.334157	0.036664	0.861778	0.715594	1.03732	0.993735727			
4-hydroxychlorothalonil levels	ebi-met1400-GCST90199977	ebi-a-GCST90001449	CD11c+ monocyte %monocyte	Weighted mode	21	-0.180553	0.088886	0.055568	-0.354693	-0.006637	0.834483	0.701319	0.99365	0.998913676			
Lignoceroyl sphingomyelin (d18:1/24:0) levels	ebi-met1400-GCST90200020	ebi-a-GCST90001449	CD11c+ monocyte %monocyte	Inverse variance weighted	24	-0.143349	0.070779	0.042666	-0.282229	-0.004475	0.866633	0.7541	0.99526	0.999092064	0.299217325	0.687466765	0.4913333
Lignoceroyl sphingomyelin (d18:1/24:0) levels	ebi-met1400-GCST90200020	ebi-a-GCST90001449	CD11c+ monocyte %monocyte	MR Egger	24	-0.272267	0.140662	0.065543	-0.548292	0.002994	0.761334	0.577794	1.00295	0.999977204			
Lignoceroyl sphingomyelin (d18:1/24:0) levels	ebi-met1400-GCST90200020	ebi-a-GCST90001449	CD11c+ monocyte %monocyte	Weighted median	24	-0.232237	0.1	0.020015	-0.428377	-0.036366	0.792665	0.651577	0.96429	0.993735727			
Lignoceroyl sphingomyelin (d18:1/24:0) levels	ebi-met1400-GCST90200020	ebi-a-GCST90001449	CD11c+ monocyte %monocyte	Weighted mode	24	-0.197764	0.122667	0.120779	-0.438072	0.042279	0.820667	0.645528	1.04372	0.998913676			
Oleate/vaccenate (18:1) levels	ebi-met1400-GCST90200042	ebi-a-GCST90001641	CD3- lymphocyte %leukocyte	Inverse variance weighted	21	0.146681	0.071995	0.041129	0.0058019	0.287783	1.158814	1.005822	1.33353	0.999092064	0.745901185	0.809285023	0.3876667
Oleate/vaccenate (18:1) levels	ebi-met1400-GCST90200042	ebi-a-GCST90001641	CD3- lymphocyte %leukocyte	MR Egger	21	0.097777	0.1656	0.561886	-0.226798	0.422334	1.10271	0.797708	1.52553	0.999977204			
Oleate/vaccenate (18:1) levels	ebi-met1400-GCST90200042	ebi-a-GCST90001641	CD3- lymphocyte %leukocyte	Weighted median	21	0.125539	0.104661	0.230666	-0.079643	0.330042	1.133559	0.923345	1.39156	0.993735727			
Oleate/vaccenate (18:1) levels	ebi-met1400-GCST90200042	ebi-a-GCST90001641	CD3- lymphocyte %leukocyte	Weighted mode	21	0.138848	0.142214	0.341555	-0.140107	0.417707	1.148853	0.869226	1.51751	0.998913676			
Ceramide (d18:1/14:0, d16:1/16:0) levels	ebi-met1400-GCST90200116	ebi-a-GCST90001449	CD11c+ monocyte %monocyte	Inverse variance weighted	25	-0.120555	0.060773	0.047716	-0.23959	-0.001511	0.886643	0.786695	0.99849	0.999092064	0.50464611	0.665931622	0.5236667
Ceramide (d18:1/14:0, d16:1/16:0) levels	ebi-met1400-GCST90200116	ebi-a-GCST90001449	CD11c+ monocyte %monocyte	MR Egger	25	-0.202227	0.135	0.147665	-0.466868	0.062233	0.816887	0.626696	1.06431	0.999977204			
Ceramide (d18:1/14:0, d16:1/16:0) levels	ebi-met1400-GCST90200116	ebi-a-GCST90001449	CD11c+ monocyte %monocyte	Weighted median	25	-0.108662	0.087716	0.212267	-0.27946	0.062221	0.897077	0.756619	1.06419	0.993735727			
Ceramide (d18:1/14:0, d16:1/16:0) levels	ebi-met1400-GCST90200116	ebi-a-GCST90001449	CD11c+ monocyte %monocyte	Weighted mode	25	-0.111145	0.094334	0.249905	-0.296354	0.073346	0.894454	0.743352	1.07623	0.998913676			
Dihydroferulic acid sulfate levels	ebi-met1400-	ebi-a-GCST90001519	CD33+ HLA DR+ CD14- %CD33+ HLA DR+	Inverse variance	26	0.1627	0.07	0.02	0.024	0.3008	1.17	1.02	1.35094	0.99909	0.8080396	0.845825476	0.8123333

	GCST90200166			weighted			046	093	6037		669	491		2064			
Dihydroferulic acid sulfate levels	ebi-met1400-GCST90200166	ebi-a-GCST90001519	CD33+ HLA DR+ CD14- %CD33+ HLA DR+	MR Egger	26	0.188835	0.125597	0.147788	-0.05854	0.435525	1.207726	0.943314	1.54534	0.999977204			
Dihydroferulic acid sulfate levels	ebi-met1400-GCST90200166	ebi-a-GCST90001519	CD33+ HLA DR+ CD14- %CD33+ HLA DR+	Weighted median	26	0.235572	0.103558	0.022286	0.0327052	0.438873	1.265582	1.033325	1.55074	0.993735727			
Dihydroferulic acid sulfate levels	ebi-met1400-GCST90200166	ebi-a-GCST90001519	CD33+ HLA DR+ CD14- %CD33+ HLA DR+	Weighted mode	26	0.202203	0.107558	0.072209	-0.0088821	0.412889	1.223889	0.991222	1.51117	0.998913676			
Caprate (10:0) levels	ebi-met1400-GCST90200428	ebi-a-GCST90001449	CD11c+ monocyte %monocyte	Inverse variance weighted	13	-0.199665	0.094889	0.035377	-0.3856366	-0.013667	0.819001	0.680002	0.98642	0.999092064	0.666098074	0.813632882	0.643333
Caprate (10:0) levels	ebi-met1400-GCST90200428	ebi-a-GCST90001449	CD11c+ monocyte %monocyte	MR Egger	13	-0.2924	0.2297	0.229928	-0.7426617	0.157882	0.746647	0.475587	1.17095	0.999977204			
Caprate (10:0) levels	ebi-met1400-GCST90200428	ebi-a-GCST90001449	CD11c+ monocyte %monocyte	Weighted median	13	-0.2716	0.122668	0.026683	-0.5120533	-0.031116	0.762216	0.599926	0.96932	0.993735727			
Caprate (10:0) levels	ebi-met1400-GCST90200428	ebi-a-GCST90001449	CD11c+ monocyte %monocyte	Weighted mode	13	-0.308788	0.195598	0.141111	-0.6929912	0.075334	0.734434	0.500012	1.07825	0.998913676			

Mendelian Randomization analysis on the causal effect between inflammatory proteins and metabolites.

exposure	id.exposure	id.outcome	outcome	method	nsnp	b	se	pval	lo_ci	up_ci	or	or_lci95	or_uci95	p_fdr	P for pleiotropy	P for heterogeneity	P for Global Test
Caspase 8 levels	ebi-IPs91-GCST90274763	ebi-met1400-GCST90199725	Campesterol levels	Inverse variance weighted	20	0.168828	0.079923	0.033668	0.01229871	0.323357	1.183327	1.013307	1.38205	0.7973452	0.188152095	0.154413719	0.483333
Caspase 8 levels	ebi-IPs91-GCST90274763	ebi-met1400-GCST90199725	Campesterol levels	MR Egger	20	0.366639	0.164244	0.038665	0.04448	0.688829	1.442551	1.045548	1.99031	0.86181566			
Caspase 8 levels	ebi-IPs91-GCST90274763	ebi-met1400-GCST90199725	Campesterol levels	Weighted median	20	0.355545	0.0984	0.0003	0.16257 49	0.548832	1.426882	1.176654	1.73034	0.129379254			
Caspase 8 levels	ebi-IPs91-GCST90274763	ebi-met1400-GCST90199725	Campesterol levels	Weighted mode	20	0.375505	0.119833	0.005552	0.14018 62	0.609992	1.455507	1.150449	1.84029	0.938470216			
Interleukin-18 levels	ebi-IPs91-GCST90274803	ebi-met1400-GCST90199684	1-linoleoylglycerol (18:2) levels	Inverse variance weighted	28	-0.0969	0.040001	0.015444	-0.1753325	-0.018448	0.907644	0.839918	0.98169	0.7973452	0.154002789	0.755733371	0.648
Interleukin-18 levels	ebi-IPs91-GCST90274803	ebi-met1400-GCST90199684	1-linoleoylglycerol (18:2) levels	MR Egger	28	-0.204667	0.083359	0.0214	-0.3685144	-0.040083	0.814491	0.691776	0.95999	0.86181566			
Interleukin-18 levels	ebi-IPs91-GCST90274803	ebi-met1400-GCST90199684	1-linoleoylglycerol (18:2) levels	Weighted median	28	-0.127749	0.0572	0.025844	-0.2396067	-0.015366	0.880031	0.786944	0.98475	0.904923048			
Interleukin-18 levels	ebi-IPs91-GCST90274803	ebi-met1400-GCST90199684	1-linoleoylglycerol (18:2) levels	Weighted mode	28	-0.127741	0.064464	0.059044	-0.2541011	-0.000072	0.880037	0.775561	0.99928	0.938470216			
Interleukin-18 levels	ebi-IPs91-GCST90274803	ebi-met1400-GCST90199689	Glycolithocholate levels	Inverse variance weighted	29	0.109946	0.042272	0.010441	0.02572 08	0.193319	1.115567	1.026605	1.21311	0.7973452	0.523252535	0.610873576	0.7856667
Interleukin-18 levels	ebi-IPs91-GCST90274803	ebi-met1400-GCST90199689	Glycolithocholate levels	MR Egger	29	0.059779	0.087888	0.502005	-0.1124 46	0.232203	1.061611	0.893655	1.26115	0.952872936			

Interleukin-18 levels	ebi-IPs91-GCST90274803	ebi-met1400-GCST90199689	Glycolithocholate levels	Weighted median	29	0.12269	0.06299	0.05145	-0.000776	0.24615	1.13053	0.99922	1.27909	0.904923048		
Interleukin-18 levels	ebi-IPs91-GCST90274803	ebi-met1400-GCST90199689	Glycolithocholate levels	Weighted mode	29	0.16723	0.06851	0.02123	0.0329479	0.30152	1.18203	1.0335	1.35191	0.938470216		
Interleukin-18 levels	ebi-IPs91-GCST90274803	ebi-met1400-GCST90199744	Eicosenoate (20:1) levels	Inverse variance weighted	29	-0.08244	0.03914	0.03519	-0.159163	-0.00572	0.92087	0.85286	0.9943	0.7973452	0.377906593	0.441419704	0.3913333
Interleukin-18 levels	ebi-IPs91-GCST90274803	ebi-met1400-GCST90199744	Eicosenoate (20:1) levels	MR Egger	29	-0.14566	0.08072	0.08232	-0.303879	0.01255	0.86445	0.73795	1.01263	0.86181566		
Interleukin-18 levels	ebi-IPs91-GCST90274803	ebi-met1400-GCST90199744	Eicosenoate (20:1) levels	Weighted median	29	-0.07643	0.05748	0.18359	-0.18909	0.03622	0.92642	0.82771	1.03689	0.904923048		
Interleukin-18 levels	ebi-IPs91-GCST90274803	ebi-met1400-GCST90199744	Eicosenoate (20:1) levels	Weighted mode	29	-0.06453	0.0607	0.29681	-0.183511	0.05444	0.9375	0.83234	1.05595	0.938470216		
Interleukin-18 levels	ebi-IPs91-GCST90274803	ebi-met1400-GCST90200250	Deoxycholic acid 12-sulfate levels	Inverse variance weighted	29	0.10012	0.04477	0.02533	0.0123726	0.18786	1.1053	1.01245	1.20666	0.7973452	0.580782911	0.255967874	0.6386667
Interleukin-18 levels	ebi-IPs91-GCST90274803	ebi-met1400-GCST90200250	Deoxycholic acid 12-sulfate levels	MR Egger	29	0.14555	0.09306	0.12947	-0.036856	0.32796	1.15668	0.96381	1.38813	0.934823669		
Interleukin-18 levels	ebi-IPs91-GCST90274803	ebi-met1400-GCST90200250	Deoxycholic acid 12-sulfate levels	Weighted median	29	0.06067	0.05826	0.29778	-0.053533	0.17487	1.06254	0.94787	1.19109	0.904923048		
Interleukin-18 levels	ebi-IPs91-GCST90274803	ebi-met1400-GCST90200250	Deoxycholic acid 12-sulfate levels	Weighted mode	29	0.05372	0.05891	0.36958	-0.061738	0.16918	1.05519	0.94013	1.18433	0.938470216		
Interleukin-6 levels	ebi-IPs91-GCST90274815	ebi-met1400-GCST90199791	1-arachidonoyl-GPE (20:4n6) levels	Inverse variance weighted	13	-0.12005	0.06	0.04542	-0.237653	-0.00245	0.88688	0.78848	0.99756	0.804955234	0.7237753	0.762848443	0.673333
Interleukin-6 levels	ebi-IPs91-GCST90274815	ebi-met1400-GCST90199791	1-arachidonoyl-GPE (20:4n6) levels	MR Egger	13	-0.08429	0.11545	0.48059	-0.310563	0.14199	0.91917	0.73303	1.15256	0.952872936		
Interleukin-6 levels	ebi-IPs91-GCST90274815	ebi-met1400-GCST90199791	1-arachidonoyl-GPE (20:4n6) levels	Weighted median	13	-0.13985	0.08074	0.08326	-0.298101	0.0184	0.86949	0.74223	1.01857	0.904923048		
Interleukin-6 levels	ebi-IPs91-GCST90274815	ebi-met1400-GCST90199791	1-arachidonoyl-GPE (20:4n6) levels	Weighted mode	13	-0.15243	0.08756	0.10725	-0.324049	0.01918	0.85862	0.72322	1.01937	0.938470216		
Neurturin levels	ebi-IPs91-GCST90274828	ebi-met1400-GCST90200331	1-palmitoyl-2-oleoyl-GPE (16:0/18:1) levels	Inverse variance weighted	23	-0.11627	0.05373	0.03045	-0.221576	-0.01097	0.89023	0.80125	0.98909	0.7973452	0.375257523	0.27027808	0.496667
Neurturin levels	ebi-IPs91-GCST90274828	ebi-met1400-GCST90200331	1-palmitoyl-2-oleoyl-GPE (16:0/18:1) levels	MR Egger	23	-0.19978	0.1068	0.07542	-0.409117	0.00956	0.81891	0.66424	1.0096	0.86181566		
Neurturin levels	ebi-IPs91-GCST90274828	ebi-met1400-GCST90200331	1-palmitoyl-2-oleoyl-GPE (16:0/18:1) levels	Weighted median	23	-0.12587	0.07158	0.07866	-0.266158	0.01442	0.88173	0.76632	1.01453	0.904923048		
Neurturin levels	ebi-IPs91-GCST90274828	ebi-met1400-GCST90200331	1-palmitoyl-2-oleoyl-GPE (16:0/18:1) levels	Weighted mode	23	-0.21883	0.12051	0.08304	-0.455035	0.01737	0.80346	0.63443	1.01752	0.938470216		

Mendelian Randomization analysis on the causal effect between metabolites and inflammatory proteins.

exposure	id.exposure	id.outcome	outcome	method	nsnp	b	se	pval	lo_ci	up_ci	or	or_lci95	or_uci95	p_fdr	P for pleiotropy	P for heterogeneity	P for Global Test
Erythronate levels	ebi-met1400-GCST90199746	ebi-IPs91-GCST90274803	Interleukin-18 levels	Inverse variance weighted	20	0.087332	0.044409	0.047763	0.0009089	0.173722	1.091224	1.000091	1.18973	0.75156385	0.730211803	0.075329344	0.742
Erythronate levels	ebi-met1400-GCST90199746	ebi-IPs91-GCST90274803	Interleukin-18 levels	MR Egger	20	0.054977	0.102788	0.599288	-0.146476	0.256422	1.056511	0.863775	1.2923	0.996820926			
Erythronate levels	ebi-met1400-GCST90199746	ebi-IPs91-GCST90274803	Interleukin-18 levels	Weighted median	20	0.015553	0.054406	0.773933	-0.090434	0.121149	1.015565	0.913533	1.12918	0.977240101			
Erythronate levels	ebi-met1400-GCST90199746	ebi-IPs91-GCST90274803	Interleukin-18 levels	Weighted mode	20	0.003329	0.067922	0.961922	-0.12984	0.136641	1.003329	0.878824	1.14615	0.995717708			
N-acetylcarnosine levels	ebi-met1400-GCST90199926	ebi-IPs91-GCST90274768	C-C motif chemokine 25 levels	Inverse variance weighted	29	-0.052	0.025771	0.043112	-0.102383	-0.00161	0.949333	0.902268	0.99839	0.745445352	0.220021065	0.750572209	0.6743333
N-acetylcarnosine levels	ebi-met1400-GCST90199926	ebi-IPs91-GCST90274768	C-C motif chemokine 25 levels	MR Egger	29	-0.0049	0.045547	0.915054	-0.094028	0.084423	0.995111	0.910226	1.08788	0.996820926			
N-acetylcarnosine levels	ebi-met1400-GCST90199926	ebi-IPs91-GCST90274768	C-C motif chemokine 25 levels	Weighted median	29	-0.02316	0.038834	0.545588	-0.098302	0.051199	0.977111	0.906638	1.05337	0.977240101			
N-acetylcarnosine levels	ebi-met1400-GCST90199926	ebi-IPs91-GCST90274768	C-C motif chemokine 25 levels	Weighted mode	29	-0.019997	0.036331	0.586766	-0.091133	0.0512	0.98023	0.9129	1.05253	0.995154017			
1-palmitoyl-2-arachidonoyl-GPE (16:0/20:4) levels	ebi-met1400-GCST90200054	ebi-IPs91-GCST90274803	Interleukin-18 levels	Inverse variance weighted	19	-0.041224	0.018889	0.029905	-0.078275	-0.00421	0.9596	0.92471	0.9958	0.651243995	0.289010049	0.610414802	0.6396667
1-palmitoyl-2-arachidonoyl-GPE (16:0/20:4) levels	ebi-met1400-GCST90200054	ebi-IPs91-GCST90274803	Interleukin-18 levels	MR Egger	19	-0.0169	0.033371	0.622557	-0.082961	0.04917	0.98324	0.92039	1.05039	0.996820926			
1-palmitoyl-2-arachidonoyl-GPE (16:0/20:4) levels	ebi-met1400-GCST90200054	ebi-IPs91-GCST90274803	Interleukin-18 levels	Weighted median	19	-0.03731	0.02591	0.14993	-0.088103	0.01348	0.96338	0.91567	1.01357	0.977240101			
1-palmitoyl-2-arachidonoyl-GPE (16:0/20:4) levels	ebi-met1400-GCST90200054	ebi-IPs91-GCST90274803	Interleukin-18 levels	Weighted mode	19	-0.03535	0.02454	0.1685	-0.083448	0.01274	0.96527	0.91994	1.01283	0.995154017			
1-linoleoyl-2-arachidonoyl-GPC (18:2/20:4n6) levels	ebi-met1400-GCST90200073	ebi-IPs91-GCST90274774	T-cell surface glycoprotein CD6 isoform levels	Inverse variance weighted	29	-0.04698	0.02253	0.03701	-0.091135	-0.00283	0.9541	0.91289	0.99717	0.745445352	0.644309499	0.662509415	0.7323333
1-linoleoyl-2-arachidonoyl-GPC (18:2/20:4n6) levels	ebi-met1400-GCST90200073	ebi-IPs91-GCST90274774	T-cell surface glycoprotein CD6 isoform levels	MR Egger	29	-0.03094	0.04109	0.45798	-0.11147	0.04959	0.96954	0.89452	1.05084	0.996820926			
1-linoleoyl-2-arachidonoyl-GPC (18:2/20:4n6) levels	ebi-met1400-GCST90200073	ebi-IPs91-GCST90274774	T-cell surface glycoprotein CD6 isoform levels	Weighted median	29	-0.0648	0.03451	0.06038	-0.13244	0.00283	0.93725	0.87596	1.00283	0.977240101			
1-linoleoyl-2-arachidonoyl-GPC (18:2/20:4n6) levels	ebi-met1400-GCST90200073	ebi-IPs91-GCST90274774	T-cell surface glycoprotein CD6 isoform levels	Weighted mode	29	-0.06984	0.03774	0.07479	-0.143804	0.00412	0.93254	0.86606	1.00413	0.995154017			
1-linoleoyl-2-arachidonoyl-GPC (18:2/20:4n6) levels	ebi-met1400-GCST90200073	ebi-IPs91-GCST90274803	Interleukin-18 levels	Inverse variance weighted	29	-0.04	0.02259	0.03816	-0.091	-0.00	0.95425	0.91291	0.99745	0.745445352	0.296417672	0.703352888	0.5766667

						683			113	256							
1-linoleoyl-2-arachidonoyl-GPC (18:2/20:4n6) levels	ebi-met1400-GCST90200073	ebi-IPs91-GCST90274803	Interleukin-18 levels	MR Egger	29	-0.08351	0.04119	0.05261	-0.16424	-0.00277	0.91988	0.84854	0.99723	0.996820926			
1-linoleoyl-2-arachidonoyl-GPC (18:2/20:4n6) levels	ebi-met1400-GCST90200073	ebi-IPs91-GCST90274803	Interleukin-18 levels	Weighted median	29	-0.06831	0.03382	0.04341	-0.134607	-0.00202	0.93397	0.87406	0.99798	0.977240101			
1-linoleoyl-2-arachidonoyl-GPC (18:2/20:4n6) levels	ebi-met1400-GCST90200073	ebi-IPs91-GCST90274803	Interleukin-18 levels	Weighted mode	29	-0.06234	0.03573	0.09199	-0.132361	0.00769	0.93957	0.87602	1.00772	0.995154017			
1-(1-enyl-stearoyl)-2-linoleoyl-GPE (p-18:0/18:2) levels	ebi-met1400-GCST90200076	ebi-IPs91-GCST90274803	Interleukin-18 levels	Inverse variance weighted	21	0.08598	0.03535	0.01502	0.0166839	0.15527	1.08978	1.01682	1.16798	0.55230638	0.620049728	0.193213282	0.754
1-(1-enyl-stearoyl)-2-linoleoyl-GPE (p-18:0/18:2) levels	ebi-met1400-GCST90200076	ebi-IPs91-GCST90274803	Interleukin-18 levels	MR Egger	21	0.12389	0.0834	0.15383	-0.039578	0.28735	1.13189	0.9612	1.33289	0.996820926			
1-(1-enyl-stearoyl)-2-linoleoyl-GPE (p-18:0/18:2) levels	ebi-met1400-GCST90200076	ebi-IPs91-GCST90274803	Interleukin-18 levels	Weighted median	21	0.04543	0.04769	0.3408	-0.048046	0.13891	1.04648	0.95309	1.14902	0.977240101			
1-(1-enyl-stearoyl)-2-linoleoyl-GPE (p-18:0/18:2) levels	ebi-met1400-GCST90200076	ebi-IPs91-GCST90274803	Interleukin-18 levels	Weighted mode	21	0.00142	0.0763	0.98534	-0.148131	0.15097	1.00142	0.86232	1.16296	0.996230303			
1-oleoyl-2-arachidonoyl-GPE (18:1/20:4) levels	ebi-met1400-GCST90200079	ebi-IPs91-GCST90274763	Caspase 8 levels	Inverse variance weighted	28	-0.04773	0.01943	0.01403	-0.085822	-0.00965	0.95339	0.91776	0.9904	0.55230638	0.577488594	0.3955485	0.8126667
1-oleoyl-2-arachidonoyl-GPE (18:1/20:4) levels	ebi-met1400-GCST90200079	ebi-IPs91-GCST90274763	Caspase 8 levels	MR Egger	28	-0.03173	0.03452	0.36642	-0.099399	0.03593	0.96876	0.90538	1.03658	0.996820926			
1-oleoyl-2-arachidonoyl-GPE (18:1/20:4) levels	ebi-met1400-GCST90200079	ebi-IPs91-GCST90274763	Caspase 8 levels	Weighted median	28	-0.05102	0.02691	0.05799	-0.103769	0.00173	0.95026	0.90143	1.00173	0.977240101			
1-oleoyl-2-arachidonoyl-GPE (18:1/20:4) levels	ebi-met1400-GCST90200079	ebi-IPs91-GCST90274763	Caspase 8 levels	Weighted mode	28	-0.05232	0.02214	0.0256	-0.095717	-0.00892	0.94903	0.90872	0.99112	0.995154017			
